# The Causal Relationship Between Multiple Modifiable Risk Factors and Hallux Valgus: A Two‐Sample Mendelian Randomization Study

**DOI:** 10.1002/fsn3.70965

**Published:** 2025-09-17

**Authors:** Siyi Liu, Wanqin Zheng, Haitao Chen, Ming Tu, Yongkang Zhong, Yihan Lou, Yinxian Wen, Liaobin Chen

**Affiliations:** ^1^ Division of Joint Surgery and Sports Medicine, Department of Orthopedic Surgery Zhongnan Hospital of Wuhan University Wuhan China; ^2^ Joint Disease Research Center Wuhan University Wuhan China

**Keywords:** hallux valgus, lifestyle, Mendelian randomization study, metabolic factor, risk factors

## Abstract

This Mendelian randomization (MR) study aimed to investigate the potential causal associations between modifiable lifestyle/metabolic factors and the risk of hallux valgus (HV), a common foot deformity characterized by lateral deviation of the great toe. We selected independent genetic variants strongly associated with five lifestyle factors (lifetime smoking index, smoking initiation, alcohol consumption, coffee intake, and vigorous physical activity) and ten metabolic traits (body mass index (BMI), waist‐hip ratio, type 2 diabetes, systolic/diastolic blood pressure, high/low‐density lipoprotein, apolipoprotein A‐1/B, and triglycerides) as instrumental variables through rigorous quality control (*R* ^2^ < 0.001, *p* ≤ 5 × 10^−8^). Genetic association estimates were derived from large‐scale genome‐wide association studies (GWAS) in the UK Biobank, GSCAN consortium, and FinnGen cohorts. A two‐sample MR approach was used to assess causal effects on HV risk. Genetically predicted smoking initiation (odds ratio (OR) 1.23, 95% confidence interval (CI) [1.06–1.43], *p* = 0.00784), lifetime smoking (OR 1.79, 95% CI [1.27–2.51] *p* = 0.000832), and higher BMI (OR 1.17, 95% CI [1.06–1.29] *p* = 0.00164) were significantly associated with increased HV risk. No significant associations were found for other tested factors. This study provides genetic evidence supporting a causal role of smoking and BMI in the development of HV. These findings highlight modifiable risk factors for targeted prevention strategies in HV management.

## Graphical abstract

This two‐sample Mendelian randomization study indicated the causal roles of genetically predicted smoking and BMI in the development of HV. Smoking and high BMI could increase the risk of HV, thus providing suggestions for early prevention and intervention.

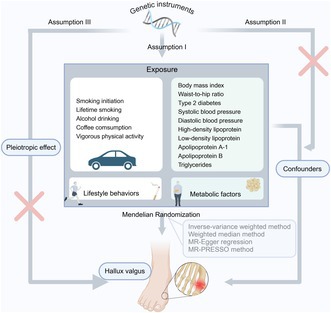

**Abbreviations**
BMI: body mass index
CI: confidence interval
GWAS: genome wide association studies
HDL: high density lipoprotein
HV: hallux valgus
IV: instrumental variable
IVW: inverse variance weighted
LDL: low density lipoprotein
MLA: medial longitudinal arch
MR: Mendelian randomization
OR: odds ratio
SNP: single nucleotide polymorphism
WM: weighted median

## 1 Introduction

Hallux valgus (HV) stands as one of the most prevalent foot deformities (Dunn et al. 2004), characterized primarily by the lateral deviation of the hallux at the first metatarsophalangeal joint exceeding the normal range (Ota et al. 2017). The degree of deviation varies, leading to varying degrees of symptoms in daily life, often manifested as restricted movement accompanied by pain, thereby diminishing patients' quality of life (Menz et al. 2011; Siefkas et al. 2022). Current surgical treatments, while diverse, demonstrate concerning complication rates (10%–50%) and patient dissatisfaction (25%–33%), underscoring the urgent need for effective prevention strategies (Faber et al. 2013; Ferrari et al. 2009; Raikin et al. 2014). The lengthy treatment and high surgical costs underscore the complexity of the disease's etiology and the potential for preventive strategies targeting modifiable factors. Thus, exploring the risk factors of HV offers valuable theoretical insights for reducing its occurrence and progression.

The etiology of HV remains incompletely understood. While observational studies have indicated that these lifestyle‐related factors, such as hyperlipidemia (Kane and Malloy 1990), hypertension (Elliott 2007; Narkiewicz 2006), diabetes (Antonopoulou et al. 2013), smoking (Goldenberg et al. 2014; Sleiman et al. 2014), alcohol consumption (Hendriks 2020), and obesity (Caballero 2019), all constitute risk exposures, the causal relationship between these factors and the risk of HV remains unclear. Investigating these lifestyle factors predisposing to HV could provide effective measures for primary prevention. Traditional observational approaches cannot overcome inherent limitations of confounding and reverse causality, while randomized trials of harmful exposures like smoking are ethically prohibitive. Consequently, the precise causal relationship between various modifiable risk factors and HV remains unclear.

Mendelian randomization (MR) (Sekula et al. 2016; Thomas and Conti 2004) is an important methodological structure utilized in epidemiology and genetics realms and is designed to infer causal relationships between exposure and outcome. This approach employs genetic variants tied to risk factors as instrumental variables (IVs) to estimate the causal effect of exposure on outcome (Birney 2022), and the natural random assortment of alleles during reproduction ensures minimal confounding in genetic associations. It aims to bolster causal conclusions from observational data, circumventing reverse causality biases, and yielding more robust causal estimates than conventional observational approaches (Davies et al. 2018; Davey Smith and Hemani 2014). Mendel's Second Law (Bowden and Holmes 2019), which posits that distinct genotypes yield specific intermediate phenotypes, MR leverages these phenotypes as proxies for exposure traits to gauge the influence of exposure on disease, sidestepping confounding and reverse causality often encountered in traditional epidemiology. In this study, an exhaustive MR analysis was undertaken on comprehensive GWAS (genome‐wide association studies) data. The primary aim was to uncover genetic variations that have a robust association with HV prolonged exposure to various factors.

This study aimed to employ MR analysis to examine the association between multiple modifiable risk factors and HV, to exposition the preventive and therapeutic effects of lifestyle habits on HV from a genetic perspective, thereby providing clinical evidence for HV prevention and treatment.

## 2 Materials and Methods

### 2.1 Study Design

**FIGURE 1:**
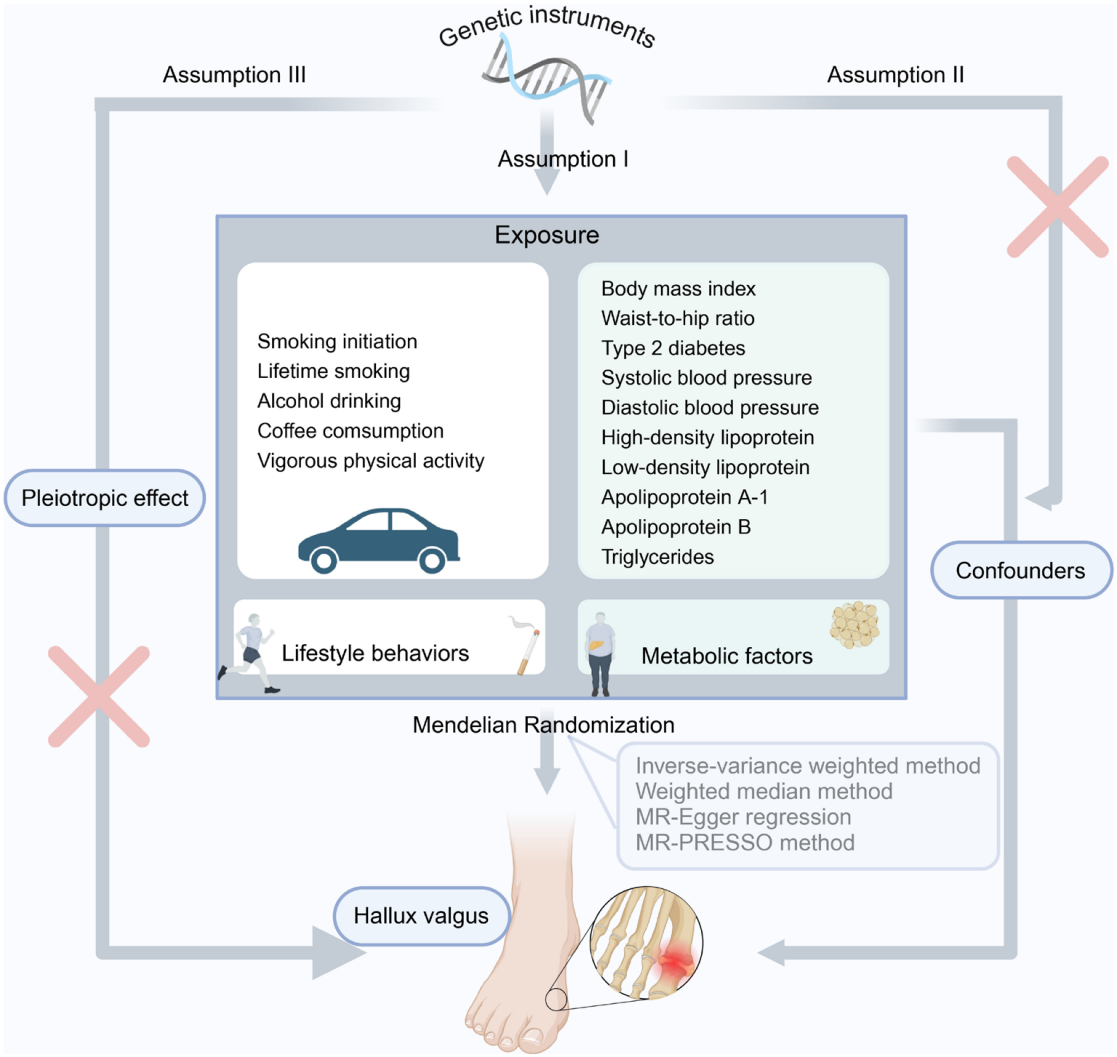
Study design overview (created in https://BioRender.com).

MR (Birney 2022) analysis constitutes an instrumental variable approach leveraging genetic variants as instruments. It relies on three pivotal assumptions (Wehby et al. 2008) (depicted in Figure 1). Firstly, the genetic variants designated as instrumental variables must exhibit a robust association with the exposure under investigation. Secondly, these genetic variants must be unconfounded, meaning they should not be associated with any factors that could potentially bias the exposure‐outcome relationship. Lastly, the selected genetic variants should exclusively influence the risk of the outcome through the mediation of the risk factor, precluding any alternative causal pathways. The current study is founded upon publicly accessible, summary‐level data derived from extensive GWAS and consortia. Our objective is to explore the causal relationship between modifiable risk factors and the risk of HV, aiming to provide insights into preventative measures. Single nucleotide polymorphisms (SNPs) associated with these risk factors were extracted as IVs; it incorporates 15 exposure factors, encompassing five adverse lifestyle factors and 10 metabolic factors. All studies included in referenced GWASs were approved by relevant review committees, and the process of our study was following the STROBE‐MR guidelines (Skrivankova et al. 2021); thus, separate ethical approval for this study was not required. Additionally, the STROBE‐MR Guidelines recommend reporting on the prespecified causal hypotheses, the justification for using MR as the study method, the measurement, quality, and selection of genetic variants, as well as any attempts to assess the validity of MR‐specific assumptions. All statistical analyses were performed using R (v4.3.1) using the TwoSampleMR (Hemani et al. 2018) (v0.5.7) and MRPRESSO (Verbanck et al. 2018) (v1.0) packages.

### 2.2 Genetic Instrument Selection

The data utilized in our study were sourced from published or publicly available GWASs, and all participating individuals had provided consent. We selected effective IV based on three fundamental assumptions of MR (Burgess et al. 2013). First, we set that each IV was significantly associated with exposure (*p* < 5 × 10^−8^) and removed those with linkage disequilibrium effects between SNPs (KB = 10,000, *R* ^2^ < 0.001). In addition, the PhenoScanner database is used to exclude SNPs associated with outcomes to eliminate confounders. Instrument strength was quantified using *F*‐statistics. The *F*‐statistic measures the ratio of the mean square of the model to the mean square of the error. The *F*‐statistic for univariable analyses was calculated using the formula $F=\frac{R^{2}\times \left(N-2\right)}{\left(1-R^{2}\right)}$ where $R^{2}=\frac{\left(2\times \beta^{2}\times \mathrm{EAF}\times \left(1-\mathrm{EAF}\right)\right)}{2\times \beta^{2}\times \left(1-\mathrm{EAF}\;\right)+2\times {\mathrm{SE}}^{2}\times N\times \mathrm{EAF}\times \left(1-\mathrm{EAF}\right)}$, *N* represents the number of participants, EAF represents the effect allele frequency, and *β* is the estimated effect of the SNP to assess its ability to uniquely predict the outcome (Gill et al. 2019; Levin et al. 2020; Palmer et al. 2012).

As presented in Table 1, a total of 15 potential risk factors were considered for analysis, categorized into lifestyle factors and metabolic traits. Lifestyle factors encompassed lifetime smoking (Liu et al. 2019), smoking initiation (Liu et al. 2019), alcohol drinking (Liu et al. 2019), coffee intake (Zhong et al. 2019), and vigorous physical activity (Klimentidis et al. 2018). Furthermore, we analyzed five lipid metabolism‐related traits and five obesity‐related characteristics. These included body mass index (BMI) (Pulit et al. 2019), waist‐to‐hip ratio (Locke et al. 2015), type 2 diabetes (Sakaue et al. 2021), systolic blood pressure, diastolic blood pressure (Evangelou et al. 2018) high‐density lipoprotein (HDL), low‐density lipoprotein (LDL), apolipoprotein A‐1, apolipoprotein B, and triglycerides (Richardson et al. 2020).

**TABLE 1:** Sources of GWAS data for instrumental variables.

Exposure or outcome	Unit	Population	Sample size	Adjustments	IVs	PubMed ID
Lifestyle factor						
Smoking initiation	SD in prevalence of smoking initiation	European	1,232,091	Age, sex, and the first 10 genetic principal components	89	30643251
Lifetime smoking index	SD change of lifetime smoking index	European	462,690	Genotyping chip and sex	125	30643251
Alcohol drinking	SD increase of log‐transformed alcoholic drinks/week	European	941,280	Age, sex, and the first 10 genetic principal components	36	30643251
Coffee consumption	50% change cup of coffee	European	375,833	Age, sex, body mass index, total energy, proportion of typical food intake, and 20 genetic principal components	27	31046077
Vigorous physical activity	≥ 3 versus 0 day/week	European	98,060 cases and 162,995 controls	Age, sex, genotyping chip, first 10 genomic principal components, and center	6	29899525
Metabolic factor						
Body mass index	SD	European	694,649	Age, sex, and genetic 1–5 principal components	532	30239722
Waist‐to‐hip ratio	SD	European	224,459	Age and study‐specific covariates	19	25673412
Type 2 diabetes	One‐unit in log‐transformed odds	European	148,726 cases and 965,732 controls	Age, sex, and the first 10 genetic principal components	232	32541925
Systolic blood pressure	10 mmHg	European	757,601	Age, sex, BMI, genotyping chip	446	30224653
Diastolic blood pressure	10 mmHg	European	757,601	Age, sex, and genotyping chips	446	30224653
High‐density lipoprotein	SD	European	403,943	Age, sex, and genotyping chips	292	32203549
Low‐density lipoprotein	SD	European—	440,546	Age, sex, and genotyping chips	139	32203549
Apolipoprotein A‐1	SD	European	393,193	Age, sex, and genotyping chips	252	32203549
Apolipoprotein B	SD	European	439,214	Age, sex, and genotyping chips	160	32203549
Triglycerides	SD	European	441,016	Age, sex, and genotyping chips	244	32203549
Outcome						
Hallux valgus	Odds ratio	European	15,886 cases and 262,844 controls	Age, sex, and genotyping chips	—	—

Abbreviations: GWAS, Genome wide association studies; IV, instrumental variable; SD, standard deviation.

### 2.3 GWAS Data for HV

In this study, HV was selected as the outcome, and its GWAS data were obtained from the FinnGen database (https://r10.finngen.fi/). This database employs ICD (International Classification of Diseases)‐10 diagnostic codes, where HV is designated by the code “M13_HALLUXVALGUS.” The analysis included 278,730 individuals of European ancestry, comprising 15,886 cases and 262,844 controls. The FinnGen database last updated this data on December 18, 2023, further enhancing the credibility of our research findings. Details of the data are presented in Table 1.

### 2.4 Statistical Analysis

In this two‐sample MR study, we utilized three complementary approaches to corroborate the causal linkage between modifiable risk factors and HV: the inverse variance weighted (IVW) method (primary approach), MR‐Egger regression, and the weighted median (WM) estimator. The IVW method assumes that all instrumental variables are valid and provides the most accurate estimates based on the calculation of Wald ratios. MR‐Egger regression accommodates pleiotropic effects across SNPs, detects horizontal pleiotropy via an intercept test, and presents adjusted estimates after pleiotropy correction. The WM estimator, on the other hand, assigns weights to the causal effect magnitudes of each SNP pair proportional to the SNP count within clusters, yielding a provisional estimate weighted by the maximum SNP count. Our findings are conveyed as odds ratios (ORs) accompanied by 95% confidence intervals (95% CIs), and statistical significance is ascribed to *p* < 0.05.

### 2.5 Sensitivity Analysis

Cochrane's *Q* was used as a heterogeneity test; *p* < 0.05 represents the existence of heterogeneity. MR‐Egger regression was used to detect horizontal pleiotropy. The intercept value of MR‐Egger regression represents the strength of horizontal pleiotropy, and a *p* > 0.05 means that there is no horizontal pleiotropy. MR‐PRESSO was used to detect SNPs that may lead to pleiotropic effects, remove outlier SNPs, and then perform MR analysis to compare whether the results have changed before and after correction. This method gradually eliminated a single SNP and performed MR analysis on the remaining SNPs to detect whether the single SNP had a significant impact on the results.

## 3 Results

**FIGURE 2:**
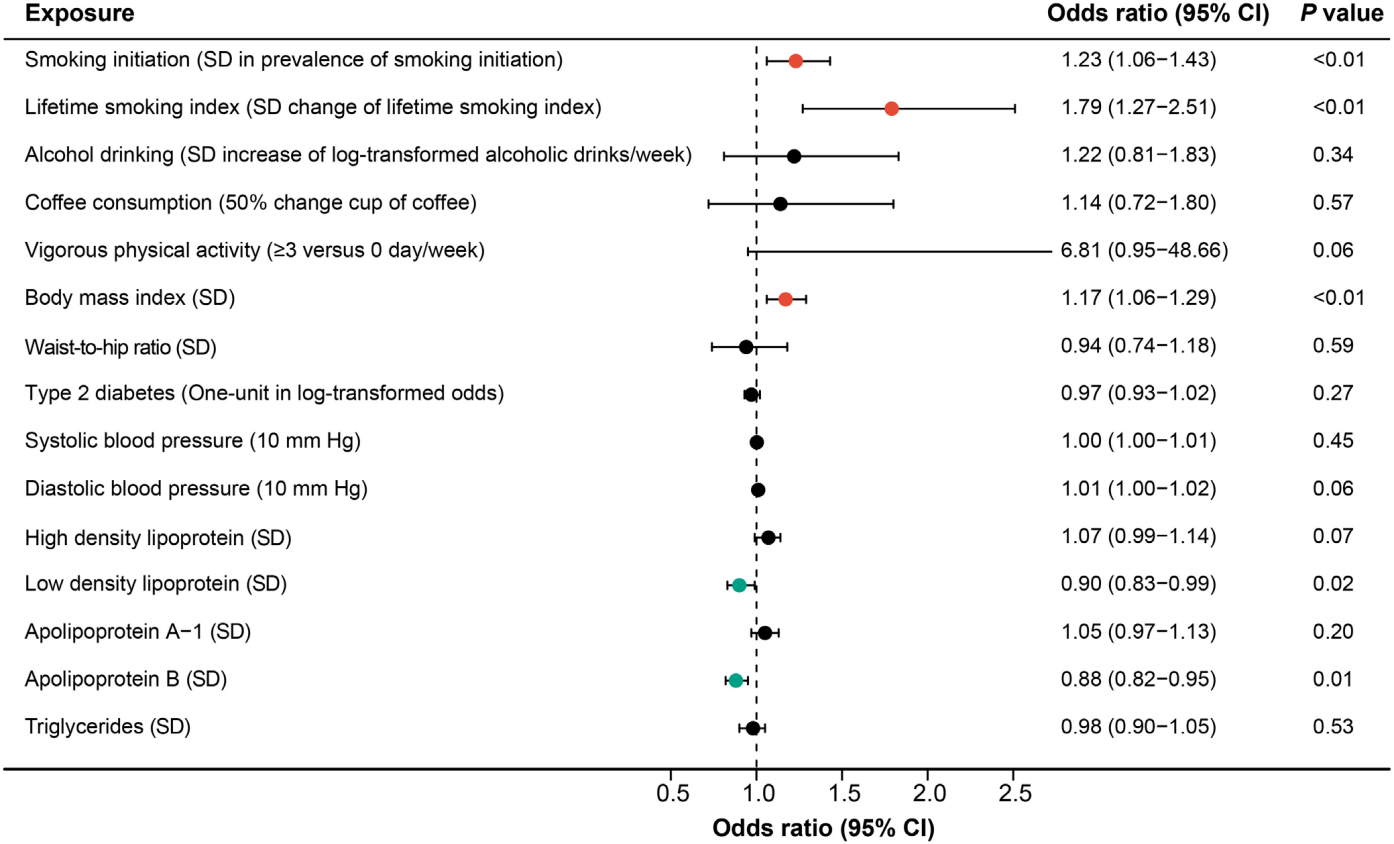
Mendelian randomization estimates for the effects of lifestyle and metabolic factors on hallux valgus. CI, confidence interval; SD, standard deviation.

The MR results are summarized in Figure 2 and Tables 2 and 3. Two‐sample MR analysis was conducted to evaluate the causal effects of five lifestyle and ten metabolic factors on HV, which utilizes genetic instruments derived from independent exposure and outcome datasets. The number of SNPs was selected as valid IVs in lifestyle and metabolic factors after quality control. No significant genetic correlation was observed between 10 out of 15 potential risk factors and HV (except LDL and apolipoprotein B, which has a significant horizontal pleiotropy with HV) in the IVW method. Univariable MR Analysis showed that in the lifestyle factors, genetically predicted smoking initiation (OR 1.23, 95% CI [1.06–1.43], *p* = 0.00784) and lifetime smoking index (OR 1.79, 95% CI [1.27–2.51], *p* = 0.000832) were associated with an increased risk of HV (Figure 2, Table 2). As for the lifetime smoking index, the results from the weighted median method were consistent (OR 1.85, 95% CI [1.25–2.73], *p* = 0.00203) with the results of the IVW method. The MR‐PRESSO test also yielded a similar result in predicted smoking initiation (OR 1.19, 95% CI [1.03–1.39], *p* = 0.0217) and lifetime smoking (OR 1.63, 95% CI [1.15–2.29], *p* = 0.00625). Among metabolic factors, higher BMI was associated with an increased risk of HV (OR 1.17, 95% CI [1.06–1.29], *p* = 0.00164). The results from the MR‐PRESSO test were consistent (OR 1.20, 95% CI [1.09–1.31], *p* = 0.000219) (Figure 2, Table 3).

**TABLE 2:** MR estimates from different methods of assessing the causal effect of lifestyle factors on hallux valgus.

Exposure	Method	Beta	OR	95% CI	*p*	Cochran's *Q* (*p*)	Egger_Intercept (*p*)	Outlier
Smoking initiation	MR‐Egger	0.179	1.20	0.55–2.61	0.65	7.64e‐05	0.0008 (0.94)	4
	Weighted‐Median	0.157	1.17	0.98–1.40	0.09			
	IVW	0.208	1.23	1.06–1.43	< 0.01	1.02e‐04		
	MR‐PRESSO	0.178	1.19	1.03–1.39	0.02			
Lifetime smoking index	MR‐Egger	0.549	1.73	0.42–7.16	0.45	2.11e‐09	0.0003 (0.96)	1
	Weighted‐Median	0.614	1.85	1.25–2.73	< 0.01			
	IVW	0.581	1.79	1.27–2.51	< 0.01	2.99e‐09		
	MR‐PRESSO	0.487	1.63	1.15–2.29	< 0.01			
Alcohol drinking	MR‐Egger	−0.225	0.80	0.30–2.11	0.65	1.24e‐03	0.0076 (0.35)	0
	Weighted‐Median	0.277	1.32	0.83–2.11	0.25			
	IVW	0.199	1.22	0.81–1.83	0.34	1.14e‐03		
	MR‐PRESSO	0.199	1.22	0.81–1.83	0.34			
Coffee consumption	MR‐Egger	−0.345	0.71	0.30–1.69	0.44	4.46e‐03	0.0095 (0.22)	1
	Weighted‐Median	−0.040	0.96	0.60–1.53	0.87			
	IVW	0.131	1.14	0.72–1.80	0.57	2.88e‐03		
	MR‐PRESSO	0.131	1.14	0.72–1.80	0.58			
Vigorous physical activity	MR‐Egger	2.167	8.73	9.03e‐16–8.45e+16	0.91	6.63e‐03	−0.0022 (0.99)	1
	Weighted‐Median	2.147	8.56	1.61–45.54	0.01			
	IVW	1.918	6.81	0.95–48.66	0.06	1.43e‐02		
	MR‐PRESSO	1.918	6.81	0.95–48.66	0.11			

Abbreviations: IVW, inverse variance weighted; MR, Mendelian randomization.

**TABLE 3:** MR estimates from different methods of assessing the causal effect of metabolic factors on hallux valgus.

Exposure	Method	Beta	OR	95% CI	*p*	Cochran's *Q* (*p*)	Egger_Intercept (*p*)	Outlier
Body mass index	MR‐Egger	0.063	1.06	0.81–1.40	0.65	9.44e‐18	0.0016 (0.48)	5
	Weighted‐Median	0.116	1.12	0.99–1.28	0.08			
	IVW	0.155	1.17	1.06–1.29	< 0.01	1.03e‐17		
	MR‐PRESSO	0.178	1.20	1.09–1.31	< 0.01			
Waist‐to‐hip ratio	MR‐Egger	−0.087	0.92	0.26–3.17	0.89	0.0470	0.0007 (0.97)	4
	Weighted‐Median	−0.175	0.84	0.64–1.10	0.20			
	IVW	−0.065	0.94	0.74–1.18	0.59	0.0647		
	MR‐PRESSO	−0.065	0.94	0.74–1.18	0.59			
Type 2 diabetes	MR‐Egger	−0.106	0.90	0.82–0.99	0.03	7.90e‐10	0.0049 (0.07)	3
	Weighted‐Median	−0.082	0.92	0.87–0.98	< 0.01			
	IVW	−0.027	0.97	0.93–1.02	0.27	3.06e‐10		
	MR‐PRESSO	−0.027	0.97	0.93–1.02	0.25			
Systolic blood pressure	MR‐Egger	0.007	1.01	0.99–1.02	0.32	1.30e‐18	−0.0017 (0.45)	4
	Weighted‐Median	0.006	1.01	1.00–1.01	0.17			
	IVW	0.002	1.00	1.00–1.01	0.45	1.39e‐18		
	MR‐PRESSO	0.002	1.00	1.00–1.01	0.51			
Diastolic blood pressure	MR‐Egger	0.012	1.01	0.99–1.04	0.34	1.34e‐24	−0.0005 (0.84)	6
	Weighted‐Median	0.012	1.01	1.00–1.03	0.08			
	IVW	0.010	1.01	1.00–1.02	0.06	1.81e‐24		
	MR‐PRESSO	0.011	1.01	1.00–1.02	0.03			
High‐density lipoprotein	MR‐Egger	0.039	1.04	0.94–1.16	0.47	1.43e‐05	0.0010 (0.53)	2
	Weighted‐Median	0.079	1.08	0.97–1.21	0.16			
	IVW	0.064	1.07	0.99–1.14	0.07	1.57e‐05		
	MR‐PRESSO	0.056	1.06	0.99–1.13	0.11			
Low‐density lipoprotein	MR‐Egger	−0.009	0.99	0.88–1.12	0.89	1.16e‐06	−0.0051 (0.04)	2
	Weighted‐Median	−0.100	0.90	0.81–1.01	0.06			
	IVW	−0.100	0.90	0.83–0.99	0.02	3.39e‐07		
	MR‐PRESSO	−0.095	0.91	0.83–0.99	0.03			
Apolipoprotein A‐1	MR‐Egger	0.063	1.07	0.95–1.20	0.29	7.72e‐06	−0.0006 (0.75)	2
	Weighted‐Median	−0.001	1.00	0.90–1.11	0.99			
	IVW	0.048	1.05	0.97–1.13	0.20	9.14e‐06		
	MR‐PRESSO	0.039	1.04	0.97–1.12	0.30			
Apolipoprotein B	MR‐Egger	−0.036	0.96	0.87–1.07	0.48	6.87e‐04	−0.0052 (0.01)	2
	Weighted‐Median	−0.085	0.92	0.82–1.03	0.14			
	IVW	−0.126	0.88	0.82–0.95	0.01	1.97e‐04		
	MR‐PRESSO	−0.122	0.89	0.82–0.96	< 0.01			
Triglycerides	MR‐Egger	−0.019	0.98	0.88–1.10	0.74	9.22e‐11	−0.0003 (0.89)	4
	Weighted‐Median	−0.0001	1.00	0.90–1.11	1.00			
	IVW	−0.025	0.98	0.90–1.05	0.53	1.20e‐10		
	MR‐PRESSO	−0.020	0.98	0.91–1.06	0.61			

Abbreviations: IVW, inverse variance weighted; MR, Mendelian randomization.

The *F* statistic for instruments and estimated power for all analyses are shown in Table S1. The *F* statistic of all SNPs used as IV was greater than 10, indicating that a good strength of the used genetic instruments. We detected possible pleiotropy in the analyses for apolipoprotein B and LDL after the removal of outlier variants in the MR‐PRESSO analysis (Table 3). Cochran's *Q* test showed heterogeneity in exposure–smoking initiation and other results (*p* < 0.05). Therefore, the IVW method with multiplicative random effects was used in the evaluation of causal effects. The funnel plots for visualizing the heterogeneity are displayed in Figure S1. The scatter plots of the SNP–exposure association against the SNP–outcome association are presented in Figure S2.

## 4 Discussion

When compared to no treatment or non‐surgical alternatives, surgical intervention for HV may result in a clinically meaningful reduction in pain. Yet, the difference in quality of life between surgical intervention and either no treatment or non‐surgical management may be scarcely perceptible or nonexistent (Torkki et al. 2001). Reports have indicated recurrence or undercorrection in 10% to 14% of cases (Kayali et al. 2008), highlighting the limited evidence available for the efficacy of both surgical and conservative treatments for HV. Given the progressive nature and the absence of definitive treatments to slow or halt its progression, exploring the causal relationship between modifiable risk factors and HV is crucial for prevention and management. MR (Davies et al. 2018) is an analytical approach that harnesses genetic variants as instrumental variables to investigate modifiable risk factors impacting population health. A notable advantage of MR is its reduced susceptibility to confounding factors or reverse causation, a feature that distinguishes it from traditional observational studies which may be more prone to such biases. Ference et al. (2019) utilized MR analysis to uncover a causal relationship between the inhibition of ATP citrate lyase and an increased risk of cardiovascular events, offering novel perspectives on the prevention and treatment of cardiovascular diseases. Turner and Staplin (2021) adopted the bidirectional MR analysis that supported renal function as a cause, rather than a consequence, of blood pressure variation, thereby providing strong evidence for the causal link between hypertension and renal disease and informing clinical treatment and prevention strategies. At present, the association between modifiable risk factors (lifestyle and metabolic factors) and the risk of HV remains unclear; therefore, the aims of this MR study were to evaluate the relationship between these modifiable factors and HV, adjusting for potential confounding factors such as gender, age, and definition of HV. This MR study revealed that smoking initiation, lifetime smoking, and BMI were associated with an elevated risk of HV. However, the precise biological mechanisms underlying the positive causal relationship between smoking behaviors, BMI indices, and HV remain elusive.

Smoking, a ubiquitous risk factor for disease progression worldwide, poses a significant public health challenge. According to the World Health Organization, smoking is projected to cause approximately 1 billion deaths globally this century, with over 8 million deaths annually, most of which occur in low‐ and middle‐income countries. China, as the largest tobacco consumer, accounts for nearly 40% of global tobacco use, with one‐third of the world's male smokers residing there (Wong et al. 2007). Smoking rates in China continue to rise, with a declining average age of smoking initiation across successive birth cohorts and an increase in the number of daily smokers. Tobacco smoke contains over 7000 chemical compounds, including at least 69 carcinogens, with nicotine being the primary psychoactive substance and a vital regulator of bone metabolism (Le Foll et al. 2022). Individuals with a genetic predisposition to initiate smoking were found to have an elevated risk of fractures, osteoarthritis, and rheumatoid arthritis. Furthermore, the findings concerning lifelong smoking were in agreement with those observed for smoking initiation (Larsson and Burgess 2022). Extensive experimental research underscores the sensitivity of bone to smoking and its crucial biological role in bone tissue and cartilage. Smoking downregulates serum vitamin D levels and inhibits parathyroid hormone production, essential for regulating serum calcium levels through bone and renal reabsorption. This, in turn, disrupts calcium absorption in the intestines, leading to bone loss. Additionally, nicotine and cotinine (Le Foll et al. 2022) suppress aromatase activity, reducing estrogen levels and promoting osteoclast differentiation and bone resorption. Considering that osteophyte formation is a concomitant clinical symptom of HV, the significant correlation between cartilage degeneration in the first metatarsophalangeal joint and the severity of HV suggests that smoking‐mediated cartilage degeneration may contribute to HV development. Thus, smoking, as an exposure factor, could potentially facilitate the onset of HV, providing a novel perspective on the etiology of this condition. Our research offers a fresh insight into the etiology of HV, suggesting that smoking may play a role in its development.

Obesity (Arias‐Martin et al. 2018), a global health crisis affecting over 502 million individuals worldwide (Finucane et al. 2011), has witnessed a striking surge in its prevalence across both developed and developing nations in recent years, posing a significant threat to both individual and public health. Defined as excessive weight gain due to excessive body fat accumulation, obesity is typically measured using the body mass index (BMI). According to data from the National Health and Nutrition Examination Survey conducted in 2009 to 2010, it was reported that 69% of adults aged 20 and older are classified as overweight (BMI ≥ 25) (Flegal et al. 2012). Furthermore, recent studies have indicated that adults who are overweight or obese are more prone to experiencing foot pain, flat feet, and elevated peak plantar pressures during walking compared to their normal‐weight counterparts (Butterworth et al. 2016; Tanamas et al. 2012). It indicates that obesity is a risk factor for foot disorders, particularly musculoskeletal issues affecting the lower limbs and feet (McGoey et al. 1990). According to the study conducted by Martín‐Casado et al. (2023), the primary risk factors for the development of HV are age, followed by heel width and foot length. Their findings reveal that overweight and obese children exhibit longer and wider feet compared to their normal‐weight counterparts. As the foot increases in length and width, which implies an augmentation in bearing forces, particularly on the rear foot, leading to a medial displacement of the subtalar joint axis during movement, hindering the optimal motion of the metatarsophalangeal joint during the toe‐off phase of gait, thereby the risk of HV injury escalates. Studies have demonstrated a robust correlation between an elevated BMI and chronic plantar heel pain within a non‐athletic demographic (Butterworth et al. 2012). Increased BMI or overweight individuals experience morphological changes in the feet due to mechanical loading and metabolic effects, causing ligament and support structure collapse, which can lead to HV. These individuals often have larger foot volumes and reduced medial longitudinal arch (MLA) height compared to those with normal weight, who tend to have narrower feet. A reduction or absence of MLA is among the most relevant orthopedic issues associated with overweight. There is a direct correlation between weight gain and a higher prevalence of flat feet, which, in turn, is linked to conditions such as adult HV or HV deformity development. A population‐based study in Denmark (*n* = 2179) reported a significantly higher incidence of foot pain among individuals with higher BMI. Specifically, obese males were three times more likely to experience foot pain than those with normal BMI. In a sample of 563 individuals from rural communities in Korea, Cho et al. (2009) found that HV patients had higher BMI. Similarly, Nguyen et al.'s (2010) population‐based study of 600 elderly individuals reported an increased likelihood of HV among women who wore high heels between the ages of 20 and 64 years, compared to those who did not. Among males, those with a BMI between 25.0 and 29.9 had an increased risk of HV compared to those with normal BMI. In this study, we found an association between higher BMI and increased risk of HV, providing a novel perspective on the prevention of HV.

These findings above align with our study's observations. As integral components of both the environment and an individual's makeup, lifestyle and metabolic factors undoubtedly influence HV development. Yet, the existence and extent of genetic causal associations between specific indicators such as smoking, alcohol consumption, BMI, and HV remain unclear at the genetic level. Our study sheds light on these relationships by analyzing and integrating various HV research, combined with past insights into genetic causal effects. Therefore, our findings contribute to guiding clinical discovery and validating novel disease prevention and treatment options to improve HV outcomes. Ultimately, our results suggest that individuals predisposed to or suffering from HV should consider smoking cessation and weight reduction. However, the interpretation of our findings must acknowledge the study's limitations. Firstly, the generalizability of our results may be constrained by the limited ancestral diversity of the available GWAS data, which are predominantly from European‐ancestry cohorts. Secondly, as the magnitude of the OR value reflects a lifetime association rather than a specific age range, further research is required to investigate how the timing of these risk factors differs among individuals with HV. Finally, this study may be subject to some “healthy participant” bias, as the volunteers participating in the study tend to have higher socioeconomic status and be healthier. This could potentially lead to some bias in the estimation of effect sizes.

## 5 Conclusion

The present study provides MR data in support of the causal roles of genetically predicted smoking and BMI in the development of HV. Smoking and high BMI could increase the risk of HV, thus providing suggestions for early prevention and intervention. However, the results of this study need to be further confirmed by basic experiments.

## Author Contributions

**Siyi Liu:** conceptualization (lead), data curation (lead), formal analysis (lead), investigation (lead), methodology (lead), project administration (equal), resources (lead), software (equal), visualization (equal), writing – original draft (equal), writing – review and editing (equal). **Wanqin Zheng:** formal analysis (equal), visualization (equal), writing – original draft (equal), writing – review and editing (equal). **Haitao Chen:** data curation (equal), formal analysis (equal), investigation (equal). **Ming Tu:** data curation (equal), formal analysis (equal), investigation (equal). **Yongkang Zhong:** data curation (equal), formal analysis (equal), investigation (equal). **Yihan Lou:** data curation (equal), formal analysis (equal), investigation (equal). **Yinxian Wen:** conceptualization (equal), data curation (equal), funding acquisition (supporting), investigation (equal), methodology (equal), project administration (equal), supervision (equal), writing – review and editing (equal). **Liaobin Chen:** conceptualization (equal), funding acquisition (lead), project administration (equal), supervision (equal), writing – review and editing (equal).

## Ethics Statement

All experiments involving humans were conducted according to the ethical policies and procedures approved by our institution. Ethical clearance was granted for all selected GWAS studies, and informed consent was obtained from participating individuals.

## Conflicts of Interest

The authors declare no conflicts of interest.

## Supporting information


**Data S1:** fsn370965‐sup‐0001‐DataS1.docx.

## Data Availability

The original contributions presented in the study are included in the article/Supporting Information; further inquiries can be directed to the corresponding author.
