# Supplementary material for: The Causal Relationship Between Multiple Modifiable Risk Factors and Hallux Valgus: A Two‐Sample Mendelian Randomization Study
Source: Food Sci Nutr. 2025 Sep 17;13(9):e70965. doi: 10.1002/fsn3.70965 (PMC12441740; doi:10.1002/fsn3.70965)
Supplement: Supplementary file 1 — Data S1: fsn370965‐sup‐0001‐DataS1.docx. [file FSN3-13-e70965-s001.docx]

**Supplementary material**

**The causal relationship between** **multiple** **modifiable risk factors and** **hallux valgus: A two-sample Mendelian randomization study**

**Figure S1 Funnel plot of the** **multiple modifiable factors in the hallux valgus GWAS.** The heterogeneity of multiple modifiable factors is quantified using Cochrane Q test, and then visualized using funnel plot. GWAS: genome-wide association study

**Figure S2** **Scatter plot of the multiple modifiable factors in the hallux valgus GWAS.** GWAS: genome-wide association study

**Table S1 Information of the instrumental variables**


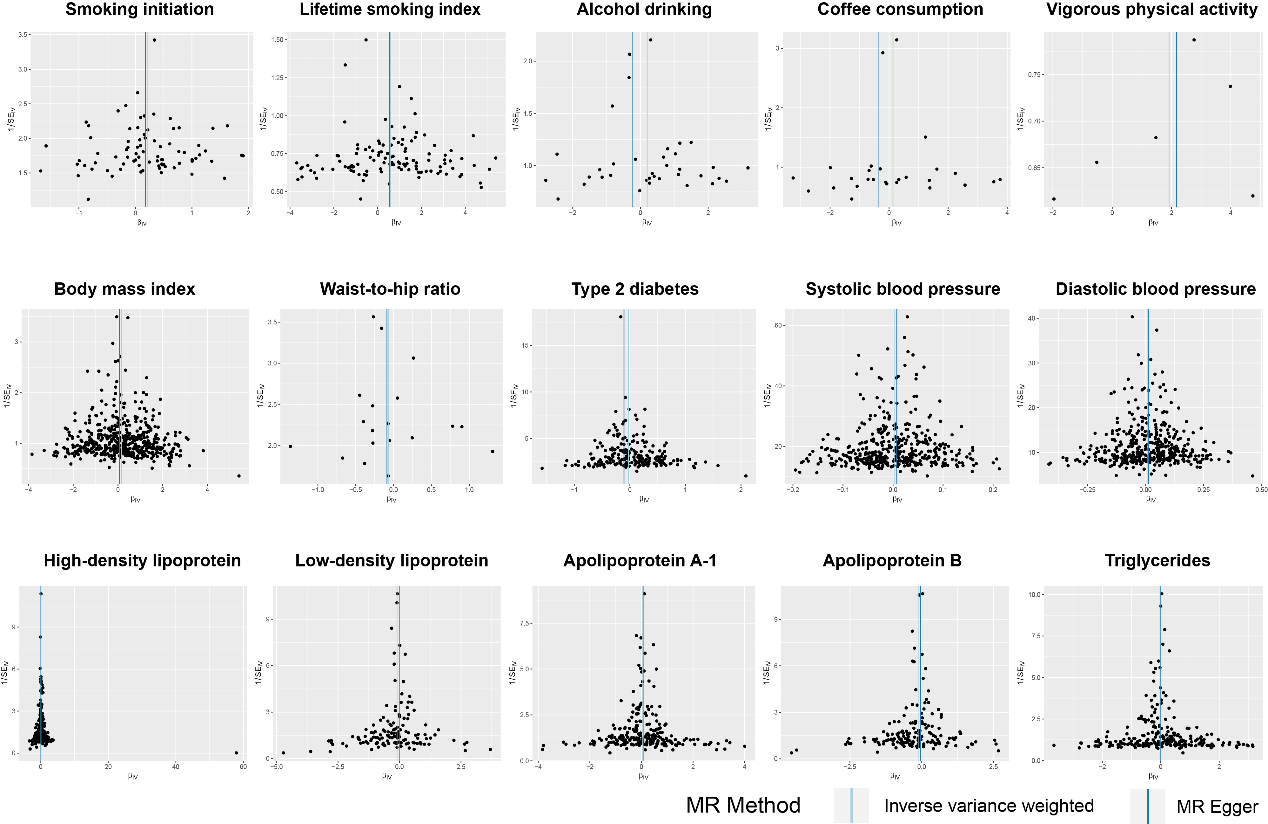


**Figure S1 Funnel plot of the multiple modifiable factors in the hallux valgus GWAS.** The heterogeneity of multiple modifiable factors is quantified using Cochrane Q test, and then visualized using funnel plot. GWAS: genome-wide association study


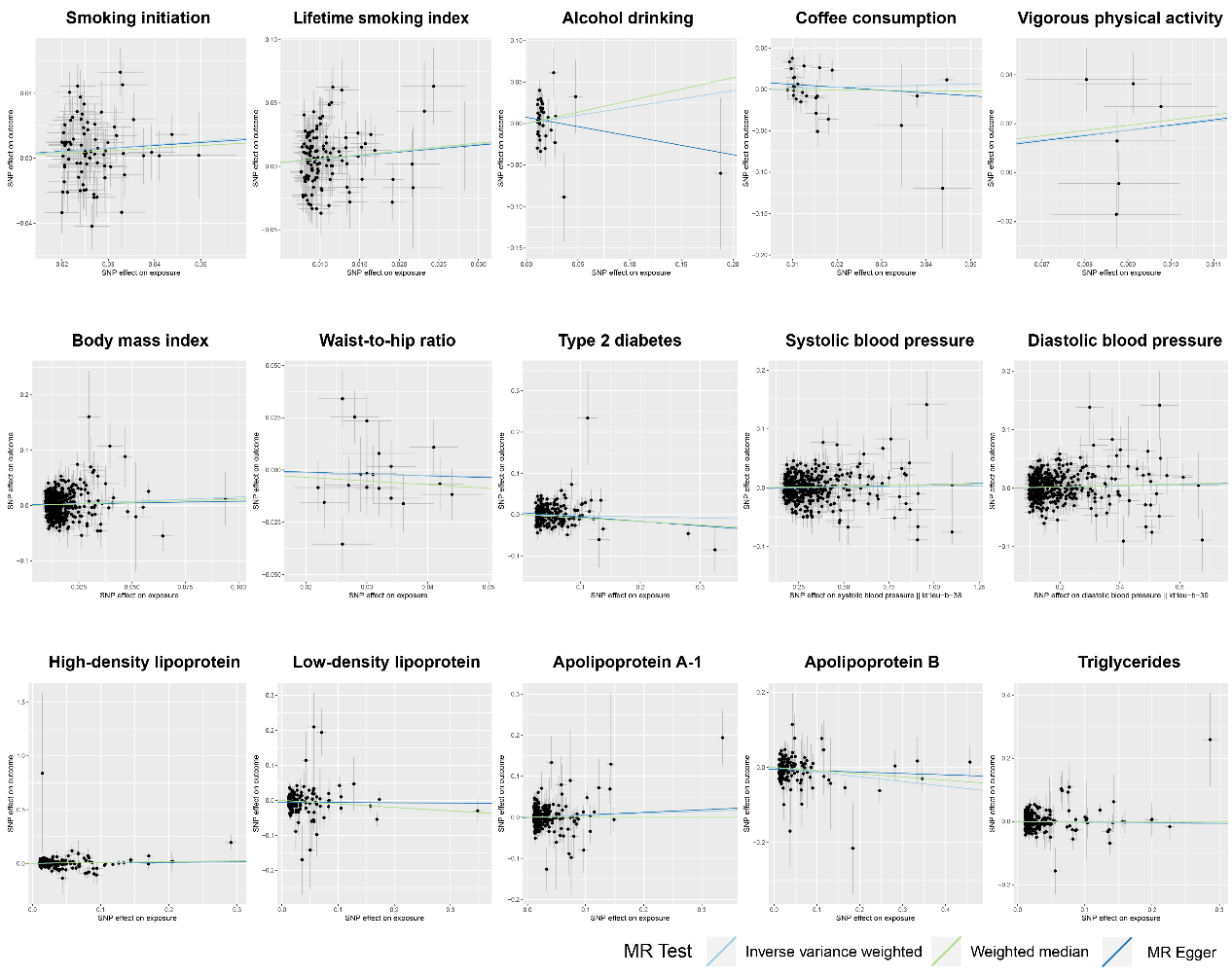


**Figure S2** **Scatter plot of the multiple modifiable factors in the hallux valgus GWAS.** GWAS: genome-wide association study

| **Table S1 Information of the instrumental variables** | | | | |  |  |  |  |  |
| --- | --- | --- | --- | --- | --- | --- | --- | --- | --- |
| Exposure | SNP | A1 | A2 | beta | eaf | pval | se | R2 | F-statistic |
| Smoking initiation | rs10001365 | A | G | -0.0249918 | 0.405 | 6.65E-12 | 3.64E-03 | 3.82E-05 | 47.10 |
| Smoking initiation | rs1004787 | A | G | 0.0299231 | 0.581 | 5.27E-17 | 3.57E-03 | 5.70E-05 | 70.20 |
| Smoking initiation | rs10114490 | A | G | -0.0255148 | 0.198 | 1.81E-08 | 4.53E-03 | 2.57E-05 | 31.70 |
| Smoking initiation | rs10159545 | G | C | 0.0262501 | 0.375 | 1.84E-12 | 3.73E-03 | 4.03E-05 | 49.60 |
| Smoking initiation | rs10233018 | G | A | 0.027069 | 0.503 | 2.75E-14 | 3.56E-03 | 4.70E-05 | 57.90 |
| Smoking initiation | rs10260968 | A | G | -0.0203218 | 0.597 | 1.75E-08 | 3.61E-03 | 2.57E-05 | 31.70 |
| Smoking initiation | rs10279261 | A | G | -0.0214194 | 0.619 | 5.00E-09 | 3.66E-03 | 2.78E-05 | 34.20 |
| Smoking initiation | rs10498846 | T | C | 0.0206103 | 0.473 | 6.62E-09 | 3.56E-03 | 2.73E-05 | 33.60 |
| Smoking initiation | rs1050847 | T | C | -0.0216231 | 0.505 | 1.67E-09 | 3.59E-03 | 2.95E-05 | 36.30 |
| Smoking initiation | rs10905461 | C | T | -0.0239554 | 0.718 | 7.35E-09 | 4.15E-03 | 2.71E-05 | 33.40 |
| Smoking initiation | rs10956809 | C | G | -0.0207811 | 0.442 | 6.32E-09 | 3.58E-03 | 2.74E-05 | 33.70 |
| Smoking initiation | rs11057005 | G | A | -0.0209298 | 0.43 | 4.85E-09 | 3.58E-03 | 2.78E-05 | 34.20 |
| Smoking initiation | rs11078713 | G | A | -0.0201721 | 0.454 | 2.23E-08 | 3.61E-03 | 2.54E-05 | 31.30 |
| Smoking initiation | rs1154693 | G | A | 0.0326217 | 0.856 | 3.12E-11 | 4.91E-03 | 3.58E-05 | 44.10 |
| Smoking initiation | rs1160685 | G | C | 0.0207724 | 0.478 | 7.20E-09 | 3.59E-03 | 2.72E-05 | 33.50 |
| Smoking initiation | rs11712680 | C | A | -0.0270476 | 0.174 | 3.51E-09 | 4.58E-03 | 2.83E-05 | 34.90 |
| Smoking initiation | rs117143374 | C | T | 0.0292897 | 0.12 | 2.76E-08 | 5.27E-03 | 2.51E-05 | 30.90 |
| Smoking initiation | rs11721059 | T | C | 0.0199357 | 0.474 | 2.17E-08 | 3.56E-03 | 2.54E-05 | 31.30 |
| Smoking initiation | rs11768481 | A | C | -0.0232017 | 0.347 | 7.00E-10 | 3.76E-03 | 3.08E-05 | 38.00 |
| Smoking initiation | rs11872397 | A | G | -0.0247725 | 0.252 | 1.43E-09 | 4.09E-03 | 2.97E-05 | 36.60 |
| Smoking initiation | rs12027999 | C | T | -0.0330855 | 0.124 | 5.76E-10 | 5.34E-03 | 3.12E-05 | 38.40 |
| Smoking initiation | rs12042107 | C | T | -0.0222834 | 0.527 | 4.22E-10 | 3.57E-03 | 3.17E-05 | 39.00 |
| Smoking initiation | rs12112638 | G | A | -0.024526 | 0.275 | 1.34E-09 | 4.04E-03 | 2.99E-05 | 36.80 |
| Smoking initiation | rs12186738 | T | G | -0.0332644 | 0.154 | 3.42E-11 | 5.02E-03 | 3.56E-05 | 43.90 |
| Smoking initiation | rs12333760 | C | T | -0.0290467 | 0.204 | 1.44E-09 | 4.80E-03 | 2.97E-05 | 36.60 |
| Smoking initiation | rs12356821 | C | G | 0.03937 | 0.14 | 6.27E-15 | 5.05E-03 | 4.93E-05 | 60.80 |
| Smoking initiation | rs12441907 | A | C | -0.0292051 | 0.186 | 1.06E-10 | 4.52E-03 | 3.38E-05 | 41.70 |
| Smoking initiation | rs12474587 | T | G | 0.0276329 | 0.404 | 1.25E-14 | 3.58E-03 | 4.83E-05 | 59.50 |
| Smoking initiation | rs12545053 | G | A | 0.0202808 | 0.397 | 2.43E-08 | 3.64E-03 | 2.52E-05 | 31.10 |
| Smoking initiation | rs12632110 | G | A | -0.0233768 | 0.647 | 4.78E-10 | 3.75E-03 | 3.15E-05 | 38.80 |
| Smoking initiation | rs12923427 | T | C | -0.0239087 | 0.204 | 4.44E-08 | 4.37E-03 | 2.43E-05 | 29.90 |
| Smoking initiation | rs13145728 | C | G | -0.0232512 | 0.358 | 2.14E-10 | 3.66E-03 | 3.27E-05 | 40.30 |
| Smoking initiation | rs13246563 | G | C | -0.0234631 | 0.526 | 3.45E-10 | 3.74E-03 | 3.20E-05 | 39.40 |
| Smoking initiation | rs13261666 | T | G | -0.0268946 | 0.522 | 3.90E-14 | 3.56E-03 | 4.64E-05 | 57.20 |
| Smoking initiation | rs134529 | C | T | -0.019984 | 0.349 | 4.85E-08 | 3.66E-03 | 2.42E-05 | 29.80 |
| Smoking initiation | rs1385108 | T | C | 0.0246617 | 0.239 | 3.00E-09 | 4.16E-03 | 2.86E-05 | 35.20 |
| Smoking initiation | rs1435741 | A | G | 0.0294151 | 0.425 | 2.64E-16 | 3.59E-03 | 5.45E-05 | 67.10 |
| Smoking initiation | rs1445649 | C | T | 0.0239932 | 0.525 | 1.68E-11 | 3.56E-03 | 3.68E-05 | 45.30 |
| Smoking initiation | rs1518393 | C | A | 0.0205357 | 0.631 | 2.03E-08 | 3.66E-03 | 2.56E-05 | 31.50 |
| Smoking initiation | rs1555445 | T | A | 0.0225548 | 0.337 | 3.65E-09 | 3.82E-03 | 2.82E-05 | 34.80 |
| Smoking initiation | rs1565735 | A | T | -0.037618 | 0.212 | 3.42E-17 | 4.46E-03 | 5.77E-05 | 71.10 |
| Smoking initiation | rs1899896 | T | C | 0.0264481 | 0.286 | 1.04E-11 | 3.89E-03 | 3.76E-05 | 46.30 |
| Smoking initiation | rs1971318 | T | C | 0.0285074 | 0.141 | 7.06E-09 | 4.93E-03 | 2.72E-05 | 33.50 |
| Smoking initiation | rs2046850 | T | C | -0.0248139 | 0.187 | 3.03E-08 | 4.48E-03 | 2.49E-05 | 30.70 |
| Smoking initiation | rs2050586 | C | G | -0.0205467 | 0.355 | 3.00E-08 | 3.71E-03 | 2.49E-05 | 30.70 |
| Smoking initiation | rs2107300 | G | C | -0.027201 | 0.845 | 3.27E-08 | 4.93E-03 | 2.48E-05 | 30.50 |
| Smoking initiation | rs2186122 | T | A | 0.0260573 | 0.561 | 3.61E-13 | 3.59E-03 | 4.29E-05 | 52.80 |
| Smoking initiation | rs222449 | T | A | -0.0253208 | 0.793 | 1.08E-08 | 4.43E-03 | 2.65E-05 | 32.70 |
| Smoking initiation | rs2378662 | A | G | 0.0209482 | 0.556 | 4.16E-09 | 3.57E-03 | 2.80E-05 | 34.50 |
| Smoking initiation | rs240963 | C | T | -0.0410444 | 0.836 | 2.16E-17 | 4.84E-03 | 5.84E-05 | 72.00 |
| Smoking initiation | rs266047 | A | G | -0.0305098 | 0.529 | 3.36E-16 | 3.74E-03 | 5.41E-05 | 66.60 |
| Smoking initiation | rs292071 | C | T | 0.0235822 | 0.244 | 4.08E-09 | 4.01E-03 | 2.81E-05 | 34.60 |
| Smoking initiation | rs3001723 | A | G | 0.0335118 | 0.321 | 8.12E-18 | 3.90E-03 | 6.00E-05 | 73.90 |
| Smoking initiation | rs301805 | G | T | 0.0214679 | 0.559 | 2.80E-09 | 3.61E-03 | 2.86E-05 | 35.30 |
| Smoking initiation | rs35702515 | T | G | 0.0252442 | 0.162 | 2.43E-09 | 4.23E-03 | 2.89E-05 | 35.60 |
| Smoking initiation | rs3904512 | A | G | -0.0211589 | 0.429 | 3.23E-09 | 3.58E-03 | 2.84E-05 | 35.00 |
| Smoking initiation | rs4236259 | G | T | -0.0247689 | 0.499 | 3.35E-12 | 3.56E-03 | 3.94E-05 | 48.50 |
| Smoking initiation | rs4523689 | G | A | -0.0206091 | 0.408 | 1.55E-08 | 3.64E-03 | 2.60E-05 | 32.00 |
| Smoking initiation | rs4543592 | C | T | 0.0219314 | 0.468 | 7.46E-10 | 3.56E-03 | 3.08E-05 | 37.90 |
| Smoking initiation | rs4571506 | T | C | -0.0275747 | 0.492 | 1.09E-14 | 3.57E-03 | 4.85E-05 | 59.70 |
| Smoking initiation | rs4674993 | G | A | -0.0252122 | 0.207 | 1.32E-08 | 4.44E-03 | 2.62E-05 | 32.30 |
| Smoking initiation | rs4759228 | C | G | -0.0216913 | 0.27 | 3.58E-08 | 3.93E-03 | 2.47E-05 | 30.40 |
| Smoking initiation | rs4785836 | C | T | -0.0204704 | 0.398 | 2.26E-08 | 3.66E-03 | 2.54E-05 | 31.30 |
| Smoking initiation | rs56820925 | T | C | -0.0218638 | 0.347 | 1.73E-08 | 3.88E-03 | 2.58E-05 | 31.80 |
| Smoking initiation | rs6265 | T | C | -0.0317863 | 0.203 | 3.77E-12 | 4.58E-03 | 3.91E-05 | 48.20 |
| Smoking initiation | rs6433897 | C | T | 0.0224483 | 0.754 | 3.16E-08 | 4.06E-03 | 2.48E-05 | 30.60 |
| Smoking initiation | rs6508144 | G | C | -0.0206935 | 0.563 | 7.97E-09 | 3.59E-03 | 2.70E-05 | 33.30 |
| Smoking initiation | rs6669839 | T | C | 0.0260041 | 0.204 | 3.36E-09 | 4.40E-03 | 2.84E-05 | 35.00 |
| Smoking initiation | rs6728726 | C | T | 0.0354486 | 0.829 | 6.73E-14 | 4.73E-03 | 4.55E-05 | 56.10 |
| Smoking initiation | rs6788098 | T | A | -0.0313461 | 0.623 | 1.91E-17 | 3.69E-03 | 5.86E-05 | 72.20 |
| Smoking initiation | rs6893752 | G | A | -0.0240995 | 0.766 | 3.25E-09 | 4.07E-03 | 2.84E-05 | 35.00 |
| Smoking initiation | rs7197072 | T | C | -0.0247672 | 0.238 | 2.77E-09 | 4.17E-03 | 2.86E-05 | 35.30 |
| Smoking initiation | rs7224742 | T | C | -0.0207099 | 0.595 | 1.43E-08 | 3.66E-03 | 2.61E-05 | 32.10 |
| Smoking initiation | rs72789632 | T | C | -0.0328856 | 0.12 | 5.02E-10 | 5.29E-03 | 3.14E-05 | 38.70 |
| Smoking initiation | rs72896886 | C | G | -0.0268885 | 0.144 | 2.75E-08 | 4.84E-03 | 2.51E-05 | 30.90 |
| Smoking initiation | rs7322872 | T | C | -0.0255713 | 0.782 | 3.58E-09 | 4.33E-03 | 2.82E-05 | 34.80 |
| Smoking initiation | rs7555507 | T | C | -0.0241444 | 0.496 | 1.14E-11 | 3.56E-03 | 3.74E-05 | 46.10 |
| Smoking initiation | rs7585579 | G | C | 0.0223996 | 0.505 | 1.88E-09 | 3.73E-03 | 2.93E-05 | 36.10 |
| Smoking initiation | rs76214862 | C | A | -0.0249903 | 0.202 | 3.99E-08 | 4.55E-03 | 2.45E-05 | 30.20 |
| Smoking initiation | rs7631735 | T | G | 0.0202273 | 0.603 | 2.79E-08 | 3.64E-03 | 2.50E-05 | 30.80 |
| Smoking initiation | rs76608582 | A | C | -0.0495575 | 0.039 | 1.94E-09 | 8.26E-03 | 2.92E-05 | 36.00 |
| Smoking initiation | rs7921378 | C | G | -0.0254601 | 0.463 | 8.26E-13 | 3.56E-03 | 4.16E-05 | 51.20 |
| Smoking initiation | rs7929518 | G | A | 0.0242377 | 0.765 | 1.56E-08 | 4.28E-03 | 2.60E-05 | 32.00 |
| Smoking initiation | rs7938812 | G | T | 0.0437914 | 0.424 | 2.71E-33 | 3.64E-03 | 1.18E-04 | 145.00 |
| Smoking initiation | rs7969559 | G | A | -0.0243756 | 0.688 | 7.31E-10 | 3.96E-03 | 3.08E-05 | 37.90 |
| Smoking initiation | rs9401770 | A | G | 0.0277307 | 0.273 | 3.47E-12 | 3.99E-03 | 3.93E-05 | 48.40 |
| Smoking initiation | rs9423279 | G | C | -0.0205132 | 0.641 | 3.21E-08 | 3.71E-03 | 2.48E-05 | 30.60 |
| Smoking initiation | rs9540729 | T | A | -0.0195522 | 0.501 | 3.82E-08 | 3.56E-03 | 2.45E-05 | 30.20 |
| Smoking initiation | rs993700 | C | T | -0.025928 | 0.766 | 1.53E-09 | 4.29E-03 | 2.96E-05 | 36.50 |
| Lifetime smoking | rs10052591 | T | C | 0.0083998 | 0.573 | 0.0014022 | 2.10E-09 | 7.76E-05 | 35.89 |
| Lifetime smoking | rs10226228 | A | G | -0.0114099 | 0.63 | 0.001437 | 2.00E-15 | 1.36E-04 | 63.04 |
| Lifetime smoking | rs10282292 | C | T | 0.0089622 | 0.362 | 0.0014469 | 5.90E-10 | 8.29E-05 | 38.37 |
| Lifetime smoking | rs1050847 | C | T | 0.0079705 | 0.426 | 0.001405 | 1.40E-08 | 6.95E-05 | 32.18 |
| Lifetime smoking | rs10823968 | A | T | 0.0081354 | 0.633 | 0.0014526 | 2.10E-08 | 6.78E-05 | 31.37 |
| Lifetime smoking | rs10879871 | T | G | -0.0095799 | 0.343 | 0.001458 | 5.00E-11 | 9.33E-05 | 43.17 |
| Lifetime smoking | rs10918701 | G | A | 0.0080239 | 0.372 | 0.0014319 | 2.10E-08 | 6.79E-05 | 31.40 |
| Lifetime smoking | rs10922907 | A | T | 0.0101748 | 0.451 | 0.001395 | 3.00E-13 | 1.15E-04 | 53.20 |
| Lifetime smoking | rs11210229 | A | G | 0.011702 | 0.384 | 0.0014227 | 2.00E-16 | 1.46E-04 | 67.65 |
| Lifetime smoking | rs112282219 | G | A | -0.0231662 | 0.959 | 0.0035042 | 3.80E-11 | 9.45E-05 | 43.71 |
| Lifetime smoking | rs11255908 | T | G | -0.0100713 | 0.743 | 0.0015881 | 2.30E-10 | 8.69E-05 | 40.22 |
| Lifetime smoking | rs113382419 | C | A | -0.0282339 | 0.889 | 0.0022138 | 3.00E-37 | 3.51E-04 | 162.66 |
| Lifetime smoking | rs11768481 | C | A | 0.0090124 | 0.666 | 0.0014746 | 9.90E-10 | 8.07E-05 | 37.35 |
| Lifetime smoking | rs11783093 | C | T | 0.01571 | 0.839 | 0.0018966 | 1.20E-16 | 1.48E-04 | 68.62 |
| Lifetime smoking | rs11861214 | G | T | 0.0094508 | 0.784 | 0.0016846 | 2.00E-08 | 6.80E-05 | 31.48 |
| Lifetime smoking | rs11948770 | T | C | -0.0102364 | 0.768 | 0.0016452 | 4.90E-10 | 8.37E-05 | 38.71 |
| Lifetime smoking | rs12202536 | A | G | -0.0082339 | 0.513 | 0.001386 | 2.80E-09 | 7.63E-05 | 35.29 |
| Lifetime smoking | rs1221148 | C | G | 0.009165 | 0.587 | 0.0014071 | 7.30E-11 | 9.17E-05 | 42.42 |
| Lifetime smoking | rs12244388 | G | A | -0.0132503 | 0.661 | 0.0014641 | 1.40E-19 | 1.77E-04 | 81.90 |
| Lifetime smoking | rs1246265 | T | C | -0.0088653 | 0.305 | 0.0015091 | 4.20E-09 | 7.46E-05 | 34.51 |
| Lifetime smoking | rs12481282 | G | C | -0.0089436 | 0.722 | 0.0015494 | 7.80E-09 | 7.20E-05 | 33.32 |
| Lifetime smoking | rs12623702 | A | G | -0.0097724 | 0.613 | 0.0014279 | 7.70E-12 | 1.01E-04 | 46.84 |
| Lifetime smoking | rs12708665 | A | G | -0.0090871 | 0.285 | 0.0015389 | 3.50E-09 | 7.54E-05 | 34.87 |
| Lifetime smoking | rs12831617 | C | T | -0.0091846 | 0.764 | 0.0016331 | 1.90E-08 | 6.84E-05 | 31.63 |
| Lifetime smoking | rs12967855 | A | G | 0.0081887 | 0.331 | 0.0014793 | 3.10E-08 | 6.62E-05 | 30.64 |
| Lifetime smoking | rs13009008 | A | G | 0.0086327 | 0.328 | 0.0014733 | 4.60E-09 | 7.42E-05 | 34.33 |
| Lifetime smoking | rs13016665 | C | A | -0.0084875 | 0.577 | 0.0014118 | 1.80E-09 | 7.81E-05 | 36.14 |
| Lifetime smoking | rs13153393 | A | G | -0.0137451 | 0.884 | 0.0021733 | 2.50E-10 | 8.64E-05 | 40.00 |
| Lifetime smoking | rs13296519 | G | T | -0.0097013 | 0.606 | 0.0014189 | 8.10E-12 | 1.01E-04 | 46.75 |
| Lifetime smoking | rs136233 | A | G | -0.0099561 | 0.809 | 0.0017692 | 1.80E-08 | 6.84E-05 | 31.67 |
| Lifetime smoking | rs147412694 | G | A | -0.0115726 | 0.85 | 0.0019487 | 2.90E-09 | 7.62E-05 | 35.27 |
| Lifetime smoking | rs17309874 | G | A | -0.0112899 | 0.74 | 0.0015823 | 9.70E-13 | 1.10E-04 | 50.91 |
| Lifetime smoking | rs17553262 | A | C | -0.0127323 | 0.885 | 0.0021815 | 5.30E-09 | 7.36E-05 | 34.07 |
| Lifetime smoking | rs17576594 | G | A | 0.0109502 | 0.724 | 0.0015516 | 1.70E-12 | 1.08E-04 | 49.81 |
| Lifetime smoking | rs1922018 | C | T | 0.0100328 | 0.364 | 0.0014377 | 3.00E-12 | 1.05E-04 | 48.70 |
| Lifetime smoking | rs1931263 | G | T | -0.0076087 | 0.51 | 0.0013858 | 4.00E-08 | 6.51E-05 | 30.15 |
| Lifetime smoking | rs1933270 | T | G | 0.0092183 | 0.364 | 0.0014381 | 1.50E-10 | 8.88E-05 | 41.09 |
| Lifetime smoking | rs202645 | A | G | -0.0101598 | 0.203 | 0.001725 | 3.90E-09 | 7.50E-05 | 34.69 |
| Lifetime smoking | rs2062882 | G | A | -0.0081103 | 0.587 | 0.00142 | 1.10E-08 | 7.05E-05 | 32.62 |
| Lifetime smoking | rs2254710 | C | A | 0.0089964 | 0.236 | 0.0016309 | 3.50E-08 | 6.58E-05 | 30.43 |
| Lifetime smoking | rs2401924 | G | C | 0.0105718 | 0.502 | 0.0013891 | 2.70E-14 | 1.25E-04 | 57.92 |
| Lifetime smoking | rs245774 | A | G | -0.009016 | 0.272 | 0.0015597 | 7.40E-09 | 7.22E-05 | 33.41 |
| Lifetime smoking | rs2675638 | G | A | 0.0084986 | 0.581 | 0.0014002 | 1.30E-09 | 7.96E-05 | 36.84 |
| Lifetime smoking | rs2678670 | A | T | 0.0087308 | 0.486 | 0.0013876 | 3.10E-10 | 8.56E-05 | 39.59 |
| Lifetime smoking | rs2838834 | C | T | -0.0093649 | 0.699 | 0.0015149 | 6.30E-10 | 8.26E-05 | 38.22 |
| Lifetime smoking | rs28485305 | C | T | 0.0080067 | 0.631 | 0.0014389 | 2.60E-08 | 6.69E-05 | 30.96 |
| Lifetime smoking | rs28635466 | G | A | 0.0084423 | 0.696 | 0.0015048 | 2.00E-08 | 6.80E-05 | 31.47 |
| Lifetime smoking | rs2867112 | T | G | 0.0147832 | 0.835 | 0.0018875 | 4.80E-15 | 1.33E-04 | 61.34 |
| Lifetime smoking | rs2890772 | G | T | -0.0136979 | 0.413 | 0.0014067 | 2.10E-22 | 2.05E-04 | 94.82 |
| Lifetime smoking | rs2894808 | T | A | -0.0152983 | 0.922 | 0.0025912 | 3.50E-09 | 7.53E-05 | 34.86 |
| Lifetime smoking | rs317021 | T | A | -0.011567 | 0.814 | 0.0017909 | 1.10E-10 | 9.02E-05 | 41.72 |
| Lifetime smoking | rs326341 | G | A | 0.0094352 | 0.525 | 0.0013925 | 1.20E-11 | 9.92E-05 | 45.91 |
| Lifetime smoking | rs329120 | C | T | 0.009657 | 0.581 | 0.0014051 | 6.30E-12 | 1.02E-04 | 47.24 |
| Lifetime smoking | rs34866095 | A | G | -0.008572 | 0.686 | 0.0015056 | 1.20E-08 | 7.01E-05 | 32.42 |
| Lifetime smoking | rs348809 | A | G | -0.0082838 | 0.348 | 0.001456 | 1.30E-08 | 7.00E-05 | 32.37 |
| Lifetime smoking | rs35169606 | T | G | 0.0087763 | 0.612 | 0.0014439 | 1.20E-09 | 7.98E-05 | 36.95 |
| Lifetime smoking | rs35175834 | G | A | -0.0164032 | 0.788 | 0.0016985 | 4.60E-22 | 2.02E-04 | 93.27 |
| Lifetime smoking | rs35343344 | C | A | 0.0091814 | 0.733 | 0.0015959 | 8.80E-09 | 7.15E-05 | 33.10 |
| Lifetime smoking | rs359243 | T | C | -0.0087198 | 0.393 | 0.0014254 | 9.50E-10 | 8.09E-05 | 37.42 |
| Lifetime smoking | rs369230 | G | T | -0.0090888 | 0.308 | 0.001511 | 1.80E-09 | 7.82E-05 | 36.18 |
| Lifetime smoking | rs3742365 | T | C | -0.0107929 | 0.595 | 0.0014159 | 2.50E-14 | 1.26E-04 | 58.10 |
| Lifetime smoking | rs3769949 | T | A | -0.0082565 | 0.528 | 0.0013856 | 2.50E-09 | 7.67E-05 | 35.51 |
| Lifetime smoking | rs3811038 | T | C | -0.0095447 | 0.724 | 0.0015575 | 8.90E-10 | 8.12E-05 | 37.56 |
| Lifetime smoking | rs3896224 | A | G | 0.009627 | 0.585 | 0.0014179 | 1.10E-11 | 9.96E-05 | 46.10 |
| Lifetime smoking | rs421983 | T | C | 0.0087074 | 0.519 | 0.0013856 | 3.30E-10 | 8.53E-05 | 39.49 |
| Lifetime smoking | rs4391802 | A | G | 0.0103191 | 0.707 | 0.0015279 | 1.40E-11 | 9.86E-05 | 45.61 |
| Lifetime smoking | rs4473348 | A | T | -0.0104317 | 0.25 | 0.0015968 | 6.40E-11 | 9.22E-05 | 42.68 |
| Lifetime smoking | rs4543592 | T | C | -0.0086624 | 0.52 | 0.0013891 | 4.50E-10 | 8.40E-05 | 38.89 |
| Lifetime smoking | rs4568549 | C | A | -0.0076207 | 0.503 | 0.0013869 | 3.90E-08 | 6.53E-05 | 30.19 |
| Lifetime smoking | rs4571506 | C | T | 0.007877 | 0.54 | 0.0013917 | 1.50E-08 | 6.92E-05 | 32.03 |
| Lifetime smoking | rs4671357 | T | C | -0.0094418 | 0.519 | 0.0013899 | 1.10E-11 | 9.97E-05 | 46.15 |
| Lifetime smoking | rs4731925 | C | T | -0.0082924 | 0.316 | 0.0014902 | 2.60E-08 | 6.69E-05 | 30.96 |
| Lifetime smoking | rs4814873 | C | T | 0.0097121 | 0.767 | 0.0016364 | 2.90E-09 | 7.61E-05 | 35.23 |
| Lifetime smoking | rs4949465 | T | C | -0.0116055 | 0.87 | 0.0020579 | 1.70E-08 | 6.87E-05 | 31.80 |
| Lifetime smoking | rs4957528 | A | C | -0.0101225 | 0.208 | 0.0017221 | 4.20E-09 | 7.47E-05 | 34.55 |
| Lifetime smoking | rs530916 | A | G | -0.0078228 | 0.44 | 0.0013946 | 2.00E-08 | 6.80E-05 | 31.46 |
| Lifetime smoking | rs549845 | G | A | 0.0112613 | 0.301 | 0.0015086 | 8.30E-14 | 1.20E-04 | 55.72 |
| Lifetime smoking | rs57611503 | G | A | 0.0077434 | 0.485 | 0.0014103 | 4.00E-08 | 6.52E-05 | 30.15 |
| Lifetime smoking | rs6011779 | C | T | 0.0191152 | 0.191 | 0.0017636 | 2.30E-27 | 2.54E-04 | 117.47 |
| Lifetime smoking | rs60952428 | T | C | 0.0134109 | 0.909 | 0.0024193 | 3.00E-08 | 6.64E-05 | 30.73 |
| Lifetime smoking | rs6119897 | G | A | -0.0128038 | 0.762 | 0.001627 | 3.60E-15 | 1.34E-04 | 61.93 |
| Lifetime smoking | rs61796681 | A | T | -0.0134163 | 0.912 | 0.0024475 | 4.20E-08 | 6.49E-05 | 30.05 |
| Lifetime smoking | rs62098013 | G | A | -0.0085666 | 0.64 | 0.0014568 | 4.10E-09 | 7.47E-05 | 34.58 |
| Lifetime smoking | rs62135536 | C | T | 0.0243444 | 0.968 | 0.0039617 | 8.00E-10 | 8.16E-05 | 37.76 |
| Lifetime smoking | rs62155874 | A | G | -0.0169073 | 0.873 | 0.0020855 | 5.20E-16 | 1.42E-04 | 65.73 |
| Lifetime smoking | rs62175972 | T | C | 0.0217461 | 0.966 | 0.0038568 | 1.70E-08 | 6.87E-05 | 31.79 |
| Lifetime smoking | rs624833 | T | G | 0.0092857 | 0.695 | 0.0015036 | 6.60E-10 | 8.24E-05 | 38.14 |
| Lifetime smoking | rs6562474 | C | G | 0.0083691 | 0.651 | 0.0014611 | 1.00E-08 | 7.09E-05 | 32.81 |
| Lifetime smoking | rs6598539 | T | C | -0.0081506 | 0.489 | 0.0013894 | 4.50E-09 | 7.44E-05 | 34.41 |
| Lifetime smoking | rs6692614 | C | G | -0.0078157 | 0.421 | 0.0014072 | 2.80E-08 | 6.67E-05 | 30.85 |
| Lifetime smoking | rs6741228 | T | C | 0.0079328 | 0.433 | 0.0014036 | 1.60E-08 | 6.90E-05 | 31.94 |
| Lifetime smoking | rs67596067 | G | A | -0.0088767 | 0.649 | 0.0014584 | 1.20E-09 | 8.01E-05 | 37.04 |
| Lifetime smoking | rs6778080 | T | C | 0.0111141 | 0.267 | 0.0015662 | 1.30E-12 | 1.09E-04 | 50.36 |
| Lifetime smoking | rs6779302 | G | T | -0.0087525 | 0.633 | 0.0014392 | 1.20E-09 | 7.99E-05 | 36.98 |
| Lifetime smoking | rs6935954 | A | G | 0.0095817 | 0.421 | 0.0014019 | 8.20E-12 | 1.01E-04 | 46.71 |
| Lifetime smoking | rs6962772 | A | G | 0.011064 | 0.846 | 0.0019165 | 7.80E-09 | 7.20E-05 | 33.33 |
| Lifetime smoking | rs7039819 | G | A | 0.0087343 | 0.427 | 0.001405 | 5.10E-10 | 8.35E-05 | 38.65 |
| Lifetime smoking | rs7077678 | C | T | 0.0085503 | 0.623 | 0.0014358 | 2.60E-09 | 7.66E-05 | 35.46 |
| Lifetime smoking | rs71367545 | G | A | -0.0103197 | 0.791 | 0.0017038 | 1.40E-09 | 7.93E-05 | 36.69 |
| Lifetime smoking | rs7155595 | A | C | -0.0088516 | 0.674 | 0.0014851 | 2.50E-09 | 7.68E-05 | 35.53 |
| Lifetime smoking | rs71627581 | G | A | 0.0132566 | 0.889 | 0.0021987 | 1.60E-09 | 7.86E-05 | 36.35 |
| Lifetime smoking | rs72674867 | A | T | 0.0089877 | 0.765 | 0.001634 | 3.80E-08 | 6.54E-05 | 30.26 |
| Lifetime smoking | rs72678864 | G | A | 0.0123829 | 0.829 | 0.0018387 | 1.60E-11 | 9.80E-05 | 45.35 |
| Lifetime smoking | rs7297175 | T | C | -0.0081165 | 0.431 | 0.0013992 | 6.60E-09 | 7.27E-05 | 33.65 |
| Lifetime smoking | rs732083 | G | A | 0.0083482 | 0.333 | 0.0014731 | 1.50E-08 | 6.94E-05 | 32.12 |
| Lifetime smoking | rs73220544 | A | C | -0.0108189 | 0.842 | 0.0019126 | 1.50E-08 | 6.92E-05 | 32.00 |
| Lifetime smoking | rs7333559 | G | A | 0.01074 | 0.212 | 0.0017072 | 3.20E-10 | 8.55E-05 | 39.58 |
| Lifetime smoking | rs74086911 | G | A | 0.0148021 | 0.925 | 0.002642 | 2.10E-08 | 6.78E-05 | 31.39 |
| Lifetime smoking | rs7519626 | C | T | 0.0084202 | 0.324 | 0.0014789 | 1.20E-08 | 7.01E-05 | 32.41 |
| Lifetime smoking | rs7528604 | G | A | 0.0096527 | 0.566 | 0.0014014 | 5.70E-12 | 1.03E-04 | 47.44 |
| Lifetime smoking | rs7553348 | G | A | 0.009634 | 0.438 | 0.0013964 | 5.20E-12 | 1.03E-04 | 47.60 |
| Lifetime smoking | rs7569203 | A | C | -0.0107595 | 0.689 | 0.0015002 | 7.40E-13 | 1.11E-04 | 51.44 |
| Lifetime smoking | rs75742406 | G | A | 0.0096195 | 0.739 | 0.0015849 | 1.30E-09 | 7.96E-05 | 36.84 |
| Lifetime smoking | rs76608582 | C | A | 0.0216317 | 0.953 | 0.00344 | 3.20E-10 | 8.55E-05 | 39.54 |
| Lifetime smoking | rs7766610 | C | A | 0.0125839 | 0.183 | 0.0017928 | 2.20E-12 | 1.06E-04 | 49.27 |
| Lifetime smoking | rs7807019 | A | G | -0.0104211 | 0.54 | 0.0013906 | 6.70E-14 | 1.21E-04 | 56.16 |
| Lifetime smoking | rs8042134 | T | G | -0.0099414 | 0.541 | 0.0014011 | 1.30E-12 | 1.09E-04 | 50.34 |
| Lifetime smoking | rs8042849 | C | T | 0.0192164 | 0.342 | 0.0014618 | 1.80E-39 | 3.73E-04 | 172.80 |
| Lifetime smoking | rs812887 | A | G | 0.008248 | 0.414 | 0.0014102 | 5.00E-09 | 7.39E-05 | 34.21 |
| Lifetime smoking | rs860326 | C | T | 0.0083381 | 0.428 | 0.0014023 | 2.70E-09 | 7.64E-05 | 35.35 |
| Lifetime smoking | rs8614 | C | A | -0.0114612 | 0.817 | 0.0017968 | 1.80E-10 | 8.79E-05 | 40.69 |
| Lifetime smoking | rs9435340 | T | A | 0.0083477 | 0.344 | 0.0014642 | 1.20E-08 | 7.02E-05 | 32.50 |
| Lifetime smoking | rs9842947 | C | T | -0.0087719 | 0.326 | 0.0014807 | 3.10E-09 | 7.58E-05 | 35.09 |
| Lifetime smoking | rs986391 | G | A | 0.0111388 | 0.367 | 0.0014379 | 9.40E-15 | 1.30E-04 | 60.01 |
| Lifetime smoking | rs9919670 | G | A | -0.0152438 | 0.612 | 0.0014211 | 7.60E-27 | 2.49E-04 | 115.06 |
| Alcohol drinking | rs10085696 | G | A | -0.0160517 | 0.201 | 1.24E-10 | 2.49E-03 | 4.40E-05 | 41.40 |
| Alcohol drinking | rs10753661 | A | G | -0.0113656 | 0.702 | 4.24E-08 | 2.08E-03 | 3.19E-05 | 30.00 |
| Alcohol drinking | rs1123285 | G | C | -0.0124183 | 0.339 | 1.36E-09 | 2.05E-03 | 3.90E-05 | 36.70 |
| Alcohol drinking | rs11860773 | C | T | -0.0150051 | 0.176 | 8.35E-10 | 2.44E-03 | 4.01E-05 | 37.70 |
| Alcohol drinking | rs1229984 | C | T | 0.1881154 | 0.953 | 1.00E-200 | 6.18E-03 | 9.84E-04 | 927.00 |
| Alcohol drinking | rs1260326 | C | T | 0.023812 | 0.595 | 3.33E-33 | 1.98E-03 | 1.53E-04 | 144.00 |
| Alcohol drinking | rs13107325 | T | C | -0.0364548 | 0.065 | 1.23E-20 | 3.91E-03 | 9.22E-05 | 86.80 |
| Alcohol drinking | rs13332432 | G | C | 0.014005 | 0.296 | 5.94E-11 | 2.14E-03 | 4.55E-05 | 42.80 |
| Alcohol drinking | rs1387766 | A | G | -0.010827 | 0.622 | 4.79E-08 | 1.98E-03 | 3.17E-05 | 29.80 |
| Alcohol drinking | rs153106 | C | T | -0.0136118 | 0.409 | 3.63E-12 | 1.96E-03 | 5.13E-05 | 48.30 |
| Alcohol drinking | rs16854020 | A | G | 0.018083 | 0.127 | 4.82E-10 | 2.91E-03 | 4.11E-05 | 38.70 |
| Alcohol drinking | rs17542254 | G | A | 0.0131418 | 0.251 | 8.96E-10 | 2.15E-03 | 3.98E-05 | 37.50 |
| Alcohol drinking | rs2049045 | C | G | -0.0137683 | 0.189 | 3.97E-08 | 2.51E-03 | 3.21E-05 | 30.20 |
| Alcohol drinking | rs2299409 | A | G | -0.0105546 | 0.493 | 4.80E-08 | 1.93E-03 | 3.17E-05 | 29.80 |
| Alcohol drinking | rs28601761 | G | C | 0.0112989 | 0.405 | 7.60E-09 | 1.96E-03 | 3.55E-05 | 33.40 |
| Alcohol drinking | rs28680958 | A | G | -0.0135848 | 0.23 | 9.78E-09 | 2.37E-03 | 3.50E-05 | 32.90 |
| Alcohol drinking | rs28712821 | A | G | 0.0283339 | 0.594 | 1.10E-46 | 1.97E-03 | 2.19E-04 | 206.00 |
| Alcohol drinking | rs28732378 | G | A | -0.0167274 | 0.729 | 2.24E-14 | 2.19E-03 | 6.19E-05 | 58.30 |
| Alcohol drinking | rs28929474 | T | C | -0.0476809 | 0.015 | 2.39E-11 | 7.14E-03 | 4.74E-05 | 44.60 |
| Alcohol drinking | rs331939 | A | G | -0.0119016 | 0.339 | 4.50E-09 | 2.03E-03 | 3.65E-05 | 34.40 |
| Alcohol drinking | rs34121753 | G | A | 0.0110694 | 0.532 | 1.39E-08 | 1.95E-03 | 3.42E-05 | 32.20 |
| Alcohol drinking | rs34704785 | T | C | -0.0105815 | 0.412 | 4.52E-08 | 1.94E-03 | 3.18E-05 | 29.90 |
| Alcohol drinking | rs4233567 | T | C | -0.0125899 | 0.34 | 3.83E-10 | 2.01E-03 | 4.16E-05 | 39.20 |
| Alcohol drinking | rs4309187 | C | A | 0.0147908 | 0.697 | 1.37E-12 | 2.09E-03 | 5.33E-05 | 50.20 |
| Alcohol drinking | rs4752999 | T | C | -0.0145643 | 0.321 | 2.03E-12 | 2.07E-03 | 5.26E-05 | 49.50 |
| Alcohol drinking | rs4916723 | C | A | -0.0112709 | 0.404 | 8.07E-09 | 1.95E-03 | 3.54E-05 | 33.30 |
| Alcohol drinking | rs528301 | A | G | 0.0155919 | 0.605 | 1.25E-15 | 1.95E-03 | 6.80E-05 | 64.00 |
| Alcohol drinking | rs55872084 | T | G | 0.0127314 | 0.218 | 1.98E-08 | 2.27E-03 | 3.35E-05 | 31.50 |
| Alcohol drinking | rs55932213 | G | A | 0.0124767 | 0.701 | 1.80E-08 | 2.22E-03 | 3.37E-05 | 31.70 |
| Alcohol drinking | rs6106989 | A | G | 0.0108994 | 0.628 | 3.81E-08 | 1.98E-03 | 3.21E-05 | 30.20 |
| Alcohol drinking | rs62135521 | T | G | -0.0263576 | 0.038 | 9.91E-09 | 4.60E-03 | 3.50E-05 | 32.90 |
| Alcohol drinking | rs6739804 | C | T | -0.0129688 | 0.66 | 4.72E-10 | 2.08E-03 | 4.12E-05 | 38.80 |
| Alcohol drinking | rs76640332 | A | G | -0.0210081 | 0.204 | 1.47E-18 | 2.39E-03 | 8.21E-05 | 77.30 |
| Alcohol drinking | rs78234152 | A | G | 0.0276538 | 0.099 | 2.18E-19 | 3.07E-03 | 8.62E-05 | 81.10 |
| Alcohol drinking | rs79616692 | C | G | 0.0188085 | 0.11 | 2.38E-09 | 3.15E-03 | 3.78E-05 | 35.60 |
| Alcohol drinking | rs838145 | A | G | -0.0157978 | 0.584 | 3.87E-16 | 1.94E-03 | 7.04E-05 | 66.30 |
| Coffee consumption | rs10127720 | T | C | -0.0118704 | 0.26 | 0.0017794 | 2.54E-11 | 1.18E-04 | 44.50 |
| Coffee consumption | rs1057868 | C | T | -0.0189046 | 0.715 | 0.00172 | 4.27E-28 | 3.21E-04 | 120.80 |
| Coffee consumption | rs10865548 | A | G | -0.0160802 | 0.172 | 0.0020613 | 6.15E-15 | 1.62E-04 | 60.86 |
| Coffee consumption | rs10997940 | C | T | 0.0088791 | 0.596 | 0.0015882 | 2.27E-08 | 8.32E-05 | 31.25 |
| Coffee consumption | rs117810762 | G | A | -0.0344484 | 0.982 | 0.0059387 | 6.61E-09 | 8.95E-05 | 33.65 |
| Coffee consumption | rs1260326 | T | C | -0.0131589 | 0.393 | 0.0015909 | 1.33E-16 | 1.82E-04 | 68.41 |
| Coffee consumption | rs16903275 | C | A | -0.0156172 | 0.844 | 0.0021409 | 3.00E-13 | 1.42E-04 | 53.21 |
| Coffee consumption | rs17687539 | A | G | 0.0104566 | 0.791 | 0.0019167 | 4.89E-08 | 7.92E-05 | 29.76 |
| Coffee consumption | rs2231142 | G | T | 0.0153129 | 0.886 | 0.0024484 | 4.00E-10 | 1.04E-04 | 39.12 |
| Coffee consumption | rs2297508 | C | G | -0.0099609 | 0.349 | 0.0016369 | 1.17E-09 | 9.85E-05 | 37.03 |
| Coffee consumption | rs2330783 | G | T | 0.0437258 | 0.986 | 0.0066392 | 4.53E-11 | 1.15E-04 | 43.37 |
| Coffee consumption | rs2465037 | C | A | 0.0112132 | 0.657 | 0.0016448 | 9.27E-12 | 1.24E-04 | 46.48 |
| Coffee consumption | rs2472297 | C | T | -0.0446599 | 0.733 | 0.0017551 | 1.09E-142 | 1.72E-03 | 647.46 |
| Coffee consumption | rs2521501 | A | T | 0.0097212 | 0.677 | 0.0016764 | 6.69E-09 | 8.95E-05 | 33.63 |
| Coffee consumption | rs2613458 | A | C | -0.0101888 | 0.279 | 0.0017481 | 5.59E-09 | 9.04E-05 | 33.97 |
| Coffee consumption | rs34060476 | A | G | -0.0180481 | 0.866 | 0.0022836 | 2.72E-15 | 1.66E-04 | 62.46 |
| Coffee consumption | rs4410790 | T | C | -0.0380043 | 0.366 | 0.0016147 | 2.15E-122 | 1.47E-03 | 553.98 |
| Coffee consumption | rs56113850 | T | C | -0.0122705 | 0.423 | 0.0015735 | 6.30E-15 | 1.62E-04 | 60.81 |
| Coffee consumption | rs574367 | G | T | -0.0108154 | 0.788 | 0.0019019 | 1.30E-08 | 8.60E-05 | 32.34 |
| Coffee consumption | rs57918684 | G | A | -0.0125708 | 0.845 | 0.0021544 | 5.38E-09 | 9.06E-05 | 34.05 |
| Coffee consumption | rs597045 | A | T | 0.0101936 | 0.695 | 0.0017098 | 2.50E-09 | 9.46E-05 | 35.54 |
| Coffee consumption | rs6062357 | C | T | -0.0102789 | 0.528 | 0.001585 | 8.89E-11 | 1.12E-04 | 42.05 |
| Coffee consumption | rs660550 | C | A | 0.0093243 | 0.474 | 0.0015572 | 2.13E-09 | 9.54E-05 | 35.85 |
| Coffee consumption | rs66723169 | C | A | -0.0152476 | 0.769 | 0.00185 | 1.70E-16 | 1.81E-04 | 67.93 |
| Coffee consumption | rs73075167 | A | T | 0.0155832 | 0.87 | 0.0023531 | 3.54E-11 | 1.17E-04 | 43.86 |
| Coffee consumption | rs75347775 | G | A | -0.0104695 | 0.755 | 0.001809 | 7.15E-09 | 8.91E-05 | 33.49 |
| Coffee consumption | rs993885 | G | A | 0.0102821 | 0.633 | 0.0016197 | 2.18E-10 | 1.07E-04 | 40.30 |
| Vigorous physical activity | rs1248860 | G | A | -0.0097697 | 0.484 | 0.0013143 | 1.10E-13 | 2.12E-04 | 55.26 |
| Vigorous physical activity | rs13243553 | G | A | 0.0087485 | 0.608 | 0.0013496 | 9.00E-11 | 1.61E-04 | 42.02 |
| Vigorous physical activity | rs2764261 | A | G | 0.0091263 | 0.374 | 0.0013605 | 2.00E-11 | 1.72E-04 | 45.00 |
| Vigorous physical activity | rs328902 | C | T | -0.0087886 | 0.685 | 0.0014168 | 5.50E-10 | 1.47E-04 | 38.48 |
| Vigorous physical activity | rs6667222 | A | C | 0.0087305 | 0.748 | 0.0015174 | 8.70E-09 | 1.27E-04 | 33.10 |
| Vigorous physical activity | rs9276758 | G | A | 0.0080319 | 0.688 | 0.0014171 | 1.40E-08 | 1.23E-04 | 32.13 |
| Body mass index | rs10002111 | A | G | 0.0125 | 0.223 | 0.0021 | 1.26E-09 | 5.10E-05 | 35.43 |
| Body mass index | rs10033843 | A | G | 0.014 | 0.215 | 0.0021 | 1.07E-11 | 6.40E-05 | 44.44 |
| Body mass index | rs10050620 | T | C | -0.013 | 0.331 | 0.002 | 1.20E-10 | 6.08E-05 | 42.25 |
| Body mass index | rs1006317 | T | G | 0.016 | 0.127 | 0.0026 | 3.75E-10 | 5.45E-05 | 37.87 |
| Body mass index | rs10099330 | A | G | -0.0119 | 0.538 | 0.0017 | 3.22E-12 | 7.05E-05 | 49.00 |
| Body mass index | rs10101364 | T | C | 0.012 | 0.68 | 0.0018 | 5.61E-11 | 6.40E-05 | 44.44 |
| Body mass index | rs10110189 | T | C | -0.0156 | 0.106 | 0.0028 | 2.20E-08 | 4.47E-05 | 31.04 |
| Body mass index | rs10121187 | C | G | 0.0102 | 0.509 | 0.0017 | 2.02E-09 | 5.18E-05 | 36.00 |
| Body mass index | rs10132280 | A | C | -0.0214 | 0.311 | 0.0018 | 2.28E-33 | 2.03E-04 | 141.35 |
| Body mass index | rs10145749 | T | C | 0.0161 | 0.155 | 0.0026 | 8.33E-10 | 5.52E-05 | 38.34 |
| Body mass index | rs10168563 | A | G | 0.0126 | 0.703 | 0.0018 | 9.33E-12 | 7.05E-05 | 49.00 |
| Body mass index | rs10169594 | T | C | -0.012 | 0.648 | 0.0018 | 1.34E-11 | 6.40E-05 | 44.44 |
| Body mass index | rs10182181 | A | G | -0.0327 | 0.505 | 0.0016 | 2.45E-91 | 6.01E-04 | 417.69 |
| Body mass index | rs10197031 | T | C | -0.0161 | 0.726 | 0.0019 | 5.05E-18 | 1.03E-04 | 71.80 |
| Body mass index | rs10261050 | T | C | 0.0113 | 0.475 | 0.0017 | 4.53E-11 | 6.36E-05 | 44.18 |
| Body mass index | rs1048637 | T | G | -0.0094 | 0.547 | 0.0017 | 2.94E-08 | 4.40E-05 | 30.57 |
| Body mass index | rs10499694 | A | G | 0.013 | 0.494 | 0.0016 | 1.29E-15 | 9.50E-05 | 66.02 |
| Body mass index | rs10506971 | A | G | -0.0142 | 0.552 | 0.0017 | 4.69E-17 | 1.00E-04 | 69.77 |
| Body mass index | rs10510419 | T | G | -0.0168 | 0.147 | 0.0023 | 2.23E-13 | 7.68E-05 | 53.35 |
| Body mass index | rs10518694 | A | C | 0.0144 | 0.139 | 0.0024 | 3.43E-09 | 5.18E-05 | 36.00 |
| Body mass index | rs10733051 | A | G | 0.0093 | 0.512 | 0.0016 | 6.96E-09 | 4.86E-05 | 33.79 |
| Body mass index | rs10733682 | A | G | 0.0148 | 0.459 | 0.0016 | 1.76E-19 | 1.23E-04 | 85.56 |
| Body mass index | rs10741329 | A | G | 0.0115 | 0.694 | 0.0018 | 3.91E-10 | 5.88E-05 | 40.82 |
| Body mass index | rs10742752 | T | C | -0.0123 | 0.376 | 0.0017 | 1.20E-13 | 7.54E-05 | 52.35 |
| Body mass index | rs1075901 | T | C | -0.0118 | 0.452 | 0.0016 | 4.43E-13 | 7.83E-05 | 54.39 |
| Body mass index | rs10761785 | T | G | -0.0133 | 0.514 | 0.0016 | 3.47E-16 | 9.95E-05 | 69.10 |
| Body mass index | rs10772983 | T | C | -0.01 | 0.539 | 0.0016 | 6.21E-10 | 5.62E-05 | 39.06 |
| Body mass index | rs10779751 | A | G | 0.0131 | 0.279 | 0.0018 | 2.66E-13 | 7.62E-05 | 52.97 |
| Body mass index | rs10788494 | C | G | 0.0132 | 0.484 | 0.0017 | 5.51E-15 | 8.68E-05 | 60.29 |
| Body mass index | rs10811868 | A | G | -0.01 | 0.32 | 0.0018 | 3.18E-08 | 4.44E-05 | 30.86 |
| Body mass index | rs10829164 | T | C | 0.0151 | 0.147 | 0.0024 | 2.37E-10 | 5.70E-05 | 39.58 |
| Body mass index | rs10842240 | C | G | 0.0195 | 0.126 | 0.0025 | 1.21E-14 | 8.76E-05 | 60.84 |
| Body mass index | rs10858334 | C | G | -0.0148 | 0.848 | 0.0025 | 4.91E-09 | 5.04E-05 | 35.05 |
| Body mass index | rs10864728 | A | G | 0.011 | 0.398 | 0.0019 | 1.20E-08 | 4.82E-05 | 33.52 |
| Body mass index | rs10909880 | T | C | -0.0135 | 0.452 | 0.0016 | 2.21E-16 | 1.02E-04 | 71.19 |
| Body mass index | rs10920336 | A | G | -0.0101 | 0.533 | 0.0017 | 4.20E-09 | 5.08E-05 | 35.30 |
| Body mass index | rs10920678 | A | G | 0.0149 | 0.425 | 0.0016 | 7.15E-20 | 1.25E-04 | 86.72 |
| Body mass index | rs10929925 | A | C | -0.0142 | 0.411 | 0.0016 | 3.05E-18 | 1.13E-04 | 78.77 |
| Body mass index | rs10938397 | A | G | -0.0322 | 0.57 | 0.0016 | 2.42E-86 | 5.83E-04 | 405.01 |
| Body mass index | rs10942267 | A | G | 0.0148 | 0.693 | 0.0018 | 6.53E-16 | 9.73E-05 | 67.60 |
| Body mass index | rs10961649 | T | C | 0.0104 | 0.324 | 0.0018 | 1.51E-08 | 4.81E-05 | 33.38 |
| Body mass index | rs10962550 | C | G | 0.0186 | 0.176 | 0.0022 | 5.68E-17 | 1.03E-04 | 71.48 |
| Body mass index | rs10968114 | A | C | 0.0113 | 0.533 | 0.0017 | 3.30E-11 | 6.36E-05 | 44.18 |
| Body mass index | rs10975933 | C | G | 0.0114 | 0.663 | 0.0018 | 2.07E-10 | 5.77E-05 | 40.11 |
| Body mass index | rs10992867 | A | G | 0.0162 | 0.269 | 0.0019 | 3.20E-17 | 1.05E-04 | 72.70 |
| Body mass index | rs11001259 | A | T | -0.0138 | 0.177 | 0.0024 | 1.22E-08 | 4.76E-05 | 33.06 |
| Body mass index | rs11030618 | T | C | 0.011 | 0.579 | 0.0017 | 1.67E-10 | 6.03E-05 | 41.87 |
| Body mass index | rs11044430 | A | T | 0.0161 | 0.844 | 0.0023 | 3.77E-12 | 7.05E-05 | 49.00 |
| Body mass index | rs11046972 | T | C | 0.0176 | 0.071 | 0.0032 | 3.72E-08 | 4.35E-05 | 30.25 |
| Body mass index | rs11060853 | A | G | -0.0107 | 0.591 | 0.0019 | 3.10E-08 | 4.57E-05 | 31.71 |
| Body mass index | rs11066188 | A | G | -0.0114 | 0.376 | 0.0016 | 3.05E-12 | 7.31E-05 | 50.77 |
| Body mass index | rs11105839 | A | T | -0.0113 | 0.373 | 0.0017 | 1.25E-11 | 6.36E-05 | 44.18 |
| Body mass index | rs11115176 | T | C | 0.0131 | 0.784 | 0.0019 | 6.79E-12 | 6.84E-05 | 47.54 |
| Body mass index | rs11121210 | T | C | -0.0111 | 0.345 | 0.0017 | 2.37E-10 | 6.14E-05 | 42.63 |
| Body mass index | rs11128021 | A | G | -0.0181 | 0.162 | 0.0024 | 1.63E-14 | 8.19E-05 | 56.88 |
| Body mass index | rs11150911 | A | C | 0.0118 | 0.285 | 0.0018 | 3.72E-11 | 6.19E-05 | 42.98 |
| Body mass index | rs11165643 | T | C | 0.0185 | 0.58 | 0.0016 | 4.49E-30 | 1.92E-04 | 133.69 |
| Body mass index | rs11170468 | A | C | 0.013 | 0.783 | 0.0019 | 1.12E-11 | 6.74E-05 | 46.81 |
| Body mass index | rs11218510 | A | G | -0.014 | 0.395 | 0.002 | 6.79E-13 | 7.05E-05 | 49.00 |
| Body mass index | rs11246136 | A | C | -0.0167 | 0.102 | 0.003 | 2.03E-08 | 4.46E-05 | 30.99 |
| Body mass index | rs112646560 | T | C | 0.018 | 0.212 | 0.0023 | 1.29E-14 | 8.82E-05 | 61.25 |
| Body mass index | rs1126930 | C | G | 0.0341 | 0.034 | 0.0053 | 1.00E-10 | 5.96E-05 | 41.40 |
| Body mass index | rs11525873 | T | C | 0.0232 | 0.9 | 0.0032 | 2.98E-13 | 7.57E-05 | 52.56 |
| Body mass index | rs11538 | A | G | -0.0138 | 0.828 | 0.0023 | 1.09E-09 | 5.18E-05 | 36.00 |
| Body mass index | rs11577094 | T | C | 0.0186 | 0.079 | 0.003 | 3.28E-10 | 5.53E-05 | 38.44 |
| Body mass index | rs11594179 | T | C | -0.0109 | 0.233 | 0.0019 | 2.16E-08 | 4.74E-05 | 32.91 |
| Body mass index | rs1159692 | A | C | 0.0135 | 0.475 | 0.0017 | 5.54E-15 | 9.08E-05 | 63.06 |
| Body mass index | rs11611246 | T | G | 0.0223 | 0.199 | 0.002 | 2.04E-28 | 1.79E-04 | 124.32 |
| Body mass index | rs11614340 | T | C | -0.0117 | 0.707 | 0.0018 | 1.87E-10 | 6.08E-05 | 42.25 |
| Body mass index | rs11615578 | T | C | 0.0117 | 0.258 | 0.002 | 3.04E-09 | 4.93E-05 | 34.22 |
| Body mass index | rs11633626 | A | C | -0.0157 | 0.631 | 0.0018 | 7.27E-19 | 1.10E-04 | 76.08 |
| Body mass index | rs11636611 | T | C | 0.0104 | 0.498 | 0.0017 | 8.85E-10 | 5.39E-05 | 37.43 |
| Body mass index | rs116374395 | A | G | 0.0321 | 0.035 | 0.0052 | 7.05E-10 | 5.49E-05 | 38.11 |
| Body mass index | rs11649864 | A | G | 0.0192 | 0.086 | 0.003 | 2.52E-10 | 5.90E-05 | 40.96 |
| Body mass index | rs11655587 | T | C | -0.021 | 0.357 | 0.002 | 6.87E-26 | 1.59E-04 | 110.25 |
| Body mass index | rs11672660 | T | C | -0.0338 | 0.187 | 0.0021 | 6.83E-60 | 3.73E-04 | 259.06 |
| Body mass index | rs11692326 | T | C | 0.0147 | 0.23 | 0.0019 | 1.69E-14 | 8.62E-05 | 59.86 |
| Body mass index | rs11695013 | T | C | -0.0104 | 0.632 | 0.0017 | 2.43E-09 | 5.39E-05 | 37.43 |
| Body mass index | rs11713193 | A | G | 0.0246 | 0.516 | 0.0017 | 3.02E-48 | 3.01E-04 | 209.40 |
| Body mass index | rs11739877 | T | C | 0.0116 | 0.623 | 0.0018 | 4.01E-11 | 5.98E-05 | 41.53 |
| Body mass index | rs11757278 | T | C | 0.0133 | 0.696 | 0.0019 | 6.92E-13 | 7.05E-05 | 49.00 |
| Body mass index | rs11772246 | T | C | 0.0145 | 0.823 | 0.0022 | 3.69E-11 | 6.25E-05 | 43.44 |
| Body mass index | rs11773362 | T | C | -0.0105 | 0.34 | 0.0018 | 6.39E-09 | 4.90E-05 | 34.03 |
| Body mass index | rs11782074 | T | G | 0.0124 | 0.368 | 0.0018 | 4.24E-12 | 6.83E-05 | 47.46 |
| Body mass index | rs118081010 | T | C | 0.0518 | 0.017 | 0.0075 | 5.28E-12 | 6.87E-05 | 47.70 |
| Body mass index | rs11882409 | A | C | 0.0121 | 0.303 | 0.0019 | 3.31E-10 | 5.84E-05 | 40.56 |
| Body mass index | rs11902450 | T | C | 0.0167 | 0.105 | 0.0027 | 1.06E-09 | 5.51E-05 | 38.26 |
| Body mass index | rs11915371 | A | C | -0.0154 | 0.802 | 0.0021 | 2.29E-13 | 7.74E-05 | 53.78 |
| Body mass index | rs11919665 | A | T | 0.0117 | 0.325 | 0.002 | 7.30E-09 | 4.93E-05 | 34.22 |
| Body mass index | rs11921432 | T | C | -0.0189 | 0.891 | 0.0027 | 5.38E-12 | 7.05E-05 | 49.00 |
| Body mass index | rs12072739 | A | G | -0.0169 | 0.776 | 0.0023 | 1.50E-13 | 7.77E-05 | 53.99 |
| Body mass index | rs12098284 | T | C | 0.0184 | 0.121 | 0.0026 | 9.87E-13 | 7.21E-05 | 50.08 |
| Body mass index | rs12140153 | T | G | -0.0353 | 0.091 | 0.0034 | 1.44E-25 | 1.55E-04 | 107.79 |
| Body mass index | rs12150665 | T | C | 0.0168 | 0.593 | 0.0016 | 1.74E-24 | 1.59E-04 | 110.25 |
| Body mass index | rs12259464 | A | G | 0.0109 | 0.481 | 0.0017 | 1.48E-10 | 5.92E-05 | 41.11 |
| Body mass index | rs12282785 | A | C | -0.0157 | 0.22 | 0.0023 | 1.43E-11 | 6.71E-05 | 46.60 |
| Body mass index | rs12286929 | A | G | -0.0177 | 0.501 | 0.0016 | 1.93E-27 | 1.76E-04 | 122.38 |
| Body mass index | rs12334877 | A | G | -0.0144 | 0.191 | 0.0021 | 2.17E-11 | 6.77E-05 | 47.02 |
| Body mass index | rs12364470 | T | G | -0.0187 | 0.854 | 0.0022 | 2.18E-17 | 1.04E-04 | 72.25 |
| Body mass index | rs12369179 | T | C | -0.034 | 0.085 | 0.0031 | 2.32E-28 | 1.73E-04 | 120.29 |
| Body mass index | rs12386885 | T | C | 0.0151 | 0.156 | 0.0026 | 9.90E-09 | 4.86E-05 | 33.73 |
| Body mass index | rs12421848 | A | G | -0.0141 | 0.396 | 0.0019 | 3.99E-13 | 7.93E-05 | 55.07 |
| Body mass index | rs12429545 | A | G | 0.0313 | 0.122 | 0.0024 | 1.42E-37 | 2.45E-04 | 170.08 |
| Body mass index | rs12439632 | C | G | 0.0145 | 0.168 | 0.0023 | 3.68E-10 | 5.72E-05 | 39.74 |
| Body mass index | rs12448257 | A | G | 0.0161 | 0.219 | 0.002 | 9.04E-16 | 9.33E-05 | 64.80 |
| Body mass index | rs12462975 | A | G | 0.0193 | 0.324 | 0.0018 | 1.47E-25 | 1.65E-04 | 114.97 |
| Body mass index | rs12527426 | A | G | 0.015 | 0.3 | 0.0019 | 5.54E-16 | 8.97E-05 | 62.33 |
| Body mass index | rs12591120 | T | C | 0.012 | 0.74 | 0.0022 | 3.84E-08 | 4.28E-05 | 29.75 |
| Body mass index | rs12594043 | C | G | 0.0102 | 0.521 | 0.0017 | 1.31E-09 | 5.18E-05 | 36.00 |
| Body mass index | rs12602912 | T | C | 0.0166 | 0.212 | 0.002 | 2.90E-16 | 9.92E-05 | 68.89 |
| Body mass index | rs12611148 | A | C | -0.0137 | 0.143 | 0.0024 | 1.42E-08 | 4.69E-05 | 32.58 |
| Body mass index | rs12628051 | T | C | 0.0161 | 0.641 | 0.0018 | 2.90E-19 | 1.15E-04 | 80.00 |
| Body mass index | rs12628891 | T | C | -0.0115 | 0.319 | 0.0019 | 5.85E-10 | 5.27E-05 | 36.63 |
| Body mass index | rs12635553 | A | T | 0.0099 | 0.486 | 0.0017 | 4.10E-09 | 4.88E-05 | 33.91 |
| Body mass index | rs12636480 | T | G | 0.0128 | 0.351 | 0.0018 | 6.96E-13 | 7.28E-05 | 50.57 |
| Body mass index | rs12642970 | C | G | -0.0109 | 0.44 | 0.0019 | 1.71E-08 | 4.74E-05 | 32.91 |
| Body mass index | rs12652212 | A | G | -0.0131 | 0.561 | 0.0016 | 1.63E-15 | 9.65E-05 | 67.03 |
| Body mass index | rs1268065 | A | G | -0.01 | 0.5 | 0.0016 | 7.12E-10 | 5.62E-05 | 39.06 |
| Body mass index | rs12680842 | A | G | 0.0142 | 0.684 | 0.0017 | 3.41E-16 | 1.00E-04 | 69.77 |
| Body mass index | rs12681792 | A | C | 0.015 | 0.202 | 0.0021 | 2.88E-12 | 7.34E-05 | 51.02 |
| Body mass index | rs12692596 | T | C | 0.012 | 0.362 | 0.0017 | 1.03E-12 | 7.17E-05 | 49.83 |
| Body mass index | rs12714199 | T | C | -0.0141 | 0.615 | 0.0017 | 3.22E-16 | 9.90E-05 | 68.79 |
| Body mass index | rs12765914 | T | C | 0.0226 | 0.08 | 0.0031 | 1.96E-13 | 7.65E-05 | 53.15 |
| Body mass index | rs12868881 | A | T | 0.0138 | 0.407 | 0.0017 | 1.40E-15 | 9.49E-05 | 65.90 |
| Body mass index | rs12888545 | A | G | -0.0133 | 0.746 | 0.002 | 1.76E-11 | 6.37E-05 | 44.22 |
| Body mass index | rs12912198 | T | C | -0.01 | 0.273 | 0.0018 | 3.44E-08 | 4.44E-05 | 30.86 |
| Body mass index | rs12914623 | C | G | -0.0159 | 0.272 | 0.0019 | 2.00E-16 | 1.01E-04 | 70.03 |
| Body mass index | rs12922346 | C | G | 0.0133 | 0.264 | 0.002 | 1.48E-11 | 6.37E-05 | 44.22 |
| Body mass index | rs12926250 | T | G | 0.018 | 0.105 | 0.0028 | 1.78E-10 | 5.95E-05 | 41.33 |
| Body mass index | rs1293037 | T | C | 0.0129 | 0.754 | 0.0022 | 5.73E-09 | 4.95E-05 | 34.38 |
| Body mass index | rs12939549 | A | G | 0.018 | 0.557 | 0.0016 | 3.68E-28 | 1.82E-04 | 126.56 |
| Body mass index | rs1296328 | A | C | 0.0166 | 0.446 | 0.0017 | 3.49E-22 | 1.37E-04 | 95.35 |
| Body mass index | rs12981256 | A | G | 0.0151 | 0.528 | 0.0017 | 1.43E-18 | 1.14E-04 | 78.90 |
| Body mass index | rs12987009 | A | T | -0.0109 | 0.585 | 0.0017 | 1.83E-10 | 5.92E-05 | 41.11 |
| Body mass index | rs13021737 | A | G | -0.0578 | 0.157 | 0.0021 | 2.89E-161 | 1.09E-03 | 757.56 |
| Body mass index | rs13033310 | A | G | 0.0146 | 0.252 | 0.0022 | 3.40E-11 | 6.34E-05 | 44.04 |
| Body mass index | rs1304549 | A | G | -0.0118 | 0.226 | 0.0021 | 1.35E-08 | 4.55E-05 | 31.57 |
| Body mass index | rs13047416 | C | G | 0.0152 | 0.626 | 0.0018 | 6.93E-18 | 1.03E-04 | 71.31 |
| Body mass index | rs13107325 | T | C | 0.0468 | 0.082 | 0.0032 | 3.81E-47 | 3.08E-04 | 213.89 |
| Body mass index | rs13110266 | A | G | -0.0124 | 0.404 | 0.0016 | 3.96E-14 | 8.65E-05 | 60.06 |
| Body mass index | rs13174863 | A | G | -0.0197 | 0.854 | 0.0023 | 1.94E-17 | 1.06E-04 | 73.36 |
| Body mass index | rs13186194 | T | C | 0.01 | 0.617 | 0.0017 | 2.20E-09 | 4.98E-05 | 34.60 |
| Body mass index | rs13191362 | A | G | 0.0235 | 0.857 | 0.0025 | 4.08E-21 | 1.27E-04 | 88.36 |
| Body mass index | rs13245051 | A | G | 0.015 | 0.456 | 0.0017 | 1.12E-18 | 1.12E-04 | 77.85 |
| Body mass index | rs13249650 | A | T | 0.011 | 0.444 | 0.0019 | 1.02E-08 | 4.82E-05 | 33.52 |
| Body mass index | rs13263601 | A | C | -0.0145 | 0.659 | 0.0018 | 4.87E-16 | 9.34E-05 | 64.89 |
| Body mass index | rs1327259 | A | G | 0.0157 | 0.61 | 0.0017 | 1.47E-19 | 1.23E-04 | 85.29 |
| Body mass index | rs13296413 | T | C | -0.0148 | 0.381 | 0.0017 | 2.49E-17 | 1.09E-04 | 75.79 |
| Body mass index | rs13298487 | T | C | 0.0115 | 0.608 | 0.002 | 1.43E-08 | 4.76E-05 | 33.06 |
| Body mass index | rs1346841 | A | G | -0.0126 | 0.412 | 0.0017 | 3.18E-13 | 7.91E-05 | 54.93 |
| Body mass index | rs1350430 | T | C | -0.0125 | 0.533 | 0.0017 | 2.08E-13 | 7.78E-05 | 54.07 |
| Body mass index | rs1356506 | T | C | 0.0137 | 0.629 | 0.0018 | 8.39E-15 | 8.34E-05 | 57.93 |
| Body mass index | rs1358980 | T | C | -0.0129 | 0.474 | 0.0017 | 5.15E-15 | 8.29E-05 | 57.58 |
| Body mass index | rs1383592 | A | G | 0.0122 | 0.212 | 0.0021 | 4.92E-09 | 4.86E-05 | 33.75 |
| Body mass index | rs1409818 | T | C | 0.0195 | 0.107 | 0.0028 | 2.59E-12 | 6.98E-05 | 48.50 |
| Body mass index | rs1412235 | C | G | 0.0237 | 0.315 | 0.0017 | 2.28E-42 | 2.80E-04 | 194.36 |
| Body mass index | rs1421334 | A | C | 0.0135 | 0.455 | 0.0017 | 3.11E-15 | 9.08E-05 | 63.06 |
| Body mass index | rs1436344 | C | G | 0.0147 | 0.57 | 0.0017 | 1.07E-17 | 1.08E-04 | 74.77 |
| Body mass index | rs1437842 | A | G | -0.0106 | 0.491 | 0.0017 | 8.49E-10 | 5.60E-05 | 38.88 |
| Body mass index | rs1441264 | A | G | 0.0174 | 0.585 | 0.0017 | 7.60E-25 | 1.51E-04 | 104.76 |
| Body mass index | rs1451077 | A | G | -0.0169 | 0.578 | 0.0019 | 1.43E-18 | 1.14E-04 | 79.12 |
| Body mass index | rs147568678 | T | C | 0.0134 | 0.768 | 0.0023 | 3.42E-09 | 4.89E-05 | 33.94 |
| Body mass index | rs1477199 | A | G | -0.0221 | 0.843 | 0.0023 | 5.15E-21 | 1.33E-04 | 92.33 |
| Body mass index | rs1492014 | T | C | -0.0171 | 0.567 | 0.0017 | 1.44E-23 | 1.46E-04 | 101.18 |
| Body mass index | rs1492767 | T | C | 0.0095 | 0.468 | 0.0016 | 3.55E-09 | 5.07E-05 | 35.25 |
| Body mass index | rs1501673 | A | G | 0.0289 | 0.136 | 0.0025 | 2.73E-31 | 1.92E-04 | 133.63 |
| Body mass index | rs150215901 | A | T | -0.0275 | 0.041 | 0.0049 | 1.54E-08 | 4.53E-05 | 31.50 |
| Body mass index | rs1522569 | T | G | 0.0141 | 0.818 | 0.0022 | 1.60E-10 | 5.91E-05 | 41.08 |
| Body mass index | rs1559673 | A | C | 0.0359 | 0.969 | 0.0049 | 2.21E-13 | 7.73E-05 | 53.68 |
| Body mass index | rs156201 | C | G | 0.0124 | 0.746 | 0.0019 | 1.65E-10 | 6.13E-05 | 42.59 |
| Body mass index | rs1624134 | C | G | 0.01 | 0.393 | 0.0017 | 6.51E-09 | 4.98E-05 | 34.60 |
| Body mass index | rs16851483 | T | G | 0.0352 | 0.072 | 0.0034 | 4.87E-25 | 1.54E-04 | 107.18 |
| Body mass index | rs16906838 | T | C | -0.0247 | 0.049 | 0.004 | 4.31E-10 | 5.49E-05 | 38.13 |
| Body mass index | rs1700082 | C | G | 0.0094 | 0.659 | 0.0017 | 3.38E-08 | 4.40E-05 | 30.57 |
| Body mass index | rs17020497 | A | G | 0.014 | 0.134 | 0.0025 | 2.04E-08 | 4.51E-05 | 31.36 |
| Body mass index | rs17024393 | T | C | -0.0644 | 0.969 | 0.0049 | 7.11E-39 | 2.49E-04 | 172.73 |
| Body mass index | rs17094222 | T | C | -0.0173 | 0.791 | 0.002 | 4.04E-18 | 1.08E-04 | 74.82 |
| Body mass index | rs17182027 | A | G | -0.011 | 0.565 | 0.0017 | 1.46E-10 | 6.03E-05 | 41.87 |
| Body mass index | rs17207196 | T | C | -0.022 | 0.42 | 0.0017 | 1.58E-36 | 2.41E-04 | 167.47 |
| Body mass index | rs1721447 | T | G | -0.01 | 0.51 | 0.0017 | 3.79E-09 | 4.98E-05 | 34.60 |
| Body mass index | rs17367750 | T | C | -0.0122 | 0.312 | 0.0018 | 1.97E-11 | 6.61E-05 | 45.94 |
| Body mass index | rs17405603 | A | T | -0.0125 | 0.706 | 0.0019 | 4.43E-11 | 6.23E-05 | 43.28 |
| Body mass index | rs17405819 | T | C | 0.0211 | 0.685 | 0.0018 | 6.04E-33 | 1.98E-04 | 137.41 |
| Body mass index | rs1750307 | A | T | 0.0129 | 0.366 | 0.0018 | 2.78E-13 | 7.39E-05 | 51.36 |
| Body mass index | rs17544384 | T | C | -0.0131 | 0.795 | 0.0023 | 2.28E-08 | 4.67E-05 | 32.44 |
| Body mass index | rs17636031 | T | C | -0.0154 | 0.724 | 0.0018 | 3.87E-17 | 1.05E-04 | 73.20 |
| Body mass index | rs17681451 | A | G | -0.0225 | 0.076 | 0.0031 | 7.37E-13 | 7.58E-05 | 52.68 |
| Body mass index | rs17724992 | A | G | 0.0172 | 0.72 | 0.0018 | 5.23E-21 | 1.31E-04 | 91.31 |
| Body mass index | rs17783165 | T | C | -0.0128 | 0.671 | 0.0017 | 2.74E-13 | 8.16E-05 | 56.69 |
| Body mass index | rs17806224 | A | G | -0.026 | 0.181 | 0.0022 | 7.91E-32 | 2.01E-04 | 139.67 |
| Body mass index | rs17814208 | A | G | -0.0129 | 0.758 | 0.002 | 7.24E-11 | 5.99E-05 | 41.60 |
| Body mass index | rs1799923 | A | G | -0.0224 | 0.112 | 0.0026 | 1.12E-17 | 1.07E-04 | 74.22 |
| Body mass index | rs1808629 | A | G | -0.0202 | 0.674 | 0.002 | 2.19E-23 | 1.47E-04 | 102.01 |
| Body mass index | rs185350 | T | C | 0.0137 | 0.49 | 0.0016 | 9.10E-17 | 1.06E-04 | 73.32 |
| Body mass index | rs1860561 | A | G | 0.0159 | 0.209 | 0.0019 | 1.71E-16 | 1.01E-04 | 70.03 |
| Body mass index | rs1877875 | T | C | -0.0109 | 0.434 | 0.0017 | 2.51E-10 | 5.92E-05 | 41.11 |
| Body mass index | rs1884389 | T | C | -0.0108 | 0.439 | 0.0017 | 3.72E-10 | 5.81E-05 | 40.36 |
| Body mass index | rs1884897 | A | G | -0.0184 | 0.375 | 0.0017 | 2.70E-28 | 1.69E-04 | 117.15 |
| Body mass index | rs1927790 | T | C | -0.014 | 0.606 | 0.0016 | 1.57E-17 | 1.10E-04 | 76.56 |
| Body mass index | rs1928295 | T | C | 0.0134 | 0.571 | 0.0016 | 2.23E-16 | 1.01E-04 | 70.14 |
| Body mass index | rs1941213 | A | C | 0.0108 | 0.711 | 0.0019 | 1.59E-08 | 4.65E-05 | 32.31 |
| Body mass index | rs1941696 | A | G | 0.0112 | 0.517 | 0.0017 | 4.74E-11 | 6.25E-05 | 43.40 |
| Body mass index | rs1945160 | A | G | -0.0104 | 0.382 | 0.0018 | 5.65E-09 | 4.81E-05 | 33.38 |
| Body mass index | rs1948080 | T | G | 0.0136 | 0.63 | 0.0018 | 1.13E-14 | 8.22E-05 | 57.09 |
| Body mass index | rs194809 | A | G | 0.0126 | 0.187 | 0.0022 | 4.86E-09 | 4.72E-05 | 32.80 |
| Body mass index | rs1951455 | T | C | -0.0148 | 0.281 | 0.0019 | 6.05E-15 | 8.73E-05 | 60.68 |
| Body mass index | rs1958898 | C | G | -0.0146 | 0.21 | 0.0021 | 4.23E-12 | 6.96E-05 | 48.34 |
| Body mass index | rs1965529 | A | G | 0.0161 | 0.773 | 0.0022 | 7.66E-14 | 7.71E-05 | 53.56 |
| Body mass index | rs197374 | T | C | 0.0141 | 0.403 | 0.0017 | 3.16E-16 | 9.90E-05 | 68.79 |
| Body mass index | rs1999433 | T | C | -0.0107 | 0.44 | 0.0017 | 2.90E-10 | 5.70E-05 | 39.62 |
| Body mass index | rs2007518 | A | G | -0.013 | 0.562 | 0.0017 | 1.96E-14 | 8.42E-05 | 58.48 |
| Body mass index | rs2047648 | A | T | -0.0133 | 0.747 | 0.002 | 7.95E-12 | 6.37E-05 | 44.22 |
| Body mass index | rs2051559 | T | C | -0.0167 | 0.859 | 0.0025 | 3.78E-11 | 6.42E-05 | 44.62 |
| Body mass index | rs2053682 | A | C | 0.017 | 0.678 | 0.0018 | 2.59E-20 | 1.28E-04 | 89.20 |
| Body mass index | rs2058527 | T | G | -0.0115 | 0.272 | 0.0019 | 1.80E-09 | 5.27E-05 | 36.63 |
| Body mass index | rs2064044 | A | C | -0.0123 | 0.806 | 0.0021 | 1.02E-08 | 4.94E-05 | 34.31 |
| Body mass index | rs2065418 | T | G | 0.0139 | 0.646 | 0.0018 | 6.07E-15 | 8.58E-05 | 59.63 |
| Body mass index | rs2066295 | A | G | 0.0142 | 0.758 | 0.002 | 2.29E-12 | 7.26E-05 | 50.41 |
| Body mass index | rs2074314 | T | C | 0.0105 | 0.636 | 0.0017 | 1.37E-09 | 5.49E-05 | 38.15 |
| Body mass index | rs2108719 | A | G | 0.0107 | 0.727 | 0.0019 | 2.28E-08 | 4.57E-05 | 31.71 |
| Body mass index | rs2112347 | T | G | 0.0276 | 0.63 | 0.0017 | 1.17E-61 | 3.79E-04 | 263.58 |
| Body mass index | rs2119753 | A | G | 0.01 | 0.623 | 0.0017 | 1.00E-08 | 4.98E-05 | 34.60 |
| Body mass index | rs2120710 | A | G | 0.0101 | 0.653 | 0.0018 | 1.98E-08 | 4.53E-05 | 31.48 |
| Body mass index | rs2134858 | T | C | -0.0117 | 0.512 | 0.0017 | 5.87E-12 | 6.82E-05 | 47.37 |
| Body mass index | rs213518 | T | C | -0.0153 | 0.85 | 0.0024 | 2.49E-10 | 5.85E-05 | 40.64 |
| Body mass index | rs214249 | T | G | 0.0138 | 0.607 | 0.0017 | 2.72E-15 | 9.49E-05 | 65.90 |
| Body mass index | rs215669 | A | G | -0.0149 | 0.602 | 0.0017 | 8.94E-18 | 1.11E-04 | 76.82 |
| Body mass index | rs217433 | T | C | -0.0115 | 0.803 | 0.0021 | 3.00E-08 | 4.32E-05 | 29.99 |
| Body mass index | rs217669 | T | C | -0.0172 | 0.722 | 0.0021 | 6.27E-16 | 9.66E-05 | 67.08 |
| Body mass index | rs2192158 | A | G | 0.0137 | 0.449 | 0.0017 | 7.42E-16 | 9.35E-05 | 64.94 |
| Body mass index | rs2196618 | A | G | -0.0137 | 0.263 | 0.0019 | 1.49E-12 | 7.48E-05 | 51.99 |
| Body mass index | rs2206277 | T | C | 0.0408 | 0.156 | 0.0021 | 1.82E-83 | 5.43E-04 | 377.47 |
| Body mass index | rs2228213 | A | G | -0.0144 | 0.337 | 0.0017 | 5.50E-17 | 1.03E-04 | 71.75 |
| Body mass index | rs2228552 | T | G | 0.0124 | 0.566 | 0.0019 | 1.80E-11 | 6.13E-05 | 42.59 |
| Body mass index | rs2238799 | A | G | 0.0102 | 0.619 | 0.0018 | 8.65E-09 | 4.62E-05 | 32.11 |
| Body mass index | rs2241423 | A | G | -0.0298 | 0.229 | 0.0019 | 3.60E-54 | 3.54E-04 | 245.99 |
| Body mass index | rs2246012 | T | C | -0.0161 | 0.851 | 0.0022 | 1.15E-13 | 7.71E-05 | 53.56 |
| Body mass index | rs2257791 | A | G | -0.0139 | 0.756 | 0.002 | 1.63E-12 | 6.95E-05 | 48.30 |
| Body mass index | rs225882 | T | C | 0.0113 | 0.751 | 0.0018 | 8.56E-10 | 5.67E-05 | 39.41 |
| Body mass index | rs2267958 | A | G | -0.013 | 0.509 | 0.0018 | 2.01E-13 | 7.51E-05 | 52.16 |
| Body mass index | rs2271046 | A | T | -0.0115 | 0.695 | 0.0018 | 3.03E-10 | 5.88E-05 | 40.82 |
| Body mass index | rs2271189 | A | G | -0.0141 | 0.396 | 0.0018 | 9.26E-16 | 8.83E-05 | 61.36 |
| Body mass index | rs2273175 | T | C | -0.0121 | 0.679 | 0.0018 | 3.69E-11 | 6.50E-05 | 45.19 |
| Body mass index | rs2275003 | A | G | 0.0111 | 0.501 | 0.0016 | 9.44E-12 | 6.93E-05 | 48.13 |
| Body mass index | rs2281819 | A | T | -0.0154 | 0.229 | 0.002 | 2.09E-14 | 8.53E-05 | 59.29 |
| Body mass index | rs2283006 | A | G | 0.0132 | 0.488 | 0.0017 | 8.06E-15 | 8.68E-05 | 60.29 |
| Body mass index | rs2283093 | T | C | 0.0121 | 0.195 | 0.0021 | 1.10E-08 | 4.78E-05 | 33.20 |
| Body mass index | rs2289379 | T | C | -0.0137 | 0.392 | 0.0018 | 7.02E-15 | 8.34E-05 | 57.93 |
| Body mass index | rs2342892 | T | G | 0.0126 | 0.486 | 0.0017 | 1.33E-13 | 7.91E-05 | 54.93 |
| Body mass index | rs2357760 | A | G | 0.0143 | 0.674 | 0.0017 | 2.11E-16 | 1.02E-04 | 70.76 |
| Body mass index | rs2365389 | T | C | -0.0168 | 0.4 | 0.0016 | 6.49E-25 | 1.59E-04 | 110.25 |
| Body mass index | rs2396625 | A | T | -0.0176 | 0.418 | 0.0017 | 2.81E-24 | 1.54E-04 | 107.18 |
| Body mass index | rs2400414 | T | C | -0.0126 | 0.346 | 0.0018 | 5.52E-13 | 7.05E-05 | 49.00 |
| Body mass index | rs2423668 | T | C | 0.0106 | 0.42 | 0.0018 | 7.84E-09 | 4.99E-05 | 34.68 |
| Body mass index | rs2436728 | A | G | 0.0189 | 0.407 | 0.0017 | 1.97E-29 | 1.78E-04 | 123.60 |
| Body mass index | rs2439823 | A | G | -0.0165 | 0.451 | 0.0017 | 6.51E-22 | 1.36E-04 | 94.20 |
| Body mass index | rs2466103 | T | G | -0.0121 | 0.693 | 0.0018 | 8.00E-12 | 6.50E-05 | 45.19 |
| Body mass index | rs2470893 | T | C | 0.0107 | 0.305 | 0.0017 | 9.43E-10 | 5.70E-05 | 39.62 |
| Body mass index | rs2503185 | A | G | 0.013 | 0.51 | 0.0017 | 1.33E-14 | 8.42E-05 | 58.48 |
| Body mass index | rs2513999 | A | G | -0.0154 | 0.159 | 0.0026 | 3.07E-09 | 5.05E-05 | 35.08 |
| Body mass index | rs2600226 | T | C | -0.0116 | 0.671 | 0.0018 | 1.42E-10 | 5.98E-05 | 41.53 |
| Body mass index | rs2605603 | A | G | -0.0103 | 0.481 | 0.0016 | 2.04E-10 | 5.97E-05 | 41.44 |
| Body mass index | rs2612576 | A | T | -0.0108 | 0.295 | 0.0019 | 8.49E-09 | 4.65E-05 | 32.31 |
| Body mass index | rs2622274 | T | G | -0.0107 | 0.455 | 0.0017 | 3.23E-10 | 5.70E-05 | 39.62 |
| Body mass index | rs264941 | A | C | -0.0124 | 0.468 | 0.0017 | 1.84E-13 | 7.66E-05 | 53.20 |
| Body mass index | rs2707183 | T | G | -0.0093 | 0.532 | 0.0017 | 4.89E-08 | 4.31E-05 | 29.93 |
| Body mass index | rs2712665 | T | C | -0.0108 | 0.697 | 0.0019 | 5.40E-09 | 4.65E-05 | 32.31 |
| Body mass index | rs2715423 | A | G | -0.0115 | 0.281 | 0.0019 | 1.90E-09 | 5.27E-05 | 36.63 |
| Body mass index | rs273512 | T | C | 0.0156 | 0.409 | 0.0018 | 4.48E-19 | 1.08E-04 | 75.11 |
| Body mass index | rs2744974 | T | C | 0.0261 | 0.324 | 0.0017 | 1.28E-51 | 3.39E-04 | 235.71 |
| Body mass index | rs274628 | A | C | -0.0102 | 0.337 | 0.0018 | 1.36E-08 | 4.62E-05 | 32.11 |
| Body mass index | rs2777768 | A | G | 0.0119 | 0.723 | 0.0019 | 6.38E-10 | 5.65E-05 | 39.23 |
| Body mass index | rs2820295 | A | G | 0.0235 | 0.327 | 0.0018 | 5.56E-39 | 2.45E-04 | 170.45 |
| Body mass index | rs2832283 | A | G | 0.0115 | 0.225 | 0.002 | 4.72E-09 | 4.76E-05 | 33.06 |
| Body mass index | rs28350 | A | G | 0.0172 | 0.172 | 0.0022 | 1.07E-14 | 8.80E-05 | 61.12 |
| Body mass index | rs28489620 | A | G | -0.0151 | 0.282 | 0.0021 | 1.42E-12 | 7.44E-05 | 51.70 |
| Body mass index | rs2861089 | A | T | 0.0105 | 0.381 | 0.0017 | 1.55E-09 | 5.49E-05 | 38.15 |
| Body mass index | rs2861685 | T | C | 0.0165 | 0.586 | 0.0019 | 7.82E-18 | 1.09E-04 | 75.42 |
| Body mass index | rs2862996 | T | G | -0.0216 | 0.701 | 0.0017 | 3.60E-35 | 2.32E-04 | 161.44 |
| Body mass index | rs2875762 | C | G | 0.0129 | 0.252 | 0.002 | 1.07E-10 | 5.99E-05 | 41.60 |
| Body mass index | rs2907948 | A | G | -0.0145 | 0.244 | 0.0019 | 1.95E-14 | 8.38E-05 | 58.24 |
| Body mass index | rs2910026 | T | C | -0.0132 | 0.722 | 0.0021 | 5.69E-10 | 5.69E-05 | 39.51 |
| Body mass index | rs2962334 | T | G | 0.0396 | 0.027 | 0.0059 | 1.78E-11 | 6.48E-05 | 45.05 |
| Body mass index | rs2984618 | T | G | 0.0165 | 0.441 | 0.0016 | 3.80E-24 | 1.53E-04 | 106.35 |
| Body mass index | rs3019466 | T | C | -0.0128 | 0.168 | 0.0024 | 4.91E-08 | 4.09E-05 | 28.44 |
| Body mass index | rs305256 | T | C | -0.0115 | 0.226 | 0.0021 | 2.79E-08 | 4.32E-05 | 29.99 |
| Body mass index | rs3101336 | T | C | -0.0254 | 0.383 | 0.0016 | 4.80E-54 | 3.63E-04 | 252.01 |
| Body mass index | rs3115667 | T | C | -0.0179 | 0.282 | 0.0019 | 5.92E-21 | 1.28E-04 | 88.76 |
| Body mass index | rs312750 | A | G | 0.0097 | 0.499 | 0.0016 | 2.57E-09 | 5.29E-05 | 36.75 |
| Body mass index | rs321237 | A | G | 0.0126 | 0.75 | 0.002 | 1.49E-10 | 5.71E-05 | 39.69 |
| Body mass index | rs326893 | T | C | 0.0121 | 0.581 | 0.0017 | 1.89E-12 | 7.29E-05 | 50.66 |
| Body mass index | rs329651 | T | G | 0.016 | 0.806 | 0.0021 | 2.13E-14 | 8.36E-05 | 58.05 |
| Body mass index | rs337637 | A | G | -0.0137 | 0.359 | 0.0017 | 6.24E-16 | 9.35E-05 | 64.94 |
| Body mass index | rs339991 | A | G | -0.0125 | 0.42 | 0.0017 | 3.51E-13 | 7.78E-05 | 54.07 |
| Body mass index | rs34184235 | T | C | -0.0115 | 0.435 | 0.0019 | 2.14E-09 | 5.27E-05 | 36.63 |
| Body mass index | rs34234296 | A | G | -0.0145 | 0.388 | 0.002 | 2.01E-13 | 7.57E-05 | 52.56 |
| Body mass index | rs34517439 | A | C | 0.0391 | 0.117 | 0.003 | 3.40E-39 | 2.44E-04 | 169.87 |
| Body mass index | rs34811474 | A | G | -0.0293 | 0.222 | 0.0023 | 8.50E-38 | 2.34E-04 | 162.28 |
| Body mass index | rs349088 | A | C | -0.013 | 0.478 | 0.0017 | 3.50E-14 | 8.42E-05 | 58.48 |
| Body mass index | rs35408866 | A | G | 0.0159 | 0.134 | 0.0028 | 1.50E-08 | 4.64E-05 | 32.25 |
| Body mass index | rs35483388 | T | C | 0.0125 | 0.379 | 0.002 | 2.61E-10 | 5.62E-05 | 39.06 |
| Body mass index | rs355777 | C | G | 0.0151 | 0.398 | 0.0017 | 2.13E-18 | 1.14E-04 | 78.90 |
| Body mass index | rs35867081 | A | G | 0.015 | 0.487 | 0.0019 | 6.65E-15 | 8.97E-05 | 62.33 |
| Body mass index | rs35949039 | T | G | -0.0215 | 0.106 | 0.0031 | 6.30E-12 | 6.92E-05 | 48.10 |
| Body mass index | rs3732927 | T | C | 0.0099 | 0.291 | 0.0017 | 1.13E-08 | 4.88E-05 | 33.91 |
| Body mass index | rs3764625 | T | G | 0.0096 | 0.399 | 0.0017 | 2.42E-08 | 4.59E-05 | 31.89 |
| Body mass index | rs3764835 | A | G | -0.013 | 0.151 | 0.0024 | 3.84E-08 | 4.22E-05 | 29.34 |
| Body mass index | rs3770890 | T | G | -0.0297 | 0.973 | 0.0053 | 2.10E-08 | 4.52E-05 | 31.40 |
| Body mass index | rs3796432 | T | G | -0.0113 | 0.367 | 0.0018 | 2.21E-10 | 5.67E-05 | 39.41 |
| Body mass index | rs3806114 | A | G | -0.012 | 0.692 | 0.0018 | 1.46E-11 | 6.40E-05 | 44.44 |
| Body mass index | rs3808477 | T | C | -0.0182 | 0.274 | 0.0019 | 8.73E-22 | 1.32E-04 | 91.76 |
| Body mass index | rs3814883 | T | C | 0.0227 | 0.48 | 0.0017 | 1.47E-40 | 2.57E-04 | 178.30 |
| Body mass index | rs3825061 | T | C | 0.014 | 0.386 | 0.0017 | 6.15E-16 | 9.76E-05 | 67.82 |
| Body mass index | rs3914628 | T | C | 0.0165 | 0.859 | 0.0023 | 6.91E-13 | 7.41E-05 | 51.46 |
| Body mass index | rs3923783 | A | C | -0.0222 | 0.178 | 0.0022 | 4.12E-23 | 1.47E-04 | 101.83 |
| Body mass index | rs39654 | A | G | -0.0163 | 0.451 | 0.0017 | 1.75E-21 | 1.32E-04 | 91.93 |
| Body mass index | rs40067 | A | G | -0.0252 | 0.17 | 0.0023 | 9.73E-29 | 1.73E-04 | 120.05 |
| Body mass index | rs4017425 | T | C | -0.0118 | 0.468 | 0.0017 | 2.91E-12 | 6.94E-05 | 48.18 |
| Body mass index | rs4148155 | A | G | 0.0192 | 0.887 | 0.0026 | 1.34E-13 | 7.85E-05 | 54.53 |
| Body mass index | rs4240673 | T | C | 0.0175 | 0.455 | 0.0016 | 1.58E-26 | 1.72E-04 | 119.63 |
| Body mass index | rs4256980 | C | G | -0.0187 | 0.331 | 0.0017 | 8.63E-29 | 1.74E-04 | 121.00 |
| Body mass index | rs427943 | A | C | -0.0177 | 0.429 | 0.0017 | 3.60E-25 | 1.56E-04 | 108.40 |
| Body mass index | rs4286488 | A | G | 0.0121 | 0.761 | 0.002 | 1.70E-09 | 5.27E-05 | 36.60 |
| Body mass index | rs429358 | T | C | 0.0257 | 0.846 | 0.0026 | 2.60E-22 | 1.41E-04 | 97.71 |
| Body mass index | rs4303732 | T | C | 0.0169 | 0.614 | 0.0017 | 1.26E-22 | 1.42E-04 | 98.83 |
| Body mass index | rs4307239 | A | G | -0.0115 | 0.536 | 0.0017 | 1.47E-11 | 6.59E-05 | 45.76 |
| Body mass index | rs4430672 | T | C | 0.0124 | 0.196 | 0.0021 | 5.00E-09 | 5.02E-05 | 34.87 |
| Body mass index | rs4482463 | A | C | -0.031 | 0.917 | 0.0031 | 4.85E-23 | 1.44E-04 | 100.00 |
| Body mass index | rs4517716 | C | G | -0.0117 | 0.792 | 0.002 | 4.27E-09 | 4.93E-05 | 34.22 |
| Body mass index | rs45486197 | A | G | 0.0279 | 0.064 | 0.004 | 1.80E-12 | 7.00E-05 | 48.65 |
| Body mass index | rs459552 | A | T | -0.0133 | 0.775 | 0.0019 | 8.36E-12 | 7.05E-05 | 49.00 |
| Body mass index | rs4653017 | T | C | 0.0118 | 0.668 | 0.0018 | 1.11E-10 | 6.19E-05 | 42.98 |
| Body mass index | rs4655141 | T | C | -0.0173 | 0.836 | 0.0022 | 6.20E-15 | 8.90E-05 | 61.84 |
| Body mass index | rs4671328 | T | G | 0.0214 | 0.45 | 0.0017 | 3.42E-36 | 2.28E-04 | 158.46 |
| Body mass index | rs4700608 | T | C | -0.0155 | 0.524 | 0.0017 | 4.32E-20 | 1.20E-04 | 83.13 |
| Body mass index | rs4721089 | T | C | 0.0167 | 0.783 | 0.0023 | 5.60E-13 | 7.59E-05 | 52.72 |
| Body mass index | rs4740383 | A | G | 0.0131 | 0.419 | 0.0018 | 7.03E-14 | 7.62E-05 | 52.97 |
| Body mass index | rs4740619 | T | C | 0.0189 | 0.544 | 0.0016 | 3.15E-31 | 2.01E-04 | 139.53 |
| Body mass index | rs474605 | A | G | -0.0125 | 0.468 | 0.0017 | 2.72E-13 | 7.78E-05 | 54.07 |
| Body mass index | rs4771218 | A | G | -0.0141 | 0.624 | 0.0018 | 3.18E-15 | 8.83E-05 | 61.36 |
| Body mass index | rs478707 | T | C | -0.015 | 0.203 | 0.0024 | 1.98E-10 | 5.62E-05 | 39.06 |
| Body mass index | rs4812405 | A | C | -0.0202 | 0.076 | 0.0033 | 1.28E-09 | 5.39E-05 | 37.47 |
| Body mass index | rs4813619 | T | G | -0.0106 | 0.531 | 0.0018 | 1.70E-09 | 4.99E-05 | 34.68 |
| Body mass index | rs4858193 | T | C | 0.0133 | 0.72 | 0.0019 | 2.44E-12 | 7.05E-05 | 49.00 |
| Body mass index | rs4864201 | T | C | 0.0137 | 0.347 | 0.0017 | 4.30E-16 | 9.35E-05 | 64.94 |
| Body mass index | rs4865796 | A | G | -0.0096 | 0.704 | 0.0018 | 4.21E-08 | 4.09E-05 | 28.44 |
| Body mass index | rs4880341 | T | C | -0.013 | 0.573 | 0.0017 | 3.06E-14 | 8.42E-05 | 58.48 |
| Body mass index | rs4900714 | T | G | 0.015 | 0.478 | 0.0017 | 1.00E-18 | 1.12E-04 | 77.85 |
| Body mass index | rs4906263 | C | G | -0.0176 | 0.656 | 0.0018 | 8.11E-23 | 1.38E-04 | 95.60 |
| Body mass index | rs4921301 | T | C | -0.0129 | 0.213 | 0.0024 | 4.03E-08 | 4.16E-05 | 28.89 |
| Body mass index | rs4970991 | T | C | 0.0116 | 0.215 | 0.0021 | 1.81E-08 | 4.39E-05 | 30.51 |
| Body mass index | rs4973618 | A | G | -0.0148 | 0.662 | 0.0018 | 1.25E-16 | 9.73E-05 | 67.60 |
| Body mass index | rs4981693 | A | G | 0.0202 | 0.774 | 0.002 | 7.89E-24 | 1.47E-04 | 102.01 |
| Body mass index | rs4986044 | T | C | -0.0177 | 0.455 | 0.0016 | 1.27E-27 | 1.76E-04 | 122.38 |
| Body mass index | rs4988235 | A | G | 0.0124 | 0.721 | 0.0017 | 7.09E-13 | 7.66E-05 | 53.20 |
| Body mass index | rs543874 | A | G | -0.0479 | 0.779 | 0.002 | 3.06E-125 | 8.25E-04 | 573.60 |
| Body mass index | rs56133507 | T | G | -0.0131 | 0.807 | 0.0024 | 4.31E-08 | 4.29E-05 | 29.79 |
| Body mass index | rs56151256 | A | C | 0.0166 | 0.756 | 0.0022 | 6.19E-14 | 8.20E-05 | 56.93 |
| Body mass index | rs56161855 | A | T | -0.0233 | 0.868 | 0.0028 | 1.41E-16 | 9.97E-05 | 69.25 |
| Body mass index | rs56211164 | A | G | -0.0128 | 0.24 | 0.0022 | 8.90E-09 | 4.87E-05 | 33.85 |
| Body mass index | rs562664 | T | C | -0.0138 | 0.187 | 0.0022 | 4.15E-10 | 5.66E-05 | 39.35 |
| Body mass index | rs570463 | A | C | -0.0121 | 0.327 | 0.0018 | 4.33E-11 | 6.50E-05 | 45.19 |
| Body mass index | rs57989773 | T | C | -0.0142 | 0.758 | 0.0023 | 5.63E-10 | 5.49E-05 | 38.12 |
| Body mass index | rs587271 | T | C | 0.012 | 0.69 | 0.0018 | 6.21E-11 | 6.40E-05 | 44.44 |
| Body mass index | rs592483 | T | C | -0.0137 | 0.593 | 0.0017 | 1.53E-16 | 9.35E-05 | 64.94 |
| Body mass index | rs59302296 | A | T | 0.0217 | 0.099 | 0.0032 | 9.12E-12 | 6.62E-05 | 45.99 |
| Body mass index | rs6010784 | T | C | 0.0106 | 0.506 | 0.0016 | 6.91E-11 | 6.32E-05 | 43.89 |
| Body mass index | rs61740466 | A | G | -0.0142 | 0.235 | 0.0022 | 2.24E-10 | 6.00E-05 | 41.66 |
| Body mass index | rs61813324 | T | C | 0.0289 | 0.131 | 0.0028 | 3.20E-24 | 1.53E-04 | 106.53 |
| Body mass index | rs61828641 | A | G | 0.0223 | 0.109 | 0.003 | 2.49E-13 | 7.95E-05 | 55.25 |
| Body mass index | rs61983990 | A | G | 0.0198 | 0.08 | 0.0035 | 1.91E-08 | 4.61E-05 | 32.00 |
| Body mass index | rs62176243 | A | T | 0.0148 | 0.757 | 0.0022 | 2.41E-11 | 6.51E-05 | 45.26 |
| Body mass index | rs6265 | T | C | -0.0413 | 0.184 | 0.0021 | 7.40E-89 | 5.56E-04 | 386.78 |
| Body mass index | rs6443750 | T | C | -0.0152 | 0.199 | 0.0021 | 7.25E-13 | 7.54E-05 | 52.39 |
| Body mass index | rs6445258 | T | C | 0.0131 | 0.207 | 0.0023 | 2.48E-08 | 4.67E-05 | 32.44 |
| Body mass index | rs6463489 | T | C | 0.0167 | 0.096 | 0.0026 | 2.50E-10 | 5.94E-05 | 41.26 |
| Body mass index | rs6468266 | A | T | -0.0113 | 0.418 | 0.0017 | 7.71E-11 | 6.36E-05 | 44.18 |
| Body mass index | rs6470144 | T | G | 0.0098 | 0.65 | 0.0018 | 3.06E-08 | 4.27E-05 | 29.64 |
| Body mass index | rs6493498 | T | C | 0.0137 | 0.447 | 0.0016 | 4.84E-17 | 1.06E-04 | 73.32 |
| Body mass index | rs6496248 | A | T | 0.0104 | 0.635 | 0.0018 | 2.93E-09 | 4.81E-05 | 33.38 |
| Body mass index | rs6500208 | A | G | 0.0146 | 0.202 | 0.002 | 3.21E-13 | 7.67E-05 | 53.29 |
| Body mass index | rs650198 | T | C | -0.0137 | 0.729 | 0.0019 | 6.43E-13 | 7.48E-05 | 51.99 |
| Body mass index | rs6512302 | C | G | 0.0134 | 0.739 | 0.002 | 1.52E-11 | 6.46E-05 | 44.89 |
| Body mass index | rs6539064 | C | G | 0.0194 | 0.747 | 0.0019 | 1.14E-23 | 1.50E-04 | 104.25 |
| Body mass index | rs6545714 | A | G | -0.0194 | 0.611 | 0.0016 | 4.01E-32 | 2.12E-04 | 147.02 |
| Body mass index | rs6556301 | T | G | -0.0113 | 0.371 | 0.0017 | 8.14E-11 | 6.36E-05 | 44.18 |
| Body mass index | rs6567160 | T | C | -0.0552 | 0.752 | 0.0019 | 7.82E-184 | 1.21E-03 | 844.05 |
| Body mass index | rs657452 | A | G | 0.0188 | 0.404 | 0.0016 | 3.17E-30 | 1.99E-04 | 138.06 |
| Body mass index | rs6591407 | A | C | -0.0124 | 0.198 | 0.0021 | 3.58E-09 | 5.02E-05 | 34.87 |
| Body mass index | rs6607337 | T | C | -0.0124 | 0.298 | 0.0019 | 2.55E-11 | 6.13E-05 | 42.59 |
| Body mass index | rs6656785 | A | G | -0.0178 | 0.613 | 0.0016 | 3.06E-27 | 1.78E-04 | 123.77 |
| Body mass index | rs66595146 | A | C | 0.0135 | 0.629 | 0.002 | 9.09E-12 | 6.56E-05 | 45.56 |
| Body mass index | rs6661316 | T | C | 0.012 | 0.589 | 0.0016 | 1.72E-13 | 8.10E-05 | 56.25 |
| Body mass index | rs6696828 | C | G | 0.012 | 0.298 | 0.0018 | 6.42E-11 | 6.40E-05 | 44.44 |
| Body mass index | rs6710091 | C | G | 0.0103 | 0.666 | 0.0018 | 5.78E-09 | 4.71E-05 | 32.74 |
| Body mass index | rs6716898 | A | G | 0.0127 | 0.487 | 0.0019 | 2.33E-11 | 6.43E-05 | 44.68 |
| Body mass index | rs6720868 | T | C | 0.0154 | 0.308 | 0.0018 | 1.82E-17 | 1.05E-04 | 73.20 |
| Body mass index | rs6725931 | T | C | 0.0187 | 0.848 | 0.0024 | 2.30E-15 | 8.74E-05 | 60.71 |
| Body mass index | rs6783054 | A | C | 0.0099 | 0.503 | 0.0017 | 6.22E-09 | 4.88E-05 | 33.91 |
| Body mass index | rs6803161 | T | C | 0.0107 | 0.395 | 0.0019 | 2.71E-08 | 4.57E-05 | 31.71 |
| Body mass index | rs6804842 | A | G | -0.0141 | 0.43 | 0.0016 | 7.57E-18 | 1.12E-04 | 77.66 |
| Body mass index | rs6808814 | T | C | 0.0118 | 0.734 | 0.0019 | 1.31E-09 | 5.55E-05 | 38.57 |
| Body mass index | rs6850421 | A | G | 0.0113 | 0.456 | 0.0019 | 3.66E-09 | 5.09E-05 | 35.37 |
| Body mass index | rs6864049 | A | G | -0.0121 | 0.489 | 0.0016 | 1.48E-13 | 8.23E-05 | 57.19 |
| Body mass index | rs687339 | T | C | 0.0188 | 0.77 | 0.0019 | 4.32E-22 | 1.41E-04 | 97.91 |
| Body mass index | rs6882366 | T | C | -0.0131 | 0.396 | 0.0017 | 4.39E-14 | 8.55E-05 | 59.38 |
| Body mass index | rs6886072 | T | C | -0.01 | 0.467 | 0.0017 | 4.13E-09 | 4.98E-05 | 34.60 |
| Body mass index | rs6888194 | T | C | -0.0127 | 0.844 | 0.0023 | 3.58E-08 | 4.39E-05 | 30.49 |
| Body mass index | rs6890310 | A | G | -0.0119 | 0.292 | 0.0019 | 3.29E-10 | 5.65E-05 | 39.23 |
| Body mass index | rs6893539 | A | C | -0.0122 | 0.698 | 0.0019 | 6.17E-11 | 5.94E-05 | 41.23 |
| Body mass index | rs6909685 | T | C | -0.0149 | 0.33 | 0.0018 | 2.78E-16 | 9.86E-05 | 68.52 |
| Body mass index | rs6915002 | T | C | 0.0099 | 0.411 | 0.0017 | 8.71E-09 | 4.88E-05 | 33.91 |
| Body mass index | rs6921533 | T | C | 0.0104 | 0.293 | 0.0018 | 1.37E-08 | 4.81E-05 | 33.38 |
| Body mass index | rs6922607 | A | G | -0.013 | 0.808 | 0.0022 | 1.85E-09 | 5.03E-05 | 34.92 |
| Body mass index | rs6950388 | A | G | 0.0135 | 0.778 | 0.0022 | 1.59E-09 | 5.42E-05 | 37.65 |
| Body mass index | rs6973656 | A | G | -0.0101 | 0.584 | 0.0017 | 6.31E-09 | 5.08E-05 | 35.30 |
| Body mass index | rs698147 | A | G | 0.0116 | 0.447 | 0.0017 | 9.67E-12 | 6.70E-05 | 46.56 |
| Body mass index | rs7024334 | T | G | 0.0135 | 0.225 | 0.002 | 4.71E-12 | 6.56E-05 | 45.56 |
| Body mass index | rs7025938 | C | G | -0.0162 | 0.68 | 0.0018 | 1.47E-19 | 1.17E-04 | 81.00 |
| Body mass index | rs7070670 | T | C | -0.0126 | 0.323 | 0.0021 | 7.14E-10 | 5.18E-05 | 36.00 |
| Body mass index | rs7084454 | A | G | 0.0198 | 0.309 | 0.0018 | 4.51E-27 | 1.74E-04 | 121.00 |
| Body mass index | rs7102454 | T | C | -0.0168 | 0.644 | 0.0018 | 3.84E-21 | 1.25E-04 | 87.11 |
| Body mass index | rs7124681 | A | C | 0.0257 | 0.416 | 0.0016 | 3.96E-55 | 3.71E-04 | 258.00 |
| Body mass index | rs7138803 | A | G | 0.0297 | 0.387 | 0.0017 | 3.10E-71 | 4.39E-04 | 305.22 |
| Body mass index | rs7144011 | T | G | 0.0263 | 0.233 | 0.002 | 2.37E-40 | 2.49E-04 | 172.92 |
| Body mass index | rs7161194 | A | G | 0.019 | 0.344 | 0.0019 | 2.23E-24 | 1.44E-04 | 100.00 |
| Body mass index | rs7171864 | A | G | 0.0132 | 0.694 | 0.0017 | 2.60E-14 | 8.68E-05 | 60.29 |
| Body mass index | rs7172627 | A | G | -0.0114 | 0.523 | 0.0017 | 1.81E-11 | 6.47E-05 | 44.97 |
| Body mass index | rs7206608 | C | G | -0.013 | 0.675 | 0.0018 | 1.20E-12 | 7.51E-05 | 52.16 |
| Body mass index | rs7206790 | C | G | -0.0606 | 0.523 | 0.0017 | 1.00E-200 | 1.83E-03 | 1270.71 |
| Body mass index | rs7245985 | T | G | 0.0119 | 0.796 | 0.0021 | 1.88E-08 | 4.62E-05 | 32.11 |
| Body mass index | rs7258722 | A | T | -0.02 | 0.413 | 0.0017 | 1.81E-30 | 1.99E-04 | 138.41 |
| Body mass index | rs72649373 | T | C | -0.0167 | 0.862 | 0.0028 | 3.20E-09 | 5.12E-05 | 35.57 |
| Body mass index | rs72673947 | A | G | -0.0223 | 0.89 | 0.0031 | 4.11E-13 | 7.45E-05 | 51.75 |
| Body mass index | rs72757415 | T | G | -0.0159 | 0.212 | 0.0023 | 1.34E-11 | 6.88E-05 | 47.79 |
| Body mass index | rs73213484 | A | T | 0.0208 | 0.857 | 0.0027 | 2.04E-14 | 8.54E-05 | 59.35 |
| Body mass index | rs73225274 | A | G | -0.0156 | 0.865 | 0.0028 | 2.33E-08 | 4.47E-05 | 31.04 |
| Body mass index | rs7323 | C | G | -0.0166 | 0.268 | 0.0019 | 2.18E-18 | 1.10E-04 | 76.33 |
| Body mass index | rs73985439 | A | C | -0.0131 | 0.691 | 0.0021 | 1.84E-10 | 5.60E-05 | 38.91 |
| Body mass index | rs742748 | T | C | -0.0113 | 0.586 | 0.0017 | 1.13E-11 | 6.36E-05 | 44.18 |
| Body mass index | rs74887628 | A | G | 0.0305 | 0.034 | 0.0054 | 1.76E-08 | 4.59E-05 | 31.90 |
| Body mass index | rs7498665 | A | G | -0.0285 | 0.618 | 0.0017 | 1.14E-66 | 4.04E-04 | 281.05 |
| Body mass index | rs750090 | T | C | 0.0113 | 0.626 | 0.0018 | 2.50E-10 | 5.67E-05 | 39.41 |
| Body mass index | rs7512146 | T | G | -0.0097 | 0.527 | 0.0017 | 1.29E-08 | 4.69E-05 | 32.56 |
| Body mass index | rs7534091 | A | G | -0.012 | 0.718 | 0.0018 | 6.65E-11 | 6.40E-05 | 44.44 |
| Body mass index | rs7561278 | T | C | 0.0169 | 0.767 | 0.0021 | 4.89E-16 | 9.32E-05 | 64.76 |
| Body mass index | rs756717 | A | G | -0.0134 | 0.394 | 0.0017 | 2.38E-15 | 8.94E-05 | 62.13 |
| Body mass index | rs7568228 | C | G | -0.0104 | 0.54 | 0.0017 | 1.00E-09 | 5.39E-05 | 37.43 |
| Body mass index | rs7588437 | A | G | -0.0165 | 0.366 | 0.0017 | 2.32E-22 | 1.36E-04 | 94.20 |
| Body mass index | rs7593917 | A | G | -0.0115 | 0.454 | 0.0016 | 9.67E-13 | 7.44E-05 | 51.66 |
| Body mass index | rs7599312 | A | G | -0.0182 | 0.274 | 0.0018 | 1.52E-23 | 1.47E-04 | 102.23 |
| Body mass index | rs7616009 | A | G | -0.0157 | 0.16 | 0.0024 | 4.33E-11 | 6.16E-05 | 42.79 |
| Body mass index | rs7631156 | A | G | 0.0215 | 0.309 | 0.0018 | 3.33E-32 | 2.05E-04 | 142.67 |
| Body mass index | rs7640424 | T | C | -0.0135 | 0.311 | 0.0018 | 1.23E-14 | 8.10E-05 | 56.25 |
| Body mass index | rs765125 | T | C | -0.0098 | 0.586 | 0.0017 | 1.27E-08 | 4.78E-05 | 33.23 |
| Body mass index | rs765875 | T | C | -0.0132 | 0.469 | 0.0017 | 1.08E-14 | 8.68E-05 | 60.29 |
| Body mass index | rs76638898 | A | G | -0.0376 | 0.023 | 0.0065 | 8.15E-09 | 4.82E-05 | 33.46 |
| Body mass index | rs7678054 | A | G | -0.0099 | 0.479 | 0.0017 | 4.57E-09 | 4.88E-05 | 33.91 |
| Body mass index | rs768023 | A | G | 0.0161 | 0.603 | 0.0016 | 1.13E-22 | 1.46E-04 | 101.25 |
| Body mass index | rs7685628 | A | T | 0.0101 | 0.401 | 0.0017 | 6.15E-09 | 5.08E-05 | 35.30 |
| Body mass index | rs76942203 | A | G | 0.0263 | 0.059 | 0.0041 | 9.08E-11 | 5.92E-05 | 41.15 |
| Body mass index | rs7696649 | A | G | 0.0116 | 0.278 | 0.0019 | 4.73E-10 | 5.37E-05 | 37.27 |
| Body mass index | rs7713317 | A | G | -0.0166 | 0.72 | 0.0018 | 1.96E-20 | 1.22E-04 | 85.05 |
| Body mass index | rs7715256 | T | G | -0.0158 | 0.564 | 0.0016 | 3.98E-22 | 1.40E-04 | 97.52 |
| Body mass index | rs77165542 | T | C | -0.0939 | 0.034 | 0.0053 | 1.51E-70 | 4.52E-04 | 313.89 |
| Body mass index | rs7727781 | T | C | 0.0093 | 0.516 | 0.0017 | 4.27E-08 | 4.31E-05 | 29.93 |
| Body mass index | rs7730004 | T | C | 0.0139 | 0.661 | 0.0018 | 1.46E-14 | 8.58E-05 | 59.63 |
| Body mass index | rs7734385 | A | G | -0.0101 | 0.445 | 0.0016 | 6.08E-10 | 5.74E-05 | 39.85 |
| Body mass index | rs77432547 | A | G | -0.017 | 0.723 | 0.0021 | 1.44E-15 | 9.43E-05 | 65.53 |
| Body mass index | rs7760482 | A | G | -0.0102 | 0.616 | 0.0018 | 6.25E-09 | 4.62E-05 | 32.11 |
| Body mass index | rs7774 | A | C | 0.0128 | 0.32 | 0.0021 | 4.22E-10 | 5.35E-05 | 37.15 |
| Body mass index | rs7802342 | T | G | -0.0124 | 0.705 | 0.0019 | 6.23E-11 | 6.13E-05 | 42.59 |
| Body mass index | rs7842934 | T | C | -0.0178 | 0.921 | 0.0031 | 1.55E-08 | 4.75E-05 | 32.97 |
| Body mass index | rs7861160 | T | C | 0.0095 | 0.594 | 0.0017 | 3.68E-08 | 4.50E-05 | 31.23 |
| Body mass index | rs7893571 | T | G | 0.0125 | 0.674 | 0.0018 | 5.83E-12 | 6.94E-05 | 48.23 |
| Body mass index | rs7899106 | A | G | -0.0327 | 0.95 | 0.0037 | 1.72E-18 | 1.12E-04 | 78.11 |
| Body mass index | rs7903146 | T | C | -0.0178 | 0.279 | 0.0018 | 1.67E-23 | 1.41E-04 | 97.79 |
| Body mass index | rs7907470 | A | G | -0.0177 | 0.916 | 0.0031 | 7.36E-09 | 4.69E-05 | 32.60 |
| Body mass index | rs79186842 | A | G | 0.0202 | 0.86 | 0.0028 | 2.60E-13 | 7.49E-05 | 52.05 |
| Body mass index | rs7944782 | T | G | -0.0144 | 0.502 | 0.0017 | 3.61E-17 | 1.03E-04 | 71.75 |
| Body mass index | rs7975187 | A | G | -0.0137 | 0.771 | 0.0021 | 3.86E-11 | 6.13E-05 | 42.56 |
| Body mass index | rs79780963 | T | C | 0.0244 | 0.08 | 0.0035 | 2.63E-12 | 7.00E-05 | 48.60 |
| Body mass index | rs79906980 | T | C | 0.0158 | 0.156 | 0.0026 | 1.69E-09 | 5.32E-05 | 36.93 |
| Body mass index | rs805412 | A | G | -0.0098 | 0.434 | 0.0017 | 1.14E-08 | 4.78E-05 | 33.23 |
| Body mass index | rs8057911 | T | C | 0.0125 | 0.234 | 0.0023 | 3.66E-08 | 4.25E-05 | 29.54 |
| Body mass index | rs8065172 | A | G | -0.0124 | 0.238 | 0.002 | 5.29E-10 | 5.53E-05 | 38.44 |
| Body mass index | rs8097672 | A | T | -0.0209 | 0.855 | 0.0024 | 3.26E-18 | 1.09E-04 | 75.83 |
| Body mass index | rs8122855 | A | G | 0.0137 | 0.338 | 0.0018 | 4.11E-14 | 8.34E-05 | 57.93 |
| Body mass index | rs8126575 | T | G | 0.0152 | 0.867 | 0.0025 | 5.88E-10 | 5.32E-05 | 36.97 |
| Body mass index | rs8134638 | T | C | -0.0133 | 0.627 | 0.002 | 1.57E-11 | 6.37E-05 | 44.22 |
| Body mass index | rs8181823 | A | C | -0.0125 | 0.234 | 0.002 | 4.36E-10 | 5.62E-05 | 39.06 |
| Body mass index | rs845084 | A | G | 0.0136 | 0.267 | 0.0019 | 3.22E-12 | 7.38E-05 | 51.24 |
| Body mass index | rs852056 | T | C | 0.0123 | 0.254 | 0.0019 | 2.15E-10 | 6.03E-05 | 41.91 |
| Body mass index | rs865809 | A | G | 0.0124 | 0.225 | 0.002 | 6.50E-10 | 5.53E-05 | 38.44 |
| Body mass index | rs879620 | T | C | 0.0226 | 0.597 | 0.0017 | 8.45E-39 | 2.54E-04 | 176.73 |
| Body mass index | rs891387 | T | C | 0.0208 | 0.495 | 0.0017 | 9.26E-35 | 2.15E-04 | 149.70 |
| Body mass index | rs895330 | C | G | 0.0196 | 0.806 | 0.0022 | 1.59E-19 | 1.14E-04 | 79.37 |
| Body mass index | rs900144 | T | C | 0.0148 | 0.578 | 0.0017 | 1.53E-18 | 1.09E-04 | 75.79 |
| Body mass index | rs9168 | A | C | -0.0139 | 0.275 | 0.0019 | 1.08E-13 | 7.70E-05 | 53.52 |
| Body mass index | rs925018 | C | G | -0.013 | 0.667 | 0.0017 | 4.33E-14 | 8.42E-05 | 58.48 |
| Body mass index | rs9294260 | A | G | 0.014 | 0.469 | 0.0016 | 8.16E-18 | 1.10E-04 | 76.56 |
| Body mass index | rs9299 | T | C | 0.0119 | 0.645 | 0.0018 | 1.87E-11 | 6.29E-05 | 43.71 |
| Body mass index | rs930295 | A | C | 0.0208 | 0.155 | 0.0023 | 2.03E-19 | 1.18E-04 | 81.78 |
| Body mass index | rs9304665 | A | T | 0.023 | 0.742 | 0.002 | 2.20E-31 | 1.90E-04 | 132.25 |
| Body mass index | rs9320823 | T | C | -0.0165 | 0.412 | 0.0017 | 2.07E-21 | 1.36E-04 | 94.20 |
| Body mass index | rs935166 | A | G | -0.0152 | 0.495 | 0.0019 | 8.69E-16 | 9.21E-05 | 64.00 |
| Body mass index | rs9370410 | A | G | 0.0105 | 0.729 | 0.0019 | 2.58E-08 | 4.40E-05 | 30.54 |
| Body mass index | rs9375702 | T | C | -0.0106 | 0.679 | 0.0018 | 5.52E-09 | 4.99E-05 | 34.68 |
| Body mass index | rs942066 | A | G | -0.0202 | 0.373 | 0.002 | 2.36E-24 | 1.47E-04 | 102.01 |
| Body mass index | rs9458814 | T | C | -0.0112 | 0.771 | 0.002 | 2.33E-08 | 4.51E-05 | 31.36 |
| Body mass index | rs946824 | T | C | 0.0197 | 0.136 | 0.0025 | 4.82E-15 | 8.94E-05 | 62.09 |
| Body mass index | rs9478496 | T | C | -0.0157 | 0.845 | 0.0023 | 8.17E-12 | 6.71E-05 | 46.60 |
| Body mass index | rs9512648 | A | G | 0.0095 | 0.474 | 0.0017 | 4.11E-08 | 4.50E-05 | 31.23 |
| Body mass index | rs9522183 | T | G | -0.0135 | 0.549 | 0.0019 | 2.10E-12 | 7.27E-05 | 50.48 |
| Body mass index | rs9527895 | T | C | -0.0158 | 0.819 | 0.0023 | 3.69E-12 | 6.79E-05 | 47.19 |
| Body mass index | rs9531786 | C | G | -0.0111 | 0.372 | 0.0018 | 4.11E-10 | 5.47E-05 | 38.03 |
| Body mass index | rs9569777 | T | G | -0.0201 | 0.187 | 0.0021 | 3.43E-21 | 1.32E-04 | 91.61 |
| Body mass index | rs9595908 | T | C | 0.0154 | 0.643 | 0.0017 | 3.73E-20 | 1.18E-04 | 82.06 |
| Body mass index | rs9599161 | T | C | 0.0098 | 0.57 | 0.0016 | 2.32E-09 | 5.40E-05 | 37.52 |
| Body mass index | rs9603697 | T | C | 0.0134 | 0.318 | 0.0018 | 1.69E-13 | 7.98E-05 | 55.42 |
| Body mass index | rs9615337 | C | G | -0.011 | 0.575 | 0.002 | 2.05E-08 | 4.35E-05 | 30.25 |
| Body mass index | rs962796 | T | C | 0.014 | 0.194 | 0.0021 | 1.88E-11 | 6.40E-05 | 44.44 |
| Body mass index | rs9816226 | A | T | -0.0315 | 0.176 | 0.0021 | 1.45E-50 | 3.24E-04 | 225.00 |
| Body mass index | rs9818122 | T | C | -0.0228 | 0.793 | 0.002 | 3.97E-30 | 1.87E-04 | 129.96 |
| Body mass index | rs9826775 | A | G | 0.0155 | 0.851 | 0.0024 | 6.61E-11 | 6.00E-05 | 41.71 |
| Body mass index | rs9827823 | T | C | 0.0182 | 0.855 | 0.0023 | 3.39E-15 | 9.01E-05 | 62.62 |
| Body mass index | rs9888533 | T | C | 0.0125 | 0.53 | 0.0019 | 1.38E-10 | 6.23E-05 | 43.28 |
| Body mass index | rs9926784 | T | C | 0.0237 | 0.801 | 0.0021 | 1.08E-30 | 1.83E-04 | 127.37 |
| Body mass index | rs9944219 | A | G | -0.0119 | 0.604 | 0.0017 | 8.14E-13 | 7.05E-05 | 49.00 |
| Body mass index | rs994596 | T | C | 0.013 | 0.325 | 0.0018 | 1.06E-12 | 7.51E-05 | 52.16 |
| Body mass index | rs9951619 | T | G | -0.0152 | 0.224 | 0.0019 | 2.33E-15 | 9.21E-05 | 64.00 |
| Body mass index | rs9992189 | C | G | -0.0097 | 0.608 | 0.0017 | 2.04E-08 | 4.69E-05 | 32.56 |
| Waist-to-hip ratio | rs10842707 | T | C | 0.036 | 0.227 | 0.005 | 4.39E-15 | 3.63E-04 | 51.84 |
| Waist-to-hip ratio | rs1128249 | G | T | 0.032 | 0.589 | 0.004 | 7.51E-16 | 4.48E-04 | 64.00 |
| Waist-to-hip ratio | rs12143789 | C | G | 0.03 | 0.203 | 0.005 | 8.29E-10 | 2.52E-04 | 36.00 |
| Waist-to-hip ratio | rs12489828 | T | G | -0.022 | 0.546 | 0.004 | 1.32E-08 | 2.12E-04 | 30.25 |
| Waist-to-hip ratio | rs1385167 | G | A | 0.032 | 0.142 | 0.006 | 9.40E-09 | 1.99E-04 | 28.44 |
| Waist-to-hip ratio | rs1443512 | A | C | 0.031 | 0.233 | 0.005 | 5.38E-12 | 2.69E-04 | 38.44 |
| Waist-to-hip ratio | rs17451107 | T | C | 0.027 | 0.614 | 0.004 | 1.48E-10 | 3.19E-04 | 45.56 |
| Waist-to-hip ratio | rs1936805 | T | C | 0.042 | 0.509 | 0.004 | 6.13E-28 | 7.72E-04 | 110.25 |
| Waist-to-hip ratio | rs2294239 | A | G | 0.028 | 0.586 | 0.004 | 1.86E-12 | 3.43E-04 | 49.00 |
| Waist-to-hip ratio | rs2371767 | G | C | 0.034 | 0.723 | 0.005 | 9.49E-14 | 3.24E-04 | 46.24 |
| Waist-to-hip ratio | rs2645294 | T | C | 0.034 | 0.575 | 0.004 | 4.42E-18 | 5.06E-04 | 72.25 |
| Waist-to-hip ratio | rs2820443 | T | C | 0.041 | 0.714 | 0.004 | 1.50E-21 | 7.35E-04 | 105.06 |
| Waist-to-hip ratio | rs3936510 | T | G | 0.029 | 0.183 | 0.005 | 2.60E-08 | 2.36E-04 | 33.64 |
| Waist-to-hip ratio | rs4646404 | G | A | 0.026 | 0.665 | 0.005 | 3.46E-08 | 1.89E-04 | 27.04 |
| Waist-to-hip ratio | rs4765219 | C | A | 0.03 | 0.667 | 0.004 | 5.99E-14 | 3.94E-04 | 56.25 |
| Waist-to-hip ratio | rs7705502 | A | G | 0.023 | 0.32 | 0.004 | 2.38E-08 | 2.32E-04 | 33.06 |
| Waist-to-hip ratio | rs905938 | T | C | 0.03 | 0.738 | 0.005 | 1.73E-10 | 2.52E-04 | 36.00 |
| Waist-to-hip ratio | rs979012 | T | C | 0.026 | 0.347 | 0.004 | 4.22E-10 | 2.96E-04 | 42.25 |
| Waist-to-hip ratio | rs998584 | A | C | 0.044 | 0.483 | 0.005 | 3.09E-21 | 5.42E-04 | 77.44 |
| Type 2 diabetes | rs10097617 | C | T | -0.037 | 0.52 | 0.005 | 1.93E-16 | 4.91E-05 | 54.76 |
| Type 2 diabetes | rs10137475 | G | A | 0.026 | 0.426 | 0.005 | 4.43E-08 | 2.43E-05 | 27.04 |
| Type 2 diabetes | rs10145154 | C | T | -0.055 | 0.784 | 0.006 | 1.06E-22 | 7.54E-05 | 84.03 |
| Type 2 diabetes | rs10188334 | C | T | 0.05 | 0.828 | 0.006 | 7.76E-16 | 6.23E-05 | 69.44 |
| Type 2 diabetes | rs10240790 | G | A | 0.028 | 0.704 | 0.005 | 4.69E-08 | 2.81E-05 | 31.36 |
| Type 2 diabetes | rs10404726 | C | T | 0.028 | 0.531 | 0.005 | 2.73E-09 | 2.81E-05 | 31.36 |
| Type 2 diabetes | rs10406327 | G | C | -0.037 | 0.475 | 0.005 | 8.75E-16 | 4.91E-05 | 54.76 |
| Type 2 diabetes | rs10407429 | G | A | 0.054 | 0.579 | 0.005 | 4.79E-31 | 1.05E-04 | 116.64 |
| Type 2 diabetes | rs10471048 | G | C | 0.034 | 0.349 | 0.005 | 2.31E-12 | 4.15E-05 | 46.24 |
| Type 2 diabetes | rs10490871 | G | A | 0.027 | 0.369 | 0.005 | 6.58E-09 | 2.62E-05 | 29.16 |
| Type 2 diabetes | rs1059592 | G | A | -0.027 | 0.646 | 0.005 | 2.40E-08 | 2.62E-05 | 29.16 |
| Type 2 diabetes | rs10750397 | G | A | -0.048 | 0.71 | 0.005 | 1.01E-20 | 8.27E-05 | 92.16 |
| Type 2 diabetes | rs10771372 | C | T | 0.072 | 0.805 | 0.006 | 2.20E-35 | 1.29E-04 | 144.00 |
| Type 2 diabetes | rs10811661 | C | T | -0.138 | 0.172 | 0.006 | 8.14E-112 | 4.74E-04 | 529.00 |
| Type 2 diabetes | rs10821311 | G | A | -0.036 | 0.681 | 0.005 | 4.00E-13 | 4.65E-05 | 51.84 |
| Type 2 diabetes | rs10830963 | G | C | 0.089 | 0.278 | 0.005 | 4.57E-68 | 2.84E-04 | 316.84 |
| Type 2 diabetes | rs10841868 | G | T | 0.032 | 0.739 | 0.005 | 1.65E-09 | 3.68E-05 | 40.96 |
| Type 2 diabetes | rs10915188 | G | A | -0.029 | 0.415 | 0.005 | 3.69E-10 | 3.02E-05 | 33.64 |
| Type 2 diabetes | rs10937721 | G | C | -0.084 | 0.412 | 0.005 | 6.31E-70 | 2.53E-04 | 282.24 |
| Type 2 diabetes | rs10938398 | G | A | -0.043 | 0.569 | 0.005 | 3.91E-20 | 6.64E-05 | 73.96 |
| Type 2 diabetes | rs10963942 | G | A | 0.037 | 0.397 | 0.005 | 4.49E-15 | 4.91E-05 | 54.76 |
| Type 2 diabetes | rs10974438 | C | A | 0.047 | 0.356 | 0.005 | 8.99E-23 | 7.93E-05 | 88.36 |
| Type 2 diabetes | rs10998304 | C | T | 0.031 | 0.452 | 0.005 | 1.77E-11 | 3.45E-05 | 38.44 |
| Type 2 diabetes | rs11038672 | G | C | -0.029 | 0.524 | 0.005 | 1.04E-10 | 3.02E-05 | 33.64 |
| Type 2 diabetes | rs1111875 | C | T | 0.092 | 0.592 | 0.005 | 5.21E-89 | 3.04E-04 | 338.56 |
| Type 2 diabetes | rs11129735 | G | A | -0.026 | 0.538 | 0.005 | 1.53E-08 | 2.43E-05 | 27.04 |
| Type 2 diabetes | rs11201999 | C | T | 0.026 | 0.537 | 0.005 | 1.25E-08 | 2.43E-05 | 27.04 |
| Type 2 diabetes | rs11257655 | C | T | -0.091 | 0.781 | 0.007 | 7.25E-35 | 1.52E-04 | 169.00 |
| Type 2 diabetes | rs112667817 | C | T | 0.061 | 0.879 | 0.011 | 5.76E-09 | 2.76E-05 | 30.75 |
| Type 2 diabetes | rs1127215 | C | T | 0.043 | 0.585 | 0.005 | 1.93E-20 | 6.64E-05 | 73.96 |
| Type 2 diabetes | rs113036477 | C | T | 0.072 | 0.938 | 0.01 | 4.09E-13 | 4.65E-05 | 51.84 |
| Type 2 diabetes | rs114136102 | C | T | 0.072 | 0.04 | 0.012 | 2.05E-09 | 3.23E-05 | 36.00 |
| Type 2 diabetes | rs114447556 | C | T | -0.058 | 0.917 | 0.009 | 7.79E-11 | 3.73E-05 | 41.53 |
| Type 2 diabetes | rs11558471 | G | A | -0.103 | 0.316 | 0.005 | 3.98E-98 | 3.81E-04 | 424.36 |
| Type 2 diabetes | rs11602873 | T | A | -0.098 | 0.156 | 0.006 | 8.91E-52 | 2.39E-04 | 266.78 |
| Type 2 diabetes | rs11616380 | G | T | 0.079 | 0.721 | 0.005 | 7.99E-54 | 2.24E-04 | 249.64 |
| Type 2 diabetes | rs11657964 | G | A | -0.059 | 0.596 | 0.005 | 1.23E-36 | 1.25E-04 | 139.24 |
| Type 2 diabetes | rs11680058 | G | A | -0.056 | 0.162 | 0.01 | 1.23E-08 | 2.81E-05 | 31.36 |
| Type 2 diabetes | rs11708067 | G | A | -0.078 | 0.229 | 0.006 | 1.50E-46 | 1.52E-04 | 169.00 |
| Type 2 diabetes | rs11716527 | C | T | 0.049 | 0.095 | 0.009 | 6.69E-09 | 2.66E-05 | 29.64 |
| Type 2 diabetes | rs117233107 | G | A | 0.325 | 0.983 | 0.021 | 4.42E-55 | 2.15E-04 | 239.51 |
| Type 2 diabetes | rs117316450 | G | C | 0.131 | 0.019 | 0.018 | 2.07E-13 | 4.75E-05 | 52.97 |
| Type 2 diabetes | rs11759026 | G | A | 0.065 | 0.231 | 0.007 | 6.68E-19 | 7.74E-05 | 86.22 |
| Type 2 diabetes | rs11793831 | G | T | -0.027 | 0.595 | 0.005 | 4.21E-09 | 2.62E-05 | 29.16 |
| Type 2 diabetes | rs11830243 | C | T | -0.044 | 0.887 | 0.007 | 2.10E-09 | 3.55E-05 | 39.51 |
| Type 2 diabetes | rs11870735 | C | T | -0.034 | 0.818 | 0.006 | 1.68E-08 | 2.88E-05 | 32.11 |
| Type 2 diabetes | rs11940813 | G | A | 0.037 | 0.137 | 0.007 | 2.65E-08 | 2.51E-05 | 27.94 |
| Type 2 diabetes | rs11967262 | G | C | 0.037 | 0.488 | 0.005 | 2.23E-15 | 4.91E-05 | 54.76 |
| Type 2 diabetes | rs12001437 | C | T | 0.034 | 0.373 | 0.005 | 6.27E-13 | 4.15E-05 | 46.24 |
| Type 2 diabetes | rs12263348 | C | T | -0.028 | 0.662 | 0.005 | 7.77E-09 | 2.81E-05 | 31.36 |
| Type 2 diabetes | rs12305809 | G | A | 0.033 | 0.607 | 0.005 | 1.45E-12 | 3.91E-05 | 43.56 |
| Type 2 diabetes | rs12505942 | C | T | -0.03 | 0.339 | 0.005 | 7.03E-10 | 3.23E-05 | 36.00 |
| Type 2 diabetes | rs12509379 | G | T | -0.031 | 0.788 | 0.006 | 2.66E-08 | 2.40E-05 | 26.69 |
| Type 2 diabetes | rs12519500 | C | A | 0.038 | 0.654 | 0.005 | 1.76E-15 | 5.18E-05 | 57.76 |
| Type 2 diabetes | rs1260326 | C | T | 0.064 | 0.595 | 0.005 | 2.55E-42 | 1.47E-04 | 163.84 |
| Type 2 diabetes | rs12625671 | C | T | 0.065 | 0.114 | 0.007 | 6.74E-19 | 7.74E-05 | 86.22 |
| Type 2 diabetes | rs12741141 | G | C | 0.04 | 0.362 | 0.005 | 1.37E-16 | 5.74E-05 | 64.00 |
| Type 2 diabetes | rs12820906 | G | A | -0.043 | 0.244 | 0.006 | 2.21E-15 | 4.61E-05 | 51.36 |
| Type 2 diabetes | rs12823740 | C | A | 0.041 | 0.661 | 0.005 | 1.48E-17 | 6.03E-05 | 67.24 |
| Type 2 diabetes | rs12890750 | G | T | 0.028 | 0.639 | 0.005 | 2.84E-09 | 2.81E-05 | 31.36 |
| Type 2 diabetes | rs12910361 | G | A | 0.072 | 0.698 | 0.005 | 2.97E-44 | 1.86E-04 | 207.36 |
| Type 2 diabetes | rs12912777 | C | T | -0.059 | 0.879 | 0.008 | 1.75E-15 | 4.88E-05 | 54.39 |
| Type 2 diabetes | rs13237518 | C | A | -0.029 | 0.586 | 0.005 | 7.73E-10 | 3.02E-05 | 33.64 |
| Type 2 diabetes | rs13262861 | C | A | 0.102 | 0.824 | 0.006 | 2.84E-59 | 2.59E-04 | 289.00 |
| Type 2 diabetes | rs13389219 | C | T | 0.065 | 0.603 | 0.005 | 7.35E-44 | 1.52E-04 | 169.00 |
| Type 2 diabetes | rs13414140 | C | T | 0.118 | 0.886 | 0.007 | 4.31E-60 | 2.55E-04 | 284.16 |
| Type 2 diabetes | rs1412234 | C | T | 0.044 | 0.319 | 0.005 | 4.00E-19 | 6.95E-05 | 77.44 |
| Type 2 diabetes | rs1449348 | C | T | 0.045 | 0.859 | 0.007 | 6.64E-12 | 3.71E-05 | 41.33 |
| Type 2 diabetes | rs1475655 | T | A | -0.044 | 0.253 | 0.005 | 2.47E-16 | 6.95E-05 | 77.44 |
| Type 2 diabetes | rs1493694 | C | T | -0.071 | 0.893 | 0.007 | 6.51E-22 | 9.23E-05 | 102.88 |
| Type 2 diabetes | rs1513272 | C | T | 0.081 | 0.509 | 0.005 | 3.97E-71 | 2.35E-04 | 262.44 |
| Type 2 diabetes | rs1531583 | G | T | -0.099 | 0.955 | 0.011 | 7.06E-19 | 7.27E-05 | 81.00 |
| Type 2 diabetes | rs1561927 | C | T | 0.035 | 0.282 | 0.005 | 8.61E-12 | 4.40E-05 | 49.00 |
| Type 2 diabetes | rs1562398 | G | C | 0.041 | 0.417 | 0.005 | 1.99E-18 | 6.03E-05 | 67.24 |
| Type 2 diabetes | rs1573090 | G | T | -0.045 | 0.465 | 0.005 | 3.88E-22 | 7.27E-05 | 81.00 |
| Type 2 diabetes | rs17035289 | C | T | 0.043 | 0.171 | 0.006 | 5.71E-12 | 4.61E-05 | 51.36 |
| Type 2 diabetes | rs17036160 | C | T | 0.103 | 0.88 | 0.007 | 3.04E-47 | 1.94E-04 | 216.51 |
| Type 2 diabetes | rs1705263 | C | A | 0.04 | 0.569 | 0.005 | 1.22E-17 | 5.74E-05 | 64.00 |
| Type 2 diabetes | rs17294565 | C | A | 0.027 | 0.381 | 0.005 | 4.74E-09 | 2.62E-05 | 29.16 |
| Type 2 diabetes | rs17354348 | G | A | -0.029 | 0.258 | 0.005 | 2.99E-08 | 3.02E-05 | 33.64 |
| Type 2 diabetes | rs174541 | C | T | -0.029 | 0.354 | 0.005 | 8.63E-10 | 3.02E-05 | 33.64 |
| Type 2 diabetes | rs17522122 | G | T | -0.034 | 0.524 | 0.005 | 1.27E-13 | 4.15E-05 | 46.24 |
| Type 2 diabetes | rs17624303 | C | T | 0.029 | 0.725 | 0.005 | 4.88E-08 | 3.02E-05 | 33.64 |
| Type 2 diabetes | rs17684074 | G | C | 0.031 | 0.739 | 0.005 | 4.12E-09 | 3.45E-05 | 38.44 |
| Type 2 diabetes | rs17772814 | G | A | 0.075 | 0.915 | 0.013 | 3.44E-09 | 2.99E-05 | 33.28 |
| Type 2 diabetes | rs17791513 | G | A | -0.069 | 0.066 | 0.01 | 2.17E-12 | 4.27E-05 | 47.61 |
| Type 2 diabetes | rs1783541 | C | T | -0.048 | 0.797 | 0.006 | 3.89E-17 | 5.74E-05 | 64.00 |
| Type 2 diabetes | rs1872635 | G | A | -0.028 | 0.314 | 0.005 | 1.28E-08 | 2.81E-05 | 31.36 |
| Type 2 diabetes | rs1968204 | C | T | -0.057 | 0.893 | 0.008 | 1.08E-12 | 4.55E-05 | 50.77 |
| Type 2 diabetes | rs2011603 | G | A | -0.038 | 0.275 | 0.005 | 1.59E-13 | 5.18E-05 | 57.76 |
| Type 2 diabetes | rs2033159 | C | A | 0.035 | 0.234 | 0.006 | 4.90E-10 | 3.05E-05 | 34.03 |
| Type 2 diabetes | rs2055997 | G | A | 0.031 | 0.701 | 0.005 | 6.53E-10 | 3.45E-05 | 38.44 |
| Type 2 diabetes | rs2080090 | T | A | -0.053 | 0.812 | 0.006 | 7.84E-19 | 7.00E-05 | 78.03 |
| Type 2 diabetes | rs2103132 | G | C | -0.032 | 0.749 | 0.005 | 1.88E-09 | 3.68E-05 | 40.96 |
| Type 2 diabetes | rs2115107 | G | A | -0.038 | 0.608 | 0.005 | 8.45E-16 | 5.18E-05 | 57.76 |
| Type 2 diabetes | rs2191349 | G | T | -0.066 | 0.461 | 0.005 | 6.41E-48 | 1.56E-04 | 174.24 |
| Type 2 diabetes | rs2237895 | C | A | 0.073 | 0.423 | 0.005 | 2.73E-54 | 1.91E-04 | 213.16 |
| Type 2 diabetes | rs2250301 | G | A | 0.032 | 0.748 | 0.005 | 1.27E-09 | 3.68E-05 | 40.96 |
| Type 2 diabetes | rs2268078 | G | A | -0.039 | 0.345 | 0.005 | 2.14E-15 | 5.46E-05 | 60.84 |
| Type 2 diabetes | rs2292662 | C | T | 0.056 | 0.844 | 0.007 | 7.64E-18 | 5.74E-05 | 64.00 |
| Type 2 diabetes | rs2297508 | G | C | -0.033 | 0.632 | 0.005 | 1.86E-12 | 3.91E-05 | 43.56 |
| Type 2 diabetes | rs2410767 | G | C | -0.033 | 0.221 | 0.006 | 6.79E-09 | 2.71E-05 | 30.25 |
| Type 2 diabetes | rs243018 | G | C | 0.056 | 0.463 | 0.005 | 1.52E-33 | 1.13E-04 | 125.44 |
| Type 2 diabetes | rs256904 | T | A | 0.069 | 0.73 | 0.005 | 2.55E-39 | 1.71E-04 | 190.44 |
| Type 2 diabetes | rs2583921 | C | A | 0.095 | 0.092 | 0.008 | 8.98E-33 | 1.27E-04 | 141.02 |
| Type 2 diabetes | rs2613503 | C | A | -0.039 | 0.199 | 0.006 | 7.48E-11 | 3.79E-05 | 42.25 |
| Type 2 diabetes | rs2658746 | C | T | 0.03 | 0.39 | 0.005 | 9.89E-11 | 3.23E-05 | 36.00 |
| Type 2 diabetes | rs2725371 | G | A | -0.037 | 0.69 | 0.005 | 2.14E-13 | 4.91E-05 | 54.76 |
| Type 2 diabetes | rs2732480 | C | A | 0.034 | 0.565 | 0.005 | 3.05E-13 | 4.15E-05 | 46.24 |
| Type 2 diabetes | rs2733289 | C | T | 0.03 | 0.478 | 0.005 | 3.98E-11 | 3.23E-05 | 36.00 |
| Type 2 diabetes | rs2796441 | G | A | 0.059 | 0.591 | 0.005 | 9.96E-37 | 1.25E-04 | 139.24 |
| Type 2 diabetes | rs28429551 | T | A | -0.073 | 0.26 | 0.006 | 9.51E-40 | 1.33E-04 | 148.03 |
| Type 2 diabetes | rs2908286 | C | T | -0.068 | 0.829 | 0.006 | 2.64E-29 | 1.15E-04 | 128.44 |
| Type 2 diabetes | rs2933211 | G | A | -0.027 | 0.499 | 0.005 | 6.24E-09 | 2.62E-05 | 29.16 |
| Type 2 diabetes | rs2972145 | C | T | 0.09 | 0.635 | 0.005 | 7.45E-80 | 2.91E-04 | 324.00 |
| Type 2 diabetes | rs303760 | C | T | -0.034 | 0.652 | 0.005 | 1.48E-12 | 4.15E-05 | 46.24 |
| Type 2 diabetes | rs3094682 | C | A | 0.06 | 0.806 | 0.006 | 2.37E-23 | 8.97E-05 | 100.00 |
| Type 2 diabetes | rs3111316 | G | A | -0.044 | 0.412 | 0.005 | 3.43E-21 | 6.95E-05 | 77.44 |
| Type 2 diabetes | rs314879 | C | T | 0.039 | 0.226 | 0.006 | 5.74E-12 | 3.79E-05 | 42.25 |
| Type 2 diabetes | rs3176466 | C | T | 0.064 | 0.903 | 0.008 | 9.36E-16 | 5.74E-05 | 64.00 |
| Type 2 diabetes | rs340874 | C | T | 0.067 | 0.553 | 0.005 | 5.37E-48 | 1.61E-04 | 179.56 |
| Type 2 diabetes | rs34143602 | G | A | 0.035 | 0.422 | 0.005 | 1.00E-13 | 4.40E-05 | 49.00 |
| Type 2 diabetes | rs34506349 | G | A | 0.068 | 0.96 | 0.012 | 1.76E-08 | 2.88E-05 | 32.11 |
| Type 2 diabetes | rs34589210 | G | A | -0.039 | 0.857 | 0.007 | 6.89E-09 | 2.79E-05 | 31.04 |
| Type 2 diabetes | rs346240 | G | A | 0.031 | 0.223 | 0.006 | 3.62E-08 | 2.40E-05 | 26.69 |
| Type 2 diabetes | rs348330 | G | A | 0.053 | 0.369 | 0.007 | 6.50E-16 | 5.14E-05 | 57.33 |
| Type 2 diabetes | rs34990153 | G | A | -0.038 | 0.439 | 0.005 | 1.99E-16 | 5.18E-05 | 57.76 |
| Type 2 diabetes | rs35004890 | G | T | -0.036 | 0.779 | 0.006 | 8.57E-10 | 3.23E-05 | 36.00 |
| Type 2 diabetes | rs35352848 | C | T | -0.062 | 0.207 | 0.006 | 7.37E-28 | 9.58E-05 | 106.78 |
| Type 2 diabetes | rs35895680 | C | A | 0.056 | 0.686 | 0.005 | 1.42E-28 | 1.13E-04 | 125.44 |
| Type 2 diabetes | rs36051838 | C | T | 0.044 | 0.089 | 0.008 | 4.54E-08 | 2.71E-05 | 30.25 |
| Type 2 diabetes | rs36111056 | G | A | 0.034 | 0.78 | 0.006 | 1.97E-09 | 2.88E-05 | 32.11 |
| Type 2 diabetes | rs3747207 | G | A | -0.047 | 0.779 | 0.006 | 9.26E-18 | 5.51E-05 | 61.36 |
| Type 2 diabetes | rs3757969 | G | C | 0.048 | 0.376 | 0.005 | 3.60E-22 | 8.27E-05 | 92.16 |
| Type 2 diabetes | rs3764002 | C | T | 0.04 | 0.741 | 0.005 | 2.69E-14 | 5.74E-05 | 64.00 |
| Type 2 diabetes | rs3798519 | C | A | 0.05 | 0.18 | 0.006 | 5.49E-17 | 6.23E-05 | 69.44 |
| Type 2 diabetes | rs3872707 | G | A | -0.045 | 0.86 | 0.007 | 3.46E-11 | 3.71E-05 | 41.33 |
| Type 2 diabetes | rs41276588 | G | A | -0.038 | 0.717 | 0.005 | 4.10E-13 | 5.18E-05 | 57.76 |
| Type 2 diabetes | rs4132228 | C | T | 0.047 | 0.703 | 0.005 | 6.22E-21 | 7.93E-05 | 88.36 |
| Type 2 diabetes | rs429358 | C | T | -0.073 | 0.145 | 0.007 | 3.98E-28 | 9.76E-05 | 108.75 |
| Type 2 diabetes | rs4688760 | C | T | -0.034 | 0.326 | 0.005 | 1.10E-11 | 4.15E-05 | 46.24 |
| Type 2 diabetes | rs4709746 | C | T | 0.058 | 0.869 | 0.007 | 1.03E-16 | 6.16E-05 | 68.65 |
| Type 2 diabetes | rs4721089 | C | T | -0.034 | 0.243 | 0.006 | 7.92E-10 | 2.88E-05 | 32.11 |
| Type 2 diabetes | rs4796224 | G | A | 0.025 | 0.474 | 0.005 | 2.71E-08 | 2.24E-05 | 25.00 |
| Type 2 diabetes | rs4805681 | C | T | 0.027 | 0.61 | 0.005 | 1.32E-08 | 2.62E-05 | 29.16 |
| Type 2 diabetes | rs4809369 | G | A | 0.034 | 0.555 | 0.005 | 4.27E-13 | 4.15E-05 | 46.24 |
| Type 2 diabetes | rs4845987 | G | C | -0.028 | 0.309 | 0.005 | 2.78E-08 | 2.81E-05 | 31.36 |
| Type 2 diabetes | rs4865796 | G | A | -0.047 | 0.318 | 0.005 | 4.64E-21 | 7.93E-05 | 88.36 |
| Type 2 diabetes | rs4899280 | C | T | -0.028 | 0.67 | 0.005 | 1.02E-08 | 2.81E-05 | 31.36 |
| Type 2 diabetes | rs4929965 | G | A | -0.062 | 0.615 | 0.005 | 2.46E-38 | 1.38E-04 | 153.76 |
| Type 2 diabetes | rs4984980 | G | A | -0.035 | 0.812 | 0.006 | 3.79E-09 | 3.05E-05 | 34.03 |
| Type 2 diabetes | rs5219 | C | T | -0.069 | 0.627 | 0.005 | 3.15E-48 | 1.71E-04 | 190.44 |
| Type 2 diabetes | rs545608 | G | C | -0.036 | 0.802 | 0.006 | 6.50E-10 | 3.23E-05 | 36.00 |
| Type 2 diabetes | rs55857387 | C | T | -0.052 | 0.195 | 0.006 | 6.71E-19 | 6.74E-05 | 75.11 |
| Type 2 diabetes | rs56348580 | G | C | 0.058 | 0.696 | 0.005 | 2.33E-30 | 1.21E-04 | 134.56 |
| Type 2 diabetes | rs56394279 | C | T | 0.031 | 0.468 | 0.005 | 5.30E-12 | 3.45E-05 | 38.44 |
| Type 2 diabetes | rs567185 | C | T | -0.037 | 0.366 | 0.005 | 1.55E-14 | 4.91E-05 | 54.76 |
| Type 2 diabetes | rs5753043 | C | A | 0.061 | 0.898 | 0.008 | 3.76E-14 | 5.22E-05 | 58.14 |
| Type 2 diabetes | rs576674 | G | A | 0.061 | 0.185 | 0.006 | 1.25E-23 | 9.27E-05 | 103.36 |
| Type 2 diabetes | rs58542926 | C | T | -0.089 | 0.925 | 0.009 | 4.67E-25 | 8.77E-05 | 97.79 |
| Type 2 diabetes | rs60519666 | G | A | 0.035 | 0.677 | 0.005 | 3.19E-12 | 4.40E-05 | 49.00 |
| Type 2 diabetes | rs6066138 | G | A | 0.045 | 0.725 | 0.005 | 1.58E-18 | 7.27E-05 | 81.00 |
| Type 2 diabetes | rs61779275 | C | T | -0.075 | 0.78 | 0.006 | 3.35E-43 | 1.40E-04 | 156.25 |
| Type 2 diabetes | rs62075585 | G | A | 0.03 | 0.474 | 0.005 | 1.25E-10 | 3.23E-05 | 36.00 |
| Type 2 diabetes | rs62515938 | C | T | -0.029 | 0.74 | 0.005 | 2.97E-08 | 3.02E-05 | 33.64 |
| Type 2 diabetes | rs62618693 | C | T | 0.085 | 0.957 | 0.011 | 6.83E-14 | 5.36E-05 | 59.71 |
| Type 2 diabetes | rs6459733 | G | C | 0.051 | 0.666 | 0.005 | 9.66E-25 | 9.33E-05 | 104.04 |
| Type 2 diabetes | rs6741676 | G | A | -0.032 | 0.332 | 0.005 | 3.64E-11 | 3.68E-05 | 40.96 |
| Type 2 diabetes | rs67755137 | G | A | -0.033 | 0.81 | 0.006 | 2.24E-08 | 2.71E-05 | 30.25 |
| Type 2 diabetes | rs6777684 | G | A | 0.057 | 0.608 | 0.005 | 3.77E-33 | 1.17E-04 | 129.96 |
| Type 2 diabetes | rs6819331 | C | T | 0.04 | 0.68 | 0.005 | 2.21E-16 | 5.74E-05 | 64.00 |
| Type 2 diabetes | rs6821438 | G | A | -0.029 | 0.469 | 0.005 | 3.66E-10 | 3.02E-05 | 33.64 |
| Type 2 diabetes | rs6878122 | G | A | 0.055 | 0.311 | 0.005 | 4.60E-29 | 1.09E-04 | 121.00 |
| Type 2 diabetes | rs6976111 | C | A | -0.032 | 0.693 | 0.005 | 7.93E-10 | 3.68E-05 | 40.96 |
| Type 2 diabetes | rs7026688 | G | A | 0.044 | 0.863 | 0.007 | 3.40E-11 | 3.55E-05 | 39.51 |
| Type 2 diabetes | rs703981 | G | C | 0.061 | 0.544 | 0.005 | 2.13E-40 | 1.34E-04 | 148.84 |
| Type 2 diabetes | rs7099048 | G | A | -0.028 | 0.496 | 0.005 | 8.93E-10 | 2.81E-05 | 31.36 |
| Type 2 diabetes | rs7163757 | C | T | 0.041 | 0.574 | 0.005 | 1.64E-18 | 6.03E-05 | 67.24 |
| Type 2 diabetes | rs7240767 | C | T | 0.037 | 0.379 | 0.006 | 5.03E-09 | 3.41E-05 | 38.03 |
| Type 2 diabetes | rs72695645 | G | A | 0.061 | 0.86 | 0.007 | 8.66E-20 | 6.81E-05 | 75.94 |
| Type 2 diabetes | rs72802342 | C | A | 0.115 | 0.921 | 0.009 | 5.58E-38 | 1.46E-04 | 163.27 |
| Type 2 diabetes | rs72926932 | C | A | 0.075 | 0.078 | 0.008 | 7.58E-20 | 7.89E-05 | 87.89 |
| Type 2 diabetes | rs72951506 | C | T | 0.042 | 0.852 | 0.007 | 9.22E-11 | 3.23E-05 | 36.00 |
| Type 2 diabetes | rs73872717 | C | T | 0.086 | 0.953 | 0.011 | 4.78E-15 | 5.48E-05 | 61.12 |
| Type 2 diabetes | rs74804697 | G | C | -0.084 | 0.043 | 0.012 | 4.68E-12 | 4.40E-05 | 49.00 |
| Type 2 diabetes | rs75432112 | G | A | -0.134 | 0.952 | 0.011 | 5.34E-36 | 1.33E-04 | 148.40 |
| Type 2 diabetes | rs756145 | G | A | -0.029 | 0.691 | 0.005 | 5.52E-09 | 3.02E-05 | 33.64 |
| Type 2 diabetes | rs7561798 | G | A | 0.028 | 0.485 | 0.005 | 1.16E-09 | 2.81E-05 | 31.36 |
| Type 2 diabetes | rs7568172 | G | A | 0.067 | 0.94 | 0.01 | 2.64E-12 | 4.03E-05 | 44.89 |
| Type 2 diabetes | rs75693095 | G | C | -0.112 | 0.979 | 0.017 | 1.42E-11 | 3.89E-05 | 43.40 |
| Type 2 diabetes | rs75756987 | G | C | 0.044 | 0.888 | 0.007 | 1.61E-09 | 3.55E-05 | 39.51 |
| Type 2 diabetes | rs7609422 | G | A | 0.03 | 0.41 | 0.005 | 2.67E-10 | 3.23E-05 | 36.00 |
| Type 2 diabetes | rs7660000 | C | T | 0.031 | 0.713 | 0.005 | 1.29E-09 | 3.45E-05 | 38.44 |
| Type 2 diabetes | rs7695096 | C | T | 0.038 | 0.521 | 0.005 | 1.27E-16 | 5.18E-05 | 57.76 |
| Type 2 diabetes | rs7756992 | G | A | 0.122 | 0.271 | 0.005 | 5.52E-128 | 5.34E-04 | 595.36 |
| Type 2 diabetes | rs7858727 | C | A | 0.034 | 0.223 | 0.006 | 8.33E-10 | 2.88E-05 | 32.11 |
| Type 2 diabetes | rs7903146 | C | T | -0.28 | 0.708 | 0.005 | 1.00E-200 | 2.81E-03 | 3135.99 |
| Type 2 diabetes | rs8008540 | C | T | 0.03 | 0.569 | 0.005 | 7.99E-11 | 3.23E-05 | 36.00 |
| Type 2 diabetes | rs8010382 | G | A | 0.032 | 0.437 | 0.005 | 5.74E-12 | 3.68E-05 | 40.96 |
| Type 2 diabetes | rs8018512 | G | A | 0.037 | 0.742 | 0.005 | 1.30E-12 | 4.91E-05 | 54.76 |
| Type 2 diabetes | rs8031576 | C | A | 0.057 | 0.289 | 0.005 | 1.74E-29 | 1.17E-04 | 129.96 |
| Type 2 diabetes | rs8054556 | G | A | -0.036 | 0.538 | 0.005 | 3.68E-15 | 4.65E-05 | 51.84 |
| Type 2 diabetes | rs8071043 | C | T | 0.054 | 0.328 | 0.005 | 5.16E-28 | 1.05E-04 | 116.64 |
| Type 2 diabetes | rs827237 | C | T | -0.037 | 0.795 | 0.006 | 3.56E-10 | 3.41E-05 | 38.03 |
| Type 2 diabetes | rs844215 | C | T | 0.026 | 0.587 | 0.005 | 2.04E-08 | 2.43E-05 | 27.04 |
| Type 2 diabetes | rs878017 | G | A | -0.035 | 0.459 | 0.006 | 1.13E-08 | 3.05E-05 | 34.03 |
| Type 2 diabetes | rs890940 | C | T | -0.048 | 0.789 | 0.006 | 9.78E-18 | 5.74E-05 | 64.00 |
| Type 2 diabetes | rs911300 | G | A | 0.035 | 0.542 | 0.005 | 5.19E-14 | 4.40E-05 | 49.00 |
| Type 2 diabetes | rs9275184 | C | T | 0.098 | 0.111 | 0.01 | 5.59E-24 | 8.62E-05 | 96.04 |
| Type 2 diabetes | rs9316500 | G | T | -0.047 | 0.292 | 0.005 | 9.97E-21 | 7.93E-05 | 88.36 |
| Type 2 diabetes | rs9319382 | C | T | 0.028 | 0.679 | 0.005 | 2.87E-08 | 2.81E-05 | 31.36 |
| Type 2 diabetes | rs9368112 | C | T | -0.026 | 0.475 | 0.005 | 2.69E-08 | 2.43E-05 | 27.04 |
| Type 2 diabetes | rs9379084 | G | A | 0.075 | 0.88 | 0.008 | 3.70E-22 | 7.89E-05 | 87.89 |
| Type 2 diabetes | rs9383649 | G | A | 0.033 | 0.419 | 0.005 | 1.06E-12 | 3.91E-05 | 43.56 |
| Type 2 diabetes | rs9555581 | C | T | 0.03 | 0.61 | 0.005 | 3.72E-10 | 3.23E-05 | 36.00 |
| Type 2 diabetes | rs9563574 | C | T | -0.041 | 0.177 | 0.006 | 1.64E-11 | 4.19E-05 | 46.69 |
| Type 2 diabetes | rs9784137 | G | A | 0.061 | 0.849 | 0.007 | 5.25E-21 | 6.81E-05 | 75.94 |
| Type 2 diabetes | rs980183 | G | A | 0.036 | 0.394 | 0.005 | 1.02E-14 | 4.65E-05 | 51.84 |
| Type 2 diabetes | rs9852406 | C | T | -0.038 | 0.746 | 0.005 | 8.96E-13 | 5.18E-05 | 57.76 |
| Type 2 diabetes | rs9854769 | G | A | 0.108 | 0.319 | 0.005 | 1.06E-107 | 4.18E-04 | 466.56 |
| Type 2 diabetes | rs9873519 | C | T | -0.038 | 0.466 | 0.005 | 3.58E-16 | 5.18E-05 | 57.76 |
| Type 2 diabetes | rs9873618 | G | A | 0.058 | 0.709 | 0.005 | 2.41E-30 | 1.21E-04 | 134.56 |
| Type 2 diabetes | rs9927842 | C | T | -0.038 | 0.838 | 0.007 | 6.84E-09 | 2.64E-05 | 29.47 |
| Systolic blood pressure | rs1000423 | T | C | 0.4138 | 0.732 | 6.50E-33 | 3.46E-02 | 1.42E-04 | 143.00 |
| Systolic blood pressure | rs10008637 | C | T | -0.2157 | 0.46 | 9.24E-13 | 3.02E-02 | 5.07E-05 | 51.00 |
| Systolic blood pressure | rs10028284 | T | A | -0.2937 | 0.182 | 1.69E-13 | 3.98E-02 | 5.41E-05 | 54.50 |
| Systolic blood pressure | rs10048404 | T | C | -0.2607 | 0.37 | 1.91E-16 | 3.17E-02 | 6.72E-05 | 67.60 |
| Systolic blood pressure | rs1006545 | T | G | 0.6846 | 0.887 | 3.50E-46 | 4.80E-02 | 2.02E-04 | 203.00 |
| Systolic blood pressure | rs10069690 | T | C | 0.3098 | 0.258 | 4.47E-17 | 3.69E-02 | 7.00E-05 | 70.50 |
| Systolic blood pressure | rs1010064 | C | A | -0.3571 | 0.184 | 3.02E-20 | 3.87E-02 | 8.46E-05 | 85.10 |
| Systolic blood pressure | rs1012089 | G | C | 0.192 | 0.525 | 1.95E-10 | 3.02E-02 | 4.01E-05 | 40.40 |
| Systolic blood pressure | rs10188003 | T | C | 0.1883 | 0.393 | 8.80E-10 | 3.07E-02 | 3.74E-05 | 37.60 |
| Systolic blood pressure | rs10207726 | T | C | -0.2142 | 0.296 | 8.06E-11 | 3.30E-02 | 4.18E-05 | 42.10 |
| Systolic blood pressure | rs10224210 | C | T | 0.3831 | 0.279 | 1.60E-29 | 3.40E-02 | 1.26E-04 | 127.00 |
| Systolic blood pressure | rs10282122 | T | C | -0.302 | 0.668 | 2.46E-20 | 3.27E-02 | 8.47E-05 | 85.30 |
| Systolic blood pressure | rs10420519 | T | G | -0.4921 | 0.035 | 2.86E-08 | 8.87E-02 | 3.06E-05 | 30.80 |
| Systolic blood pressure | rs1044822 | T | C | -0.248 | 0.148 | 5.16E-09 | 4.24E-02 | 3.40E-05 | 34.20 |
| Systolic blood pressure | rs10460108 | G | A | -0.2141 | 0.52 | 1.12E-12 | 3.01E-02 | 5.02E-05 | 50.60 |
| Systolic blood pressure | rs1049212 | G | A | 0.299 | 0.569 | 4.59E-23 | 3.02E-02 | 9.73E-05 | 98.00 |
| Systolic blood pressure | rs10501410 | A | G | 0.4122 | 0.069 | 1.10E-11 | 6.07E-02 | 4.58E-05 | 46.10 |
| Systolic blood pressure | rs1052501 | T | C | 0.2262 | 0.833 | 4.14E-08 | 4.12E-02 | 2.99E-05 | 30.10 |
| Systolic blood pressure | rs10749572 | T | G | -0.203 | 0.544 | 1.88E-11 | 3.02E-02 | 4.49E-05 | 45.20 |
| Systolic blood pressure | rs10750441 | T | C | 0.1754 | 0.662 | 3.74E-08 | 3.19E-02 | 3.00E-05 | 30.20 |
| Systolic blood pressure | rs10776752 | T | G | 0.8211 | 0.081 | 4.61E-46 | 5.76E-02 | 2.02E-04 | 203.00 |
| Systolic blood pressure | rs10777213 | A | G | -0.1786 | 0.524 | 2.45E-09 | 2.99E-02 | 3.54E-05 | 35.70 |
| Systolic blood pressure | rs10779795 | G | A | -0.2191 | 0.339 | 7.44E-12 | 3.20E-02 | 4.66E-05 | 46.90 |
| Systolic blood pressure | rs10782230 | A | G | 0.2106 | 0.485 | 2.91E-12 | 3.02E-02 | 4.83E-05 | 48.60 |
| Systolic blood pressure | rs10804330 | C | T | -0.2351 | 0.433 | 1.62E-14 | 3.06E-02 | 5.86E-05 | 59.00 |
| Systolic blood pressure | rs10914124 | C | T | -0.234 | 0.383 | 6.32E-14 | 3.12E-02 | 5.59E-05 | 56.20 |
| Systolic blood pressure | rs10941043 | G | T | 0.2585 | 0.29 | 6.42E-15 | 3.32E-02 | 6.02E-05 | 60.60 |
| Systolic blood pressure | rs10980408 | C | T | 0.7606 | 0.036 | 3.83E-20 | 8.27E-02 | 8.40E-05 | 84.60 |
| Systolic blood pressure | rs11097909 | C | T | 0.3628 | 0.853 | 3.35E-17 | 4.30E-02 | 7.07E-05 | 71.20 |
| Systolic blood pressure | rs11120093 | T | C | -0.1792 | 0.408 | 5.13E-09 | 3.07E-02 | 3.38E-05 | 34.10 |
| Systolic blood pressure | rs11145807 | G | A | -0.2135 | 0.594 | 3.54E-11 | 3.22E-02 | 4.37E-05 | 44.00 |
| Systolic blood pressure | rs11159091 | A | G | 0.1978 | 0.462 | 6.79E-11 | 3.03E-02 | 4.23E-05 | 42.60 |
| Systolic blood pressure | rs111866816 | T | C | 0.3569 | 0.071 | 2.29E-09 | 5.97E-02 | 3.55E-05 | 35.70 |
| Systolic blood pressure | rs11191580 | C | T | -1.0995 | 0.082 | 7.74E-89 | 5.50E-02 | 3.97E-04 | 400.00 |
| Systolic blood pressure | rs11210029 | G | A | 0.203 | 0.368 | 8.92E-11 | 3.13E-02 | 4.18E-05 | 42.10 |
| Systolic blood pressure | rs11222084 | T | A | 0.3363 | 0.362 | 1.80E-26 | 3.16E-02 | 1.12E-04 | 113.00 |
| Systolic blood pressure | rs11241313 | T | C | -0.2071 | 0.311 | 2.23E-10 | 3.26E-02 | 4.01E-05 | 40.40 |
| Systolic blood pressure | rs112509803 | C | G | -0.2641 | 0.114 | 3.18E-08 | 4.77E-02 | 3.04E-05 | 30.70 |
| Systolic blood pressure | rs11252324 | T | G | -0.4164 | 0.077 | 3.61E-13 | 5.73E-02 | 5.24E-05 | 52.80 |
| Systolic blood pressure | rs112854918 | G | C | 0.5577 | 0.026 | 2.77E-08 | 1.00E-01 | 3.06E-05 | 30.90 |
| Systolic blood pressure | rs113086489 | T | C | 0.3249 | 0.553 | 3.80E-26 | 3.07E-02 | 1.11E-04 | 112.00 |
| Systolic blood pressure | rs1133400 | G | A | 0.2975 | 0.214 | 2.53E-15 | 3.76E-02 | 6.22E-05 | 62.60 |
| Systolic blood pressure | rs113695818 | T | C | -0.1835 | 0.303 | 2.62E-08 | 3.30E-02 | 3.07E-05 | 30.90 |
| Systolic blood pressure | rs115262049 | T | A | -0.5893 | 0.087 | 1.29E-26 | 5.52E-02 | 1.13E-04 | 114.00 |
| Systolic blood pressure | rs1154214 | G | T | 0.2031 | 0.604 | 3.27E-11 | 3.06E-02 | 4.38E-05 | 44.10 |
| Systolic blood pressure | rs11585169 | A | T | 0.1796 | 0.577 | 5.34E-09 | 3.08E-02 | 3.38E-05 | 34.00 |
| Systolic blood pressure | rs11592107 | A | G | 0.3024 | 0.31 | 1.55E-20 | 3.26E-02 | 8.55E-05 | 86.00 |
| Systolic blood pressure | rs11604357 | A | C | -0.277 | 0.162 | 1.60E-11 | 4.11E-02 | 4.51E-05 | 45.40 |
| Systolic blood pressure | rs11636952 | C | T | -0.5313 | 0.686 | 4.22E-59 | 3.28E-02 | 2.61E-04 | 262.00 |
| Systolic blood pressure | rs11641374 | A | C | -0.1943 | 0.6 | 3.26E-10 | 3.09E-02 | 3.93E-05 | 39.50 |
| Systolic blood pressure | rs11655604 | T | C | -0.2033 | 0.358 | 1.09E-09 | 3.33E-02 | 3.70E-05 | 37.30 |
| Systolic blood pressure | rs11672660 | T | C | 0.2212 | 0.2 | 6.32E-09 | 3.81E-02 | 3.35E-05 | 33.70 |
| Systolic blood pressure | rs11694601 | G | A | 0.1909 | 0.403 | 6.41E-10 | 3.09E-02 | 3.79E-05 | 38.20 |
| Systolic blood pressure | rs117206641 | T | C | 0.3154 | 0.111 | 2.66E-10 | 4.99E-02 | 3.97E-05 | 40.00 |
| Systolic blood pressure | rs117464403 | A | G | 0.864 | 0.018 | 5.80E-13 | 1.20E-01 | 5.16E-05 | 51.90 |
| Systolic blood pressure | rs11874246 | T | C | 0.2856 | 0.296 | 3.22E-18 | 3.28E-02 | 7.53E-05 | 75.80 |
| Systolic blood pressure | rs11925504 | A | G | -0.2901 | 0.572 | 1.78E-21 | 3.05E-02 | 8.98E-05 | 90.50 |
| Systolic blood pressure | rs11960210 | C | T | -0.4727 | 0.376 | 1.25E-51 | 3.13E-02 | 2.26E-04 | 228.00 |
| Systolic blood pressure | rs11977526 | A | G | -0.3213 | 0.401 | 6.62E-25 | 3.12E-02 | 1.05E-04 | 106.00 |
| Systolic blood pressure | rs1199330 | G | A | 0.2654 | 0.118 | 1.65E-08 | 4.70E-02 | 3.17E-05 | 31.90 |
| Systolic blood pressure | rs12042924 | C | T | 0.1807 | 0.472 | 2.62E-09 | 3.03E-02 | 3.53E-05 | 35.60 |
| Systolic blood pressure | rs12063372 | A | G | 0.1989 | 0.385 | 3.86E-10 | 3.18E-02 | 3.89E-05 | 39.10 |
| Systolic blood pressure | rs1209384 | G | A | -0.2558 | 0.612 | 2.85E-16 | 3.13E-02 | 6.63E-05 | 66.80 |
| Systolic blood pressure | rs12136922 | A | G | 0.2027 | 0.495 | 2.69E-11 | 3.04E-02 | 4.42E-05 | 44.50 |
| Systolic blood pressure | rs12153395 | A | G | -0.3303 | 0.115 | 1.07E-11 | 4.86E-02 | 4.59E-05 | 46.20 |
| Systolic blood pressure | rs12255372 | T | G | 0.2358 | 0.288 | 1.94E-12 | 3.35E-02 | 4.92E-05 | 49.50 |
| Systolic blood pressure | rs12258967 | G | C | -0.6327 | 0.295 | 1.08E-78 | 3.37E-02 | 3.50E-04 | 352.00 |
| Systolic blood pressure | rs12264186 | T | C | 0.2135 | 0.187 | 3.58E-08 | 3.87E-02 | 3.02E-05 | 30.40 |
| Systolic blood pressure | rs12321 | C | G | -0.2292 | 0.433 | 3.81E-14 | 3.03E-02 | 5.68E-05 | 57.20 |
| Systolic blood pressure | rs12426261 | G | A | -0.3775 | 0.621 | 2.31E-34 | 3.09E-02 | 1.48E-04 | 149.00 |
| Systolic blood pressure | rs12464602 | A | G | -0.2437 | 0.621 | 1.02E-14 | 3.15E-02 | 5.94E-05 | 59.90 |
| Systolic blood pressure | rs12509595 | C | T | 0.8367 | 0.292 | 2.55E-138 | 3.34E-02 | 6.23E-04 | 628.00 |
| Systolic blood pressure | rs12511987 | G | T | 0.2329 | 0.177 | 5.39E-09 | 3.99E-02 | 3.38E-05 | 34.10 |
| Systolic blood pressure | rs12528975 | A | G | 0.4751 | 0.049 | 1.98E-10 | 7.47E-02 | 4.02E-05 | 40.50 |
| Systolic blood pressure | rs12596630 | T | C | 0.4278 | 0.09 | 5.01E-15 | 5.47E-02 | 6.07E-05 | 61.20 |
| Systolic blood pressure | rs12610654 | G | A | -0.2315 | 0.344 | 4.41E-13 | 3.20E-02 | 5.20E-05 | 52.30 |
| Systolic blood pressure | rs12627651 | A | G | 0.3498 | 0.287 | 1.02E-24 | 3.41E-02 | 1.04E-04 | 105.00 |
| Systolic blood pressure | rs12637573 | G | A | 0.1731 | 0.528 | 9.95E-09 | 3.02E-02 | 3.26E-05 | 32.90 |
| Systolic blood pressure | rs12643599 | G | A | -0.3134 | 0.361 | 1.23E-23 | 3.13E-02 | 9.96E-05 | 100.00 |
| Systolic blood pressure | rs12656497 | C | T | 0.6382 | 0.597 | 7.14E-96 | 3.07E-02 | 4.29E-04 | 432.00 |
| Systolic blood pressure | rs12657950 | T | C | 0.455 | 0.074 | 1.27E-14 | 5.90E-02 | 5.91E-05 | 59.50 |
| Systolic blood pressure | rs12661036 | C | T | 0.2104 | 0.225 | 1.82E-08 | 3.74E-02 | 3.14E-05 | 31.60 |
| Systolic blood pressure | rs12668436 | C | T | 0.2151 | 0.246 | 7.88E-10 | 3.50E-02 | 3.75E-05 | 37.80 |
| Systolic blood pressure | rs12693982 | T | C | 0.2575 | 0.402 | 7.49E-17 | 3.09E-02 | 6.90E-05 | 69.40 |
| Systolic blood pressure | rs12694277 | C | T | 0.2018 | 0.705 | 1.80E-09 | 3.35E-02 | 3.60E-05 | 36.30 |
| Systolic blood pressure | rs12731646 | T | C | -0.189 | 0.409 | 7.21E-10 | 3.07E-02 | 3.76E-05 | 37.90 |
| Systolic blood pressure | rs1275985 | T | C | -0.5411 | 0.613 | 4.73E-69 | 3.08E-02 | 3.06E-04 | 309.00 |
| Systolic blood pressure | rs12883810 | T | C | -0.2382 | 0.146 | 2.70E-08 | 4.28E-02 | 3.08E-05 | 31.00 |
| Systolic blood pressure | rs12885878 | G | A | 0.2291 | 0.766 | 4.32E-10 | 3.67E-02 | 3.87E-05 | 39.00 |
| Systolic blood pressure | rs12906962 | C | T | 0.2653 | 0.324 | 3.28E-16 | 3.25E-02 | 6.62E-05 | 66.60 |
| Systolic blood pressure | rs1290784 | T | C | 0.4124 | 0.448 | 2.97E-42 | 3.03E-02 | 1.84E-04 | 185.00 |
| Systolic blood pressure | rs1290933 | A | C | -0.2847 | 0.692 | 3.17E-18 | 3.27E-02 | 7.53E-05 | 75.80 |
| Systolic blood pressure | rs12926550 | A | G | -0.2548 | 0.316 | 3.43E-15 | 3.24E-02 | 6.14E-05 | 61.80 |
| Systolic blood pressure | rs1293969 | C | T | 0.1988 | 0.252 | 1.03E-08 | 3.47E-02 | 3.26E-05 | 32.80 |
| Systolic blood pressure | rs12978472 | G | C | -0.845 | 0.124 | 1.23E-66 | 4.90E-02 | 2.95E-04 | 297.00 |
| Systolic blood pressure | rs13016772 | T | C | 0.2522 | 0.765 | 1.23E-12 | 3.55E-02 | 5.01E-05 | 50.50 |
| Systolic blood pressure | rs13091418 | G | C | 0.2234 | 0.334 | 6.15E-12 | 3.25E-02 | 4.69E-05 | 47.20 |
| Systolic blood pressure | rs13107261 | A | G | -0.1778 | 0.369 | 1.57E-08 | 3.14E-02 | 3.18E-05 | 32.10 |
| Systolic blood pressure | rs13107325 | T | C | -0.9086 | 0.074 | 4.22E-53 | 5.92E-02 | 2.34E-04 | 236.00 |
| Systolic blood pressure | rs13204703 | C | T | -0.1967 | 0.249 | 1.94E-08 | 3.50E-02 | 3.14E-05 | 31.60 |
| Systolic blood pressure | rs13253358 | T | C | 0.2127 | 0.298 | 1.13E-10 | 3.30E-02 | 4.13E-05 | 41.50 |
| Systolic blood pressure | rs1332813 | C | T | -0.2203 | 0.649 | 2.32E-12 | 3.14E-02 | 4.89E-05 | 49.20 |
| Systolic blood pressure | rs13358657 | G | A | 0.388 | 0.133 | 2.95E-18 | 4.45E-02 | 7.55E-05 | 76.00 |
| Systolic blood pressure | rs1340030 | C | T | -0.1936 | 0.366 | 5.77E-10 | 3.12E-02 | 3.82E-05 | 38.50 |
| Systolic blood pressure | rs13412750 | A | G | -0.2889 | 0.271 | 2.32E-17 | 3.41E-02 | 7.13E-05 | 71.80 |
| Systolic blood pressure | rs13420463 | G | A | -0.3143 | 0.227 | 2.72E-18 | 3.60E-02 | 7.57E-05 | 76.20 |
| Systolic blood pressure | rs1375564 | T | C | 0.2579 | 0.64 | 2.84E-16 | 3.15E-02 | 6.66E-05 | 67.00 |
| Systolic blood pressure | rs1382472 | A | G | -0.1917 | 0.404 | 4.47E-10 | 3.07E-02 | 3.87E-05 | 39.00 |
| Systolic blood pressure | rs139354822 | C | T | -0.6115 | 0.03 | 3.51E-10 | 9.75E-02 | 3.91E-05 | 39.30 |
| Systolic blood pressure | rs1408945 | T | G | -0.3196 | 0.424 | 8.33E-26 | 3.04E-02 | 1.10E-04 | 111.00 |
| Systolic blood pressure | rs1410222 | T | C | 0.2173 | 0.817 | 2.17E-08 | 3.88E-02 | 3.12E-05 | 31.40 |
| Systolic blood pressure | rs141958336 | A | G | 0.7807 | 0.043 | 1.36E-23 | 7.80E-02 | 9.95E-05 | 100.00 |
| Systolic blood pressure | rs1422279 | T | C | 0.331 | 0.386 | 1.05E-26 | 3.09E-02 | 1.14E-04 | 115.00 |
| Systolic blood pressure | rs1433121 | T | C | -0.228 | 0.691 | 2.66E-12 | 3.26E-02 | 4.86E-05 | 48.90 |
| Systolic blood pressure | rs1436138 | G | A | -0.3119 | 0.363 | 4.73E-23 | 3.15E-02 | 9.74E-05 | 98.00 |
| Systolic blood pressure | rs1437649 | A | G | -0.2189 | 0.235 | 8.57E-10 | 3.57E-02 | 3.73E-05 | 37.60 |
| Systolic blood pressure | rs146550789 | C | T | 0.4824 | 0.042 | 5.64E-10 | 7.78E-02 | 3.82E-05 | 38.40 |
| Systolic blood pressure | rs148140538 | T | C | -0.3252 | 0.081 | 7.39E-09 | 5.62E-02 | 3.33E-05 | 33.50 |
| Systolic blood pressure | rs148401029 | A | C | -0.4623 | 0.035 | 4.97E-08 | 8.48E-02 | 2.95E-05 | 29.70 |
| Systolic blood pressure | rs1493132 | C | T | 0.1766 | 0.34 | 2.73E-08 | 3.18E-02 | 3.06E-05 | 30.80 |
| Systolic blood pressure | rs1544861 | C | T | -0.1969 | 0.661 | 5.66E-10 | 3.18E-02 | 3.81E-05 | 38.30 |
| Systolic blood pressure | rs1551355 | T | C | 0.2098 | 0.233 | 3.89E-09 | 3.56E-02 | 3.45E-05 | 34.70 |
| Systolic blood pressure | rs1565440 | A | G | 0.1746 | 0.375 | 1.94E-08 | 3.11E-02 | 3.13E-05 | 31.50 |
| Systolic blood pressure | rs1575290 | T | C | 0.1973 | 0.473 | 5.59E-11 | 3.01E-02 | 4.27E-05 | 43.00 |
| Systolic blood pressure | rs1623474 | T | C | 0.3827 | 0.33 | 7.66E-33 | 3.21E-02 | 1.41E-04 | 142.00 |
| Systolic blood pressure | rs1630736 | T | C | -0.1706 | 0.465 | 3.52E-08 | 3.09E-02 | 3.03E-05 | 30.50 |
| Systolic blood pressure | rs1664781 | A | G | 0.2643 | 0.693 | 5.69E-16 | 3.26E-02 | 6.53E-05 | 65.70 |
| Systolic blood pressure | rs17010957 | C | T | 0.534 | 0.146 | 1.78E-35 | 4.30E-02 | 1.53E-04 | 154.00 |
| Systolic blood pressure | rs17035181 | G | T | -0.3074 | 0.145 | 7.61E-13 | 4.29E-02 | 5.10E-05 | 51.30 |
| Systolic blood pressure | rs17080102 | C | G | -0.8085 | 0.069 | 3.52E-42 | 5.94E-02 | 1.84E-04 | 185.00 |
| Systolic blood pressure | rs17245822 | C | A | 0.1899 | 0.373 | 1.15E-09 | 3.12E-02 | 3.68E-05 | 37.00 |
| Systolic blood pressure | rs17249754 | A | G | -0.8446 | 0.168 | 1.25E-97 | 4.03E-02 | 4.36E-04 | 439.00 |
| Systolic blood pressure | rs17257081 | G | A | -0.2274 | 0.194 | 6.35E-09 | 3.92E-02 | 3.34E-05 | 33.70 |
| Systolic blood pressure | rs17562391 | T | C | 0.1967 | 0.419 | 1.35E-10 | 3.06E-02 | 4.10E-05 | 41.30 |
| Systolic blood pressure | rs17608766 | C | T | 0.6903 | 0.145 | 2.48E-57 | 4.33E-02 | 2.52E-04 | 254.00 |
| Systolic blood pressure | rs177551 | A | C | 0.373 | 0.134 | 3.47E-17 | 4.42E-02 | 7.07E-05 | 71.20 |
| Systolic blood pressure | rs17760259 | C | T | 0.2654 | 0.428 | 2.25E-18 | 3.04E-02 | 7.57E-05 | 76.20 |
| Systolic blood pressure | rs17762 | A | G | 0.4117 | 0.078 | 5.60E-13 | 5.71E-02 | 5.16E-05 | 52.00 |
| Systolic blood pressure | rs17807723 | A | G | -0.2721 | 0.138 | 8.43E-10 | 4.43E-02 | 3.75E-05 | 37.70 |
| Systolic blood pressure | rs17812022 | T | C | -0.3613 | 0.096 | 5.65E-12 | 5.25E-02 | 4.70E-05 | 47.40 |
| Systolic blood pressure | rs1786345 | C | A | -0.2073 | 0.434 | 1.34E-11 | 3.07E-02 | 4.53E-05 | 45.60 |
| Systolic blood pressure | rs1814951 | A | G | -0.3231 | 0.879 | 3.91E-12 | 4.66E-02 | 4.77E-05 | 48.10 |
| Systolic blood pressure | rs1821002 | G | C | -0.3794 | 0.589 | 5.19E-35 | 3.07E-02 | 1.52E-04 | 153.00 |
| Systolic blood pressure | rs1848994 | A | G | 0.2012 | 0.283 | 1.79E-09 | 3.34E-02 | 3.60E-05 | 36.30 |
| Systolic blood pressure | rs1870735 | G | C | -0.206 | 0.547 | 3.60E-11 | 3.11E-02 | 4.36E-05 | 43.90 |
| Systolic blood pressure | rs1871190 | T | G | 0.1954 | 0.335 | 1.66E-09 | 3.24E-02 | 3.61E-05 | 36.40 |
| Systolic blood pressure | rs1882212 | G | A | -0.2753 | 0.221 | 3.34E-14 | 3.63E-02 | 5.71E-05 | 57.50 |
| Systolic blood pressure | rs1882961 | T | C | 0.2443 | 0.309 | 6.69E-14 | 3.26E-02 | 5.58E-05 | 56.20 |
| Systolic blood pressure | rs1889785 | A | G | 0.1782 | 0.455 | 4.35E-09 | 3.04E-02 | 3.41E-05 | 34.40 |
| Systolic blood pressure | rs189267552 | A | T | -0.8664 | 0.013 | 4.55E-10 | 1.39E-01 | 3.86E-05 | 38.90 |
| Systolic blood pressure | rs1896326 | A | G | -0.2797 | 0.229 | 4.40E-14 | 3.71E-02 | 5.64E-05 | 56.80 |
| Systolic blood pressure | rs1906672 | A | G | 0.2966 | 0.232 | 1.20E-16 | 3.58E-02 | 6.82E-05 | 68.60 |
| Systolic blood pressure | rs1957563 | T | C | 0.3629 | 0.265 | 2.32E-26 | 3.42E-02 | 1.12E-04 | 113.00 |
| Systolic blood pressure | rs1994158 | G | A | -0.2513 | 0.181 | 1.23E-10 | 3.91E-02 | 4.10E-05 | 41.30 |
| Systolic blood pressure | rs2014408 | T | C | 0.5169 | 0.209 | 1.26E-43 | 3.73E-02 | 1.91E-04 | 192.00 |
| Systolic blood pressure | rs2024385 | A | T | -0.2642 | 0.424 | 5.88E-18 | 3.06E-02 | 7.40E-05 | 74.50 |
| Systolic blood pressure | rs2111557 | T | C | 0.1764 | 0.468 | 5.22E-09 | 3.02E-02 | 3.39E-05 | 34.10 |
| Systolic blood pressure | rs2126474 | T | G | -0.2601 | 0.413 | 1.87E-17 | 3.06E-02 | 7.18E-05 | 72.20 |
| Systolic blood pressure | rs2129869 | T | A | 0.2643 | 0.222 | 2.44E-13 | 3.61E-02 | 5.32E-05 | 53.60 |
| Systolic blood pressure | rs2161967 | G | T | -0.2836 | 0.572 | 2.87E-20 | 3.07E-02 | 8.47E-05 | 85.30 |
| Systolic blood pressure | rs2177843 | T | C | 0.4394 | 0.151 | 2.80E-24 | 4.32E-02 | 1.03E-04 | 103.00 |
| Systolic blood pressure | rs2236295 | T | G | -0.3028 | 0.398 | 1.04E-22 | 3.09E-02 | 9.54E-05 | 96.00 |
| Systolic blood pressure | rs2238787 | A | G | 0.2552 | 0.292 | 1.45E-14 | 3.32E-02 | 5.87E-05 | 59.10 |
| Systolic blood pressure | rs2249105 | G | A | -0.2927 | 0.368 | 7.63E-21 | 3.13E-02 | 8.68E-05 | 87.40 |
| Systolic blood pressure | rs2276153 | G | C | -0.3296 | 0.243 | 6.56E-21 | 3.51E-02 | 8.76E-05 | 88.20 |
| Systolic blood pressure | rs2283500 | C | A | -0.3106 | 0.11 | 1.08E-10 | 4.81E-02 | 4.14E-05 | 41.70 |
| Systolic blood pressure | rs2289124 | A | G | -0.308 | 0.167 | 1.14E-13 | 4.15E-02 | 5.47E-05 | 55.10 |
| Systolic blood pressure | rs2291434 | T | G | -0.2622 | 0.534 | 5.10E-18 | 3.03E-02 | 7.44E-05 | 74.90 |
| Systolic blood pressure | rs2291516 | A | G | 0.3708 | 0.103 | 2.17E-13 | 5.05E-02 | 5.35E-05 | 53.90 |
| Systolic blood pressure | rs2353940 | C | T | 0.2075 | 0.249 | 6.85E-09 | 3.58E-02 | 3.34E-05 | 33.60 |
| Systolic blood pressure | rs2354862 | C | A | -0.2507 | 0.359 | 2.42E-15 | 3.17E-02 | 6.21E-05 | 62.50 |
| Systolic blood pressure | rs2384063 | T | C | 0.3266 | 0.761 | 6.33E-20 | 3.57E-02 | 8.31E-05 | 83.70 |
| Systolic blood pressure | rs2392929 | G | T | 0.7507 | 0.203 | 1.96E-87 | 3.79E-02 | 3.90E-04 | 392.00 |
| Systolic blood pressure | rs2423514 | G | A | -0.3011 | 0.459 | 1.77E-23 | 3.02E-02 | 9.87E-05 | 99.40 |
| Systolic blood pressure | rs246973 | T | C | 0.2479 | 0.288 | 1.45E-13 | 3.35E-02 | 5.44E-05 | 54.80 |
| Systolic blood pressure | rs2470004 | T | C | -0.3454 | 0.818 | 1.28E-18 | 3.92E-02 | 7.71E-05 | 77.60 |
| Systolic blood pressure | rs2493134 | C | T | 0.3736 | 0.407 | 1.10E-33 | 3.09E-02 | 1.45E-04 | 146.00 |
| Systolic blood pressure | rs2493296 | T | C | 0.4183 | 0.143 | 3.14E-21 | 4.42E-02 | 8.89E-05 | 89.60 |
| Systolic blood pressure | rs2498323 | A | G | 0.3171 | 0.098 | 8.51E-10 | 5.17E-02 | 3.74E-05 | 37.60 |
| Systolic blood pressure | rs2580350 | A | G | 0.1769 | 0.561 | 8.39E-09 | 3.07E-02 | 3.30E-05 | 33.20 |
| Systolic blood pressure | rs2589218 | C | T | 0.2258 | 0.27 | 2.54E-11 | 3.39E-02 | 4.41E-05 | 44.40 |
| Systolic blood pressure | rs2598 | G | A | -0.168 | 0.467 | 2.87E-08 | 3.03E-02 | 3.05E-05 | 30.70 |
| Systolic blood pressure | rs2608029 | G | C | -0.1805 | 0.335 | 1.61E-08 | 3.20E-02 | 3.16E-05 | 31.80 |
| Systolic blood pressure | rs2610990 | G | A | 0.2903 | 0.736 | 2.86E-17 | 3.43E-02 | 7.11E-05 | 71.60 |
| Systolic blood pressure | rs2627313 | T | C | 0.3208 | 0.445 | 3.55E-26 | 3.03E-02 | 1.11E-04 | 112.00 |
| Systolic blood pressure | rs263532 | C | T | -0.1798 | 0.425 | 4.72E-09 | 3.07E-02 | 3.41E-05 | 34.30 |
| Systolic blood pressure | rs2643826 | T | C | 0.4473 | 0.451 | 1.74E-48 | 3.06E-02 | 2.12E-04 | 214.00 |
| Systolic blood pressure | rs2652812 | T | C | -0.2516 | 0.754 | 1.03E-12 | 3.53E-02 | 5.05E-05 | 50.80 |
| Systolic blood pressure | rs268263 | A | T | 0.5938 | 0.75 | 1.77E-63 | 3.53E-02 | 2.81E-04 | 283.00 |
| Systolic blood pressure | rs2689690 | T | C | -0.2702 | 0.368 | 1.15E-17 | 3.16E-02 | 7.26E-05 | 73.10 |
| Systolic blood pressure | rs2724377 | G | A | -0.1938 | 0.47 | 1.29E-10 | 3.01E-02 | 4.12E-05 | 41.50 |
| Systolic blood pressure | rs2744139 | C | T | -0.2369 | 0.238 | 2.48E-11 | 3.55E-02 | 4.42E-05 | 44.50 |
| Systolic blood pressure | rs2745599 | G | A | -0.2164 | 0.448 | 8.96E-12 | 3.17E-02 | 4.63E-05 | 46.60 |
| Systolic blood pressure | rs2760748 | A | T | 0.3626 | 0.098 | 1.05E-12 | 5.09E-02 | 5.04E-05 | 50.70 |
| Systolic blood pressure | rs2776037 | C | T | 0.1851 | 0.585 | 2.15E-09 | 3.09E-02 | 3.56E-05 | 35.90 |
| Systolic blood pressure | rs2801008 | G | T | 0.1876 | 0.318 | 7.37E-09 | 3.24E-02 | 3.33E-05 | 33.50 |
| Systolic blood pressure | rs2815063 | A | C | 0.2755 | 0.132 | 1.76E-09 | 4.58E-02 | 3.59E-05 | 36.20 |
| Systolic blood pressure | rs2833834 | A | C | 0.2177 | 0.277 | 1.22E-10 | 3.38E-02 | 4.12E-05 | 41.50 |
| Systolic blood pressure | rs28429256 | A | G | 0.215 | 0.334 | 3.89E-11 | 3.25E-02 | 4.35E-05 | 43.80 |
| Systolic blood pressure | rs2853736 | G | T | -0.2368 | 0.306 | 8.34E-13 | 3.31E-02 | 5.08E-05 | 51.20 |
| Systolic blood pressure | rs28572357 | C | A | 0.2733 | 0.398 | 6.34E-19 | 3.08E-02 | 7.82E-05 | 78.70 |
| Systolic blood pressure | rs28578714 | C | T | -0.2066 | 0.394 | 2.53E-10 | 3.27E-02 | 3.96E-05 | 39.90 |
| Systolic blood pressure | rs28650790 | T | C | 0.2287 | 0.189 | 3.30E-09 | 3.87E-02 | 3.47E-05 | 34.90 |
| Systolic blood pressure | rs28688791 | C | T | 0.3222 | 0.198 | 2.34E-17 | 3.80E-02 | 7.14E-05 | 71.90 |
| Systolic blood pressure | rs28866311 | G | T | 0.2762 | 0.474 | 5.45E-20 | 3.02E-02 | 8.31E-05 | 83.60 |
| Systolic blood pressure | rs2904315 | G | A | 0.2081 | 0.687 | 1.58E-10 | 3.25E-02 | 4.07E-05 | 41.00 |
| Systolic blood pressure | rs2913920 | T | C | 0.2418 | 0.765 | 1.62E-11 | 3.59E-02 | 4.51E-05 | 45.40 |
| Systolic blood pressure | rs2957688 | A | G | 0.3472 | 0.471 | 2.74E-30 | 3.04E-02 | 1.30E-04 | 130.00 |
| Systolic blood pressure | rs3098186 | T | C | -0.2422 | 0.516 | 1.41E-15 | 3.03E-02 | 6.35E-05 | 63.90 |
| Systolic blood pressure | rs3104552 | C | T | -0.2449 | 0.439 | 6.31E-16 | 3.03E-02 | 6.49E-05 | 65.30 |
| Systolic blood pressure | rs34025993 | G | A | -0.223 | 0.586 | 4.71E-13 | 3.08E-02 | 5.21E-05 | 52.40 |
| Systolic blood pressure | rs34072724 | A | G | -0.2422 | 0.489 | 1.37E-15 | 3.03E-02 | 6.35E-05 | 63.90 |
| Systolic blood pressure | rs34079867 | T | C | 0.1992 | 0.266 | 1.78E-08 | 3.54E-02 | 3.14E-05 | 31.70 |
| Systolic blood pressure | rs34130368 | T | G | -0.3016 | 0.117 | 1.28E-09 | 4.97E-02 | 3.66E-05 | 36.80 |
| Systolic blood pressure | rs34413141 | A | T | -0.3531 | 0.182 | 2.47E-19 | 3.93E-02 | 8.02E-05 | 80.70 |
| Systolic blood pressure | rs34518929 | A | G | -0.22 | 0.262 | 1.79E-10 | 3.45E-02 | 4.04E-05 | 40.70 |
| Systolic blood pressure | rs34535756 | T | C | 0.478 | 0.039 | 1.18E-09 | 7.86E-02 | 3.67E-05 | 37.00 |
| Systolic blood pressure | rs34727427 | C | T | 0.2353 | 0.317 | 4.02E-13 | 3.24E-02 | 5.24E-05 | 52.70 |
| Systolic blood pressure | rs34917849 | C | G | 0.3124 | 0.127 | 5.97E-12 | 4.54E-02 | 4.70E-05 | 47.30 |
| Systolic blood pressure | rs34941092 | A | G | -0.3225 | 0.15 | 3.23E-14 | 4.25E-02 | 5.72E-05 | 57.60 |
| Systolic blood pressure | rs35098810 | C | A | -0.1967 | 0.232 | 3.20E-08 | 3.56E-02 | 3.03E-05 | 30.50 |
| Systolic blood pressure | rs35413927 | G | A | 0.3002 | 0.305 | 5.25E-20 | 3.28E-02 | 8.32E-05 | 83.80 |
| Systolic blood pressure | rs35444 | G | A | -0.4368 | 0.386 | 3.47E-45 | 3.10E-02 | 1.97E-04 | 199.00 |
| Systolic blood pressure | rs35680304 | T | C | 0.2694 | 0.593 | 3.76E-18 | 3.10E-02 | 7.50E-05 | 75.50 |
| Systolic blood pressure | rs35783704 | A | G | -0.4619 | 0.104 | 8.81E-20 | 5.07E-02 | 8.24E-05 | 83.00 |
| Systolic blood pressure | rs3735533 | C | T | 0.91 | 0.926 | 5.29E-56 | 5.77E-02 | 2.47E-04 | 249.00 |
| Systolic blood pressure | rs3764400 | C | T | -0.3748 | 0.137 | 3.69E-17 | 4.45E-02 | 7.04E-05 | 70.90 |
| Systolic blood pressure | rs3772219 | C | A | -0.2733 | 0.318 | 3.10E-17 | 3.24E-02 | 7.07E-05 | 71.20 |
| Systolic blood pressure | rs3802517 | A | T | 0.2527 | 0.462 | 4.65E-17 | 3.01E-02 | 7.00E-05 | 70.50 |
| Systolic blood pressure | rs3807925 | G | A | 0.1859 | 0.35 | 5.39E-09 | 3.19E-02 | 3.37E-05 | 34.00 |
| Systolic blood pressure | rs3815460 | G | C | 0.285 | 0.102 | 1.21E-08 | 5.00E-02 | 3.23E-05 | 32.50 |
| Systolic blood pressure | rs3819532 | C | T | 0.1875 | 0.609 | 9.44E-10 | 3.06E-02 | 3.73E-05 | 37.50 |
| Systolic blood pressure | rs3828282 | G | C | -0.1857 | 0.572 | 5.29E-09 | 3.18E-02 | 3.39E-05 | 34.10 |
| Systolic blood pressure | rs3845811 | G | C | 0.2942 | 0.434 | 1.88E-21 | 3.09E-02 | 9.00E-05 | 90.60 |
| Systolic blood pressure | rs3860770 | A | G | -0.2663 | 0.292 | 1.20E-15 | 3.33E-02 | 6.35E-05 | 64.00 |
| Systolic blood pressure | rs3918226 | T | C | 0.664 | 0.081 | 8.46E-31 | 5.75E-02 | 1.32E-04 | 133.00 |
| Systolic blood pressure | rs3950627 | A | C | 0.1851 | 0.531 | 1.82E-09 | 3.08E-02 | 3.59E-05 | 36.10 |
| Systolic blood pressure | rs3980686 | T | G | -0.4998 | 0.108 | 1.03E-24 | 4.87E-02 | 1.05E-04 | 105.00 |
| Systolic blood pressure | rs404100 | T | C | 0.1935 | 0.451 | 1.68E-10 | 3.03E-02 | 4.05E-05 | 40.80 |
| Systolic blood pressure | rs4143175 | C | T | -0.2187 | 0.759 | 5.10E-10 | 3.52E-02 | 3.83E-05 | 38.60 |
| Systolic blood pressure | rs42032 | A | G | -0.3231 | 0.264 | 7.39E-21 | 3.45E-02 | 8.71E-05 | 87.70 |
| Systolic blood pressure | rs4245599 | G | A | 0.1794 | 0.542 | 4.03E-09 | 3.05E-02 | 3.44E-05 | 34.60 |
| Systolic blood pressure | rs4260863 | G | C | -0.1911 | 0.384 | 1.17E-09 | 3.14E-02 | 3.68E-05 | 37.00 |
| Systolic blood pressure | rs4274337 | G | A | 0.2968 | 0.83 | 2.48E-13 | 4.06E-02 | 5.31E-05 | 53.40 |
| Systolic blood pressure | rs4284362 | A | C | -0.2256 | 0.718 | 2.61E-11 | 3.38E-02 | 4.42E-05 | 44.50 |
| Systolic blood pressure | rs4408839 | G | A | 0.2301 | 0.257 | 2.42E-11 | 3.45E-02 | 4.42E-05 | 44.50 |
| Systolic blood pressure | rs4440615 | A | G | -0.2201 | 0.632 | 1.87E-12 | 3.12E-02 | 4.94E-05 | 49.80 |
| Systolic blood pressure | rs4499560 | T | A | 0.2199 | 0.683 | 1.46E-11 | 3.26E-02 | 4.52E-05 | 45.50 |
| Systolic blood pressure | rs4511593 | T | C | -0.2881 | 0.653 | 1.28E-19 | 3.18E-02 | 8.15E-05 | 82.10 |
| Systolic blood pressure | rs4553000 | T | C | -0.2035 | 0.514 | 1.09E-11 | 3.00E-02 | 4.57E-05 | 46.00 |
| Systolic blood pressure | rs4577304 | C | T | 0.1767 | 0.477 | 4.99E-09 | 3.02E-02 | 3.40E-05 | 34.20 |
| Systolic blood pressure | rs4606697 | A | G | -0.3196 | 0.104 | 9.71E-10 | 5.23E-02 | 3.71E-05 | 37.30 |
| Systolic blood pressure | rs4651224 | T | C | 0.1986 | 0.447 | 9.00E-11 | 3.06E-02 | 4.18E-05 | 42.10 |
| Systolic blood pressure | rs4734868 | G | A | 0.1844 | 0.33 | 9.22E-09 | 3.21E-02 | 3.28E-05 | 33.00 |
| Systolic blood pressure | rs4775769 | G | T | 0.4162 | 0.906 | 7.76E-16 | 5.17E-02 | 6.44E-05 | 64.80 |
| Systolic blood pressure | rs4784541 | C | T | 0.2015 | 0.525 | 4.93E-11 | 3.07E-02 | 4.28E-05 | 43.10 |
| Systolic blood pressure | rs483071 | T | C | 0.2709 | 0.625 | 5.09E-18 | 3.13E-02 | 7.44E-05 | 74.90 |
| Systolic blood pressure | rs4834792 | A | T | 0.1973 | 0.48 | 7.24E-11 | 3.03E-02 | 4.21E-05 | 42.40 |
| Systolic blood pressure | rs4838021 | T | C | -0.3009 | 0.129 | 3.13E-11 | 4.53E-02 | 4.38E-05 | 44.10 |
| Systolic blood pressure | rs4873492 | T | C | 0.3431 | 0.172 | 1.61E-17 | 4.03E-02 | 7.20E-05 | 72.50 |
| Systolic blood pressure | rs4876133 | C | G | 0.22 | 0.711 | 6.23E-11 | 3.36E-02 | 4.26E-05 | 42.90 |
| Systolic blood pressure | rs488834 | T | C | -0.3799 | 0.765 | 2.35E-25 | 3.65E-02 | 1.08E-04 | 108.00 |
| Systolic blood pressure | rs4888408 | A | G | 0.3653 | 0.586 | 1.42E-32 | 3.07E-02 | 1.41E-04 | 142.00 |
| Systolic blood pressure | rs4925159 | A | G | 0.2174 | 0.425 | 9.66E-13 | 3.05E-02 | 5.05E-05 | 50.80 |
| Systolic blood pressure | rs4926499 | C | G | 0.2965 | 0.826 | 1.33E-11 | 4.38E-02 | 4.55E-05 | 45.80 |
| Systolic blood pressure | rs4932373 | C | A | 0.635 | 0.326 | 2.49E-83 | 3.28E-02 | 3.72E-04 | 375.00 |
| Systolic blood pressure | rs4952609 | G | A | -0.2124 | 0.256 | 9.60E-10 | 3.47E-02 | 3.72E-05 | 37.50 |
| Systolic blood pressure | rs4955575 | C | A | -0.2158 | 0.254 | 5.63E-10 | 3.48E-02 | 3.82E-05 | 38.50 |
| Systolic blood pressure | rs4957026 | G | A | -0.1982 | 0.66 | 8.12E-10 | 3.23E-02 | 3.74E-05 | 37.70 |
| Systolic blood pressure | rs4961293 | T | C | 0.2268 | 0.451 | 7.35E-14 | 3.03E-02 | 5.56E-05 | 56.00 |
| Systolic blood pressure | rs4980379 | T | C | 0.5764 | 0.372 | 2.47E-72 | 3.20E-02 | 3.22E-04 | 324.00 |
| Systolic blood pressure | rs509833 | G | A | -0.329 | 0.861 | 7.08E-14 | 4.40E-02 | 5.55E-05 | 55.90 |
| Systolic blood pressure | rs55732192 | T | G | -0.3358 | 0.095 | 1.15E-10 | 5.21E-02 | 4.13E-05 | 41.50 |
| Systolic blood pressure | rs55924432 | T | C | 0.2651 | 0.401 | 5.70E-17 | 3.17E-02 | 6.95E-05 | 69.90 |
| Systolic blood pressure | rs55944332 | G | A | 0.2613 | 0.237 | 1.79E-13 | 3.55E-02 | 5.38E-05 | 54.20 |
| Systolic blood pressure | rs56288724 | G | A | 0.2178 | 0.417 | 2.01E-12 | 3.10E-02 | 4.90E-05 | 49.40 |
| Systolic blood pressure | rs56407827 | T | C | 0.3603 | 0.269 | 2.78E-26 | 3.40E-02 | 1.12E-04 | 112.00 |
| Systolic blood pressure | rs571689 | T | C | 0.228 | 0.52 | 6.77E-14 | 3.04E-02 | 5.59E-05 | 56.20 |
| Systolic blood pressure | rs573455 | G | A | -0.1994 | 0.539 | 4.77E-11 | 3.03E-02 | 4.30E-05 | 43.30 |
| Systolic blood pressure | rs5742643 | C | T | 0.2233 | 0.751 | 1.53E-10 | 3.49E-02 | 4.07E-05 | 40.90 |
| Systolic blood pressure | rs57786342 | A | G | 0.2317 | 0.206 | 5.63E-10 | 3.74E-02 | 3.81E-05 | 38.40 |
| Systolic blood pressure | rs57946343 | C | T | -0.716 | 0.147 | 2.10E-63 | 4.26E-02 | 2.80E-04 | 282.00 |
| Systolic blood pressure | rs59980837 | T | G | 1.0997 | 0.018 | 3.32E-21 | 1.16E-01 | 8.88E-05 | 89.40 |
| Systolic blood pressure | rs60138042 | G | C | -0.3395 | 0.065 | 4.14E-08 | 6.19E-02 | 2.99E-05 | 30.10 |
| Systolic blood pressure | rs60191654 | G | A | 0.2382 | 0.188 | 5.88E-10 | 3.85E-02 | 3.80E-05 | 38.30 |
| Systolic blood pressure | rs6026578 | G | C | 0.185 | 0.627 | 4.59E-09 | 3.16E-02 | 3.40E-05 | 34.30 |
| Systolic blood pressure | rs6026744 | T | A | 0.7131 | 0.123 | 7.00E-54 | 4.61E-02 | 2.38E-04 | 239.00 |
| Systolic blood pressure | rs6029756 | A | G | -0.2712 | 0.323 | 1.88E-16 | 3.30E-02 | 6.71E-05 | 67.50 |
| Systolic blood pressure | rs6031431 | G | A | 0.2617 | 0.462 | 7.05E-18 | 3.04E-02 | 7.36E-05 | 74.10 |
| Systolic blood pressure | rs60444686 | A | G | 0.5905 | 0.041 | 4.42E-14 | 7.82E-02 | 5.66E-05 | 57.00 |
| Systolic blood pressure | rs604723 | C | T | 0.655 | 0.724 | 2.55E-83 | 3.39E-02 | 3.71E-04 | 373.00 |
| Systolic blood pressure | rs6054139 | A | G | 0.2094 | 0.606 | 8.23E-12 | 3.06E-02 | 4.65E-05 | 46.80 |
| Systolic blood pressure | rs6058088 | G | T | -0.2832 | 0.156 | 1.14E-11 | 4.17E-02 | 4.58E-05 | 46.10 |
| Systolic blood pressure | rs6062324 | A | G | -0.3294 | 0.236 | 1.18E-19 | 3.63E-02 | 8.18E-05 | 82.30 |
| Systolic blood pressure | rs6078093 | A | G | -0.1849 | 0.428 | 1.20E-09 | 3.04E-02 | 3.67E-05 | 37.00 |
| Systolic blood pressure | rs6090907 | A | G | -0.3854 | 0.147 | 1.29E-19 | 4.25E-02 | 8.17E-05 | 82.20 |
| Systolic blood pressure | rs60909079 | C | G | -0.2114 | 0.249 | 1.73E-09 | 3.51E-02 | 3.60E-05 | 36.30 |
| Systolic blood pressure | rs60991988 | G | T | -0.3789 | 0.107 | 2.82E-14 | 4.98E-02 | 5.75E-05 | 57.90 |
| Systolic blood pressure | rs6108787 | G | T | 0.4274 | 0.47 | 5.38E-46 | 3.00E-02 | 2.02E-04 | 203.00 |
| Systolic blood pressure | rs61772592 | G | A | 0.3181 | 0.126 | 2.86E-12 | 4.55E-02 | 4.85E-05 | 48.90 |
| Systolic blood pressure | rs61917655 | T | C | 0.3427 | 0.101 | 2.68E-11 | 5.14E-02 | 4.41E-05 | 44.50 |
| Systolic blood pressure | rs62047964 | T | C | 0.5115 | 0.062 | 9.29E-14 | 6.86E-02 | 5.52E-05 | 55.60 |
| Systolic blood pressure | rs62076622 | G | A | -0.2363 | 0.199 | 3.79E-10 | 3.77E-02 | 3.90E-05 | 39.30 |
| Systolic blood pressure | rs62082230 | A | T | -0.1884 | 0.277 | 4.69E-08 | 3.45E-02 | 2.96E-05 | 29.80 |
| Systolic blood pressure | rs62170470 | C | T | -0.1972 | 0.398 | 7.68E-10 | 3.21E-02 | 3.75E-05 | 37.70 |
| Systolic blood pressure | rs62309747 | A | G | -0.2244 | 0.473 | 1.59E-13 | 3.04E-02 | 5.41E-05 | 54.50 |
| Systolic blood pressure | rs62512914 | G | A | -0.2066 | 0.415 | 1.42E-11 | 3.06E-02 | 4.53E-05 | 45.60 |
| Systolic blood pressure | rs6271 | T | C | -0.5547 | 0.074 | 1.18E-19 | 6.11E-02 | 8.19E-05 | 82.40 |
| Systolic blood pressure | rs641620 | C | T | 0.3193 | 0.145 | 3.74E-13 | 4.40E-02 | 5.23E-05 | 52.70 |
| Systolic blood pressure | rs6438857 | C | T | -0.2736 | 0.423 | 3.13E-19 | 3.05E-02 | 7.99E-05 | 80.50 |
| Systolic blood pressure | rs6445583 | A | G | 0.2774 | 0.747 | 1.90E-15 | 3.49E-02 | 6.27E-05 | 63.20 |
| Systolic blood pressure | rs6452769 | A | G | -0.3143 | 0.205 | 7.82E-17 | 3.77E-02 | 6.90E-05 | 69.50 |
| Systolic blood pressure | rs6490019 | G | A | 0.2897 | 0.62 | 6.61E-21 | 3.09E-02 | 8.73E-05 | 87.90 |
| Systolic blood pressure | rs6504213 | C | T | 0.2982 | 0.582 | 1.25E-21 | 3.12E-02 | 9.07E-05 | 91.30 |
| Systolic blood pressure | rs6540119 | T | A | -0.2016 | 0.666 | 3.93E-10 | 3.22E-02 | 3.89E-05 | 39.20 |
| Systolic blood pressure | rs6562778 | G | A | -0.178 | 0.541 | 4.95E-09 | 3.04E-02 | 3.40E-05 | 34.30 |
| Systolic blood pressure | rs658780 | G | T | 0.2028 | 0.255 | 5.29E-09 | 3.47E-02 | 3.39E-05 | 34.20 |
| Systolic blood pressure | rs665445 | A | C | -0.1909 | 0.279 | 1.15E-08 | 3.34E-02 | 3.24E-05 | 32.70 |
| Systolic blood pressure | rs66864335 | A | G | -0.3963 | 0.221 | 1.79E-27 | 3.65E-02 | 1.17E-04 | 118.00 |
| Systolic blood pressure | rs6699618 | G | C | -0.9115 | 0.16 | 1.68E-109 | 4.10E-02 | 4.91E-04 | 494.00 |
| Systolic blood pressure | rs6731373 | A | G | 0.1913 | 0.349 | 4.18E-09 | 3.26E-02 | 3.42E-05 | 34.40 |
| Systolic blood pressure | rs6732123 | C | G | -0.1737 | 0.417 | 1.52E-08 | 3.07E-02 | 3.18E-05 | 32.00 |
| Systolic blood pressure | rs6737318 | G | A | -0.2348 | 0.222 | 1.13E-10 | 3.64E-02 | 4.13E-05 | 41.60 |
| Systolic blood pressure | rs67617547 | G | C | -0.1799 | 0.33 | 2.39E-08 | 3.22E-02 | 3.10E-05 | 31.20 |
| Systolic blood pressure | rs6771917 | C | T | 0.3793 | 0.752 | 1.39E-26 | 3.55E-02 | 1.13E-04 | 114.00 |
| Systolic blood pressure | rs6788907 | A | G | 0.221 | 0.268 | 7.31E-11 | 3.39E-02 | 4.22E-05 | 42.50 |
| Systolic blood pressure | rs6788984 | G | A | -0.2999 | 0.144 | 3.81E-12 | 4.32E-02 | 4.79E-05 | 48.20 |
| Systolic blood pressure | rs68085857 | T | C | 0.274 | 0.234 | 1.68E-14 | 3.57E-02 | 5.85E-05 | 58.90 |
| Systolic blood pressure | rs68115553 | G | A | 0.6445 | 0.02 | 1.74E-08 | 1.14E-01 | 3.16E-05 | 31.80 |
| Systolic blood pressure | rs6870654 | C | T | -0.2136 | 0.255 | 7.58E-10 | 3.47E-02 | 3.76E-05 | 37.90 |
| Systolic blood pressure | rs6892983 | A | C | 0.3427 | 0.402 | 7.11E-29 | 3.07E-02 | 1.24E-04 | 125.00 |
| Systolic blood pressure | rs6921291 | T | C | 0.3575 | 0.191 | 1.58E-20 | 3.85E-02 | 8.56E-05 | 86.20 |
| Systolic blood pressure | rs6957161 | G | A | -0.2064 | 0.738 | 2.20E-09 | 3.45E-02 | 3.55E-05 | 35.80 |
| Systolic blood pressure | rs6961048 | G | C | 0.5304 | 0.104 | 1.43E-26 | 4.97E-02 | 1.13E-04 | 114.00 |
| Systolic blood pressure | rs6986368 | T | A | 0.2132 | 0.327 | 9.62E-11 | 3.29E-02 | 4.17E-05 | 42.00 |
| Systolic blood pressure | rs7012866 | G | T | 0.2325 | 0.501 | 1.21E-14 | 3.01E-02 | 5.93E-05 | 59.70 |
| Systolic blood pressure | rs702395 | T | C | 0.2318 | 0.437 | 3.24E-14 | 3.05E-02 | 5.74E-05 | 57.80 |
| Systolic blood pressure | rs7026176 | T | G | -0.1869 | 0.512 | 4.01E-10 | 2.99E-02 | 3.88E-05 | 39.10 |
| Systolic blood pressure | rs7045409 | A | T | -0.1862 | 0.367 | 2.55E-09 | 3.13E-02 | 3.51E-05 | 35.40 |
| Systolic blood pressure | rs708117 | A | G | 0.2874 | 0.52 | 1.59E-21 | 3.02E-02 | 8.99E-05 | 90.60 |
| Systolic blood pressure | rs7093894 | A | C | 0.236 | 0.151 | 3.16E-08 | 4.27E-02 | 3.03E-05 | 30.50 |
| Systolic blood pressure | rs7107356 | G | A | 0.4598 | 0.504 | 1.63E-52 | 3.01E-02 | 2.32E-04 | 233.00 |
| Systolic blood pressure | rs7134440 | T | C | 0.4788 | 0.082 | 1.58E-17 | 5.62E-02 | 7.21E-05 | 72.60 |
| Systolic blood pressure | rs7134677 | T | C | -0.3851 | 0.298 | 4.46E-31 | 3.32E-02 | 1.34E-04 | 135.00 |
| Systolic blood pressure | rs7154723 | A | G | 0.253 | 0.385 | 2.72E-16 | 3.09E-02 | 6.66E-05 | 67.00 |
| Systolic blood pressure | rs7186298 | T | C | -0.2315 | 0.43 | 1.88E-14 | 3.02E-02 | 5.84E-05 | 58.80 |
| Systolic blood pressure | rs7198817 | A | C | -0.1824 | 0.632 | 5.99E-09 | 3.13E-02 | 3.37E-05 | 34.00 |
| Systolic blood pressure | rs7211535 | G | A | 0.1779 | 0.524 | 4.61E-09 | 3.04E-02 | 3.40E-05 | 34.20 |
| Systolic blood pressure | rs7213273 | A | G | -0.4 | 0.655 | 6.24E-37 | 3.15E-02 | 1.60E-04 | 161.00 |
| Systolic blood pressure | rs7236548 | A | C | 0.3431 | 0.185 | 8.51E-19 | 3.88E-02 | 7.77E-05 | 78.20 |
| Systolic blood pressure | rs7255933 | A | G | 0.2306 | 0.257 | 2.44E-11 | 3.45E-02 | 4.44E-05 | 44.70 |
| Systolic blood pressure | rs72683923 | C | T | -0.9587 | 0.021 | 3.08E-18 | 1.10E-01 | 7.53E-05 | 75.80 |
| Systolic blood pressure | rs72719160 | T | A | 0.2243 | 0.317 | 4.34E-12 | 3.24E-02 | 4.76E-05 | 47.90 |
| Systolic blood pressure | rs72742507 | T | C | -0.2053 | 0.3 | 3.80E-10 | 3.28E-02 | 3.89E-05 | 39.20 |
| Systolic blood pressure | rs72931748 | G | A | -0.3967 | 0.099 | 6.40E-14 | 5.29E-02 | 5.58E-05 | 56.20 |
| Systolic blood pressure | rs73046792 | A | G | -0.3554 | 0.159 | 7.23E-17 | 4.26E-02 | 6.91E-05 | 69.60 |
| Systolic blood pressure | rs73049928 | G | A | 0.2382 | 0.194 | 1.20E-09 | 3.92E-02 | 3.67E-05 | 36.90 |
| Systolic blood pressure | rs7306710 | C | T | 0.2429 | 0.519 | 1.02E-15 | 3.03E-02 | 6.38E-05 | 64.30 |
| Systolic blood pressure | rs73075659 | G | A | -0.3962 | 0.335 | 5.52E-35 | 3.21E-02 | 1.51E-04 | 152.00 |
| Systolic blood pressure | rs73103937 | C | T | -0.2051 | 0.268 | 2.36E-09 | 3.44E-02 | 3.53E-05 | 35.50 |
| Systolic blood pressure | rs7310615 | G | C | -0.585 | 0.518 | 1.32E-81 | 3.06E-02 | 3.63E-04 | 365.00 |
| Systolic blood pressure | rs7331680 | T | G | 0.4101 | 0.149 | 3.35E-22 | 4.23E-02 | 9.33E-05 | 94.00 |
| Systolic blood pressure | rs73727605 | A | G | 0.3616 | 0.066 | 6.60E-09 | 6.23E-02 | 3.35E-05 | 33.70 |
| Systolic blood pressure | rs73855810 | A | G | 0.2732 | 0.141 | 3.04E-10 | 4.34E-02 | 3.94E-05 | 39.60 |
| Systolic blood pressure | rs7395791 | A | G | -0.2162 | 0.442 | 2.19E-12 | 3.08E-02 | 4.89E-05 | 49.30 |
| Systolic blood pressure | rs74048190 | C | T | 0.4404 | 0.048 | 6.07E-09 | 7.57E-02 | 3.36E-05 | 33.80 |
| Systolic blood pressure | rs740746 | A | G | 0.4557 | 0.732 | 1.42E-40 | 3.42E-02 | 1.76E-04 | 178.00 |
| Systolic blood pressure | rs743395 | T | C | 0.2597 | 0.383 | 2.55E-16 | 3.17E-02 | 6.67E-05 | 67.10 |
| Systolic blood pressure | rs7439567 | T | C | 0.2537 | 0.411 | 2.31E-16 | 3.09E-02 | 6.69E-05 | 67.40 |
| Systolic blood pressure | rs74538877 | C | G | -0.3855 | 0.056 | 4.97E-08 | 7.07E-02 | 2.95E-05 | 29.70 |
| Systolic blood pressure | rs7463212 | A | T | -0.2753 | 0.545 | 1.81E-19 | 3.05E-02 | 8.09E-05 | 81.50 |
| Systolic blood pressure | rs7491248 | A | G | 0.2163 | 0.224 | 2.37E-09 | 3.62E-02 | 3.55E-05 | 35.70 |
| Systolic blood pressure | rs7493678 | T | A | 0.189 | 0.349 | 2.31E-09 | 3.16E-02 | 3.55E-05 | 35.80 |
| Systolic blood pressure | rs75016974 | T | C | -0.2513 | 0.142 | 1.05E-08 | 4.39E-02 | 3.25E-05 | 32.80 |
| Systolic blood pressure | rs7514579 | C | A | -0.2243 | 0.229 | 5.45E-10 | 3.61E-02 | 3.83E-05 | 38.60 |
| Systolic blood pressure | rs75461554 | T | C | -0.3016 | 0.201 | 1.18E-15 | 3.77E-02 | 6.36E-05 | 64.00 |
| Systolic blood pressure | rs7555285 | C | G | 0.2294 | 0.801 | 1.05E-09 | 3.76E-02 | 3.70E-05 | 37.20 |
| Systolic blood pressure | rs75672964 | T | C | 0.5885 | 0.042 | 2.35E-12 | 8.39E-02 | 4.89E-05 | 49.20 |
| Systolic blood pressure | rs75961402 | A | G | 0.2659 | 0.153 | 1.95E-10 | 4.18E-02 | 4.02E-05 | 40.50 |
| Systolic blood pressure | rs7615099 | G | A | -0.1891 | 0.333 | 3.90E-09 | 3.21E-02 | 3.45E-05 | 34.70 |
| Systolic blood pressure | rs7618284 | C | G | -0.1891 | 0.339 | 1.10E-08 | 3.31E-02 | 3.24E-05 | 32.60 |
| Systolic blood pressure | rs76443575 | C | G | -0.5233 | 0.036 | 1.40E-10 | 8.16E-02 | 4.08E-05 | 41.10 |
| Systolic blood pressure | rs76452347 | T | C | -0.2974 | 0.205 | 7.13E-14 | 3.97E-02 | 5.57E-05 | 56.10 |
| Systolic blood pressure | rs76719272 | T | C | -0.2738 | 0.131 | 2.97E-09 | 4.61E-02 | 3.50E-05 | 35.30 |
| Systolic blood pressure | rs7683728 | T | C | -0.3654 | 0.531 | 2.43E-33 | 3.04E-02 | 1.43E-04 | 144.00 |
| Systolic blood pressure | rs7703560 | G | A | 0.2246 | 0.3 | 1.51E-11 | 3.33E-02 | 4.52E-05 | 45.50 |
| Systolic blood pressure | rs7722243 | A | G | -0.2048 | 0.504 | 1.21E-11 | 3.02E-02 | 4.57E-05 | 46.00 |
| Systolic blood pressure | rs7725413 | T | C | -0.1985 | 0.77 | 3.07E-08 | 3.59E-02 | 3.04E-05 | 30.60 |
| Systolic blood pressure | rs77375686 | G | A | 0.3467 | 0.112 | 8.38E-13 | 4.85E-02 | 5.07E-05 | 51.10 |
| Systolic blood pressure | rs7744902 | A | G | -0.4088 | 0.077 | 5.64E-12 | 5.93E-02 | 4.72E-05 | 47.50 |
| Systolic blood pressure | rs7763558 | A | G | 0.3363 | 0.324 | 1.17E-25 | 3.21E-02 | 1.09E-04 | 110.00 |
| Systolic blood pressure | rs7765526 | G | A | -0.201 | 0.537 | 5.88E-11 | 3.07E-02 | 4.26E-05 | 42.90 |
| Systolic blood pressure | rs778124 | A | G | 0.2965 | 0.374 | 1.45E-21 | 3.11E-02 | 9.03E-05 | 90.90 |
| Systolic blood pressure | rs77924615 | A | G | -0.4081 | 0.199 | 1.12E-25 | 3.90E-02 | 1.09E-04 | 109.00 |
| Systolic blood pressure | rs7796 | G | C | -0.3385 | 0.489 | 5.00E-27 | 3.14E-02 | 1.15E-04 | 116.00 |
| Systolic blood pressure | rs7821832 | G | T | -0.4222 | 0.255 | 6.67E-34 | 3.48E-02 | 1.46E-04 | 147.00 |
| Systolic blood pressure | rs7844887 | A | G | 0.2662 | 0.221 | 2.40E-13 | 3.63E-02 | 5.34E-05 | 53.80 |
| Systolic blood pressure | rs78474310 | G | A | 0.4699 | 0.045 | 1.51E-10 | 7.34E-02 | 4.07E-05 | 41.00 |
| Systolic blood pressure | rs78648104 | C | T | 0.4287 | 0.093 | 2.36E-15 | 5.41E-02 | 6.24E-05 | 62.80 |
| Systolic blood pressure | rs786923 | T | C | -0.3082 | 0.624 | 2.82E-23 | 3.10E-02 | 9.82E-05 | 98.80 |
| Systolic blood pressure | rs78998485 | G | C | 0.2449 | 0.256 | 1.48E-12 | 3.46E-02 | 4.98E-05 | 50.10 |
| Systolic blood pressure | rs79069610 | C | T | 0.4005 | 0.05 | 3.68E-08 | 7.27E-02 | 3.01E-05 | 30.30 |
| Systolic blood pressure | rs7912283 | A | G | -0.2144 | 0.647 | 2.94E-11 | 3.22E-02 | 4.40E-05 | 44.30 |
| Systolic blood pressure | rs7926110 | G | T | -0.2603 | 0.327 | 5.71E-16 | 3.21E-02 | 6.53E-05 | 65.80 |
| Systolic blood pressure | rs79384779 | T | C | 0.3179 | 0.151 | 1.08E-13 | 4.28E-02 | 5.48E-05 | 55.20 |
| Systolic blood pressure | rs79539362 | C | T | -0.4003 | 0.101 | 2.09E-15 | 5.04E-02 | 6.26E-05 | 63.10 |
| Systolic blood pressure | rs7963801 | C | T | 0.2362 | 0.578 | 2.87E-14 | 3.11E-02 | 5.73E-05 | 57.70 |
| Systolic blood pressure | rs7980644 | G | A | -0.2641 | 0.833 | 6.30E-11 | 4.04E-02 | 4.24E-05 | 42.70 |
| Systolic blood pressure | rs79930761 | T | C | -0.4688 | 0.087 | 4.90E-17 | 5.59E-02 | 6.98E-05 | 70.30 |
| Systolic blood pressure | rs8003103 | A | G | -0.1755 | 0.345 | 3.60E-08 | 3.19E-02 | 3.01E-05 | 30.30 |
| Systolic blood pressure | rs8030856 | G | C | 0.1764 | 0.395 | 1.21E-08 | 3.10E-02 | 3.22E-05 | 32.40 |
| Systolic blood pressure | rs8044992 | C | T | -0.2138 | 0.288 | 1.07E-10 | 3.31E-02 | 4.14E-05 | 41.70 |
| Systolic blood pressure | rs8054587 | C | T | -0.1665 | 0.473 | 3.41E-08 | 3.02E-02 | 3.02E-05 | 30.40 |
| Systolic blood pressure | rs8079811 | G | C | 0.2101 | 0.652 | 1.03E-10 | 3.25E-02 | 4.15E-05 | 41.80 |
| Systolic blood pressure | rs8113613 | T | C | -0.2334 | 0.181 | 4.59E-09 | 3.98E-02 | 3.42E-05 | 34.40 |
| Systolic blood pressure | rs8125763 | A | C | 0.1761 | 0.472 | 4.84E-09 | 3.01E-02 | 3.40E-05 | 34.20 |
| Systolic blood pressure | rs8142376 | T | C | 0.1676 | 0.491 | 2.19E-08 | 3.00E-02 | 3.10E-05 | 31.20 |
| Systolic blood pressure | rs8180684 | T | C | 0.2134 | 0.29 | 1.80E-10 | 3.35E-02 | 4.03E-05 | 40.60 |
| Systolic blood pressure | rs848445 | C | T | 0.2025 | 0.715 | 2.28E-09 | 3.39E-02 | 3.54E-05 | 35.70 |
| Systolic blood pressure | rs869396 | A | C | -0.2115 | 0.466 | 4.12E-12 | 3.05E-02 | 4.78E-05 | 48.10 |
| Systolic blood pressure | rs871004 | A | G | 0.2336 | 0.348 | 1.65E-13 | 3.17E-02 | 5.39E-05 | 54.30 |
| Systolic blood pressure | rs8904 | A | G | 0.3061 | 0.368 | 1.71E-22 | 3.14E-02 | 9.44E-05 | 95.00 |
| Systolic blood pressure | rs908951 | T | C | -0.2261 | 0.438 | 7.14E-13 | 3.15E-02 | 5.12E-05 | 51.50 |
| Systolic blood pressure | rs927315 | T | C | 0.1689 | 0.471 | 2.44E-08 | 3.03E-02 | 3.09E-05 | 31.10 |
| Systolic blood pressure | rs9285476 | G | C | -0.1844 | 0.293 | 3.07E-08 | 3.33E-02 | 3.05E-05 | 30.70 |
| Systolic blood pressure | rs9302885 | G | A | -0.2242 | 0.555 | 1.03E-13 | 3.02E-02 | 5.47E-05 | 55.10 |
| Systolic blood pressure | rs9327297 | G | C | -0.2747 | 0.332 | 8.07E-18 | 3.19E-02 | 7.36E-05 | 74.20 |
| Systolic blood pressure | rs9349379 | G | A | -0.2664 | 0.407 | 1.31E-17 | 3.12E-02 | 7.24E-05 | 72.90 |
| Systolic blood pressure | rs9361836 | T | C | 0.2196 | 0.317 | 1.25E-11 | 3.24E-02 | 4.56E-05 | 45.90 |
| Systolic blood pressure | rs9368222 | A | C | 0.2281 | 0.269 | 1.84E-11 | 3.39E-02 | 4.50E-05 | 45.30 |
| Systolic blood pressure | rs9401913 | A | G | 0.5202 | 0.439 | 3.66E-65 | 3.05E-02 | 2.89E-04 | 291.00 |
| Systolic blood pressure | rs9486916 | T | C | 0.2657 | 0.198 | 5.42E-12 | 3.85E-02 | 4.73E-05 | 47.60 |
| Systolic blood pressure | rs9507885 | T | C | -0.3208 | 0.095 | 3.23E-09 | 5.42E-02 | 3.48E-05 | 35.00 |
| Systolic blood pressure | rs9508495 | T | C | -0.3557 | 0.757 | 6.34E-24 | 3.53E-02 | 1.01E-04 | 102.00 |
| Systolic blood pressure | rs9526707 | A | G | -0.2039 | 0.322 | 2.77E-10 | 3.23E-02 | 3.96E-05 | 39.90 |
| Systolic blood pressure | rs9549627 | A | G | 0.2846 | 0.118 | 1.25E-08 | 5.00E-02 | 3.22E-05 | 32.40 |
| Systolic blood pressure | rs961764 | G | C | 0.1909 | 0.575 | 3.74E-10 | 3.05E-02 | 3.89E-05 | 39.20 |
| Systolic blood pressure | rs9848170 | C | G | 0.3231 | 0.597 | 7.01E-26 | 3.07E-02 | 1.10E-04 | 111.00 |
| Systolic blood pressure | rs9857362 | C | A | -0.1727 | 0.471 | 1.62E-08 | 3.06E-02 | 3.16E-05 | 31.90 |
| Systolic blood pressure | rs9869437 | A | C | -0.2001 | 0.352 | 3.22E-10 | 3.18E-02 | 3.93E-05 | 39.60 |
| Systolic blood pressure | rs9876694 | T | C | 0.4713 | 0.058 | 4.64E-13 | 6.51E-02 | 5.21E-05 | 52.40 |
| Systolic blood pressure | rs9880098 | A | G | 0.3081 | 0.395 | 1.59E-23 | 3.08E-02 | 9.94E-05 | 100.00 |
| Systolic blood pressure | rs9886665 | C | T | -0.2048 | 0.733 | 2.47E-09 | 3.43E-02 | 3.54E-05 | 35.70 |
| Systolic blood pressure | rs9897429 | A | G | 0.2645 | 0.52 | 1.19E-16 | 3.19E-02 | 6.83E-05 | 68.70 |
| Systolic blood pressure | rs9899540 | T | A | -0.2011 | 0.6 | 1.87E-10 | 3.16E-02 | 4.02E-05 | 40.50 |
| Systolic blood pressure | rs9918876 | A | C | -0.2975 | 0.104 | 2.28E-09 | 4.98E-02 | 3.54E-05 | 35.70 |
| Diastolic blood pressure | rs10048404 | T | C | -0.1096 | 0.369 | 2.00E-09 | 1.83E-02 | 3.56E-05 | 35.87 |
| Diastolic blood pressure | rs10054208 | T | C | 0.1187 | 0.362 | 1.49E-10 | 1.85E-02 | 4.09E-05 | 41.17 |
| Diastolic blood pressure | rs10062049 | T | C | 0.2208 | 0.136 | 4.50E-18 | 2.55E-02 | 7.45E-05 | 74.98 |
| Diastolic blood pressure | rs1006545 | T | G | 0.3633 | 0.888 | 7.96E-40 | 2.75E-02 | 1.73E-04 | 174.53 |
| Diastolic blood pressure | rs10069690 | T | C | 0.1615 | 0.258 | 1.42E-14 | 2.10E-02 | 5.87E-05 | 59.14 |
| Diastolic blood pressure | rs10087280 | G | A | -0.1381 | 0.168 | 2.54E-09 | 2.32E-02 | 3.52E-05 | 35.43 |
| Diastolic blood pressure | rs10164193 | G | T | 0.2196 | 0.078 | 1.87E-11 | 3.27E-02 | 4.48E-05 | 45.10 |
| Diastolic blood pressure | rs10279432 | A | C | 0.1178 | 0.623 | 5.73E-11 | 1.80E-02 | 4.25E-05 | 42.83 |
| Diastolic blood pressure | rs1035673 | C | T | -0.1625 | 0.603 | 3.00E-20 | 1.76E-02 | 8.47E-05 | 85.25 |
| Diastolic blood pressure | rs1039897 | A | G | -0.1085 | 0.65 | 3.26E-09 | 1.83E-02 | 3.49E-05 | 35.15 |
| Diastolic blood pressure | rs1044608 | G | C | 0.2018 | 0.077 | 2.76E-09 | 3.39E-02 | 3.52E-05 | 35.44 |
| Diastolic blood pressure | rs1044822 | T | C | -0.1334 | 0.149 | 4.14E-08 | 2.43E-02 | 2.99E-05 | 30.14 |
| Diastolic blood pressure | rs10490923 | A | G | 0.1533 | 0.126 | 5.02E-09 | 2.62E-02 | 3.40E-05 | 34.24 |
| Diastolic blood pressure | rs10491713 | T | G | -0.1219 | 0.198 | 2.05E-08 | 2.17E-02 | 3.13E-05 | 31.56 |
| Diastolic blood pressure | rs1049212 | G | A | 0.1788 | 0.569 | 1.30E-24 | 1.75E-02 | 1.04E-04 | 104.39 |
| Diastolic blood pressure | rs10493408 | A | C | 0.1584 | 0.133 | 5.09E-10 | 2.55E-02 | 3.83E-05 | 38.59 |
| Diastolic blood pressure | rs10500932 | A | G | 0.2784 | 0.074 | 5.79E-17 | 3.33E-02 | 6.94E-05 | 69.90 |
| Diastolic blood pressure | rs10759697 | A | G | 0.1308 | 0.491 | 3.93E-14 | 1.73E-02 | 5.68E-05 | 57.16 |
| Diastolic blood pressure | rs10776752 | T | G | 0.4573 | 0.081 | 1.25E-43 | 3.30E-02 | 1.91E-04 | 192.03 |
| Diastolic blood pressure | rs10804330 | C | T | -0.1331 | 0.433 | 4.60E-14 | 1.76E-02 | 5.68E-05 | 57.19 |
| Diastolic blood pressure | rs10832586 | C | A | 0.3083 | 0.202 | 2.53E-46 | 2.16E-02 | 2.02E-04 | 203.72 |
| Diastolic blood pressure | rs10838702 | T | G | 0.2375 | 0.388 | 1.27E-40 | 1.78E-02 | 1.77E-04 | 178.03 |
| Diastolic blood pressure | rs10873612 | T | C | -0.1096 | 0.596 | 9.51E-10 | 1.79E-02 | 3.72E-05 | 37.49 |
| Diastolic blood pressure | rs10941043 | G | T | 0.1269 | 0.291 | 2.52E-11 | 1.90E-02 | 4.43E-05 | 44.61 |
| Diastolic blood pressure | rs10980408 | C | T | 0.3745 | 0.036 | 4.17E-15 | 4.77E-02 | 6.12E-05 | 61.64 |
| Diastolic blood pressure | rs11021221 | A | T | -0.1877 | 0.167 | 6.93E-16 | 2.33E-02 | 6.44E-05 | 64.90 |
| Diastolic blood pressure | rs11070245 | G | T | 0.1287 | 0.532 | 1.57E-13 | 1.74E-02 | 5.43E-05 | 54.71 |
| Diastolic blood pressure | rs11077961 | G | A | -0.1073 | 0.368 | 8.55E-09 | 1.86E-02 | 3.31E-05 | 33.28 |
| Diastolic blood pressure | rs11112548 | T | A | -0.2742 | 0.044 | 5.80E-10 | 4.43E-02 | 3.80E-05 | 38.31 |
| Diastolic blood pressure | rs11130602 | A | G | 0.1467 | 0.443 | 4.27E-17 | 1.75E-02 | 6.98E-05 | 70.27 |
| Diastolic blood pressure | rs11141731 | T | C | -0.1258 | 0.228 | 1.31E-09 | 2.07E-02 | 3.67E-05 | 36.93 |
| Diastolic blood pressure | rs1114347 | G | A | 0.1792 | 0.482 | 3.32E-25 | 1.73E-02 | 1.07E-04 | 107.30 |
| Diastolic blood pressure | rs11145807 | G | A | -0.155 | 0.594 | 4.10E-17 | 1.84E-02 | 7.05E-05 | 70.96 |
| Diastolic blood pressure | rs11153590 | A | G | -0.1145 | 0.383 | 1.12E-10 | 1.78E-02 | 4.11E-05 | 41.38 |
| Diastolic blood pressure | rs11153730 | C | T | -0.1551 | 0.491 | 2.57E-19 | 1.73E-02 | 7.98E-05 | 80.38 |
| Diastolic blood pressure | rs11191580 | C | T | -0.5071 | 0.082 | 6.63E-58 | 3.16E-02 | 2.56E-04 | 257.52 |
| Diastolic blood pressure | rs112393817 | G | C | -0.116 | 0.217 | 3.80E-08 | 2.11E-02 | 3.00E-05 | 30.22 |
| Diastolic blood pressure | rs11245631 | T | C | -0.1474 | 0.196 | 3.99E-11 | 2.23E-02 | 4.34E-05 | 43.69 |
| Diastolic blood pressure | rs11252324 | T | G | -0.2339 | 0.077 | 1.03E-12 | 3.28E-02 | 5.05E-05 | 50.85 |
| Diastolic blood pressure | rs1133400 | G | A | 0.1318 | 0.215 | 8.30E-10 | 2.15E-02 | 3.73E-05 | 37.58 |
| Diastolic blood pressure | rs114503346 | T | C | -0.2678 | 0.046 | 3.10E-10 | 4.26E-02 | 3.92E-05 | 39.52 |
| Diastolic blood pressure | rs115447786 | T | C | 0.2904 | 0.043 | 1.75E-10 | 4.55E-02 | 4.05E-05 | 40.74 |
| Diastolic blood pressure | rs11556924 | T | C | -0.181 | 0.383 | 1.83E-23 | 1.81E-02 | 9.93E-05 | 100.00 |
| Diastolic blood pressure | rs11578696 | G | A | -0.1459 | 0.134 | 1.65E-08 | 2.58E-02 | 3.18E-05 | 31.98 |
| Diastolic blood pressure | rs11592107 | A | G | 0.1203 | 0.309 | 1.23E-10 | 1.87E-02 | 4.11E-05 | 41.39 |
| Diastolic blood pressure | rs116063464 | A | G | 0.2017 | 0.06 | 4.68E-08 | 3.69E-02 | 2.97E-05 | 29.88 |
| Diastolic blood pressure | rs11636952 | C | T | -0.3997 | 0.687 | 5.21E-99 | 1.89E-02 | 4.44E-04 | 447.24 |
| Diastolic blood pressure | rs11661473 | A | G | 0.2007 | 0.268 | 1.54E-24 | 1.96E-02 | 1.04E-04 | 104.85 |
| Diastolic blood pressure | rs11664194 | A | T | -0.1078 | 0.46 | 8.69E-10 | 1.76E-02 | 3.73E-05 | 37.52 |
| Diastolic blood pressure | rs11692619 | T | C | -0.1281 | 0.361 | 3.31E-12 | 1.84E-02 | 4.81E-05 | 48.47 |
| Diastolic blood pressure | rs11721984 | T | C | -0.1409 | 0.453 | 1.89E-15 | 1.77E-02 | 6.29E-05 | 63.37 |
| Diastolic blood pressure | rs11745207 | G | C | -0.1132 | 0.258 | 1.29E-08 | 1.99E-02 | 3.21E-05 | 32.36 |
| Diastolic blood pressure | rs11778153 | C | T | -0.1192 | 0.357 | 5.84E-11 | 1.82E-02 | 4.26E-05 | 42.90 |
| Diastolic blood pressure | rs1178979 | C | T | -0.1504 | 0.195 | 9.96E-12 | 2.21E-02 | 4.60E-05 | 46.31 |
| Diastolic blood pressure | rs11859505 | G | A | 0.1037 | 0.581 | 9.76E-09 | 1.81E-02 | 3.26E-05 | 32.82 |
| Diastolic blood pressure | rs11923343 | G | A | 0.1138 | 0.64 | 3.10E-10 | 1.81E-02 | 3.93E-05 | 39.53 |
| Diastolic blood pressure | rs11923667 | A | T | 0.1175 | 0.407 | 3.10E-11 | 1.77E-02 | 4.38E-05 | 44.07 |
| Diastolic blood pressure | rs11945489 | T | C | -0.1392 | 0.291 | 3.99E-13 | 1.92E-02 | 5.22E-05 | 52.56 |
| Diastolic blood pressure | rs11960210 | C | T | -0.2474 | 0.375 | 3.36E-43 | 1.80E-02 | 1.88E-04 | 188.91 |
| Diastolic blood pressure | rs11961593 | T | C | -0.3158 | 0.069 | 1.49E-19 | 3.49E-02 | 8.13E-05 | 81.88 |
| Diastolic blood pressure | rs12088448 | C | A | 0.1544 | 0.356 | 2.53E-17 | 1.82E-02 | 7.15E-05 | 71.97 |
| Diastolic blood pressure | rs1212061 | C | G | 0.1281 | 0.732 | 7.91E-11 | 1.97E-02 | 4.20E-05 | 42.28 |
| Diastolic blood pressure | rs12149254 | A | G | -0.1324 | 0.17 | 1.14E-08 | 2.32E-02 | 3.23E-05 | 32.57 |
| Diastolic blood pressure | rs12152463 | T | C | 0.1006 | 0.425 | 8.01E-09 | 1.74E-02 | 3.32E-05 | 33.43 |
| Diastolic blood pressure | rs1215469 | C | A | 0.1383 | 0.771 | 5.23E-11 | 2.11E-02 | 4.27E-05 | 42.96 |
| Diastolic blood pressure | rs12247028 | A | G | -0.1396 | 0.632 | 1.18E-13 | 1.88E-02 | 5.48E-05 | 55.14 |
| Diastolic blood pressure | rs12258967 | G | C | -0.354 | 0.296 | 3.27E-75 | 1.93E-02 | 3.34E-04 | 336.43 |
| Diastolic blood pressure | rs12321 | C | G | -0.1492 | 0.433 | 1.44E-17 | 1.75E-02 | 7.22E-05 | 72.69 |
| Diastolic blood pressure | rs12337056 | T | C | 0.1364 | 0.176 | 2.18E-09 | 2.28E-02 | 3.55E-05 | 35.79 |
| Diastolic blood pressure | rs12363520 | A | T | 0.1672 | 0.229 | 4.24E-15 | 2.13E-02 | 6.12E-05 | 61.62 |
| Diastolic blood pressure | rs1243876 | T | C | -0.1063 | 0.701 | 2.14E-08 | 1.90E-02 | 3.11E-05 | 31.30 |
| Diastolic blood pressure | rs12503341 | A | G | -0.2993 | 0.039 | 9.43E-11 | 4.62E-02 | 4.17E-05 | 41.97 |
| Diastolic blood pressure | rs12509595 | C | T | 0.4972 | 0.292 | 1.58E-148 | 1.92E-02 | 6.66E-04 | 670.59 |
| Diastolic blood pressure | rs12515541 | T | G | 0.1156 | 0.607 | 6.23E-11 | 1.77E-02 | 4.24E-05 | 42.65 |
| Diastolic blood pressure | rs12574332 | T | C | 0.2072 | 0.123 | 6.14E-15 | 2.66E-02 | 6.03E-05 | 60.68 |
| Diastolic blood pressure | rs12596630 | T | C | 0.2606 | 0.091 | 1.03E-16 | 3.14E-02 | 6.84E-05 | 68.88 |
| Diastolic blood pressure | rs12609484 | T | G | -0.1398 | 0.316 | 1.16E-13 | 1.88E-02 | 5.49E-05 | 55.30 |
| Diastolic blood pressure | rs12627514 | G | C | 0.2164 | 0.29 | 1.99E-28 | 1.96E-02 | 1.21E-04 | 121.90 |
| Diastolic blood pressure | rs1263671 | C | T | 0.1394 | 0.163 | 4.69E-09 | 2.38E-02 | 3.41E-05 | 34.31 |
| Diastolic blood pressure | rs1265157 | G | C | -0.1444 | 0.352 | 1.18E-14 | 1.87E-02 | 5.92E-05 | 59.63 |
| Diastolic blood pressure | rs12656497 | C | T | 0.3063 | 0.597 | 1.47E-67 | 1.76E-02 | 3.01E-04 | 302.88 |
| Diastolic blood pressure | rs1265842 | C | T | -0.1113 | 0.517 | 1.70E-10 | 1.74E-02 | 4.06E-05 | 40.92 |
| Diastolic blood pressure | rs12693302 | A | G | -0.2378 | 0.652 | 2.15E-39 | 1.81E-02 | 1.71E-04 | 172.61 |
| Diastolic blood pressure | rs12728150 | G | A | 0.2045 | 0.081 | 1.28E-10 | 3.18E-02 | 4.11E-05 | 41.36 |
| Diastolic blood pressure | rs1275985 | T | C | -0.2943 | 0.613 | 2.77E-62 | 1.77E-02 | 2.75E-04 | 276.46 |
| Diastolic blood pressure | rs12790943 | T | C | -0.1002 | 0.421 | 1.14E-08 | 1.75E-02 | 3.26E-05 | 32.78 |
| Diastolic blood pressure | rs12866098 | A | G | 0.1033 | 0.342 | 2.73E-08 | 1.86E-02 | 3.06E-05 | 30.84 |
| Diastolic blood pressure | rs12906962 | C | T | 0.2378 | 0.323 | 8.73E-37 | 1.88E-02 | 1.59E-04 | 160.00 |
| Diastolic blood pressure | rs12919839 | T | C | -0.1098 | 0.284 | 1.04E-08 | 1.92E-02 | 3.25E-05 | 32.70 |
| Diastolic blood pressure | rs12929303 | A | G | 0.1572 | 0.533 | 1.58E-19 | 1.74E-02 | 8.11E-05 | 81.62 |
| Diastolic blood pressure | rs12938803 | C | A | 0.1598 | 0.811 | 8.76E-13 | 2.24E-02 | 5.05E-05 | 50.89 |
| Diastolic blood pressure | rs12978472 | G | C | -0.4779 | 0.125 | 8.46E-65 | 2.81E-02 | 2.87E-04 | 289.24 |
| Diastolic blood pressure | rs12990959 | C | T | 0.1271 | 0.313 | 1.11E-11 | 1.87E-02 | 4.59E-05 | 46.20 |
| Diastolic blood pressure | rs13004222 | G | C | -0.2944 | 0.051 | 7.11E-14 | 3.93E-02 | 5.57E-05 | 56.12 |
| Diastolic blood pressure | rs13107325 | T | C | -0.6747 | 0.074 | 3.72E-88 | 3.39E-02 | 3.93E-04 | 396.11 |
| Diastolic blood pressure | rs13118687 | A | G | -0.1496 | 0.47 | 1.37E-17 | 1.75E-02 | 7.26E-05 | 73.08 |
| Diastolic blood pressure | rs13124515 | C | T | 0.1052 | 0.687 | 1.98E-08 | 1.87E-02 | 3.14E-05 | 31.65 |
| Diastolic blood pressure | rs13139571 | A | C | -0.2408 | 0.237 | 2.29E-32 | 2.03E-02 | 1.40E-04 | 140.71 |
| Diastolic blood pressure | rs13152154 | T | C | -0.1186 | 0.729 | 1.23E-09 | 1.95E-02 | 3.67E-05 | 36.99 |
| Diastolic blood pressure | rs13215166 | G | A | 0.3094 | 0.442 | 1.79E-70 | 1.74E-02 | 3.14E-04 | 316.18 |
| Diastolic blood pressure | rs1322639 | A | G | -0.1584 | 0.777 | 3.87E-14 | 2.09E-02 | 5.70E-05 | 57.44 |
| Diastolic blood pressure | rs13237249 | T | C | 0.1366 | 0.398 | 1.02E-14 | 1.77E-02 | 5.92E-05 | 59.56 |
| Diastolic blood pressure | rs1327235 | G | A | 0.3018 | 0.471 | 4.76E-68 | 1.73E-02 | 3.02E-04 | 304.33 |
| Diastolic blood pressure | rs1332812 | A | T | -0.1145 | 0.647 | 2.71E-10 | 1.81E-02 | 3.97E-05 | 40.02 |
| Diastolic blood pressure | rs13355146 | T | C | 0.1224 | 0.383 | 6.39E-12 | 1.78E-02 | 4.70E-05 | 47.28 |
| Diastolic blood pressure | rs13358657 | G | A | 0.224 | 0.133 | 1.70E-18 | 2.55E-02 | 7.66E-05 | 77.16 |
| Diastolic blood pressure | rs135023 | G | A | 0.1036 | 0.578 | 3.53E-09 | 1.75E-02 | 3.48E-05 | 35.05 |
| Diastolic blood pressure | rs1373780 | C | G | 0.1246 | 0.185 | 2.58E-08 | 2.24E-02 | 3.07E-05 | 30.94 |
| Diastolic blood pressure | rs138420351 | T | C | 0.5568 | 0.016 | 7.11E-11 | 8.54E-02 | 4.22E-05 | 42.51 |
| Diastolic blood pressure | rs1390754 | T | C | 0.1258 | 0.399 | 1.19E-12 | 1.77E-02 | 5.02E-05 | 50.51 |
| Diastolic blood pressure | rs142449193 | T | C | -0.2573 | 0.046 | 1.51E-09 | 4.26E-02 | 3.62E-05 | 36.48 |
| Diastolic blood pressure | rs1425486 | T | C | -0.1331 | 0.321 | 1.11E-12 | 1.87E-02 | 5.03E-05 | 50.66 |
| Diastolic blood pressure | rs1433121 | T | C | -0.1352 | 0.691 | 6.91E-13 | 1.88E-02 | 5.14E-05 | 51.72 |
| Diastolic blood pressure | rs1436138 | G | A | -0.1991 | 0.363 | 7.33E-28 | 1.82E-02 | 1.19E-04 | 119.67 |
| Diastolic blood pressure | rs1446468 | C | T | 0.253 | 0.546 | 1.21E-47 | 1.74E-02 | 2.10E-04 | 211.42 |
| Diastolic blood pressure | rs1449596 | G | C | 0.1085 | 0.646 | 1.92E-09 | 1.81E-02 | 3.57E-05 | 35.93 |
| Diastolic blood pressure | rs145422110 | T | C | 0.5356 | 0.016 | 6.41E-13 | 7.45E-02 | 5.13E-05 | 51.69 |
| Diastolic blood pressure | rs1467049 | G | T | -0.1244 | 0.195 | 1.35E-08 | 2.19E-02 | 3.20E-05 | 32.27 |
| Diastolic blood pressure | rs1468816 | C | A | -0.1242 | 0.228 | 4.65E-09 | 2.12E-02 | 3.41E-05 | 34.32 |
| Diastolic blood pressure | rs147501096 | C | G | -0.1955 | 0.072 | 9.94E-09 | 3.41E-02 | 3.26E-05 | 32.87 |
| Diastolic blood pressure | rs148401029 | A | C | -0.3122 | 0.035 | 1.32E-10 | 4.86E-02 | 4.10E-05 | 41.27 |
| Diastolic blood pressure | rs15009 | G | C | 0.1233 | 0.353 | 1.00E-11 | 1.81E-02 | 4.61E-05 | 46.41 |
| Diastolic blood pressure | rs1502358 | A | G | -0.1127 | 0.681 | 1.13E-09 | 1.85E-02 | 3.69E-05 | 37.11 |
| Diastolic blood pressure | rs150816167 | C | T | 0.2873 | 0.045 | 1.17E-10 | 4.46E-02 | 4.12E-05 | 41.50 |
| Diastolic blood pressure | rs1523871 | G | C | 0.1186 | 0.434 | 1.46E-11 | 1.76E-02 | 4.51E-05 | 45.41 |
| Diastolic blood pressure | rs1527797 | T | C | -0.1409 | 0.74 | 8.68E-13 | 1.97E-02 | 5.08E-05 | 51.16 |
| Diastolic blood pressure | rs1528293 | T | A | -0.2764 | 0.508 | 1.48E-57 | 1.73E-02 | 2.53E-04 | 255.26 |
| Diastolic blood pressure | rs1534338 | A | G | -0.1144 | 0.603 | 1.24E-10 | 1.78E-02 | 4.10E-05 | 41.31 |
| Diastolic blood pressure | rs1582931 | A | G | 0.2161 | 0.475 | 4.51E-35 | 1.75E-02 | 1.51E-04 | 152.49 |
| Diastolic blood pressure | rs1623474 | T | C | 0.2234 | 0.33 | 6.24E-34 | 1.84E-02 | 1.46E-04 | 147.41 |
| Diastolic blood pressure | rs1669907 | G | T | -0.1158 | 0.697 | 1.36E-09 | 1.91E-02 | 3.65E-05 | 36.76 |
| Diastolic blood pressure | rs16853198 | G | A | -0.3386 | 0.076 | 4.44E-25 | 3.27E-02 | 1.06E-04 | 107.22 |
| Diastolic blood pressure | rs1687295 | C | T | -0.2061 | 0.73 | 2.99E-26 | 1.94E-02 | 1.12E-04 | 112.86 |
| Diastolic blood pressure | rs16875357 | G | T | 0.1205 | 0.243 | 2.70E-09 | 2.03E-02 | 3.50E-05 | 35.24 |
| Diastolic blood pressure | rs16896276 | A | T | -0.1309 | 0.263 | 3.82E-11 | 1.98E-02 | 4.34E-05 | 43.71 |
| Diastolic blood pressure | rs1693560 | G | A | -0.1525 | 0.46 | 3.41E-18 | 1.75E-02 | 7.54E-05 | 75.94 |
| Diastolic blood pressure | rs17321041 | T | C | 0.2313 | 0.063 | 1.78E-10 | 3.63E-02 | 4.03E-05 | 40.60 |
| Diastolic blood pressure | rs1732664 | C | T | 0.1083 | 0.678 | 2.58E-08 | 1.94E-02 | 3.10E-05 | 31.16 |
| Diastolic blood pressure | rs173396 | A | G | 0.2146 | 0.579 | 1.38E-34 | 1.75E-02 | 1.49E-04 | 150.38 |
| Diastolic blood pressure | rs17396055 | A | G | -0.115 | 0.332 | 4.13E-10 | 1.84E-02 | 3.88E-05 | 39.06 |
| Diastolic blood pressure | rs17432462 | C | T | 0.1036 | 0.377 | 7.31E-09 | 1.79E-02 | 3.33E-05 | 33.50 |
| Diastolic blood pressure | rs17454517 | G | A | -0.1216 | 0.506 | 2.65E-12 | 1.74E-02 | 4.85E-05 | 48.84 |
| Diastolic blood pressure | rs17677603 | G | A | 0.2 | 0.384 | 3.90E-29 | 1.78E-02 | 1.25E-04 | 126.25 |
| Diastolic blood pressure | rs17696749 | G | C | 0.1255 | 0.586 | 9.43E-13 | 1.76E-02 | 5.05E-05 | 50.85 |
| Diastolic blood pressure | rs17807723 | A | G | -0.1757 | 0.138 | 7.44E-12 | 2.57E-02 | 4.64E-05 | 46.74 |
| Diastolic blood pressure | rs17832905 | A | C | 0.1923 | 0.072 | 2.80E-08 | 3.46E-02 | 3.07E-05 | 30.89 |
| Diastolic blood pressure | rs17880989 | A | G | 0.4014 | 0.026 | 1.11E-11 | 5.91E-02 | 4.58E-05 | 46.13 |
| Diastolic blood pressure | rs1790123 | T | C | 0.1991 | 0.803 | 6.87E-20 | 2.18E-02 | 8.28E-05 | 83.41 |
| Diastolic blood pressure | rs1799945 | G | C | 0.3888 | 0.15 | 3.88E-57 | 2.44E-02 | 2.52E-04 | 253.91 |
| Diastolic blood pressure | rs1819663 | G | A | -0.1147 | 0.493 | 4.62E-11 | 1.74E-02 | 4.32E-05 | 43.45 |
| Diastolic blood pressure | rs1867624 | T | C | 0.1412 | 0.615 | 2.08E-15 | 1.78E-02 | 6.25E-05 | 62.93 |
| Diastolic blood pressure | rs1871190 | T | G | 0.1078 | 0.334 | 6.63E-09 | 1.86E-02 | 3.34E-05 | 33.59 |
| Diastolic blood pressure | rs1876490 | A | G | 0.1364 | 0.717 | 1.16E-12 | 1.92E-02 | 5.01E-05 | 50.47 |
| Diastolic blood pressure | rs1882961 | T | C | 0.1272 | 0.309 | 1.40E-11 | 1.88E-02 | 4.55E-05 | 45.78 |
| Diastolic blood pressure | rs1889785 | A | G | 0.1255 | 0.455 | 5.61E-13 | 1.74E-02 | 5.17E-05 | 52.02 |
| Diastolic blood pressure | rs1903752 | T | C | -0.0987 | 0.539 | 3.20E-08 | 1.78E-02 | 3.05E-05 | 30.75 |
| Diastolic blood pressure | rs1906672 | A | G | 0.1402 | 0.232 | 8.47E-12 | 2.05E-02 | 4.65E-05 | 46.77 |
| Diastolic blood pressure | rs194742 | C | T | -0.1281 | 0.831 | 3.17E-08 | 2.32E-02 | 3.03E-05 | 30.49 |
| Diastolic blood pressure | rs1948151 | A | G | -0.1355 | 0.278 | 2.12E-12 | 1.93E-02 | 4.90E-05 | 49.29 |
| Diastolic blood pressure | rs1950500 | C | T | -0.1396 | 0.708 | 2.20E-13 | 1.90E-02 | 5.36E-05 | 53.98 |
| Diastolic blood pressure | rs1999996 | G | A | 0.1124 | 0.441 | 1.67E-10 | 1.76E-02 | 4.05E-05 | 40.79 |
| Diastolic blood pressure | rs2067831 | C | G | -0.128 | 0.272 | 5.08E-11 | 1.95E-02 | 4.28E-05 | 43.09 |
| Diastolic blood pressure | rs2133386 | A | C | -0.1322 | 0.433 | 5.21E-14 | 1.76E-02 | 5.60E-05 | 56.42 |
| Diastolic blood pressure | rs2146315 | T | C | -0.1197 | 0.232 | 5.03E-09 | 2.05E-02 | 3.39E-05 | 34.09 |
| Diastolic blood pressure | rs2160236 | C | G | -0.1421 | 0.379 | 4.31E-15 | 1.81E-02 | 6.12E-05 | 61.64 |
| Diastolic blood pressure | rs2169137 | C | G | 0.1588 | 0.729 | 3.17E-16 | 1.94E-02 | 6.65E-05 | 67.00 |
| Diastolic blood pressure | rs2191046 | G | T | -0.1184 | 0.265 | 1.78E-09 | 1.97E-02 | 3.59E-05 | 36.12 |
| Diastolic blood pressure | rs2236295 | T | G | -0.207 | 0.399 | 1.42E-31 | 1.77E-02 | 1.36E-04 | 136.77 |
| Diastolic blood pressure | rs2239268 | A | G | 0.1097 | 0.701 | 7.40E-09 | 1.90E-02 | 3.31E-05 | 33.34 |
| Diastolic blood pressure | rs2239917 | C | T | -0.1731 | 0.575 | 9.69E-23 | 1.76E-02 | 9.61E-05 | 96.73 |
| Diastolic blood pressure | rs2256187 | A | G | -0.1446 | 0.158 | 1.39E-09 | 2.39E-02 | 3.64E-05 | 36.60 |
| Diastolic blood pressure | rs2271139 | A | C | -0.1247 | 0.286 | 8.23E-11 | 1.92E-02 | 4.19E-05 | 42.18 |
| Diastolic blood pressure | rs2273654 | C | T | -0.1165 | 0.439 | 2.75E-11 | 1.75E-02 | 4.40E-05 | 44.32 |
| Diastolic blood pressure | rs227426 | T | G | 0.1119 | 0.562 | 1.75E-10 | 1.75E-02 | 4.06E-05 | 40.89 |
| Diastolic blood pressure | rs2291516 | A | G | 0.2422 | 0.103 | 9.88E-17 | 2.92E-02 | 6.83E-05 | 68.80 |
| Diastolic blood pressure | rs2305654 | A | C | 0.1707 | 0.345 | 3.99E-20 | 1.86E-02 | 8.36E-05 | 84.22 |
| Diastolic blood pressure | rs2307111 | C | T | 0.1742 | 0.397 | 1.62E-22 | 1.78E-02 | 9.51E-05 | 95.78 |
| Diastolic blood pressure | rs234616 | A | G | -0.1152 | 0.311 | 8.79E-10 | 1.88E-02 | 3.73E-05 | 37.55 |
| Diastolic blood pressure | rs2384061 | A | G | -0.1741 | 0.422 | 2.25E-23 | 1.75E-02 | 9.83E-05 | 98.97 |
| Diastolic blood pressure | rs2397060 | C | T | 0.161 | 0.141 | 1.46E-10 | 2.51E-02 | 4.09E-05 | 41.14 |
| Diastolic blood pressure | rs2421200 | T | G | -0.1097 | 0.488 | 2.59E-10 | 1.73E-02 | 3.99E-05 | 40.21 |
| Diastolic blood pressure | rs2444769 | A | C | 0.158 | 0.795 | 4.85E-13 | 2.19E-02 | 5.17E-05 | 52.05 |
| Diastolic blood pressure | rs2469141 | C | T | -0.1351 | 0.163 | 1.39E-08 | 2.38E-02 | 3.20E-05 | 32.22 |
| Diastolic blood pressure | rs2484294 | A | G | 0.3165 | 0.733 | 1.17E-58 | 1.96E-02 | 2.59E-04 | 260.76 |
| Diastolic blood pressure | rs2487926 | G | A | -0.0972 | 0.43 | 3.31E-08 | 1.76E-02 | 3.03E-05 | 30.50 |
| Diastolic blood pressure | rs2493136 | T | C | 0.2345 | 0.408 | 1.92E-40 | 1.76E-02 | 1.76E-04 | 177.52 |
| Diastolic blood pressure | rs2493296 | T | C | 0.2496 | 0.142 | 7.45E-23 | 2.54E-02 | 9.59E-05 | 96.57 |
| Diastolic blood pressure | rs2515424 | T | C | 0.1278 | 0.434 | 2.18E-13 | 1.74E-02 | 5.36E-05 | 53.95 |
| Diastolic blood pressure | rs2548459 | C | T | 0.132 | 0.52 | 5.95E-14 | 1.76E-02 | 5.59E-05 | 56.25 |
| Diastolic blood pressure | rs2569882 | C | T | -0.1199 | 0.434 | 4.28E-11 | 1.82E-02 | 4.31E-05 | 43.40 |
| Diastolic blood pressure | rs2586970 | G | A | 0.1493 | 0.564 | 1.56E-17 | 1.75E-02 | 7.23E-05 | 72.79 |
| Diastolic blood pressure | rs2589218 | C | T | 0.1207 | 0.27 | 6.90E-10 | 1.96E-02 | 3.77E-05 | 37.92 |
| Diastolic blood pressure | rs2598 | G | A | -0.1387 | 0.467 | 1.94E-15 | 1.75E-02 | 6.24E-05 | 62.82 |
| Diastolic blood pressure | rs2627313 | T | C | 0.151 | 0.446 | 5.85E-18 | 1.75E-02 | 7.39E-05 | 74.45 |
| Diastolic blood pressure | rs2643826 | T | C | 0.1857 | 0.451 | 2.83E-26 | 1.75E-02 | 1.12E-04 | 112.60 |
| Diastolic blood pressure | rs2681485 | A | G | 0.2945 | 0.598 | 1.31E-62 | 1.76E-02 | 2.78E-04 | 279.99 |
| Diastolic blood pressure | rs2744133 | G | A | -0.1435 | 0.275 | 1.17E-13 | 1.93E-02 | 5.49E-05 | 55.28 |
| Diastolic blood pressure | rs28377357 | A | G | -0.1243 | 0.294 | 6.03E-11 | 1.90E-02 | 4.25E-05 | 42.80 |
| Diastolic blood pressure | rs28429256 | A | G | 0.1636 | 0.334 | 2.83E-18 | 1.88E-02 | 7.52E-05 | 75.73 |
| Diastolic blood pressure | rs28544928 | G | T | -0.1543 | 0.254 | 9.13E-15 | 1.99E-02 | 5.97E-05 | 60.12 |
| Diastolic blood pressure | rs2854746 | C | G | 0.113 | 0.4 | 3.26E-10 | 1.80E-02 | 3.91E-05 | 39.41 |
| Diastolic blood pressure | rs28661492 | T | C | -0.1359 | 0.202 | 9.56E-10 | 2.22E-02 | 3.72E-05 | 37.47 |
| Diastolic blood pressure | rs28667801 | T | A | 0.1622 | 0.407 | 1.90E-19 | 1.80E-02 | 8.06E-05 | 81.20 |
| Diastolic blood pressure | rs28675079 | A | G | -0.1444 | 0.187 | 8.34E-11 | 2.22E-02 | 4.20E-05 | 42.31 |
| Diastolic blood pressure | rs2906152 | A | G | -0.1873 | 0.63 | 5.55E-25 | 1.81E-02 | 1.06E-04 | 107.08 |
| Diastolic blood pressure | rs2921604 | C | T | 0.096 | 0.463 | 4.46E-08 | 1.76E-02 | 2.95E-05 | 29.75 |
| Diastolic blood pressure | rs2925345 | C | T | -0.189 | 0.532 | 1.60E-27 | 1.74E-02 | 1.17E-04 | 117.98 |
| Diastolic blood pressure | rs2957468 | G | A | -0.1377 | 0.665 | 8.43E-14 | 1.85E-02 | 5.50E-05 | 55.40 |
| Diastolic blood pressure | rs3006583 | C | T | 0.1303 | 0.189 | 4.66E-09 | 2.22E-02 | 3.42E-05 | 34.45 |
| Diastolic blood pressure | rs310597 | G | A | 0.1152 | 0.37 | 2.18E-10 | 1.81E-02 | 4.02E-05 | 40.51 |
| Diastolic blood pressure | rs311564 | A | G | -0.133 | 0.346 | 4.23E-13 | 1.83E-02 | 5.25E-05 | 52.82 |
| Diastolic blood pressure | rs3117736 | T | C | 0.2374 | 0.266 | 9.71E-34 | 1.96E-02 | 1.46E-04 | 146.71 |
| Diastolic blood pressure | rs318712 | C | T | 0.2389 | 0.076 | 9.17E-13 | 3.34E-02 | 5.08E-05 | 51.16 |
| Diastolic blood pressure | rs335170 | C | A | -0.113 | 0.592 | 1.61E-10 | 1.77E-02 | 4.05E-05 | 40.76 |
| Diastolic blood pressure | rs34130368 | T | G | -0.2027 | 0.117 | 8.77E-13 | 2.84E-02 | 5.06E-05 | 50.94 |
| Diastolic blood pressure | rs342977 | A | G | -0.1577 | 0.772 | 1.67E-14 | 2.05E-02 | 5.88E-05 | 59.18 |
| Diastolic blood pressure | rs34413141 | A | T | -0.1808 | 0.182 | 1.49E-15 | 2.27E-02 | 6.30E-05 | 63.44 |
| Diastolic blood pressure | rs34517439 | A | C | -0.2514 | 0.12 | 2.02E-19 | 2.79E-02 | 8.06E-05 | 81.19 |
| Diastolic blood pressure | rs34587839 | A | G | -0.1669 | 0.154 | 8.22E-12 | 2.44E-02 | 4.65E-05 | 46.79 |
| Diastolic blood pressure | rs34645159 | A | G | -0.133 | 0.501 | 2.07E-14 | 1.74E-02 | 5.80E-05 | 58.43 |
| Diastolic blood pressure | rs347585 | T | C | 0.1506 | 0.701 | 1.57E-15 | 1.89E-02 | 6.31E-05 | 63.49 |
| Diastolic blood pressure | rs35091929 | C | T | -0.1828 | 0.603 | 6.46E-25 | 1.77E-02 | 1.06E-04 | 106.66 |
| Diastolic blood pressure | rs35213536 | T | G | 0.2044 | 0.247 | 2.54E-23 | 2.05E-02 | 9.87E-05 | 99.42 |
| Diastolic blood pressure | rs35261542 | A | C | 0.1196 | 0.268 | 9.29E-10 | 1.95E-02 | 3.74E-05 | 37.62 |
| Diastolic blood pressure | rs35413927 | G | A | 0.1274 | 0.305 | 1.77E-11 | 1.89E-02 | 4.51E-05 | 45.44 |
| Diastolic blood pressure | rs35443 | C | G | -0.2661 | 0.386 | 1.20E-50 | 1.78E-02 | 2.22E-04 | 223.49 |
| Diastolic blood pressure | rs35506078 | C | T | 0.1348 | 0.337 | 1.54E-13 | 1.83E-02 | 5.39E-05 | 54.26 |
| Diastolic blood pressure | rs36169093 | A | G | 0.1231 | 0.504 | 5.56E-12 | 1.79E-02 | 4.70E-05 | 47.29 |
| Diastolic blood pressure | rs3735533 | C | T | 0.487 | 0.926 | 6.32E-49 | 3.31E-02 | 2.15E-04 | 216.47 |
| Diastolic blood pressure | rs3743111 | A | G | 0.1517 | 0.613 | 1.62E-17 | 1.78E-02 | 7.21E-05 | 72.63 |
| Diastolic blood pressure | rs3743369 | A | G | 0.104 | 0.628 | 6.82E-09 | 1.79E-02 | 3.35E-05 | 33.76 |
| Diastolic blood pressure | rs3761077 | T | G | 0.1726 | 0.111 | 1.04E-09 | 2.83E-02 | 3.69E-05 | 37.20 |
| Diastolic blood pressure | rs3772219 | C | A | -0.1754 | 0.319 | 2.94E-21 | 1.85E-02 | 8.93E-05 | 89.89 |
| Diastolic blood pressure | rs3774702 | A | G | 0.147 | 0.177 | 1.18E-10 | 2.28E-02 | 4.13E-05 | 41.57 |
| Diastolic blood pressure | rs3776299 | A | G | 0.1266 | 0.456 | 5.06E-13 | 1.75E-02 | 5.20E-05 | 52.33 |
| Diastolic blood pressure | rs3785837 | A | G | 0.1453 | 0.764 | 9.57E-12 | 2.13E-02 | 4.62E-05 | 46.53 |
| Diastolic blood pressure | rs3798293 | G | A | 0.1328 | 0.217 | 2.70E-10 | 2.10E-02 | 3.97E-05 | 39.99 |
| Diastolic blood pressure | rs3802230 | A | C | -0.1605 | 0.545 | 2.75E-20 | 1.74E-02 | 8.45E-05 | 85.08 |
| Diastolic blood pressure | rs3802517 | A | T | 0.1286 | 0.462 | 9.29E-14 | 1.73E-02 | 5.49E-05 | 55.26 |
| Diastolic blood pressure | rs3807101 | T | C | -0.1743 | 0.123 | 4.57E-11 | 2.65E-02 | 4.30E-05 | 43.26 |
| Diastolic blood pressure | rs3861113 | A | C | 0.2126 | 0.083 | 3.95E-11 | 3.22E-02 | 4.33E-05 | 43.59 |
| Diastolic blood pressure | rs3864004 | A | G | 0.1004 | 0.469 | 6.28E-09 | 1.73E-02 | 3.34E-05 | 33.68 |
| Diastolic blood pressure | rs3916033 | T | C | -0.1233 | 0.557 | 2.41E-11 | 1.85E-02 | 4.41E-05 | 44.42 |
| Diastolic blood pressure | rs3918226 | T | C | 0.6117 | 0.081 | 5.31E-77 | 3.29E-02 | 3.43E-04 | 345.69 |
| Diastolic blood pressure | rs3943093 | T | C | 0.2477 | 0.323 | 3.94E-41 | 1.84E-02 | 1.80E-04 | 181.22 |
| Diastolic blood pressure | rs4074812 | A | G | -0.1336 | 0.554 | 2.07E-14 | 1.75E-02 | 5.79E-05 | 58.28 |
| Diastolic blood pressure | rs4077158 | C | T | 0.1832 | 0.529 | 3.09E-26 | 1.73E-02 | 1.11E-04 | 112.14 |
| Diastolic blood pressure | rs4141663 | T | C | -0.1496 | 0.422 | 1.41E-17 | 1.75E-02 | 7.26E-05 | 73.08 |
| Diastolic blood pressure | rs4244200 | C | G | -0.1215 | 0.28 | 3.23E-10 | 1.93E-02 | 3.94E-05 | 39.63 |
| Diastolic blood pressure | rs4245930 | A | G | -0.1219 | 0.632 | 1.14E-11 | 1.80E-02 | 4.55E-05 | 45.86 |
| Diastolic blood pressure | rs4284362 | A | C | -0.1586 | 0.718 | 3.24E-16 | 1.94E-02 | 6.64E-05 | 66.83 |
| Diastolic blood pressure | rs4295 | G | C | -0.1802 | 0.62 | 1.71E-23 | 1.80E-02 | 9.95E-05 | 100.22 |
| Diastolic blood pressure | rs4306343 | T | A | 0.317 | 0.721 | 8.22E-61 | 1.93E-02 | 2.68E-04 | 269.78 |
| Diastolic blood pressure | rs440454 | G | A | 0.2602 | 0.684 | 7.52E-42 | 1.92E-02 | 1.82E-04 | 183.66 |
| Diastolic blood pressure | rs4424827 | T | C | -0.0981 | 0.567 | 2.11E-08 | 1.75E-02 | 3.12E-05 | 31.42 |
| Diastolic blood pressure | rs4507125 | C | A | 0.1244 | 0.214 | 3.60E-09 | 2.11E-02 | 3.45E-05 | 34.76 |
| Diastolic blood pressure | rs4507656 | G | C | 0.1487 | 0.307 | 8.69E-14 | 1.99E-02 | 5.55E-05 | 55.84 |
| Diastolic blood pressure | rs45474499 | T | C | 0.3562 | 0.047 | 8.50E-18 | 4.15E-02 | 7.32E-05 | 73.67 |
| Diastolic blood pressure | rs4556017 | T | C | -0.1601 | 0.852 | 9.66E-11 | 2.47E-02 | 4.17E-05 | 42.01 |
| Diastolic blood pressure | rs4615669 | G | A | 0.114 | 0.44 | 6.10E-11 | 1.74E-02 | 4.26E-05 | 42.93 |
| Diastolic blood pressure | rs4645335 | G | A | -0.1142 | 0.664 | 7.04E-10 | 1.85E-02 | 3.78E-05 | 38.11 |
| Diastolic blood pressure | rs4675682 | C | T | 0.1409 | 0.462 | 4.49E-16 | 1.73E-02 | 6.59E-05 | 66.33 |
| Diastolic blood pressure | rs4704514 | T | C | 0.1087 | 0.283 | 1.71E-08 | 1.93E-02 | 3.15E-05 | 31.72 |
| Diastolic blood pressure | rs4739832 | C | A | -0.1323 | 0.417 | 5.81E-14 | 1.76E-02 | 5.61E-05 | 56.51 |
| Diastolic blood pressure | rs4743021 | C | T | 0.108 | 0.315 | 2.41E-08 | 1.94E-02 | 3.08E-05 | 30.99 |
| Diastolic blood pressure | rs4756779 | G | A | 0.1456 | 0.176 | 1.48E-10 | 2.27E-02 | 4.09E-05 | 41.14 |
| Diastolic blood pressure | rs4814837 | T | C | -0.1003 | 0.342 | 4.62E-08 | 1.84E-02 | 2.95E-05 | 29.71 |
| Diastolic blood pressure | rs4873492 | T | C | 0.1401 | 0.173 | 1.28E-09 | 2.31E-02 | 3.65E-05 | 36.78 |
| Diastolic blood pressure | rs488834 | T | C | -0.1931 | 0.764 | 1.94E-20 | 2.08E-02 | 8.56E-05 | 86.19 |
| Diastolic blood pressure | rs4890499 | A | G | 0.1131 | 0.255 | 1.33E-08 | 1.99E-02 | 3.21E-05 | 32.30 |
| Diastolic blood pressure | rs4891258 | G | A | 0.1159 | 0.317 | 5.72E-10 | 1.87E-02 | 3.82E-05 | 38.41 |
| Diastolic blood pressure | rs4903064 | C | T | -0.1543 | 0.236 | 7.84E-14 | 2.06E-02 | 5.57E-05 | 56.10 |
| Diastolic blood pressure | rs4909314 | A | T | 0.1339 | 0.395 | 3.41E-14 | 1.77E-02 | 5.68E-05 | 57.23 |
| Diastolic blood pressure | rs4926499 | C | G | 0.1694 | 0.826 | 9.37E-12 | 2.48E-02 | 4.63E-05 | 46.66 |
| Diastolic blood pressure | rs4926901 | A | G | 0.0984 | 0.355 | 4.82E-08 | 1.80E-02 | 2.97E-05 | 29.88 |
| Diastolic blood pressure | rs4926923 | C | T | -0.1918 | 0.088 | 4.75E-10 | 3.08E-02 | 3.85E-05 | 38.78 |
| Diastolic blood pressure | rs4930295 | G | C | -0.2418 | 0.222 | 5.54E-31 | 2.09E-02 | 1.33E-04 | 133.85 |
| Diastolic blood pressure | rs4932373 | C | A | 0.3664 | 0.326 | 7.71E-84 | 1.89E-02 | 3.73E-04 | 375.83 |
| Diastolic blood pressure | rs4936099 | A | C | 0.1745 | 0.599 | 1.16E-22 | 1.78E-02 | 9.54E-05 | 96.11 |
| Diastolic blood pressure | rs4952668 | A | G | -0.192 | 0.624 | 1.13E-26 | 1.80E-02 | 1.13E-04 | 113.78 |
| Diastolic blood pressure | rs4954192 | T | C | -0.1225 | 0.387 | 8.15E-12 | 1.79E-02 | 4.65E-05 | 46.83 |
| Diastolic blood pressure | rs5012479 | G | T | -0.0956 | 0.521 | 3.66E-08 | 1.74E-02 | 3.00E-05 | 30.19 |
| Diastolic blood pressure | rs504217 | T | C | 0.2745 | 0.074 | 2.51E-16 | 3.35E-02 | 6.67E-05 | 67.14 |
| Diastolic blood pressure | rs504691 | A | C | -0.1177 | 0.4 | 3.14E-11 | 1.77E-02 | 4.39E-05 | 44.22 |
| Diastolic blood pressure | rs509067 | C | T | 0.1436 | 0.586 | 2.65E-16 | 1.75E-02 | 6.69E-05 | 67.33 |
| Diastolic blood pressure | rs520592 | G | T | 0.1727 | 0.141 | 3.51E-12 | 2.48E-02 | 4.82E-05 | 48.49 |
| Diastolic blood pressure | rs544012 | G | T | -0.1143 | 0.734 | 1.37E-08 | 2.01E-02 | 3.21E-05 | 32.34 |
| Diastolic blood pressure | rs55684003 | G | A | -0.122 | 0.304 | 1.01E-10 | 1.89E-02 | 4.14E-05 | 41.67 |
| Diastolic blood pressure | rs55770741 | T | C | -0.1281 | 0.561 | 2.20E-13 | 1.75E-02 | 5.32E-05 | 53.58 |
| Diastolic blood pressure | rs55857306 | A | G | -0.5224 | 0.16 | 5.05E-109 | 2.35E-02 | 4.91E-04 | 494.16 |
| Diastolic blood pressure | rs55868524 | A | G | 0.1443 | 0.611 | 5.54E-16 | 1.78E-02 | 6.53E-05 | 65.72 |
| Diastolic blood pressure | rs55944332 | G | A | 0.2365 | 0.237 | 3.27E-31 | 2.04E-02 | 1.33E-04 | 134.40 |
| Diastolic blood pressure | rs55993676 | T | G | -0.2097 | 0.292 | 3.82E-28 | 1.91E-02 | 1.20E-04 | 120.54 |
| Diastolic blood pressure | rs56256111 | A | G | 0.1926 | 0.144 | 2.60E-13 | 2.63E-02 | 5.33E-05 | 53.63 |
| Diastolic blood pressure | rs56809883 | T | G | 0.1102 | 0.26 | 3.03E-08 | 1.99E-02 | 3.05E-05 | 30.67 |
| Diastolic blood pressure | rs57708073 | G | A | -0.1907 | 0.261 | 4.73E-19 | 2.14E-02 | 7.89E-05 | 79.41 |
| Diastolic blood pressure | rs57748895 | T | A | 0.6627 | 0.018 | 2.49E-23 | 6.66E-02 | 9.83E-05 | 99.01 |
| Diastolic blood pressure | rs58693787 | G | A | -0.1584 | 0.246 | 3.82E-15 | 2.02E-02 | 6.11E-05 | 61.49 |
| Diastolic blood pressure | rs5992929 | T | C | 0.1684 | 0.283 | 3.07E-18 | 1.93E-02 | 7.56E-05 | 76.13 |
| Diastolic blood pressure | rs602521 | A | G | 0.1351 | 0.266 | 3.97E-12 | 1.95E-02 | 4.77E-05 | 48.00 |
| Diastolic blood pressure | rs6026739 | T | A | 0.5032 | 0.123 | 1.49E-79 | 2.66E-02 | 3.55E-04 | 357.86 |
| Diastolic blood pressure | rs6031431 | G | A | 0.1153 | 0.462 | 4.94E-11 | 1.75E-02 | 4.31E-05 | 43.41 |
| Diastolic blood pressure | rs604723 | C | T | 0.3848 | 0.725 | 2.32E-87 | 1.94E-02 | 3.91E-04 | 393.43 |
| Diastolic blood pressure | rs6058261 | A | C | -0.1201 | 0.274 | 6.83E-10 | 1.95E-02 | 3.77E-05 | 37.93 |
| Diastolic blood pressure | rs6062477 | T | C | 0.1191 | 0.576 | 1.81E-11 | 1.77E-02 | 4.50E-05 | 45.28 |
| Diastolic blood pressure | rs6078393 | G | T | -0.1205 | 0.411 | 7.66E-12 | 1.76E-02 | 4.66E-05 | 46.88 |
| Diastolic blood pressure | rs6108168 | A | C | -0.1901 | 0.255 | 1.10E-21 | 1.99E-02 | 9.06E-05 | 91.26 |
| Diastolic blood pressure | rs61789369 | G | A | 0.3039 | 0.044 | 3.06E-12 | 4.36E-02 | 4.82E-05 | 48.58 |
| Diastolic blood pressure | rs61909958 | G | C | -0.1275 | 0.188 | 2.21E-08 | 2.28E-02 | 3.11E-05 | 31.27 |
| Diastolic blood pressure | rs61912333 | G | C | -0.1191 | 0.504 | 1.13E-11 | 1.76E-02 | 4.55E-05 | 45.79 |
| Diastolic blood pressure | rs61917655 | T | C | 0.2246 | 0.101 | 3.72E-14 | 2.97E-02 | 5.68E-05 | 57.19 |
| Diastolic blood pressure | rs61948065 | C | A | 0.1737 | 0.121 | 1.17E-10 | 2.70E-02 | 4.11E-05 | 41.39 |
| Diastolic blood pressure | rs62030049 | G | A | -0.1336 | 0.24 | 1.55E-10 | 2.09E-02 | 4.06E-05 | 40.86 |
| Diastolic blood pressure | rs62155750 | G | A | 0.2177 | 0.307 | 8.27E-29 | 1.96E-02 | 1.23E-04 | 123.37 |
| Diastolic blood pressure | rs62158170 | G | A | -0.1645 | 0.217 | 6.63E-15 | 2.11E-02 | 6.04E-05 | 60.78 |
| Diastolic blood pressure | rs62234672 | A | C | 0.1248 | 0.175 | 4.92E-08 | 2.29E-02 | 2.95E-05 | 29.70 |
| Diastolic blood pressure | rs62301873 | G | A | 0.1734 | 0.106 | 1.06E-09 | 2.84E-02 | 3.70E-05 | 37.28 |
| Diastolic blood pressure | rs62378003 | T | C | -0.1714 | 0.114 | 8.90E-10 | 2.80E-02 | 3.72E-05 | 37.47 |
| Diastolic blood pressure | rs62413546 | T | C | -0.1877 | 0.085 | 4.58E-09 | 3.20E-02 | 3.42E-05 | 34.41 |
| Diastolic blood pressure | rs62434124 | T | C | -0.4853 | 0.071 | 7.83E-47 | 3.38E-02 | 2.05E-04 | 206.15 |
| Diastolic blood pressure | rs62503324 | T | C | 0.2033 | 0.24 | 2.11E-23 | 2.04E-02 | 9.86E-05 | 99.31 |
| Diastolic blood pressure | rs6271 | T | C | -0.4313 | 0.074 | 1.72E-34 | 3.52E-02 | 1.49E-04 | 150.13 |
| Diastolic blood pressure | rs636202 | C | T | -0.1023 | 0.519 | 4.40E-09 | 1.74E-02 | 3.43E-05 | 34.57 |
| Diastolic blood pressure | rs6464165 | C | T | 0.217 | 0.281 | 7.34E-29 | 1.95E-02 | 1.23E-04 | 123.84 |
| Diastolic blood pressure | rs6487076 | G | A | -0.174 | 0.223 | 8.69E-17 | 2.09E-02 | 6.88E-05 | 69.31 |
| Diastolic blood pressure | rs6546810 | C | T | 0.12 | 0.353 | 3.16E-11 | 1.81E-02 | 4.37E-05 | 43.95 |
| Diastolic blood pressure | rs6556384 | A | C | -0.152 | 0.811 | 5.91E-12 | 2.21E-02 | 4.70E-05 | 47.30 |
| Diastolic blood pressure | rs6580970 | T | C | -0.1661 | 0.299 | 4.03E-18 | 1.91E-02 | 7.51E-05 | 75.63 |
| Diastolic blood pressure | rs6602177 | T | C | -0.1203 | 0.707 | 6.52E-09 | 2.07E-02 | 3.35E-05 | 33.77 |
| Diastolic blood pressure | rs66682451 | G | A | -0.1348 | 0.275 | 3.44E-12 | 1.94E-02 | 4.79E-05 | 48.28 |
| Diastolic blood pressure | rs6686889 | T | C | 0.1918 | 0.253 | 6.95E-22 | 1.99E-02 | 9.23E-05 | 92.89 |
| Diastolic blood pressure | rs66887589 | C | T | 0.161 | 0.478 | 1.83E-20 | 1.74E-02 | 8.50E-05 | 85.62 |
| Diastolic blood pressure | rs6715901 | A | G | -0.1377 | 0.496 | 2.76E-15 | 1.74E-02 | 6.22E-05 | 62.63 |
| Diastolic blood pressure | rs672272 | T | C | -0.1851 | 0.604 | 2.77E-25 | 1.78E-02 | 1.07E-04 | 108.14 |
| Diastolic blood pressure | rs6735275 | C | T | -0.1225 | 0.271 | 2.62E-10 | 1.94E-02 | 3.96E-05 | 39.87 |
| Diastolic blood pressure | rs6763931 | A | G | 0.1383 | 0.444 | 1.48E-15 | 1.73E-02 | 6.35E-05 | 63.91 |
| Diastolic blood pressure | rs6777317 | A | G | 0.1249 | 0.29 | 1.51E-10 | 1.95E-02 | 4.07E-05 | 41.03 |
| Diastolic blood pressure | rs6779368 | G | A | 0.1791 | 0.342 | 2.28E-22 | 1.84E-02 | 9.41E-05 | 94.74 |
| Diastolic blood pressure | rs6795735 | T | C | -0.1438 | 0.411 | 3.05E-16 | 1.76E-02 | 6.63E-05 | 66.76 |
| Diastolic blood pressure | rs6800730 | G | A | 0.2476 | 0.67 | 8.00E-41 | 1.85E-02 | 1.78E-04 | 179.13 |
| Diastolic blood pressure | rs68085857 | T | C | 0.191 | 0.234 | 9.82E-21 | 2.05E-02 | 8.62E-05 | 86.81 |
| Diastolic blood pressure | rs682681 | C | T | 0.1454 | 0.667 | 4.47E-15 | 1.85E-02 | 6.13E-05 | 61.77 |
| Diastolic blood pressure | rs6875967 | G | A | -0.1344 | 0.648 | 1.21E-13 | 1.81E-02 | 5.48E-05 | 55.14 |
| Diastolic blood pressure | rs6905288 | A | G | 0.1759 | 0.568 | 7.79E-23 | 1.79E-02 | 9.59E-05 | 96.57 |
| Diastolic blood pressure | rs6934891 | A | G | 0.1275 | 0.426 | 5.21E-13 | 1.77E-02 | 5.15E-05 | 51.89 |
| Diastolic blood pressure | rs6961048 | G | C | 0.2729 | 0.104 | 1.28E-21 | 2.86E-02 | 9.04E-05 | 91.05 |
| Diastolic blood pressure | rs6983239 | T | G | 0.1159 | 0.219 | 3.71E-08 | 2.11E-02 | 3.00E-05 | 30.17 |
| Diastolic blood pressure | rs7012891 | C | T | 0.1391 | 0.237 | 1.20E-11 | 2.05E-02 | 4.57E-05 | 46.04 |
| Diastolic blood pressure | rs710249 | C | G | 0.1501 | 0.426 | 6.43E-18 | 1.74E-02 | 7.39E-05 | 74.42 |
| Diastolic blood pressure | rs7106104 | C | T | 0.1186 | 0.281 | 7.72E-10 | 1.93E-02 | 3.75E-05 | 37.76 |
| Diastolic blood pressure | rs710698 | G | A | -0.1059 | 0.414 | 1.88E-09 | 1.76E-02 | 3.60E-05 | 36.20 |
| Diastolic blood pressure | rs7115331 | G | T | 0.1266 | 0.286 | 3.91E-11 | 1.92E-02 | 4.32E-05 | 43.48 |
| Diastolic blood pressure | rs7132012 | G | A | -0.1563 | 0.325 | 3.17E-17 | 1.85E-02 | 7.09E-05 | 71.38 |
| Diastolic blood pressure | rs7134440 | T | C | 0.2278 | 0.083 | 1.75E-12 | 3.23E-02 | 4.94E-05 | 49.74 |
| Diastolic blood pressure | rs7137828 | T | C | -0.5027 | 0.518 | 4.80E-180 | 1.76E-02 | 8.10E-04 | 815.81 |
| Diastolic blood pressure | rs7155504 | C | T | -0.2286 | 0.088 | 5.16E-13 | 3.17E-02 | 5.16E-05 | 52.00 |
| Diastolic blood pressure | rs7192407 | C | T | -0.1019 | 0.528 | 4.53E-09 | 1.74E-02 | 3.41E-05 | 34.30 |
| Diastolic blood pressure | rs7217916 | G | A | -0.1111 | 0.615 | 5.63E-10 | 1.79E-02 | 3.83E-05 | 38.52 |
| Diastolic blood pressure | rs722783 | A | G | -0.2093 | 0.222 | 9.03E-24 | 2.08E-02 | 1.01E-04 | 101.25 |
| Diastolic blood pressure | rs7235890 | T | G | -0.1692 | 0.896 | 4.12E-09 | 2.88E-02 | 3.43E-05 | 34.52 |
| Diastolic blood pressure | rs7257694 | T | C | 0.1837 | 0.4 | 6.28E-25 | 1.78E-02 | 1.06E-04 | 106.51 |
| Diastolic blood pressure | rs7259285 | A | G | 0.105 | 0.447 | 2.65E-09 | 1.76E-02 | 3.53E-05 | 35.59 |
| Diastolic blood pressure | rs72613227 | T | A | 0.1884 | 0.127 | 3.87E-11 | 2.85E-02 | 4.34E-05 | 43.70 |
| Diastolic blood pressure | rs7265695 | C | T | -0.1967 | 0.197 | 2.48E-19 | 2.19E-02 | 8.01E-05 | 80.67 |
| Diastolic blood pressure | rs72683923 | C | T | -0.5325 | 0.021 | 5.02E-17 | 6.35E-02 | 6.98E-05 | 70.32 |
| Diastolic blood pressure | rs72704264 | C | G | 0.117 | 0.217 | 3.60E-08 | 2.12E-02 | 3.02E-05 | 30.46 |
| Diastolic blood pressure | rs72719149 | C | T | 0.1279 | 0.316 | 6.34E-12 | 1.86E-02 | 4.70E-05 | 47.28 |
| Diastolic blood pressure | rs72831343 | G | T | -0.4936 | 0.142 | 4.77E-88 | 2.48E-02 | 3.93E-04 | 396.14 |
| Diastolic blood pressure | rs72842207 | T | C | -0.2112 | 0.215 | 1.10E-23 | 2.11E-02 | 9.95E-05 | 100.19 |
| Diastolic blood pressure | rs72976750 | C | T | 0.1718 | 0.14 | 7.37E-12 | 2.51E-02 | 4.65E-05 | 46.85 |
| Diastolic blood pressure | rs7299936 | A | G | 0.1759 | 0.578 | 1.11E-23 | 1.75E-02 | 1.00E-04 | 101.03 |
| Diastolic blood pressure | rs73033340 | G | A | -0.5312 | 0.036 | 5.06E-24 | 5.25E-02 | 1.02E-04 | 102.38 |
| Diastolic blood pressure | rs73036520 | C | G | 0.1557 | 0.254 | 1.34E-14 | 2.02E-02 | 5.90E-05 | 59.41 |
| Diastolic blood pressure | rs73046792 | A | G | -0.1518 | 0.159 | 5.87E-10 | 2.45E-02 | 3.81E-05 | 38.39 |
| Diastolic blood pressure | rs7321688 | A | C | 0.1507 | 0.233 | 1.99E-13 | 2.05E-02 | 5.37E-05 | 54.04 |
| Diastolic blood pressure | rs7324697 | A | C | 0.1047 | 0.325 | 1.59E-08 | 1.85E-02 | 3.18E-05 | 32.03 |
| Diastolic blood pressure | rs73276406 | C | G | 0.1564 | 0.146 | 1.99E-10 | 2.46E-02 | 4.01E-05 | 40.42 |
| Diastolic blood pressure | rs7350752 | A | G | -0.1504 | 0.124 | 1.97E-08 | 2.68E-02 | 3.13E-05 | 31.49 |
| Diastolic blood pressure | rs7427249 | A | G | -0.1098 | 0.58 | 4.34E-10 | 1.76E-02 | 3.87E-05 | 38.92 |
| Diastolic blood pressure | rs74439044 | C | T | 0.3496 | 0.098 | 1.38E-32 | 2.94E-02 | 1.40E-04 | 141.40 |
| Diastolic blood pressure | rs751984 | C | T | -0.3937 | 0.117 | 1.38E-46 | 2.75E-02 | 2.04E-04 | 204.96 |
| Diastolic blood pressure | rs7524019 | T | C | 0.1036 | 0.492 | 2.60E-09 | 1.74E-02 | 3.52E-05 | 35.45 |
| Diastolic blood pressure | rs75507123 | T | G | -0.1434 | 0.127 | 3.94E-08 | 2.61E-02 | 3.00E-05 | 30.19 |
| Diastolic blood pressure | rs75511781 | G | A | 0.3721 | 0.043 | 2.45E-15 | 4.70E-02 | 6.22E-05 | 62.68 |
| Diastolic blood pressure | rs7569128 | A | C | 0.1981 | 0.819 | 1.22E-18 | 2.25E-02 | 7.70E-05 | 77.52 |
| Diastolic blood pressure | rs7572130 | G | A | 0.1796 | 0.104 | 4.12E-10 | 2.87E-02 | 3.89E-05 | 39.16 |
| Diastolic blood pressure | rs7576060 | T | C | -0.1017 | 0.35 | 2.05E-08 | 1.81E-02 | 3.14E-05 | 31.57 |
| Diastolic blood pressure | rs7611674 | G | T | -0.1576 | 0.196 | 1.67E-12 | 2.23E-02 | 4.96E-05 | 49.95 |
| Diastolic blood pressure | rs7623706 | G | A | -0.0975 | 0.435 | 2.83E-08 | 1.76E-02 | 3.05E-05 | 30.69 |
| Diastolic blood pressure | rs76326501 | C | A | -0.3618 | 0.091 | 2.17E-32 | 3.05E-02 | 1.40E-04 | 140.71 |
| Diastolic blood pressure | rs76452347 | T | C | -0.2246 | 0.205 | 9.37E-23 | 2.29E-02 | 9.55E-05 | 96.19 |
| Diastolic blood pressure | rs76785130 | G | A | 0.4285 | 0.02 | 9.36E-11 | 6.62E-02 | 4.16E-05 | 41.90 |
| Diastolic blood pressure | rs7694000 | T | A | 0.0965 | 0.461 | 3.47E-08 | 1.75E-02 | 3.02E-05 | 30.41 |
| Diastolic blood pressure | rs76954792 | T | C | 0.1213 | 0.232 | 5.06E-09 | 2.08E-02 | 3.38E-05 | 34.01 |
| Diastolic blood pressure | rs7788746 | T | G | -0.1644 | 0.669 | 3.19E-19 | 1.83E-02 | 8.01E-05 | 80.71 |
| Diastolic blood pressure | rs77924615 | A | G | -0.3163 | 0.198 | 3.72E-45 | 2.24E-02 | 1.98E-04 | 199.39 |
| Diastolic blood pressure | rs7800558 | C | T | -0.096 | 0.422 | 4.46E-08 | 1.75E-02 | 2.99E-05 | 30.09 |
| Diastolic blood pressure | rs7805035 | A | T | 0.134 | 0.412 | 2.40E-14 | 1.76E-02 | 5.76E-05 | 57.97 |
| Diastolic blood pressure | rs78151625 | C | T | 0.1869 | 0.166 | 1.04E-15 | 2.33E-02 | 6.39E-05 | 64.34 |
| Diastolic blood pressure | rs78256308 | G | T | -0.4113 | 0.019 | 3.73E-10 | 6.57E-02 | 3.89E-05 | 39.19 |
| Diastolic blood pressure | rs786921 | A | G | -0.1145 | 0.596 | 8.63E-11 | 1.76E-02 | 4.20E-05 | 42.32 |
| Diastolic blood pressure | rs78809139 | A | G | -0.2281 | 0.101 | 2.58E-15 | 2.88E-02 | 6.23E-05 | 62.73 |
| Diastolic blood pressure | rs78909293 | C | T | -0.321 | 0.045 | 7.31E-14 | 4.29E-02 | 5.56E-05 | 55.99 |
| Diastolic blood pressure | rs79044887 | G | C | -0.2427 | 0.148 | 4.01E-23 | 2.45E-02 | 9.75E-05 | 98.13 |
| Diastolic blood pressure | rs7926335 | T | C | 0.1804 | 0.27 | 2.05E-20 | 1.95E-02 | 8.50E-05 | 85.59 |
| Diastolic blood pressure | rs79286081 | A | G | -0.1631 | 0.102 | 4.83E-08 | 2.99E-02 | 2.96E-05 | 29.76 |
| Diastolic blood pressure | rs7933758 | T | C | -0.1138 | 0.305 | 2.58E-09 | 1.91E-02 | 3.53E-05 | 35.50 |
| Diastolic blood pressure | rs7938342 | A | T | 0.2672 | 0.588 | 1.92E-49 | 1.81E-02 | 2.16E-04 | 217.93 |
| Diastolic blood pressure | rs7959649 | C | T | -0.1166 | 0.758 | 8.14E-09 | 2.02E-02 | 3.31E-05 | 33.32 |
| Diastolic blood pressure | rs7967705 | C | T | -0.2694 | 0.62 | 1.54E-51 | 1.78E-02 | 2.27E-04 | 229.06 |
| Diastolic blood pressure | rs79724577 | C | A | -0.1362 | 0.183 | 3.47E-09 | 2.30E-02 | 3.48E-05 | 35.07 |
| Diastolic blood pressure | rs79889784 | T | G | -0.3941 | 0.018 | 3.86E-08 | 7.17E-02 | 3.00E-05 | 30.21 |
| Diastolic blood pressure | rs7992292 | A | G | 0.1367 | 0.824 | 3.19E-09 | 2.31E-02 | 3.48E-05 | 35.02 |
| Diastolic blood pressure | rs80095680 | G | A | 0.1566 | 0.263 | 2.81E-15 | 1.98E-02 | 6.21E-05 | 62.55 |
| Diastolic blood pressure | rs8014182 | T | C | -0.1942 | 0.132 | 3.94E-14 | 2.57E-02 | 5.67E-05 | 57.10 |
| Diastolic blood pressure | rs8078510 | A | G | -0.1277 | 0.269 | 9.84E-11 | 1.97E-02 | 4.17E-05 | 42.02 |
| Diastolic blood pressure | rs824523 | A | C | 0.1226 | 0.334 | 2.26E-11 | 1.83E-02 | 4.46E-05 | 44.88 |
| Diastolic blood pressure | rs881858 | A | G | 0.1553 | 0.694 | 4.65E-16 | 1.91E-02 | 6.57E-05 | 66.11 |
| Diastolic blood pressure | rs882624 | T | C | -0.1571 | 0.333 | 2.33E-17 | 1.85E-02 | 7.16E-05 | 72.11 |
| Diastolic blood pressure | rs908951 | T | C | -0.1983 | 0.437 | 7.73E-28 | 1.81E-02 | 1.19E-04 | 120.03 |
| Diastolic blood pressure | rs917522 | T | C | 0.1665 | 0.885 | 1.04E-09 | 2.73E-02 | 3.69E-05 | 37.20 |
| Diastolic blood pressure | rs926335 | T | C | -0.1215 | 0.476 | 3.37E-12 | 1.75E-02 | 4.79E-05 | 48.20 |
| Diastolic blood pressure | rs9286351 | G | A | 0.1412 | 0.419 | 1.61E-15 | 1.77E-02 | 6.32E-05 | 63.64 |
| Diastolic blood pressure | rs9289557 | T | C | -0.119 | 0.26 | 8.68E-09 | 2.07E-02 | 3.28E-05 | 33.05 |
| Diastolic blood pressure | rs9326869 | C | T | -0.1096 | 0.751 | 3.99E-08 | 2.00E-02 | 2.98E-05 | 30.03 |
| Diastolic blood pressure | rs9365555 | G | A | -0.1254 | 0.326 | 1.96E-11 | 1.87E-02 | 4.47E-05 | 44.97 |
| Diastolic blood pressure | rs9368 | A | C | 0.1121 | 0.387 | 3.24E-10 | 1.78E-02 | 3.94E-05 | 39.66 |
| Diastolic blood pressure | rs9399137 | C | T | -0.1148 | 0.262 | 5.83E-09 | 1.97E-02 | 3.37E-05 | 33.96 |
| Diastolic blood pressure | rs9406076 | T | C | 0.101 | 0.328 | 4.65E-08 | 1.85E-02 | 2.96E-05 | 29.81 |
| Diastolic blood pressure | rs9419374 | G | A | -0.1164 | 0.646 | 3.44E-10 | 1.85E-02 | 3.93E-05 | 39.59 |
| Diastolic blood pressure | rs9467545 | T | A | 0.2545 | 0.157 | 7.39E-27 | 2.37E-02 | 1.15E-04 | 115.31 |
| Diastolic blood pressure | rs9478282 | T | C | -0.1994 | 0.112 | 8.70E-13 | 2.79E-02 | 5.07E-05 | 51.08 |
| Diastolic blood pressure | rs9508495 | T | C | -0.1944 | 0.757 | 1.34E-21 | 2.04E-02 | 9.02E-05 | 90.81 |
| Diastolic blood pressure | rs951914 | C | G | 0.1904 | 0.713 | 5.06E-23 | 1.93E-02 | 9.67E-05 | 97.32 |
| Diastolic blood pressure | rs9526707 | A | G | -0.1217 | 0.322 | 6.59E-11 | 1.86E-02 | 4.25E-05 | 42.81 |
| Diastolic blood pressure | rs962369 | C | T | -0.1684 | 0.301 | 6.02E-19 | 1.89E-02 | 7.88E-05 | 79.39 |
| Diastolic blood pressure | rs964941 | A | G | 0.1726 | 0.518 | 3.31E-23 | 1.74E-02 | 9.77E-05 | 98.40 |
| Diastolic blood pressure | rs9791312 | C | A | 0.1225 | 0.345 | 2.89E-11 | 1.84E-02 | 4.40E-05 | 44.32 |
| Diastolic blood pressure | rs9841978 | A | G | 0.1766 | 0.325 | 1.12E-21 | 1.85E-02 | 9.05E-05 | 91.12 |
| Diastolic blood pressure | rs9889262 | A | T | 0.2283 | 0.367 | 7.11E-37 | 1.80E-02 | 1.60E-04 | 160.87 |
| Diastolic blood pressure | rs9893005 | G | C | 0.1205 | 0.465 | 7.90E-12 | 1.76E-02 | 4.66E-05 | 46.88 |
| Diastolic blood pressure | rs9900637 | A | C | 0.0992 | 0.496 | 1.73E-08 | 1.76E-02 | 3.16E-05 | 31.77 |
| Diastolic blood pressure | rs990619 | G | C | 0.1592 | 0.523 | 2.90E-20 | 1.73E-02 | 8.41E-05 | 84.68 |
| Diastolic blood pressure | rs9918907 | G | A | 0.1188 | 0.216 | 1.59E-08 | 2.10E-02 | 3.18E-05 | 32.00 |
| Diastolic blood pressure | rs9932220 | A | G | -0.1591 | 0.218 | 3.76E-14 | 2.10E-02 | 5.70E-05 | 57.40 |
| Diastolic blood pressure | rs9937801 | C | T | -0.1554 | 0.431 | 4.81E-19 | 1.74E-02 | 7.92E-05 | 79.76 |
| High-density lipoprotein | rs1012306 | C | T | -0.0106663 | 0.439 | 0.0019221 | 2.90E-08 | 7.62E-05 | 30.79 |
| High-density lipoprotein | rs10162642 | G | A | 0.0480088 | 0.788 | 0.0023422 | 2.30E-93 | 1.04E-03 | 420.15 |
| High-density lipoprotein | rs10233430 | T | C | 0.0204688 | 0.573 | 0.001928 | 2.50E-26 | 2.79E-04 | 112.72 |
| High-density lipoprotein | rs1045241 | C | T | -0.0164773 | 0.729 | 0.002156 | 2.10E-14 | 1.45E-04 | 58.41 |
| High-density lipoprotein | rs1047891 | C | A | 0.0188663 | 0.684 | 0.0020439 | 2.70E-20 | 2.11E-04 | 85.20 |
| High-density lipoprotein | rs10504477 | T | C | 0.0150542 | 0.588 | 0.0019312 | 6.40E-15 | 1.50E-04 | 60.77 |
| High-density lipoprotein | rs10513801 | T | G | 0.030283 | 0.863 | 0.0027706 | 8.30E-28 | 2.96E-04 | 119.46 |
| High-density lipoprotein | rs1052373 | C | T | -0.0446282 | 0.678 | 0.0020352 | 1.40E-106 | 1.19E-03 | 480.83 |
| High-density lipoprotein | rs1055582 | C | T | -0.013948 | 0.495 | 0.0019099 | 2.80E-13 | 1.32E-04 | 53.33 |
| High-density lipoprotein | rs10750766 | C | A | 0.018633 | 0.29 | 0.0020995 | 7.00E-19 | 1.95E-04 | 78.77 |
| High-density lipoprotein | rs10761737 | T | C | -0.0140087 | 0.583 | 0.0019375 | 4.80E-13 | 1.29E-04 | 52.28 |
| High-density lipoprotein | rs10774439 | G | A | -0.0205218 | 0.185 | 0.0024834 | 1.40E-16 | 1.69E-04 | 68.28 |
| High-density lipoprotein | rs10786114 | C | T | -0.0237884 | 0.125 | 0.0028826 | 1.60E-16 | 1.69E-04 | 68.10 |
| High-density lipoprotein | rs1083470 | G | A | -0.0115491 | 0.384 | 0.0019599 | 3.80E-09 | 8.60E-05 | 34.72 |
| High-density lipoprotein | rs10846690 | T | C | -0.0213727 | 0.153 | 0.0026736 | 1.30E-15 | 1.58E-04 | 63.91 |
| High-density lipoprotein | rs10879184 | C | T | 0.0108668 | 0.504 | 0.0019184 | 1.50E-08 | 7.94E-05 | 32.09 |
| High-density lipoprotein | rs10901802 | G | C | -0.0107953 | 0.48 | 0.0019118 | 1.60E-08 | 7.89E-05 | 31.88 |
| High-density lipoprotein | rs11009262 | G | T | 0.0234022 | 0.943 | 0.0040929 | 1.10E-08 | 8.09E-05 | 32.69 |
| High-density lipoprotein | rs11021232 | T | C | 0.0167738 | 0.819 | 0.0024871 | 1.50E-11 | 1.13E-04 | 45.49 |
| High-density lipoprotein | rs11045171 | A | G | -0.0283371 | 0.802 | 0.0024059 | 5.00E-32 | 3.43E-04 | 138.73 |
| High-density lipoprotein | rs11057468 | C | T | -0.0139632 | 0.607 | 0.0019715 | 1.40E-12 | 1.24E-04 | 50.16 |
| High-density lipoprotein | rs11118320 | C | G | 0.0147871 | 0.436 | 0.0019166 | 1.20E-14 | 1.47E-04 | 59.53 |
| High-density lipoprotein | rs11171710 | G | A | 0.0113701 | 0.552 | 0.0019295 | 3.80E-09 | 8.60E-05 | 34.72 |
| High-density lipoprotein | rs112001035 | G | A | 0.0466962 | 0.94 | 0.0040836 | 2.80E-30 | 3.24E-04 | 130.76 |
| High-density lipoprotein | rs112192770 | C | T | -0.0286032 | 0.947 | 0.004553 | 3.30E-10 | 9.77E-05 | 39.47 |
| High-density lipoprotein | rs11239536 | T | A | -0.0287591 | 0.759 | 0.0022316 | 5.30E-38 | 4.11E-04 | 166.09 |
| High-density lipoprotein | rs11254464 | T | C | -0.0126591 | 0.577 | 0.0019311 | 5.50E-11 | 1.06E-04 | 42.98 |
| High-density lipoprotein | rs1132274 | C | A | 0.0218615 | 0.846 | 0.0026401 | 1.20E-16 | 1.70E-04 | 68.57 |
| High-density lipoprotein | rs115912456 | A | G | -0.0281485 | 0.959 | 0.004789 | 4.20E-09 | 8.55E-05 | 34.55 |
| High-density lipoprotein | rs116006942 | G | A | 0.0300143 | 0.939 | 0.0040353 | 1.00E-13 | 1.37E-04 | 55.32 |
| High-density lipoprotein | rs11631178 | T | C | -0.0210646 | 0.896 | 0.0031239 | 1.50E-11 | 1.13E-04 | 45.47 |
| High-density lipoprotein | rs11640494 | G | A | 0.0154267 | 0.543 | 0.0018999 | 4.70E-16 | 1.63E-04 | 65.93 |
| High-density lipoprotein | rs11645157 | T | G | -0.0186404 | 0.505 | 0.0018854 | 4.80E-23 | 2.42E-04 | 97.74 |
| High-density lipoprotein | rs11664369 | C | T | 0.0230294 | 0.733 | 0.0021589 | 1.40E-26 | 2.82E-04 | 113.79 |
| High-density lipoprotein | rs1168114 | A | G | -0.0155704 | 0.348 | 0.0019984 | 6.60E-15 | 1.50E-04 | 60.71 |
| High-density lipoprotein | rs116843064 | G | A | -0.206057 | 0.981 | 0.0069185 | 6.40E-195 | 2.19E-03 | 887.06 |
| High-density lipoprotein | rs11688682 | G | C | -0.0147911 | 0.729 | 0.0022135 | 2.40E-11 | 1.11E-04 | 44.65 |
| High-density lipoprotein | rs116978226 | C | A | -0.0678207 | 0.967 | 0.0053845 | 2.20E-36 | 3.93E-04 | 158.65 |
| High-density lipoprotein | rs117230571 | A | G | 0.0266942 | 0.923 | 0.0036315 | 2.00E-13 | 1.34E-04 | 54.03 |
| High-density lipoprotein | rs117687565 | C | T | -0.0826934 | 0.988 | 0.0091208 | 1.20E-19 | 2.03E-04 | 82.20 |
| High-density lipoprotein | rs11789603 | C | T | -0.0685258 | 0.892 | 0.0030766 | 6.70E-110 | 1.23E-03 | 496.11 |
| High-density lipoprotein | rs11878235 | G | A | -0.0127368 | 0.407 | 0.0019607 | 8.30E-11 | 1.04E-04 | 42.20 |
| High-density lipoprotein | rs11948445 | A | G | 0.0111655 | 0.589 | 0.0019717 | 1.50E-08 | 7.94E-05 | 32.07 |
| High-density lipoprotein | rs12146566 | A | C | 0.0136347 | 0.803 | 0.0023948 | 1.20E-08 | 8.02E-05 | 32.42 |
| High-density lipoprotein | rs1225053 | T | C | 0.0149852 | 0.736 | 0.0021671 | 4.70E-12 | 1.18E-04 | 47.82 |
| High-density lipoprotein | rs1240820 | G | A | -0.0131815 | 0.707 | 0.0021007 | 3.50E-10 | 9.75E-05 | 39.37 |
| High-density lipoprotein | rs12411959 | A | T | 0.015565 | 0.779 | 0.0023065 | 1.50E-11 | 1.13E-04 | 45.54 |
| High-density lipoprotein | rs12485478 | A | G | 0.0524164 | 0.973 | 0.0059402 | 1.10E-18 | 1.93E-04 | 77.86 |
| High-density lipoprotein | rs12510382 | A | G | -0.0165672 | 0.54 | 0.0019233 | 7.10E-18 | 1.84E-04 | 74.20 |
| High-density lipoprotein | rs12705595 | G | A | -0.0110687 | 0.627 | 0.0019833 | 2.40E-08 | 7.71E-05 | 31.15 |
| High-density lipoprotein | rs12740374 | G | T | -0.0288626 | 0.779 | 0.0022913 | 2.20E-36 | 3.93E-04 | 158.68 |
| High-density lipoprotein | rs12803463 | G | A | -0.0237269 | 0.919 | 0.0034928 | 1.10E-11 | 1.14E-04 | 46.15 |
| High-density lipoprotein | rs12926854 | A | G | -0.0123559 | 0.73 | 0.0021336 | 7.00E-09 | 8.30E-05 | 33.54 |
| High-density lipoprotein | rs12928099 | C | A | -0.0213868 | 0.704 | 0.0020681 | 4.60E-25 | 2.65E-04 | 106.95 |
| High-density lipoprotein | rs12976395 | G | C | -0.041418 | 0.496 | 0.0020517 | 1.30E-90 | 1.01E-03 | 407.53 |
| High-density lipoprotein | rs12986742 | T | C | 0.0105189 | 0.524 | 0.0019133 | 3.80E-08 | 7.48E-05 | 30.22 |
| High-density lipoprotein | rs12998038 | C | T | -0.0132202 | 0.741 | 0.0021849 | 1.40E-09 | 9.06E-05 | 36.61 |
| High-density lipoprotein | rs13066793 | A | G | -0.0220822 | 0.91 | 0.0033292 | 3.30E-11 | 1.09E-04 | 43.99 |
| High-density lipoprotein | rs13107325 | C | T | 0.0801624 | 0.925 | 0.0036247 | 2.20E-108 | 1.21E-03 | 489.10 |
| High-density lipoprotein | rs13111599 | A | G | -0.0128995 | 0.263 | 0.0021669 | 2.60E-09 | 8.77E-05 | 35.44 |
| High-density lipoprotein | rs13144151 | A | G | -0.0178651 | 0.15 | 0.0027037 | 3.90E-11 | 1.08E-04 | 43.66 |
| High-density lipoprotein | rs13235365 | C | T | -0.0257631 | 0.726 | 0.0021441 | 2.90E-33 | 3.57E-04 | 144.39 |
| High-density lipoprotein | rs13269725 | A | G | 0.0259348 | 0.922 | 0.0035329 | 2.10E-13 | 1.33E-04 | 53.89 |
| High-density lipoprotein | rs13300004 | A | G | 0.0487788 | 0.987 | 0.0085631 | 1.20E-08 | 8.03E-05 | 32.45 |
| High-density lipoprotein | rs133015 | C | G | -0.0205714 | 0.56 | 0.0019288 | 1.50E-26 | 2.82E-04 | 113.75 |
| High-density lipoprotein | rs13323506 | A | C | -0.0131152 | 0.311 | 0.0020583 | 1.90E-10 | 1.00E-04 | 40.60 |
| High-density lipoprotein | rs13379043 | T | C | -0.0198078 | 0.72 | 0.0021674 | 6.30E-20 | 2.07E-04 | 83.52 |
| High-density lipoprotein | rs13389219 | C | T | -0.0276526 | 0.607 | 0.0019463 | 8.20E-46 | 4.99E-04 | 201.87 |
| High-density lipoprotein | rs1349852 | A | C | -0.011216 | 0.525 | 0.0019259 | 5.70E-09 | 8.40E-05 | 33.92 |
| High-density lipoprotein | rs138026745 | G | A | -0.0336451 | 0.974 | 0.0061597 | 4.70E-08 | 7.39E-05 | 29.83 |
| High-density lipoprotein | rs1395221 | G | T | 0.0111605 | 0.602 | 0.0019538 | 1.10E-08 | 8.08E-05 | 32.63 |
| High-density lipoprotein | rs140064750 | T | C | 0.0439765 | 0.975 | 0.0063425 | 4.10E-12 | 1.19E-04 | 48.08 |
| High-density lipoprotein | rs140201358 | C | G | 0.0578616 | 0.986 | 0.0081741 | 1.50E-12 | 1.24E-04 | 50.11 |
| High-density lipoprotein | rs141062196 | G | A | 0.0188322 | 0.806 | 0.0024112 | 5.70E-15 | 1.51E-04 | 61.00 |
| High-density lipoprotein | rs1411432 | A | C | 0.0138488 | 0.814 | 0.0024559 | 1.70E-08 | 7.87E-05 | 31.80 |
| High-density lipoprotein | rs1412234 | T | C | 0.0120266 | 0.673 | 0.0020361 | 3.50E-09 | 8.64E-05 | 34.89 |
| High-density lipoprotein | rs141440048 | C | T | -0.0438819 | 0.982 | 0.0075683 | 6.70E-09 | 8.32E-05 | 33.62 |
| High-density lipoprotein | rs141469619 | A | G | 0.203826 | 0.99 | 0.0101869 | 4.60E-89 | 9.90E-04 | 400.34 |
| High-density lipoprotein | rs142288236 | C | T | 0.0775348 | 0.985 | 0.0079569 | 2.00E-22 | 2.35E-04 | 94.95 |
| High-density lipoprotein | rs142493909 | T | C | -0.0671377 | 0.981 | 0.0070155 | 1.10E-21 | 2.27E-04 | 91.58 |
| High-density lipoprotein | rs1431659 | A | G | -0.0127629 | 0.272 | 0.0021413 | 2.50E-09 | 8.79E-05 | 35.53 |
| High-density lipoprotein | rs144033177 | A | C | 0.0558275 | 0.984 | 0.0078399 | 1.10E-12 | 1.26E-04 | 50.71 |
| High-density lipoprotein | rs144311893 | C | T | -0.0810821 | 0.978 | 0.0066923 | 8.70E-34 | 3.63E-04 | 146.79 |
| High-density lipoprotein | rs1446585 | A | G | -0.0167703 | 0.756 | 0.0021717 | 1.10E-14 | 1.48E-04 | 59.63 |
| High-density lipoprotein | rs150237291 | T | C | -0.045222 | 0.978 | 0.0065637 | 5.60E-12 | 1.17E-04 | 47.47 |
| High-density lipoprotein | rs150844304 | A | C | 0.0904093 | 0.974 | 0.0059927 | 2.00E-51 | 5.63E-04 | 227.60 |
| High-density lipoprotein | rs1534696 | C | A | -0.0166792 | 0.459 | 0.0019115 | 2.60E-18 | 1.88E-04 | 76.14 |
| High-density lipoprotein | rs1567353 | C | G | 0.0113421 | 0.692 | 0.0020738 | 4.50E-08 | 7.40E-05 | 29.91 |
| High-density lipoprotein | rs16928809 | G | A | 0.0263539 | 0.907 | 0.0033003 | 1.40E-15 | 1.58E-04 | 63.77 |
| High-density lipoprotein | rs16969990 | C | T | -0.0248864 | 0.93 | 0.0037327 | 2.60E-11 | 1.10E-04 | 44.45 |
| High-density lipoprotein | rs17124112 | C | A | 0.0207538 | 0.92 | 0.0035298 | 4.10E-09 | 8.56E-05 | 34.57 |
| High-density lipoprotein | rs17138358 | G | C | 0.0271383 | 0.602 | 0.0019483 | 4.20E-44 | 4.80E-04 | 194.02 |
| High-density lipoprotein | rs17309930 | C | A | 0.021922 | 0.795 | 0.0023556 | 1.30E-20 | 2.14E-04 | 86.61 |
| High-density lipoprotein | rs17326656 | G | T | 0.0225271 | 0.761 | 0.0022391 | 8.20E-24 | 2.51E-04 | 101.22 |
| High-density lipoprotein | rs174566 | A | G | 0.0562453 | 0.65 | 0.0019957 | 9.50E-175 | 1.96E-03 | 794.26 |
| High-density lipoprotein | rs1760940 | A | C | -0.012146 | 0.753 | 0.0022159 | 4.20E-08 | 7.44E-05 | 30.04 |
| High-density lipoprotein | rs1771582 | T | G | -0.0128979 | 0.443 | 0.0019785 | 7.10E-11 | 1.05E-04 | 42.50 |
| High-density lipoprotein | rs1800588 | C | T | -0.117819 | 0.784 | 0.0023118 | 1.00E-200 | 6.39E-03 | 2597.29 |
| High-density lipoprotein | rs183906992 | T | C | -0.029467 | 0.957 | 0.004748 | 5.40E-10 | 9.53E-05 | 38.52 |
| High-density lipoprotein | rs185073199 | T | A | -0.0637345 | 0.99 | 0.010073 | 2.50E-10 | 9.91E-05 | 40.03 |
| High-density lipoprotein | rs1862205 | G | A | -0.0112811 | 0.595 | 0.001944 | 6.50E-09 | 8.34E-05 | 33.67 |
| High-density lipoprotein | rs193084249 | A | G | 0.0828018 | 0.977 | 0.0064127 | 3.80E-38 | 4.13E-04 | 166.72 |
| High-density lipoprotein | rs1955512 | G | A | -0.0110036 | 0.424 | 0.0019703 | 2.30E-08 | 7.72E-05 | 31.19 |
| High-density lipoprotein | rs1970811 | T | C | 0.0115949 | 0.542 | 0.0019178 | 1.50E-09 | 9.05E-05 | 36.55 |
| High-density lipoprotein | rs200540247 | A | C | -0.0133506 | 0.289 | 0.0021247 | 3.30E-10 | 9.77E-05 | 39.48 |
| High-density lipoprotein | rs2011614 | G | A | 0.0144905 | 0.649 | 0.0019993 | 4.20E-13 | 1.30E-04 | 52.53 |
| High-density lipoprotein | rs201441 | T | G | 0.0110517 | 0.419 | 0.0019344 | 1.10E-08 | 8.08E-05 | 32.64 |
| High-density lipoprotein | rs2066714 | T | C | -0.0465057 | 0.871 | 0.002844 | 4.20E-60 | 6.62E-04 | 267.39 |
| High-density lipoprotein | rs2068888 | G | A | -0.0191975 | 0.55 | 0.0019154 | 1.20E-23 | 2.49E-04 | 100.45 |
| High-density lipoprotein | rs2071379 | A | G | 0.0134628 | 0.405 | 0.001943 | 4.20E-12 | 1.19E-04 | 48.01 |
| High-density lipoprotein | rs2098918 | C | T | -0.0118408 | 0.545 | 0.0019162 | 6.40E-10 | 9.45E-05 | 38.18 |
| High-density lipoprotein | rs2155220 | C | T | 0.0105782 | 0.562 | 0.0019134 | 3.20E-08 | 7.57E-05 | 30.57 |
| High-density lipoprotein | rs2159607 | G | T | 0.0238894 | 0.189 | 0.0024324 | 9.10E-23 | 2.39E-04 | 96.46 |
| High-density lipoprotein | rs2237035 | G | T | -0.0138982 | 0.614 | 0.0019585 | 1.30E-12 | 1.25E-04 | 50.36 |
| High-density lipoprotein | rs2247355 | C | T | -0.0205787 | 0.817 | 0.002459 | 5.80E-17 | 1.73E-04 | 70.04 |
| High-density lipoprotein | rs2256609 | A | G | 0.0328809 | 0.811 | 0.0024409 | 2.30E-41 | 4.49E-04 | 181.46 |
| High-density lipoprotein | rs2268840 | T | C | -0.0171774 | 0.772 | 0.0022679 | 3.60E-14 | 1.42E-04 | 57.37 |
| High-density lipoprotein | rs2281718 | A | T | -0.0596767 | 0.387 | 0.0019543 | 1.00E-200 | 2.30E-03 | 932.47 |
| High-density lipoprotein | rs2297402 | C | T | 0.0684609 | 0.976 | 0.0065068 | 6.90E-26 | 2.74E-04 | 110.70 |
| High-density lipoprotein | rs2298214 | C | A | 0.0124547 | 0.423 | 0.0019412 | 1.40E-10 | 1.02E-04 | 41.17 |
| High-density lipoprotein | rs2298624 | C | T | -0.0300608 | 0.867 | 0.0028095 | 1.00E-26 | 2.83E-04 | 114.49 |
| High-density lipoprotein | rs2298632 | C | T | -0.0142985 | 0.502 | 0.0019376 | 1.60E-13 | 1.35E-04 | 54.46 |
| High-density lipoprotein | rs2302367 | T | C | 0.0106511 | 0.509 | 0.0019164 | 2.70E-08 | 7.65E-05 | 30.89 |
| High-density lipoprotein | rs2307111 | T | C | -0.0190204 | 0.605 | 0.0019523 | 2.00E-22 | 2.35E-04 | 94.92 |
| High-density lipoprotein | rs2339234 | G | A | 0.0119298 | 0.317 | 0.0020574 | 6.70E-09 | 8.32E-05 | 33.62 |
| High-density lipoprotein | rs235314 | C | T | 0.0177558 | 0.468 | 0.0019202 | 2.30E-20 | 2.12E-04 | 85.51 |
| High-density lipoprotein | rs2362541 | T | G | 0.0108519 | 0.492 | 0.0019034 | 1.20E-08 | 8.05E-05 | 32.50 |
| High-density lipoprotein | rs2364723 | G | C | -0.0121743 | 0.681 | 0.0020466 | 2.70E-09 | 8.76E-05 | 35.39 |
| High-density lipoprotein | rs2417125 | A | G | 0.0132127 | 0.716 | 0.0021168 | 4.30E-10 | 9.64E-05 | 38.96 |
| High-density lipoprotein | rs2435307 | C | T | -0.0161055 | 0.514 | 0.0019094 | 3.30E-17 | 1.76E-04 | 71.14 |
| High-density lipoprotein | rs248653 | T | A | 0.0319312 | 0.963 | 0.0050863 | 3.40E-10 | 9.76E-05 | 39.41 |
| High-density lipoprotein | rs2498786 | C | G | 0.0253952 | 0.384 | 0.0019685 | 4.40E-38 | 4.12E-04 | 166.43 |
| High-density lipoprotein | rs2520096 | A | G | -0.0147445 | 0.73 | 0.0021514 | 7.20E-12 | 1.16E-04 | 46.97 |
| High-density lipoprotein | rs2534596 | A | G | -0.0113602 | 0.62 | 0.0019751 | 8.80E-09 | 8.19E-05 | 33.08 |
| High-density lipoprotein | rs254024 | G | T | 0.0104752 | 0.562 | 0.0019209 | 4.90E-08 | 7.36E-05 | 29.74 |
| High-density lipoprotein | rs254559 | C | A | 0.0114965 | 0.595 | 0.0019446 | 3.40E-09 | 8.65E-05 | 34.95 |
| High-density lipoprotein | rs2585398 | C | A | 0.0129471 | 0.451 | 0.001927 | 1.80E-11 | 1.12E-04 | 45.14 |
| High-density lipoprotein | rs2642438 | A | G | -0.027676 | 0.297 | 0.0020787 | 1.90E-40 | 4.39E-04 | 177.26 |
| High-density lipoprotein | rs2645979 | G | A | -0.0114379 | 0.643 | 0.0019863 | 8.50E-09 | 8.21E-05 | 33.16 |
| High-density lipoprotein | rs267738 | T | G | -0.0214169 | 0.781 | 0.0022978 | 1.20E-20 | 2.15E-04 | 86.87 |
| High-density lipoprotein | rs2723065 | A | G | -0.0149555 | 0.623 | 0.0019626 | 2.50E-14 | 1.44E-04 | 58.07 |
| High-density lipoprotein | rs2740488 | A | C | 0.0687851 | 0.735 | 0.002162 | 1.00E-200 | 2.50E-03 | 1012.20 |
| High-density lipoprotein | rs2792751 | T | C | 0.0361015 | 0.275 | 0.0021339 | 3.30E-64 | 7.08E-04 | 286.23 |
| High-density lipoprotein | rs2804894 | G | A | -0.0173213 | 0.265 | 0.0021844 | 2.20E-15 | 1.56E-04 | 62.88 |
| High-density lipoprotein | rs28362901 | C | A | 0.0239698 | 0.912 | 0.0033633 | 1.00E-12 | 1.26E-04 | 50.79 |
| High-density lipoprotein | rs28455602 | A | G | -0.0138196 | 0.815 | 0.0024622 | 2.00E-08 | 7.80E-05 | 31.50 |
| High-density lipoprotein | rs28510484 | G | C | 0.0154202 | 0.83 | 0.0025396 | 1.30E-09 | 9.13E-05 | 36.87 |
| High-density lipoprotein | rs2910949 | T | G | -0.0134098 | 0.645 | 0.0019991 | 2.00E-11 | 1.11E-04 | 44.99 |
| High-density lipoprotein | rs2925979 | T | C | -0.0372895 | 0.3 | 0.0020568 | 1.90E-73 | 8.13E-04 | 328.69 |
| High-density lipoprotein | rs2943645 | C | T | 0.0434402 | 0.353 | 0.0019885 | 8.60E-106 | 1.18E-03 | 477.22 |
| High-density lipoprotein | rs2963468 | A | G | 0.0196752 | 0.765 | 0.0022628 | 3.50E-18 | 1.87E-04 | 75.60 |
| High-density lipoprotein | rs2965169 | A | C | -0.0121294 | 0.611 | 0.0019547 | 5.50E-10 | 9.53E-05 | 38.50 |
| High-density lipoprotein | rs308 | T | G | -0.126438 | 0.979 | 0.0066938 | 1.40E-79 | 8.82E-04 | 356.78 |
| High-density lipoprotein | rs3184504 | T | C | -0.0265573 | 0.483 | 0.0019047 | 3.50E-44 | 4.81E-04 | 194.40 |
| High-density lipoprotein | rs32578 | G | A | -0.0133241 | 0.691 | 0.0020654 | 1.10E-10 | 1.03E-04 | 41.62 |
| High-density lipoprotein | rs330042 | G | A | -0.0145553 | 0.734 | 0.0021518 | 1.30E-11 | 1.13E-04 | 45.76 |
| High-density lipoprotein | rs34138141 | G | T | 0.0175419 | 0.719 | 0.0021249 | 1.50E-16 | 1.69E-04 | 68.15 |
| High-density lipoprotein | rs343 | C | A | -0.133707 | 0.917 | 0.0034645 | 1.00E-200 | 3.67E-03 | 1489.41 |
| High-density lipoprotein | rs34717889 | T | A | -0.0144811 | 0.803 | 0.0024522 | 3.50E-09 | 8.63E-05 | 34.87 |
| High-density lipoprotein | rs34940374 | G | A | 0.0170097 | 0.817 | 0.0024705 | 5.80E-12 | 1.17E-04 | 47.40 |
| High-density lipoprotein | rs35137994 | C | T | -0.0463913 | 0.944 | 0.0041362 | 3.40E-29 | 3.11E-04 | 125.79 |
| High-density lipoprotein | rs35493868 | C | G | -0.0372747 | 0.798 | 0.002378 | 2.30E-55 | 6.08E-04 | 245.70 |
| High-density lipoprotein | rs35580606 | A | G | 0.0108931 | 0.483 | 0.0019103 | 1.20E-08 | 8.05E-05 | 32.52 |
| High-density lipoprotein | rs35777071 | T | C | -0.0158975 | 0.346 | 0.0020194 | 3.50E-15 | 1.53E-04 | 61.98 |
| High-density lipoprotein | rs35828909 | A | C | 0.0110498 | 0.447 | 0.0019502 | 1.50E-08 | 7.95E-05 | 32.10 |
| High-density lipoprotein | rs36092527 | T | C | 0.0168993 | 0.885 | 0.0029844 | 1.50E-08 | 7.94E-05 | 32.06 |
| High-density lipoprotein | rs367070 | A | G | -0.0417967 | 0.774 | 0.0022874 | 1.40E-74 | 8.26E-04 | 333.90 |
| High-density lipoprotein | rs367677 | A | G | -0.0167198 | 0.76 | 0.0022481 | 1.00E-13 | 1.37E-04 | 55.31 |
| High-density lipoprotein | rs3732356 | G | T | 0.0299853 | 0.066 | 0.0038656 | 8.70E-15 | 1.49E-04 | 60.17 |
| High-density lipoprotein | rs3736802 | T | C | -0.0168562 | 0.484 | 0.0019101 | 1.10E-18 | 1.93E-04 | 77.88 |
| High-density lipoprotein | rs3747973 | A | G | -0.0141432 | 0.407 | 0.0019382 | 2.90E-13 | 1.32E-04 | 53.24 |
| High-density lipoprotein | rs3763236 | T | C | -0.0147886 | 0.491 | 0.0019045 | 8.20E-15 | 1.49E-04 | 60.29 |
| High-density lipoprotein | rs3768321 | G | T | 0.0452523 | 0.803 | 0.0023951 | 1.30E-79 | 8.83E-04 | 356.98 |
| High-density lipoprotein | rs3814883 | C | T | 0.0152578 | 0.518 | 0.0018917 | 7.30E-16 | 1.61E-04 | 65.05 |
| High-density lipoprotein | rs38166 | T | C | -0.0125618 | 0.249 | 0.0022147 | 1.40E-08 | 7.96E-05 | 32.17 |
| High-density lipoprotein | rs3903399 | T | C | 0.0129246 | 0.789 | 0.0023297 | 2.90E-08 | 7.62E-05 | 30.78 |
| High-density lipoprotein | rs3936511 | A | G | 0.0303959 | 0.808 | 0.0024205 | 3.60E-36 | 3.90E-04 | 157.69 |
| High-density lipoprotein | rs407133 | G | C | 0.0105516 | 0.441 | 0.0019326 | 4.80E-08 | 7.38E-05 | 29.81 |
| High-density lipoprotein | rs4078216 | G | A | -0.0139898 | 0.76 | 0.0022304 | 3.60E-10 | 9.74E-05 | 39.34 |
| High-density lipoprotein | rs42125 | A | G | 0.0398455 | 0.977 | 0.0070056 | 1.30E-08 | 8.01E-05 | 32.35 |
| High-density lipoprotein | rs4239651 | T | C | -0.0259714 | 0.206 | 0.0023605 | 3.70E-28 | 3.00E-04 | 121.05 |
| High-density lipoprotein | rs429358 | T | C | 0.0754686 | 0.846 | 0.002639 | 7.20E-180 | 2.02E-03 | 817.81 |
| High-density lipoprotein | rs4356188 | C | G | 0.0157423 | 0.299 | 0.0020804 | 3.80E-14 | 1.42E-04 | 57.26 |
| High-density lipoprotein | rs454968 | T | C | -0.0109508 | 0.355 | 0.0019949 | 4.00E-08 | 7.46E-05 | 30.13 |
| High-density lipoprotein | rs4650994 | G | A | 0.0180675 | 0.468 | 0.0019008 | 2.00E-21 | 2.24E-04 | 90.35 |
| High-density lipoprotein | rs4686739 | A | G | -0.0167646 | 0.366 | 0.0019833 | 2.80E-17 | 1.77E-04 | 71.45 |
| High-density lipoprotein | rs4691379 | C | T | -0.0118813 | 0.681 | 0.0020435 | 6.10E-09 | 8.37E-05 | 33.81 |
| High-density lipoprotein | rs4731701 | C | T | -0.029758 | 0.507 | 0.0019103 | 1.00E-54 | 6.00E-04 | 242.65 |
| High-density lipoprotein | rs4755720 | C | T | -0.0110921 | 0.393 | 0.0019556 | 1.40E-08 | 7.96E-05 | 32.17 |
| High-density lipoprotein | rs4855582 | C | T | -0.0110991 | 0.57 | 0.0019239 | 8.00E-09 | 8.24E-05 | 33.28 |
| High-density lipoprotein | rs4871603 | C | T | -0.0360442 | 0.347 | 0.0019925 | 3.80E-73 | 8.09E-04 | 327.25 |
| High-density lipoprotein | rs4871624 | T | G | 0.0204064 | 0.713 | 0.0021105 | 4.10E-22 | 2.31E-04 | 93.49 |
| High-density lipoprotein | rs4875043 | A | C | 0.0149835 | 0.783 | 0.0023287 | 1.20E-10 | 1.02E-04 | 41.40 |
| High-density lipoprotein | rs4930352 | G | T | -0.0161324 | 0.506 | 0.0019387 | 8.70E-17 | 1.71E-04 | 69.24 |
| High-density lipoprotein | rs4947121 | T | C | 0.0141734 | 0.226 | 0.0022763 | 4.80E-10 | 9.60E-05 | 38.77 |
| High-density lipoprotein | rs4969141 | C | T | -0.0296327 | 0.51 | 0.0019117 | 3.40E-54 | 5.94E-04 | 240.26 |
| High-density lipoprotein | rs549058 | G | T | -0.0171598 | 0.88 | 0.0029373 | 5.20E-09 | 8.45E-05 | 34.13 |
| High-density lipoprotein | rs55781197 | A | G | -0.0588019 | 0.885 | 0.0029517 | 2.70E-88 | 9.82E-04 | 396.86 |
| High-density lipoprotein | rs557933 | A | C | -0.0153071 | 0.48 | 0.001908 | 1.00E-15 | 1.59E-04 | 64.36 |
| High-density lipoprotein | rs55935382 | C | A | -0.0176192 | 0.675 | 0.0020385 | 5.50E-18 | 1.85E-04 | 74.71 |
| High-density lipoprotein | rs559355 | A | T | 0.0349543 | 0.843 | 0.0026128 | 8.10E-41 | 4.43E-04 | 178.97 |
| High-density lipoprotein | rs57074291 | C | G | -0.0131032 | 0.739 | 0.0021655 | 1.40E-09 | 9.06E-05 | 36.61 |
| High-density lipoprotein | rs58298943 | C | T | -0.0201854 | 0.916 | 0.0034279 | 3.90E-09 | 8.58E-05 | 34.68 |
| High-density lipoprotein | rs59104589 | C | T | -0.0150125 | 0.642 | 0.0019818 | 3.60E-14 | 1.42E-04 | 57.38 |
| High-density lipoprotein | rs6002946 | G | T | -0.0116715 | 0.323 | 0.0020533 | 1.30E-08 | 8.00E-05 | 32.31 |
| High-density lipoprotein | rs6073958 | T | C | 0.0608935 | 0.801 | 0.0023923 | 6.50E-143 | 1.60E-03 | 647.88 |
| High-density lipoprotein | rs6123685 | G | A | -0.0159703 | 0.745 | 0.0021914 | 3.10E-13 | 1.31E-04 | 53.11 |
| High-density lipoprotein | rs6131012 | A | G | -0.011299 | 0.603 | 0.0019695 | 9.60E-09 | 8.15E-05 | 32.91 |
| High-density lipoprotein | rs61352607 | G | T | -0.0305532 | 0.759 | 0.0022247 | 6.40E-43 | 4.67E-04 | 188.62 |
| High-density lipoprotein | rs61435086 | T | C | -0.0892037 | 0.988 | 0.0086222 | 4.40E-25 | 2.65E-04 | 107.03 |
| High-density lipoprotein | rs61596977 | C | T | 0.0161666 | 0.86 | 0.0027375 | 3.50E-09 | 8.63E-05 | 34.88 |
| High-density lipoprotein | rs61748951 | C | A | 0.0359832 | 0.976 | 0.0064095 | 2.00E-08 | 7.80E-05 | 31.52 |
| High-density lipoprotein | rs61805075 | G | A | 0.0256926 | 0.671 | 0.0020255 | 7.20E-37 | 3.98E-04 | 160.89 |
| High-density lipoprotein | rs61884030 | T | C | -0.0177467 | 0.861 | 0.0027597 | 1.30E-10 | 1.02E-04 | 41.35 |
| High-density lipoprotein | rs62102718 | A | T | 0.023726 | 0.714 | 0.0021128 | 2.90E-29 | 3.12E-04 | 126.11 |
| High-density lipoprotein | rs62271373 | T | A | 0.0407194 | 0.94 | 0.0040871 | 2.20E-23 | 2.46E-04 | 99.26 |
| High-density lipoprotein | rs62428831 | T | C | -0.0177819 | 0.858 | 0.0027557 | 1.10E-10 | 1.03E-04 | 41.64 |
| High-density lipoprotein | rs62492368 | G | A | 0.0171783 | 0.695 | 0.0020807 | 1.50E-16 | 1.69E-04 | 68.16 |
| High-density lipoprotein | rs632057 | T | G | -0.0196758 | 0.372 | 0.001969 | 1.60E-23 | 2.47E-04 | 99.86 |
| High-density lipoprotein | rs6469605 | C | T | -0.0317165 | 0.432 | 0.0019198 | 2.60E-61 | 6.75E-04 | 272.94 |
| High-density lipoprotein | rs6542680 | C | T | 0.0249275 | 0.18 | 0.0024966 | 1.80E-23 | 2.47E-04 | 99.69 |
| High-density lipoprotein | rs6590207 | A | G | 0.0148093 | 0.709 | 0.0021709 | 9.00E-12 | 1.15E-04 | 46.54 |
| High-density lipoprotein | rs676210 | G | A | -0.0592929 | 0.795 | 0.0023526 | 3.70E-140 | 1.57E-03 | 635.20 |
| High-density lipoprotein | rs6762415 | T | G | 0.010907 | 0.465 | 0.0019136 | 1.20E-08 | 8.04E-05 | 32.49 |
| High-density lipoprotein | rs6765484 | C | T | -0.0224418 | 0.527 | 0.0019088 | 6.50E-32 | 3.42E-04 | 138.22 |
| High-density lipoprotein | rs680321 | T | C | -0.0108871 | 0.543 | 0.0019168 | 1.30E-08 | 7.99E-05 | 32.26 |
| High-density lipoprotein | rs6824451 | G | A | 0.0200543 | 0.536 | 0.0019119 | 9.70E-26 | 2.72E-04 | 110.02 |
| High-density lipoprotein | rs686030 | C | A | -0.0497684 | 0.141 | 0.0027398 | 9.80E-74 | 8.16E-04 | 329.97 |
| High-density lipoprotein | rs6934962 | C | T | -0.016256 | 0.598 | 0.0019412 | 5.60E-17 | 1.74E-04 | 70.13 |
| High-density lipoprotein | rs6940493 | A | T | -0.0120893 | 0.315 | 0.0020677 | 5.00E-09 | 8.46E-05 | 34.18 |
| High-density lipoprotein | rs703966 | G | A | -0.0156745 | 0.581 | 0.0019348 | 5.40E-16 | 1.62E-04 | 65.63 |
| High-density lipoprotein | rs7136506 | T | C | 0.036204 | 0.784 | 0.0023521 | 1.80E-53 | 5.86E-04 | 236.92 |
| High-density lipoprotein | rs7158166 | T | C | -0.0140645 | 0.406 | 0.0019513 | 5.70E-13 | 1.29E-04 | 51.95 |
| High-density lipoprotein | rs7170463 | A | G | -0.0188509 | 0.689 | 0.0020593 | 5.50E-20 | 2.07E-04 | 83.79 |
| High-density lipoprotein | rs7188071 | T | C | -0.0109104 | 0.353 | 0.0019711 | 3.10E-08 | 7.58E-05 | 30.64 |
| High-density lipoprotein | rs7218647 | G | A | -0.0108574 | 0.441 | 0.0019263 | 1.70E-08 | 7.86E-05 | 31.77 |
| High-density lipoprotein | rs7251640 | T | C | -0.014263 | 0.806 | 0.0024284 | 4.30E-09 | 8.54E-05 | 34.50 |
| High-density lipoprotein | rs72647336 | G | A | 0.0441541 | 0.943 | 0.0044288 | 2.10E-23 | 2.46E-04 | 99.40 |
| High-density lipoprotein | rs72801474 | G | A | -0.0212955 | 0.908 | 0.0033027 | 1.10E-10 | 1.03E-04 | 41.58 |
| High-density lipoprotein | rs72836561 | C | T | 0.170535 | 0.969 | 0.0054829 | 1.00E-200 | 2.39E-03 | 967.41 |
| High-density lipoprotein | rs72926946 | C | A | 0.0207168 | 0.704 | 0.0020806 | 2.30E-23 | 2.45E-04 | 99.14 |
| High-density lipoprotein | rs72959041 | G | A | 0.0474304 | 0.951 | 0.0044659 | 2.40E-26 | 2.79E-04 | 112.80 |
| High-density lipoprotein | rs73151974 | C | T | 0.0161097 | 0.856 | 0.002734 | 3.80E-09 | 8.59E-05 | 34.72 |
| High-density lipoprotein | rs73243877 | A | G | 0.0253996 | 0.832 | 0.0025494 | 2.20E-23 | 2.46E-04 | 99.26 |
| High-density lipoprotein | rs73455693 | G | A | -0.0281128 | 0.963 | 0.005106 | 3.70E-08 | 7.50E-05 | 30.31 |
| High-density lipoprotein | rs74456742 | G | A | -0.0361479 | 0.962 | 0.0051475 | 2.20E-12 | 1.22E-04 | 49.31 |
| High-density lipoprotein | rs74500135 | T | C | -0.0670791 | 0.99 | 0.0100302 | 2.30E-11 | 1.11E-04 | 44.73 |
| High-density lipoprotein | rs7488780 | G | C | -0.0145424 | 0.796 | 0.0023713 | 8.60E-10 | 9.31E-05 | 37.61 |
| High-density lipoprotein | rs75032664 | C | G | 0.0549281 | 0.987 | 0.009089 | 1.50E-09 | 9.04E-05 | 36.52 |
| High-density lipoprotein | rs75152587 | G | T | 0.0944212 | 0.987 | 0.0085 | 1.10E-28 | 3.05E-04 | 123.40 |
| High-density lipoprotein | rs75246752 | G | C | -0.0490747 | 0.987 | 0.0084172 | 5.50E-09 | 8.41E-05 | 33.99 |
| High-density lipoprotein | rs75479205 | A | G | -0.0139462 | 0.811 | 0.0024367 | 1.00E-08 | 8.11E-05 | 32.76 |
| High-density lipoprotein | rs75572195 | G | A | -0.0225924 | 0.906 | 0.003267 | 4.70E-12 | 1.18E-04 | 47.82 |
| High-density lipoprotein | rs75609851 | G | A | -0.170013 | 0.99 | 0.0096477 | 1.70E-69 | 7.68E-04 | 310.54 |
| High-density lipoprotein | rs75662196 | G | C | -0.067763 | 0.972 | 0.0058731 | 8.50E-31 | 3.29E-04 | 133.12 |
| High-density lipoprotein | rs7583067 | C | T | -0.0145605 | 0.76 | 0.0022356 | 7.40E-11 | 1.05E-04 | 42.42 |
| High-density lipoprotein | rs76213248 | C | T | -0.0133361 | 0.59 | 0.0019444 | 6.90E-12 | 1.16E-04 | 47.04 |
| High-density lipoprotein | rs7622114 | C | A | -0.0115403 | 0.419 | 0.0019402 | 2.70E-09 | 8.76E-05 | 35.38 |
| High-density lipoprotein | rs76428106 | T | C | 0.0620148 | 0.987 | 0.0087903 | 1.70E-12 | 1.23E-04 | 49.77 |
| High-density lipoprotein | rs76602912 | T | C | 0.04341 | 0.976 | 0.0062644 | 4.20E-12 | 1.19E-04 | 48.02 |
| High-density lipoprotein | rs771481 | T | A | -0.0288308 | 0.816 | 0.0024571 | 8.60E-32 | 3.41E-04 | 137.68 |
| High-density lipoprotein | rs7725218 | G | A | 0.012181 | 0.662 | 0.0020146 | 1.50E-09 | 9.05E-05 | 36.56 |
| High-density lipoprotein | rs7750688 | C | T | -0.0214656 | 0.243 | 0.002221 | 4.30E-22 | 2.31E-04 | 93.41 |
| High-density lipoprotein | rs7757193 | G | A | 0.0190593 | 0.635 | 0.0019843 | 7.60E-22 | 2.28E-04 | 92.25 |
| High-density lipoprotein | rs77605964 | G | A | -0.0170398 | 0.773 | 0.0022785 | 7.50E-14 | 1.38E-04 | 55.93 |
| High-density lipoprotein | rs7769059 | G | A | 0.0150833 | 0.717 | 0.0021209 | 1.10E-12 | 1.25E-04 | 50.57 |
| High-density lipoprotein | rs77960347 | A | G | -0.291004 | 0.987 | 0.0083025 | 1.00E-200 | 3.03E-03 | 1228.51 |
| High-density lipoprotein | rs78058190 | G | A | 0.0784127 | 0.95 | 0.0049049 | 1.60E-57 | 6.32E-04 | 255.57 |
| High-density lipoprotein | rs7817574 | T | C | -0.0331636 | 0.815 | 0.0024397 | 4.40E-42 | 4.57E-04 | 184.78 |
| High-density lipoprotein | rs7826177 | T | C | -0.0112826 | 0.365 | 0.0019794 | 1.20E-08 | 8.04E-05 | 32.49 |
| High-density lipoprotein | rs78456138 | C | T | -0.0346587 | 0.976 | 0.0062845 | 3.50E-08 | 7.53E-05 | 30.42 |
| High-density lipoprotein | rs79153732 | C | T | 0.0938573 | 0.983 | 0.0072832 | 5.30E-38 | 4.11E-04 | 166.07 |
| High-density lipoprotein | rs79600951 | C | G | 0.106785 | 0.908 | 0.0032594 | 1.00E-200 | 2.65E-03 | 1073.35 |
| High-density lipoprotein | rs79616633 | C | T | 0.0358494 | 0.978 | 0.0064003 | 2.10E-08 | 7.77E-05 | 31.37 |
| High-density lipoprotein | rs79634051 | G | C | -0.0390728 | 0.972 | 0.0057495 | 1.10E-11 | 1.14E-04 | 46.18 |
| High-density lipoprotein | rs80005209 | T | G | 0.144058 | 0.97 | 0.0056374 | 5.00E-144 | 1.61E-03 | 653.00 |
| High-density lipoprotein | rs8014289 | A | G | -0.0150509 | 0.438 | 0.0019273 | 5.80E-15 | 1.51E-04 | 60.98 |
| High-density lipoprotein | rs8081548 | T | A | -0.0184231 | 0.341 | 0.0020212 | 7.90E-20 | 2.06E-04 | 83.08 |
| High-density lipoprotein | rs8086351 | C | G | -0.0838881 | 0.176 | 0.0025071 | 1.00E-200 | 2.76E-03 | 1119.62 |
| High-density lipoprotein | rs830620 | C | T | -0.0149922 | 0.585 | 0.001931 | 8.20E-15 | 1.49E-04 | 60.28 |
| High-density lipoprotein | rs880674 | T | C | -0.0150058 | 0.858 | 0.002738 | 4.20E-08 | 7.44E-05 | 30.04 |
| High-density lipoprotein | rs907866 | G | A | 0.0182898 | 0.555 | 0.001919 | 1.60E-21 | 2.25E-04 | 90.84 |
| High-density lipoprotein | rs921919 | G | A | 0.0415939 | 0.331 | 0.0020648 | 3.00E-90 | 1.00E-03 | 405.81 |
| High-density lipoprotein | rs9604045 | G | T | -0.0175533 | 0.746 | 0.0022894 | 1.80E-14 | 1.46E-04 | 58.79 |
| High-density lipoprotein | rs9622830 | C | G | 0.0162347 | 0.646 | 0.0019999 | 4.70E-16 | 1.63E-04 | 65.90 |
| High-density lipoprotein | rs964184 | G | C | -0.105482 | 0.134 | 0.0027949 | 1.00E-200 | 3.51E-03 | 1424.39 |
| High-density lipoprotein | rs9647335 | A | T | -0.0277611 | 0.808 | 0.002425 | 2.40E-30 | 3.24E-04 | 131.06 |
| High-density lipoprotein | rs968050 | C | T | -0.0135416 | 0.518 | 0.0019083 | 1.30E-12 | 1.25E-04 | 50.36 |
| High-density lipoprotein | rs9877304 | G | A | 0.0135927 | 0.737 | 0.0021738 | 4.00E-10 | 9.68E-05 | 39.10 |
| High-density lipoprotein | rs998584 | C | A | 0.0340915 | 0.517 | 0.0019099 | 2.90E-71 | 7.88E-04 | 318.60 |
| High-density lipoprotein | rs9987289 | A | G | -0.08734 | 0.091 | 0.003294 | 6.60E-155 | 1.74E-03 | 703.02 |
| High-density lipoprotein | rs9989419 | A | G | -0.143745 | 0.394 | 0.0019265 | 1.00E-200 | 1.36E-02 | 5567.19 |
| Low-density lipoprotein | rs1007938 | A | G | -0.0121506 | 0.595 | 0.002135 | 1.30E-08 | 7.35E-05 | 32.39 |
| Low-density lipoprotein | rs10231941 | T | C | -0.0198963 | 0.822 | 0.0027283 | 3.00E-13 | 1.21E-04 | 53.18 |
| Low-density lipoprotein | rs10448340 | T | G | 0.0151234 | 0.68 | 0.0022384 | 1.40E-11 | 1.04E-04 | 45.65 |
| Low-density lipoprotein | rs10832963 | T | G | -0.0173852 | 0.256 | 0.0023973 | 4.10E-13 | 1.19E-04 | 52.59 |
| Low-density lipoprotein | rs10910476 | C | T | -0.0123459 | 0.445 | 0.0021029 | 4.30E-09 | 7.82E-05 | 34.47 |
| Low-density lipoprotein | rs11014204 | C | T | -0.0137929 | 0.72 | 0.0023311 | 3.30E-09 | 7.95E-05 | 35.01 |
| Low-density lipoprotein | rs11206517 | T | G | -0.0683407 | 0.967 | 0.0058087 | 5.90E-32 | 3.14E-04 | 138.42 |
| Low-density lipoprotein | rs113177823 | G | A | 0.0410816 | 0.946 | 0.0046626 | 1.20E-18 | 1.76E-04 | 77.63 |
| Low-density lipoprotein | rs114165349 | G | C | -0.0560406 | 0.977 | 0.0069585 | 8.00E-16 | 1.47E-04 | 64.86 |
| Low-density lipoprotein | rs115458560 | T | C | 0.0503283 | 0.981 | 0.0077083 | 6.60E-11 | 9.68E-05 | 42.63 |
| Low-density lipoprotein | rs115478735 | A | T | -0.0558766 | 0.816 | 0.0026909 | 9.00E-96 | 9.78E-04 | 431.18 |
| Low-density lipoprotein | rs11568318 | C | A | -0.0257407 | 0.934 | 0.0041804 | 7.40E-10 | 8.61E-05 | 37.91 |
| Low-density lipoprotein | rs11591147 | G | T | 0.348511 | 0.983 | 0.0079346 | 1.00E-200 | 4.36E-03 | 1929.20 |
| Low-density lipoprotein | rs11601507 | C | A | -0.0322609 | 0.931 | 0.0040517 | 1.70E-15 | 1.44E-04 | 63.40 |
| Low-density lipoprotein | rs11621792 | C | T | -0.0191305 | 0.547 | 0.0021113 | 1.30E-19 | 1.86E-04 | 82.11 |
| Low-density lipoprotein | rs116734477 | C | T | 0.0474419 | 0.959 | 0.0052843 | 2.80E-19 | 1.83E-04 | 80.60 |
| Low-density lipoprotein | rs1169294 | G | A | -0.0245145 | 0.69 | 0.0022595 | 2.00E-27 | 2.67E-04 | 117.72 |
| Low-density lipoprotein | rs117139027 | G | A | 0.0569281 | 0.982 | 0.0079336 | 7.20E-13 | 1.17E-04 | 51.49 |
| Low-density lipoprotein | rs117733303 | A | G | -0.0838432 | 0.982 | 0.0077418 | 2.50E-27 | 2.66E-04 | 117.29 |
| Low-density lipoprotein | rs11786083 | G | A | -0.0155482 | 0.626 | 0.0021664 | 7.10E-13 | 1.17E-04 | 51.51 |
| Low-density lipoprotein | rs11789603 | C | T | -0.0249753 | 0.892 | 0.0033679 | 1.20E-13 | 1.25E-04 | 54.99 |
| Low-density lipoprotein | rs118039278 | G | A | -0.083508 | 0.921 | 0.0038871 | 2.20E-102 | 1.05E-03 | 461.53 |
| Low-density lipoprotein | rs12162782 | T | G | -0.012882 | 0.656 | 0.0021945 | 4.40E-09 | 7.82E-05 | 34.46 |
| Low-density lipoprotein | rs12208357 | C | T | -0.057141 | 0.93 | 0.004098 | 3.40E-44 | 4.41E-04 | 194.42 |
| Low-density lipoprotein | rs12246352 | A | G | -0.025694 | 0.896 | 0.0034294 | 6.80E-14 | 1.27E-04 | 56.13 |
| Low-density lipoprotein | rs1229984 | T | C | -0.0528485 | 0.027 | 0.0063365 | 7.40E-17 | 1.58E-04 | 69.56 |
| Low-density lipoprotein | rs12445804 | G | A | -0.0231166 | 0.925 | 0.004007 | 8.00E-09 | 7.55E-05 | 33.28 |
| Low-density lipoprotein | rs12471768 | T | C | -0.0136102 | 0.296 | 0.0022822 | 2.50E-09 | 8.07E-05 | 35.57 |
| Low-density lipoprotein | rs1250258 | C | T | -0.0137293 | 0.263 | 0.0023688 | 6.80E-09 | 7.62E-05 | 33.59 |
| Low-density lipoprotein | rs12603885 | G | A | -0.0175036 | 0.3 | 0.0022741 | 1.40E-14 | 1.34E-04 | 59.24 |
| Low-density lipoprotein | rs12916 | T | C | -0.0621277 | 0.599 | 0.002128 | 2.20E-187 | 1.93E-03 | 852.36 |
| Low-density lipoprotein | rs13076933 | T | G | 0.0208296 | 0.741 | 0.0023914 | 3.00E-18 | 1.72E-04 | 75.87 |
| Low-density lipoprotein | rs13107325 | C | T | 0.0245488 | 0.925 | 0.003966 | 6.00E-10 | 8.70E-05 | 38.31 |
| Low-density lipoprotein | rs13108218 | A | G | 0.0177573 | 0.385 | 0.0021608 | 2.10E-16 | 1.53E-04 | 67.54 |
| Low-density lipoprotein | rs1350559 | C | G | -0.0136979 | 0.599 | 0.0021378 | 1.50E-10 | 9.32E-05 | 41.06 |
| Low-density lipoprotein | rs143020224 | C | G | 0.169039 | 0.881 | 0.0032098 | 1.00E-200 | 6.26E-03 | 2773.35 |
| Low-density lipoprotein | rs145730801 | T | C | -0.0359582 | 0.956 | 0.0051715 | 3.60E-12 | 1.10E-04 | 48.35 |
| Low-density lipoprotein | rs1458038 | C | T | 0.0185934 | 0.709 | 0.0023036 | 6.90E-16 | 1.48E-04 | 65.15 |
| Low-density lipoprotein | rs146433259 | C | T | 0.0567916 | 0.988 | 0.0103876 | 4.60E-08 | 6.78E-05 | 29.89 |
| Low-density lipoprotein | rs146534110 | G | T | -0.0679152 | 0.987 | 0.0091046 | 8.70E-14 | 1.26E-04 | 55.64 |
| Low-density lipoprotein | rs150474434 | G | A | 0.0347466 | 0.899 | 0.0034722 | 1.40E-23 | 2.27E-04 | 100.14 |
| Low-density lipoprotein | rs1532085 | A | G | 0.017242 | 0.386 | 0.0021443 | 8.90E-16 | 1.47E-04 | 64.66 |
| Low-density lipoprotein | rs1551891 | G | A | 0.173541 | 0.912 | 0.003662 | 1.00E-200 | 5.07E-03 | 2245.75 |
| Low-density lipoprotein | rs1556562 | G | T | -0.0190535 | 0.21 | 0.0024902 | 2.00E-14 | 1.33E-04 | 58.54 |
| Low-density lipoprotein | rs17050272 | G | A | 0.0205207 | 0.591 | 0.0021165 | 3.10E-22 | 2.13E-04 | 94.01 |
| Low-density lipoprotein | rs174564 | A | G | 0.0319674 | 0.651 | 0.0021906 | 3.10E-48 | 4.83E-04 | 212.95 |
| Low-density lipoprotein | rs17476364 | T | C | 0.0217849 | 0.892 | 0.0033608 | 9.10E-11 | 9.54E-05 | 42.02 |
| Low-density lipoprotein | rs1800961 | C | T | 0.0596747 | 0.969 | 0.006026 | 4.00E-23 | 2.23E-04 | 98.07 |
| Low-density lipoprotein | rs1801689 | A | C | -0.0619438 | 0.969 | 0.0060607 | 1.60E-24 | 2.37E-04 | 104.46 |
| Low-density lipoprotein | rs1883711 | G | C | -0.102599 | 0.969 | 0.0060947 | 1.40E-63 | 6.43E-04 | 283.39 |
| Low-density lipoprotein | rs204469 | A | G | -0.0294656 | 0.044 | 0.0051188 | 8.60E-09 | 7.52E-05 | 33.14 |
| Low-density lipoprotein | rs2066714 | T | C | -0.0209673 | 0.871 | 0.0031111 | 1.60E-11 | 1.03E-04 | 45.42 |
| Low-density lipoprotein | rs2068888 | G | A | 0.0191878 | 0.549 | 0.0020951 | 5.30E-20 | 1.90E-04 | 83.87 |
| Low-density lipoprotein | rs2073547 | A | G | -0.0355544 | 0.816 | 0.0026741 | 2.40E-40 | 4.01E-04 | 176.78 |
| Low-density lipoprotein | rs2160994 | T | C | -0.0183788 | 0.353 | 0.0021851 | 4.10E-17 | 1.61E-04 | 70.74 |
| Low-density lipoprotein | rs2238162 | C | T | 0.0164506 | 0.477 | 0.0020894 | 3.50E-15 | 1.41E-04 | 61.99 |
| Low-density lipoprotein | rs2250802 | G | A | 0.0182094 | 0.276 | 0.0023333 | 6.00E-15 | 1.38E-04 | 60.90 |
| Low-density lipoprotein | rs2256814 | G | A | -0.0152271 | 0.802 | 0.0026263 | 6.70E-09 | 7.63E-05 | 33.62 |
| Low-density lipoprotein | rs2287622 | A | G | 0.0211456 | 0.397 | 0.0021303 | 3.20E-23 | 2.24E-04 | 98.53 |
| Low-density lipoprotein | rs261334 | G | C | 0.0218856 | 0.213 | 0.0025477 | 8.70E-18 | 1.67E-04 | 73.79 |
| Low-density lipoprotein | rs2618566 | G | T | 0.0248873 | 0.34 | 0.0022009 | 1.20E-29 | 2.90E-04 | 127.86 |
| Low-density lipoprotein | rs2642438 | A | G | -0.0253446 | 0.297 | 0.0022741 | 7.60E-29 | 2.82E-04 | 124.21 |
| Low-density lipoprotein | rs2737265 | A | G | 0.0203604 | 0.72 | 0.002323 | 1.90E-18 | 1.74E-04 | 76.82 |
| Low-density lipoprotein | rs2738447 | A | C | -0.0422733 | 0.407 | 0.0021112 | 3.50E-89 | 9.09E-04 | 400.92 |
| Low-density lipoprotein | rs2740488 | A | C | 0.0253762 | 0.735 | 0.0023661 | 7.80E-27 | 2.61E-04 | 115.03 |
| Low-density lipoprotein | rs28406917 | C | T | -0.0119167 | 0.572 | 0.0021153 | 1.80E-08 | 7.20E-05 | 31.74 |
| Low-density lipoprotein | rs28601761 | C | G | 0.0619374 | 0.581 | 0.0021372 | 1.20E-184 | 1.90E-03 | 839.87 |
| Low-density lipoprotein | rs28631087 | T | C | 0.0161715 | 0.787 | 0.0025471 | 2.20E-10 | 9.15E-05 | 40.31 |
| Low-density lipoprotein | rs28768427 | G | A | -0.0122459 | 0.48 | 0.0021517 | 1.30E-08 | 7.35E-05 | 32.39 |
| Low-density lipoprotein | rs34042070 | C | G | -0.0485842 | 0.812 | 0.0026813 | 2.20E-73 | 7.45E-04 | 328.32 |
| Low-density lipoprotein | rs35081008 | C | T | 0.0319653 | 0.852 | 0.0029304 | 1.10E-27 | 2.70E-04 | 118.99 |
| Low-density lipoprotein | rs35882350 | A | G | -0.0138882 | 0.739 | 0.0023752 | 5.00E-09 | 7.76E-05 | 34.19 |
| Low-density lipoprotein | rs3732359 | G | A | 0.0174313 | 0.22 | 0.0025215 | 4.70E-12 | 1.08E-04 | 47.79 |
| Low-density lipoprotein | rs3764261 | C | A | 0.0330572 | 0.676 | 0.0022299 | 1.00E-49 | 4.99E-04 | 219.76 |
| Low-density lipoprotein | rs3780181 | A | G | 0.0278415 | 0.932 | 0.0041851 | 2.90E-11 | 1.00E-04 | 44.26 |
| Low-density lipoprotein | rs3822855 | G | T | -0.017915 | 0.598 | 0.0021254 | 3.50E-17 | 1.61E-04 | 71.05 |
| Low-density lipoprotein | rs4148826 | T | C | 0.0158111 | 0.82 | 0.0027245 | 6.50E-09 | 7.64E-05 | 33.68 |
| Low-density lipoprotein | rs4299376 | G | T | 0.0542425 | 0.323 | 0.0022257 | 3.50E-131 | 1.35E-03 | 593.94 |
| Low-density lipoprotein | rs430096 | A | G | 0.0542983 | 0.779 | 0.0025153 | 2.30E-103 | 1.06E-03 | 466.02 |
| Low-density lipoprotein | rs438568 | A | G | -0.0125944 | 0.391 | 0.0021408 | 4.00E-09 | 7.86E-05 | 34.61 |
| Low-density lipoprotein | rs4452060 | C | A | -0.0833597 | 0.579 | 0.0021052 | 1.00E-200 | 3.55E-03 | 1567.89 |
| Low-density lipoprotein | rs472495 | G | T | -0.0426246 | 0.351 | 0.0021819 | 5.50E-85 | 8.66E-04 | 381.62 |
| Low-density lipoprotein | rs4782568 | C | G | 0.0164593 | 0.549 | 0.002109 | 6.00E-15 | 1.38E-04 | 60.91 |
| Low-density lipoprotein | rs4954192 | C | T | -0.0147212 | 0.627 | 0.0021375 | 5.70E-12 | 1.08E-04 | 47.43 |
| Low-density lipoprotein | rs4970834 | C | T | 0.105228 | 0.813 | 0.0026831 | 1.00E-200 | 3.48E-03 | 1538.17 |
| Low-density lipoprotein | rs556107 | C | T | -0.0352397 | 0.477 | 0.0020894 | 8.00E-64 | 6.45E-04 | 284.45 |
| Low-density lipoprotein | rs55714927 | C | T | 0.0263355 | 0.81 | 0.0026567 | 3.70E-23 | 2.23E-04 | 98.26 |
| Low-density lipoprotein | rs55921103 | G | T | -0.0136729 | 0.351 | 0.0021993 | 5.10E-10 | 8.77E-05 | 38.65 |
| Low-density lipoprotein | rs56130071 | G | C | -0.0331915 | 0.783 | 0.0025391 | 4.70E-39 | 3.88E-04 | 170.88 |
| Low-density lipoprotein | rs56236159 | T | G | -0.01784 | 0.869 | 0.0030812 | 7.00E-09 | 7.61E-05 | 33.52 |
| Low-density lipoprotein | rs597808 | A | G | -0.0271233 | 0.484 | 0.0020924 | 2.00E-38 | 3.81E-04 | 168.03 |
| Low-density lipoprotein | rs6050463 | G | A | -0.0125353 | 0.507 | 0.0020846 | 1.80E-09 | 8.21E-05 | 36.16 |
| Low-density lipoprotein | rs6093446 | G | A | -0.022143 | 0.712 | 0.0023047 | 7.40E-22 | 2.09E-04 | 92.31 |
| Low-density lipoprotein | rs61754230 | C | T | -0.0427126 | 0.98 | 0.0074878 | 1.20E-08 | 7.39E-05 | 32.54 |
| Low-density lipoprotein | rs61775180 | C | T | 0.0262622 | 0.58 | 0.0021118 | 1.70E-35 | 3.51E-04 | 154.65 |
| Low-density lipoprotein | rs61988556 | T | C | 0.0222954 | 0.914 | 0.0037226 | 2.10E-09 | 8.14E-05 | 35.87 |
| Low-density lipoprotein | rs62033400 | A | G | 0.0144912 | 0.605 | 0.0021354 | 1.20E-11 | 1.05E-04 | 46.05 |
| Low-density lipoprotein | rs62116889 | T | C | 0.04951 | 0.932 | 0.0041142 | 2.40E-33 | 3.29E-04 | 144.81 |
| Low-density lipoprotein | rs62118464 | G | A | -0.0276497 | 0.882 | 0.0032957 | 4.90E-17 | 1.60E-04 | 70.39 |
| Low-density lipoprotein | rs6475606 | C | T | 0.02031 | 0.516 | 0.0020846 | 2.00E-22 | 2.15E-04 | 94.92 |
| Low-density lipoprotein | rs6560499 | G | A | 0.0122425 | 0.424 | 0.0021229 | 8.10E-09 | 7.55E-05 | 33.26 |
| Low-density lipoprotein | rs6573971 | G | A | 0.0133161 | 0.445 | 0.0021208 | 3.40E-10 | 8.95E-05 | 39.42 |
| Low-density lipoprotein | rs6602912 | T | G | -0.0222758 | 0.715 | 0.002311 | 5.50E-22 | 2.11E-04 | 92.91 |
| Low-density lipoprotein | rs6693893 | T | C | 0.0749332 | 0.965 | 0.0056434 | 3.10E-40 | 4.00E-04 | 176.30 |
| Low-density lipoprotein | rs6709904 | A | G | 0.0435284 | 0.887 | 0.0032975 | 8.70E-40 | 3.95E-04 | 174.25 |
| Low-density lipoprotein | rs6857 | C | T | -0.157677 | 0.83 | 0.0027594 | 1.00E-200 | 7.36E-03 | 3265.14 |
| Low-density lipoprotein | rs6874202 | T | C | -0.0323831 | 0.366 | 0.0021639 | 1.20E-50 | 5.08E-04 | 223.95 |
| Low-density lipoprotein | rs7108486 | T | C | 0.0389569 | 0.976 | 0.0069234 | 1.80E-08 | 7.19E-05 | 31.66 |
| Low-density lipoprotein | rs71311871 | A | G | 0.0283771 | 0.917 | 0.0037863 | 6.60E-14 | 1.27E-04 | 56.17 |
| Low-density lipoprotein | rs7202323 | T | G | 0.0255122 | 0.77 | 0.0024784 | 7.50E-25 | 2.40E-04 | 105.96 |
| Low-density lipoprotein | rs7241918 | G | T | -0.0156669 | 0.176 | 0.0027506 | 1.20E-08 | 7.36E-05 | 32.44 |
| Low-density lipoprotein | rs72631343 | C | G | 0.0292878 | 0.871 | 0.0031117 | 4.90E-21 | 2.01E-04 | 88.59 |
| Low-density lipoprotein | rs72638977 | A | G | 0.0328423 | 0.966 | 0.0057795 | 1.30E-08 | 7.33E-05 | 32.29 |
| Low-density lipoprotein | rs73025516 | A | G | 0.0309443 | 0.955 | 0.0050545 | 9.20E-10 | 8.51E-05 | 37.48 |
| Low-density lipoprotein | rs7734476 | G | A | -0.0187267 | 0.45 | 0.002095 | 3.90E-19 | 1.81E-04 | 79.90 |
| Low-density lipoprotein | rs7746081 | G | A | 0.0234538 | 0.696 | 0.002268 | 4.60E-25 | 2.43E-04 | 106.94 |
| Low-density lipoprotein | rs77542162 | A | G | -0.128166 | 0.978 | 0.0070433 | 5.50E-74 | 7.51E-04 | 331.12 |
| Low-density lipoprotein | rs7776054 | A | G | 0.0162915 | 0.739 | 0.002376 | 7.00E-12 | 1.07E-04 | 47.01 |
| Low-density lipoprotein | rs77960347 | A | G | -0.0706855 | 0.987 | 0.0090904 | 7.50E-15 | 1.37E-04 | 60.46 |
| Low-density lipoprotein | rs79220007 | T | C | 0.0574396 | 0.924 | 0.0039232 | 1.50E-48 | 4.86E-04 | 214.36 |
| Low-density lipoprotein | rs79828839 | C | T | -0.0145916 | 0.801 | 0.0026162 | 2.40E-08 | 7.06E-05 | 31.11 |
| Low-density lipoprotein | rs8107974 | A | T | 0.105172 | 0.924 | 0.0039211 | 1.80E-158 | 1.63E-03 | 719.43 |
| Low-density lipoprotein | rs836550 | A | G | -0.0116032 | 0.594 | 0.0021234 | 4.60E-08 | 6.78E-05 | 29.86 |
| Low-density lipoprotein | rs869412 | T | C | 0.0142708 | 0.774 | 0.0025021 | 1.20E-08 | 7.38E-05 | 32.53 |
| Low-density lipoprotein | rs880315 | T | C | 0.015148 | 0.66 | 0.0022078 | 6.80E-12 | 1.07E-04 | 47.07 |
| Low-density lipoprotein | rs907866 | G | A | 0.0176886 | 0.555 | 0.0021005 | 3.70E-17 | 1.61E-04 | 70.92 |
| Low-density lipoprotein | rs934197 | G | A | -0.0831886 | 0.665 | 0.002204 | 1.00E-200 | 3.22E-03 | 1424.62 |
| Low-density lipoprotein | rs9391803 | T | A | -0.0266262 | 0.852 | 0.0029453 | 1.60E-19 | 1.85E-04 | 81.72 |
| Low-density lipoprotein | rs9471975 | T | C | 0.012882 | 0.417 | 0.0021142 | 1.10E-09 | 8.43E-05 | 37.12 |
| Low-density lipoprotein | rs9496567 | G | A | 0.0175328 | 0.757 | 0.0024338 | 5.90E-13 | 1.18E-04 | 51.89 |
| Low-density lipoprotein | rs960596 | C | T | -0.0134522 | 0.661 | 0.0022186 | 1.30E-09 | 8.34E-05 | 36.77 |
| Low-density lipoprotein | rs964184 | G | C | 0.0574978 | 0.134 | 0.003065 | 1.60E-78 | 7.98E-04 | 351.93 |
| Low-density lipoprotein | rs9832727 | C | G | 0.0147771 | 0.66 | 0.0022049 | 2.10E-11 | 1.02E-04 | 44.92 |
| Low-density lipoprotein | rs9834932 | A | G | 0.0323458 | 0.911 | 0.0036658 | 1.10E-18 | 1.77E-04 | 77.86 |
| Low-density lipoprotein | rs9884390 | T | C | -0.0251131 | 0.766 | 0.0024927 | 7.20E-24 | 2.30E-04 | 101.50 |
| Low-density lipoprotein | rs9894946 | A | G | 0.0175495 | 0.159 | 0.0029026 | 1.50E-09 | 8.30E-05 | 36.56 |
| Low-density lipoprotein | rs990619 | C | G | 0.0117349 | 0.476 | 0.0020862 | 1.90E-08 | 7.18E-05 | 31.64 |
| Low-density lipoprotein | rs9929977 | T | A | -0.0167251 | 0.63 | 0.0021622 | 1.00E-14 | 1.36E-04 | 59.83 |
| Low-density lipoprotein | rs9987289 | A | G | -0.0454194 | 0.091 | 0.0036234 | 4.80E-36 | 3.57E-04 | 157.13 |
| Apolipoprotein A-1 | rs10023962 | T | G | -0.0175664 | 0.183 | 0.0025437 | 5.00E-12 | 1.21E-04 | 47.69 |
| Apolipoprotein A-1 | rs1037117 | G | A | -0.0160491 | 0.745 | 0.0022575 | 1.20E-12 | 1.29E-04 | 50.54 |
| Apolipoprotein A-1 | rs10458643 | G | A | 0.0108425 | 0.534 | 0.0019827 | 4.50E-08 | 7.61E-05 | 29.90 |
| Apolipoprotein A-1 | rs1047891 | C | A | 0.025611 | 0.684 | 0.0021058 | 4.90E-34 | 3.76E-04 | 147.92 |
| Apolipoprotein A-1 | rs10489044 | A | G | 0.0184304 | 0.801 | 0.002467 | 8.00E-14 | 1.42E-04 | 55.81 |
| Apolipoprotein A-1 | rs10504477 | T | C | 0.0121249 | 0.588 | 0.0019941 | 1.20E-09 | 9.40E-05 | 36.97 |
| Apolipoprotein A-1 | rs1055582 | C | T | -0.0154913 | 0.495 | 0.0019665 | 3.30E-15 | 1.58E-04 | 62.05 |
| Apolipoprotein A-1 | rs10745954 | A | G | 0.0143879 | 0.52 | 0.0019642 | 2.40E-13 | 1.36E-04 | 53.66 |
| Apolipoprotein A-1 | rs10748165 | C | T | -0.0110222 | 0.496 | 0.001965 | 2.00E-08 | 8.00E-05 | 31.46 |
| Apolipoprotein A-1 | rs10752898 | T | C | -0.0125655 | 0.442 | 0.0019687 | 1.70E-10 | 1.04E-04 | 40.74 |
| Apolipoprotein A-1 | rs10798615 | T | G | 0.0153327 | 0.467 | 0.0019649 | 6.00E-15 | 1.55E-04 | 60.89 |
| Apolipoprotein A-1 | rs10846497 | A | G | -0.0498539 | 0.907 | 0.0033887 | 5.40E-49 | 5.50E-04 | 216.43 |
| Apolipoprotein A-1 | rs1086056 | T | G | 0.0188421 | 0.155 | 0.0027137 | 3.80E-12 | 1.23E-04 | 48.21 |
| Apolipoprotein A-1 | rs10883451 | T | C | -0.0220804 | 0.504 | 0.0019615 | 2.10E-29 | 3.22E-04 | 126.72 |
| Apolipoprotein A-1 | rs10951983 | A | G | -0.0286839 | 0.767 | 0.002322 | 4.70E-35 | 3.88E-04 | 152.60 |
| Apolipoprotein A-1 | rs11021232 | T | C | 0.0164804 | 0.819 | 0.0025615 | 1.20E-10 | 1.05E-04 | 41.40 |
| Apolipoprotein A-1 | rs11045172 | A | C | -0.0222132 | 0.802 | 0.0024771 | 3.00E-19 | 2.04E-04 | 80.41 |
| Apolipoprotein A-1 | rs11057390 | T | G | -0.0190602 | 0.705 | 0.0021576 | 1.00E-18 | 1.98E-04 | 78.04 |
| Apolipoprotein A-1 | rs11066320 | A | G | -0.012641 | 0.425 | 0.0019883 | 2.00E-10 | 1.03E-04 | 40.42 |
| Apolipoprotein A-1 | rs11067231 | C | A | -0.0220437 | 0.476 | 0.0019627 | 2.90E-29 | 3.21E-04 | 126.14 |
| Apolipoprotein A-1 | rs11089620 | C | G | 0.0321551 | 0.811 | 0.0025143 | 1.90E-37 | 4.16E-04 | 163.56 |
| Apolipoprotein A-1 | rs11159261 | T | C | -0.0117711 | 0.466 | 0.0019737 | 2.50E-09 | 9.05E-05 | 35.57 |
| Apolipoprotein A-1 | rs111849006 | G | A | -0.0146667 | 0.819 | 0.0026034 | 1.80E-08 | 8.07E-05 | 31.74 |
| Apolipoprotein A-1 | rs11245482 | T | C | 0.0112421 | 0.615 | 0.0020207 | 2.60E-08 | 7.87E-05 | 30.95 |
| Apolipoprotein A-1 | rs1132274 | C | A | 0.0288548 | 0.846 | 0.0027173 | 2.40E-26 | 2.87E-04 | 112.76 |
| Apolipoprotein A-1 | rs113710278 | C | T | -0.0462237 | 0.985 | 0.0081768 | 1.60E-08 | 8.13E-05 | 31.96 |
| Apolipoprotein A-1 | rs116006942 | G | A | 0.0284678 | 0.939 | 0.0041556 | 7.40E-12 | 1.19E-04 | 46.93 |
| Apolipoprotein A-1 | rs11632618 | G | A | -0.10804 | 0.93 | 0.0038476 | 1.70E-173 | 2.00E-03 | 788.47 |
| Apolipoprotein A-1 | rs11641548 | A | C | 0.0131611 | 0.56 | 0.0019759 | 2.70E-11 | 1.13E-04 | 44.37 |
| Apolipoprotein A-1 | rs116857878 | C | T | -0.0412261 | 0.98 | 0.0072293 | 1.20E-08 | 8.27E-05 | 32.52 |
| Apolipoprotein A-1 | rs11691486 | T | C | -0.0140998 | 0.76 | 0.002303 | 9.20E-10 | 9.53E-05 | 37.48 |
| Apolipoprotein A-1 | rs117399007 | C | T | 0.0286477 | 0.957 | 0.0048501 | 3.50E-09 | 8.87E-05 | 34.89 |
| Apolipoprotein A-1 | rs117687565 | C | T | -0.0967707 | 0.988 | 0.0093936 | 6.90E-25 | 2.70E-04 | 106.13 |
| Apolipoprotein A-1 | rs117739035 | G | T | 0.0306976 | 0.964 | 0.0052734 | 5.80E-09 | 8.62E-05 | 33.89 |
| Apolipoprotein A-1 | rs11789603 | C | T | -0.0737059 | 0.892 | 0.0031698 | 1.30E-119 | 1.37E-03 | 540.67 |
| Apolipoprotein A-1 | rs11973318 | T | C | 0.0161304 | 0.856 | 0.0028097 | 9.40E-09 | 8.38E-05 | 32.96 |
| Apolipoprotein A-1 | rs12044156 | G | C | 0.0118923 | 0.436 | 0.0019733 | 1.70E-09 | 9.24E-05 | 36.32 |
| Apolipoprotein A-1 | rs12074528 | T | C | -0.0425351 | 0.353 | 0.0020491 | 1.00E-95 | 1.09E-03 | 430.89 |
| Apolipoprotein A-1 | rs12204488 | C | T | -0.0135932 | 0.757 | 0.0022971 | 3.30E-09 | 8.90E-05 | 35.02 |
| Apolipoprotein A-1 | rs12229372 | T | C | -0.0298352 | 0.897 | 0.0033035 | 1.70E-19 | 2.07E-04 | 81.57 |
| Apolipoprotein A-1 | rs12273363 | T | C | 0.0197647 | 0.794 | 0.0024261 | 3.70E-16 | 1.69E-04 | 66.37 |
| Apolipoprotein A-1 | rs12357890 | A | G | 0.0115686 | 0.443 | 0.0019886 | 6.00E-09 | 8.61E-05 | 33.84 |
| Apolipoprotein A-1 | rs12411959 | A | T | 0.0147206 | 0.779 | 0.0023742 | 5.60E-10 | 9.78E-05 | 38.44 |
| Apolipoprotein A-1 | rs12436555 | G | A | 0.0147022 | 0.827 | 0.0026045 | 1.70E-08 | 8.10E-05 | 31.86 |
| Apolipoprotein A-1 | rs12449758 | A | G | 0.0127472 | 0.687 | 0.0021285 | 2.10E-09 | 9.12E-05 | 35.87 |
| Apolipoprotein A-1 | rs12546096 | A | G | 0.0150286 | 0.747 | 0.0022885 | 5.10E-11 | 1.10E-04 | 43.13 |
| Apolipoprotein A-1 | rs1260326 | T | C | 0.025843 | 0.395 | 0.0020019 | 4.00E-38 | 4.24E-04 | 166.65 |
| Apolipoprotein A-1 | rs12740374 | G | T | -0.0443235 | 0.779 | 0.0023598 | 1.10E-78 | 8.96E-04 | 352.78 |
| Apolipoprotein A-1 | rs12976395 | G | C | -0.0380868 | 0.496 | 0.0021122 | 1.10E-72 | 8.26E-04 | 325.15 |
| Apolipoprotein A-1 | rs12984021 | A | C | 0.0175912 | 0.895 | 0.0032199 | 4.70E-08 | 7.59E-05 | 29.85 |
| Apolipoprotein A-1 | rs12987470 | A | T | -0.0120515 | 0.705 | 0.0021532 | 2.20E-08 | 7.97E-05 | 31.33 |
| Apolipoprotein A-1 | rs13024140 | G | C | 0.0115609 | 0.585 | 0.001988 | 6.00E-09 | 8.60E-05 | 33.82 |
| Apolipoprotein A-1 | rs13107325 | C | T | 0.0715695 | 0.925 | 0.0037309 | 5.10E-82 | 9.35E-04 | 367.98 |
| Apolipoprotein A-1 | rs13108218 | A | G | 0.0132813 | 0.385 | 0.0020325 | 6.40E-11 | 1.09E-04 | 42.70 |
| Apolipoprotein A-1 | rs1318175 | C | T | 0.0781476 | 0.84 | 0.0026833 | 1.80E-186 | 2.15E-03 | 848.19 |
| Apolipoprotein A-1 | rs13300004 | A | G | 0.0535297 | 0.987 | 0.0088106 | 1.20E-09 | 9.39E-05 | 36.91 |
| Apolipoprotein A-1 | rs133015 | C | G | -0.020012 | 0.56 | 0.0019848 | 6.60E-24 | 2.58E-04 | 101.66 |
| Apolipoprotein A-1 | rs13326165 | A | G | 0.0194797 | 0.205 | 0.002428 | 1.00E-15 | 1.64E-04 | 64.37 |
| Apolipoprotein A-1 | rs13379043 | T | C | -0.0217249 | 0.72 | 0.0022306 | 2.00E-22 | 2.41E-04 | 94.85 |
| Apolipoprotein A-1 | rs1364422 | C | T | 0.0270358 | 0.72 | 0.0021907 | 5.40E-35 | 3.87E-04 | 152.30 |
| Apolipoprotein A-1 | rs1395221 | G | T | 0.0132541 | 0.602 | 0.0020114 | 4.40E-11 | 1.10E-04 | 43.42 |
| Apolipoprotein A-1 | rs139915535 | A | G | 0.143689 | 0.982 | 0.0073634 | 8.40E-85 | 9.68E-04 | 380.79 |
| Apolipoprotein A-1 | rs1400362 | T | C | -0.0125421 | 0.259 | 0.0022682 | 3.20E-08 | 7.78E-05 | 30.58 |
| Apolipoprotein A-1 | rs140164052 | G | A | 0.0354054 | 0.968 | 0.0055996 | 2.60E-10 | 1.02E-04 | 39.98 |
| Apolipoprotein A-1 | rs1406982 | T | A | 0.0142864 | 0.686 | 0.0021125 | 1.40E-11 | 1.16E-04 | 45.74 |
| Apolipoprotein A-1 | rs144033177 | A | C | 0.0496563 | 0.984 | 0.0080677 | 7.50E-10 | 9.63E-05 | 37.88 |
| Apolipoprotein A-1 | rs144311893 | C | T | -0.122774 | 0.978 | 0.0069051 | 1.00E-70 | 8.03E-04 | 316.13 |
| Apolipoprotein A-1 | rs144475339 | G | C | -0.0581392 | 0.989 | 0.0104988 | 3.10E-08 | 7.80E-05 | 30.67 |
| Apolipoprotein A-1 | rs1446585 | A | G | -0.018597 | 0.756 | 0.0022376 | 9.50E-17 | 1.76E-04 | 69.08 |
| Apolipoprotein A-1 | rs150237291 | T | C | -0.04942 | 0.978 | 0.0067509 | 2.50E-13 | 1.36E-04 | 53.59 |
| Apolipoprotein A-1 | rs150483923 | C | A | -0.0197272 | 0.774 | 0.0023566 | 5.70E-17 | 1.78E-04 | 70.07 |
| Apolipoprotein A-1 | rs150844304 | A | C | 0.075233 | 0.974 | 0.0061589 | 2.60E-34 | 3.79E-04 | 149.21 |
| Apolipoprotein A-1 | rs1601933 | C | T | 0.0843028 | 0.533 | 0.0019967 | 1.00E-200 | 4.51E-03 | 1782.61 |
| Apolipoprotein A-1 | rs17008972 | G | A | -0.0193784 | 0.876 | 0.0029814 | 8.00E-11 | 1.07E-04 | 42.25 |
| Apolipoprotein A-1 | rs17039171 | A | G | 0.0329044 | 0.971 | 0.0058754 | 2.10E-08 | 7.98E-05 | 31.36 |
| Apolipoprotein A-1 | rs17138358 | G | C | 0.0260742 | 0.601 | 0.0020064 | 1.30E-38 | 4.29E-04 | 168.88 |
| Apolipoprotein A-1 | rs1716393 | G | T | 0.0240823 | 0.404 | 0.0020002 | 2.20E-33 | 3.69E-04 | 144.96 |
| Apolipoprotein A-1 | rs1718859 | T | C | -0.0116606 | 0.415 | 0.0019979 | 5.30E-09 | 8.66E-05 | 34.06 |
| Apolipoprotein A-1 | rs17326656 | G | T | 0.0183006 | 0.761 | 0.0023067 | 2.10E-15 | 1.60E-04 | 62.94 |
| Apolipoprotein A-1 | rs174566 | A | G | 0.0349304 | 0.65 | 0.0020555 | 9.20E-65 | 7.34E-04 | 288.77 |
| Apolipoprotein A-1 | rs17520254 | C | G | -0.0581661 | 0.94 | 0.0041468 | 1.10E-44 | 5.00E-04 | 196.75 |
| Apolipoprotein A-1 | rs1800961 | C | T | 0.148995 | 0.969 | 0.0056649 | 1.80E-152 | 1.76E-03 | 691.77 |
| Apolipoprotein A-1 | rs183078 | A | G | -0.0143065 | 0.597 | 0.0020043 | 9.50E-13 | 1.30E-04 | 50.95 |
| Apolipoprotein A-1 | rs1852922 | G | A | -0.0123805 | 0.31 | 0.0021293 | 6.10E-09 | 8.60E-05 | 33.81 |
| Apolipoprotein A-1 | rs1862205 | G | A | -0.0110277 | 0.595 | 0.0020019 | 3.60E-08 | 7.72E-05 | 30.35 |
| Apolipoprotein A-1 | rs1919309 | T | C | -0.0117374 | 0.491 | 0.0019684 | 2.50E-09 | 9.04E-05 | 35.56 |
| Apolipoprotein A-1 | rs2066714 | T | C | -0.0542774 | 0.871 | 0.0029285 | 1.10E-76 | 8.73E-04 | 343.51 |
| Apolipoprotein A-1 | rs2071379 | A | G | 0.0214008 | 0.405 | 0.0020002 | 1.00E-26 | 2.91E-04 | 114.48 |
| Apolipoprotein A-1 | rs2111705 | G | A | 0.0148521 | 0.456 | 0.0019726 | 5.10E-14 | 1.44E-04 | 56.69 |
| Apolipoprotein A-1 | rs2159935 | G | A | -0.0114556 | 0.51 | 0.0019633 | 5.40E-09 | 8.66E-05 | 34.05 |
| Apolipoprotein A-1 | rs2247355 | C | T | -0.0176676 | 0.817 | 0.0025396 | 3.50E-12 | 1.23E-04 | 48.40 |
| Apolipoprotein A-1 | rs2269434 | T | C | -0.0345679 | 0.655 | 0.0020586 | 2.80E-63 | 7.17E-04 | 281.97 |
| Apolipoprotein A-1 | rs2281718 | A | T | -0.0534923 | 0.388 | 0.0020124 | 1.10E-155 | 1.79E-03 | 706.56 |
| Apolipoprotein A-1 | rs2297402 | C | T | 0.0700364 | 0.976 | 0.0066952 | 1.30E-25 | 2.78E-04 | 109.43 |
| Apolipoprotein A-1 | rs2298624 | C | T | -0.0319773 | 0.868 | 0.0028909 | 1.90E-28 | 3.11E-04 | 122.36 |
| Apolipoprotein A-1 | rs235314 | C | T | 0.0214868 | 0.468 | 0.0019757 | 1.50E-27 | 3.01E-04 | 118.28 |
| Apolipoprotein A-1 | rs2419605 | A | G | 0.0312617 | 0.852 | 0.0027812 | 2.60E-29 | 3.21E-04 | 126.35 |
| Apolipoprotein A-1 | rs2494748 | C | T | 0.0322415 | 0.384 | 0.0020252 | 4.60E-57 | 6.44E-04 | 253.45 |
| Apolipoprotein A-1 | rs2520096 | A | G | -0.014065 | 0.73 | 0.0022152 | 2.20E-10 | 1.03E-04 | 40.32 |
| Apolipoprotein A-1 | rs2540951 | A | G | -0.012111 | 0.624 | 0.0020204 | 2.00E-09 | 9.14E-05 | 35.93 |
| Apolipoprotein A-1 | rs2544654 | G | T | -0.0127698 | 0.285 | 0.0021795 | 4.70E-09 | 8.73E-05 | 34.33 |
| Apolipoprotein A-1 | rs254559 | C | A | 0.0170733 | 0.595 | 0.0020022 | 1.50E-17 | 1.85E-04 | 72.71 |
| Apolipoprotein A-1 | rs2642438 | A | G | -0.0291299 | 0.297 | 0.0021405 | 3.60E-42 | 4.71E-04 | 185.20 |
| Apolipoprotein A-1 | rs267738 | T | G | -0.0343544 | 0.781 | 0.0023665 | 9.50E-48 | 5.36E-04 | 210.74 |
| Apolipoprotein A-1 | rs2700892 | A | C | 0.0138725 | 0.547 | 0.001973 | 2.00E-12 | 1.26E-04 | 49.44 |
| Apolipoprotein A-1 | rs2740488 | A | C | 0.0805177 | 0.734 | 0.0022251 | 1.00E-200 | 3.32E-03 | 1309.46 |
| Apolipoprotein A-1 | rs2792751 | T | C | 0.0375265 | 0.275 | 0.0021969 | 2.00E-65 | 7.42E-04 | 291.78 |
| Apolipoprotein A-1 | rs2804894 | G | A | -0.0162576 | 0.265 | 0.0022488 | 4.90E-13 | 1.33E-04 | 52.26 |
| Apolipoprotein A-1 | rs28362901 | C | A | 0.0246903 | 0.912 | 0.0034583 | 9.40E-13 | 1.30E-04 | 50.97 |
| Apolipoprotein A-1 | rs28567061 | A | G | 0.0156946 | 0.777 | 0.0024119 | 7.70E-11 | 1.08E-04 | 42.34 |
| Apolipoprotein A-1 | rs286965 | T | C | 0.0159839 | 0.368 | 0.0020348 | 4.00E-15 | 1.57E-04 | 61.71 |
| Apolipoprotein A-1 | rs28824216 | C | T | -0.03756 | 0.978 | 0.0067523 | 2.70E-08 | 7.87E-05 | 30.94 |
| Apolipoprotein A-1 | rs2925979 | T | C | -0.0285198 | 0.3 | 0.0021301 | 7.00E-41 | 4.56E-04 | 179.26 |
| Apolipoprotein A-1 | rs2943645 | C | T | 0.0338545 | 0.353 | 0.0020487 | 2.40E-61 | 6.94E-04 | 273.06 |
| Apolipoprotein A-1 | rs2965169 | A | C | -0.0188854 | 0.611 | 0.0020125 | 6.30E-21 | 2.24E-04 | 88.06 |
| Apolipoprotein A-1 | rs2972166 | G | A | -0.014956 | 0.725 | 0.0022083 | 1.30E-11 | 1.17E-04 | 45.87 |
| Apolipoprotein A-1 | rs2982521 | A | T | -0.0151936 | 0.372 | 0.0020289 | 7.00E-14 | 1.43E-04 | 56.08 |
| Apolipoprotein A-1 | rs3014246 | C | T | -0.0117175 | 0.296 | 0.0021445 | 4.70E-08 | 7.59E-05 | 29.85 |
| Apolipoprotein A-1 | rs33042 | G | A | -0.0166637 | 0.754 | 0.0022779 | 2.60E-13 | 1.36E-04 | 53.51 |
| Apolipoprotein A-1 | rs331 | G | A | -0.0875575 | 0.735 | 0.0022205 | 1.00E-200 | 3.94E-03 | 1554.90 |
| Apolipoprotein A-1 | rs34138141 | G | T | 0.0180979 | 0.719 | 0.0021874 | 1.30E-16 | 1.74E-04 | 68.46 |
| Apolipoprotein A-1 | rs34180494 | A | C | 0.0127805 | 0.726 | 0.0021993 | 6.20E-09 | 8.59E-05 | 33.77 |
| Apolipoprotein A-1 | rs34397747 | T | C | 0.0393519 | 0.916 | 0.0035613 | 2.20E-28 | 3.10E-04 | 122.10 |
| Apolipoprotein A-1 | rs34642857 | T | C | 0.013374 | 0.749 | 0.0022769 | 4.30E-09 | 8.77E-05 | 34.50 |
| Apolipoprotein A-1 | rs34712273 | C | A | 0.0133466 | 0.421 | 0.0019939 | 2.20E-11 | 1.14E-04 | 44.81 |
| Apolipoprotein A-1 | rs34767118 | A | G | -0.0168906 | 0.674 | 0.0021036 | 9.80E-16 | 1.64E-04 | 64.47 |
| Apolipoprotein A-1 | rs34955778 | T | C | 0.0123761 | 0.581 | 0.0019808 | 4.20E-10 | 9.93E-05 | 39.04 |
| Apolipoprotein A-1 | rs351862 | C | T | -0.0184288 | 0.885 | 0.0030954 | 2.60E-09 | 9.01E-05 | 35.45 |
| Apolipoprotein A-1 | rs35650976 | C | T | -0.0150252 | 0.7 | 0.0021524 | 2.90E-12 | 1.24E-04 | 48.73 |
| Apolipoprotein A-1 | rs36096231 | C | T | 0.020767 | 0.928 | 0.0037877 | 4.20E-08 | 7.64E-05 | 30.06 |
| Apolipoprotein A-1 | rs367070 | A | G | -0.0390569 | 0.775 | 0.0023556 | 9.60E-62 | 6.99E-04 | 274.91 |
| Apolipoprotein A-1 | rs367677 | A | G | -0.0174973 | 0.76 | 0.0023134 | 3.90E-14 | 1.45E-04 | 57.21 |
| Apolipoprotein A-1 | rs3732356 | G | T | 0.036854 | 0.066 | 0.0039819 | 2.10E-20 | 2.18E-04 | 85.66 |
| Apolipoprotein A-1 | rs3740688 | G | T | -0.0156318 | 0.455 | 0.0019735 | 2.40E-15 | 1.60E-04 | 62.74 |
| Apolipoprotein A-1 | rs3747973 | A | G | -0.014824 | 0.407 | 0.0019959 | 1.10E-13 | 1.40E-04 | 55.16 |
| Apolipoprotein A-1 | rs3749748 | C | T | -0.0220869 | 0.753 | 0.002284 | 4.00E-22 | 2.38E-04 | 93.51 |
| Apolipoprotein A-1 | rs3768321 | G | T | 0.0423077 | 0.803 | 0.0024662 | 5.70E-66 | 7.48E-04 | 294.30 |
| Apolipoprotein A-1 | rs3798233 | A | C | -0.0180323 | 0.598 | 0.0019999 | 1.90E-19 | 2.07E-04 | 81.30 |
| Apolipoprotein A-1 | rs3802548 | T | A | -0.0340926 | 0.76 | 0.0022983 | 8.90E-50 | 5.59E-04 | 220.03 |
| Apolipoprotein A-1 | rs3859588 | T | A | 0.0146912 | 0.782 | 0.0024023 | 9.60E-10 | 9.51E-05 | 37.40 |
| Apolipoprotein A-1 | rs3915932 | G | C | -0.015788 | 0.585 | 0.0019957 | 2.60E-15 | 1.59E-04 | 62.58 |
| Apolipoprotein A-1 | rs3936511 | A | G | 0.0220026 | 0.808 | 0.0024924 | 1.10E-18 | 1.98E-04 | 77.93 |
| Apolipoprotein A-1 | rs41272086 | G | A | 0.0372996 | 0.894 | 0.0031961 | 1.80E-31 | 3.46E-04 | 136.20 |
| Apolipoprotein A-1 | rs4239651 | T | C | -0.0264197 | 0.206 | 0.0024294 | 1.50E-27 | 3.01E-04 | 118.26 |
| Apolipoprotein A-1 | rs429358 | T | C | 0.102369 | 0.846 | 0.0027163 | 1.00E-200 | 3.60E-03 | 1420.27 |
| Apolipoprotein A-1 | rs4441609 | T | C | -0.0111193 | 0.374 | 0.0020254 | 4.00E-08 | 7.66E-05 | 30.14 |
| Apolipoprotein A-1 | rs4450131 | T | C | -0.01161 | 0.475 | 0.0019736 | 4.00E-09 | 8.80E-05 | 34.61 |
| Apolipoprotein A-1 | rs450244 | T | C | -0.0302382 | 0.089 | 0.0034508 | 1.90E-18 | 1.95E-04 | 76.78 |
| Apolipoprotein A-1 | rs4632228 | G | T | 0.0198726 | 0.791 | 0.0024087 | 1.60E-16 | 1.73E-04 | 68.07 |
| Apolipoprotein A-1 | rs4652192 | C | A | -0.0150196 | 0.809 | 0.0025069 | 2.10E-09 | 9.13E-05 | 35.90 |
| Apolipoprotein A-1 | rs4676609 | C | T | -0.0150331 | 0.804 | 0.0024749 | 1.20E-09 | 9.38E-05 | 36.90 |
| Apolipoprotein A-1 | rs4714554 | A | G | -0.0143264 | 0.389 | 0.0020313 | 1.80E-12 | 1.26E-04 | 49.74 |
| Apolipoprotein A-1 | rs4725969 | C | T | -0.0123429 | 0.66 | 0.0020852 | 3.20E-09 | 8.91E-05 | 35.04 |
| Apolipoprotein A-1 | rs4762756 | T | C | -0.0140001 | 0.264 | 0.0022482 | 4.70E-10 | 9.86E-05 | 38.78 |
| Apolipoprotein A-1 | rs4775033 | A | T | 0.0500198 | 0.88 | 0.0030268 | 2.40E-61 | 6.94E-04 | 273.09 |
| Apolipoprotein A-1 | rs4784709 | T | A | 0.0618129 | 0.041 | 0.0049456 | 7.60E-36 | 3.97E-04 | 156.21 |
| Apolipoprotein A-1 | rs4820346 | C | G | -0.0129181 | 0.307 | 0.0021389 | 1.50E-09 | 9.28E-05 | 36.48 |
| Apolipoprotein A-1 | rs4871603 | C | T | -0.0161806 | 0.347 | 0.0020576 | 3.70E-15 | 1.57E-04 | 61.84 |
| Apolipoprotein A-1 | rs4875043 | A | C | 0.0151542 | 0.783 | 0.0024048 | 2.90E-10 | 1.01E-04 | 39.71 |
| Apolipoprotein A-1 | rs4930352 | G | T | -0.0167052 | 0.506 | 0.0019971 | 6.00E-17 | 1.78E-04 | 69.97 |
| Apolipoprotein A-1 | rs49675 | G | A | -0.0211674 | 0.902 | 0.0033092 | 1.60E-10 | 1.04E-04 | 40.92 |
| Apolipoprotein A-1 | rs55710224 | G | A | -0.01218 | 0.501 | 0.0019653 | 5.70E-10 | 9.77E-05 | 38.41 |
| Apolipoprotein A-1 | rs55781197 | A | G | -0.056117 | 0.885 | 0.0030579 | 3.20E-75 | 8.56E-04 | 336.78 |
| Apolipoprotein A-1 | rs557933 | A | C | -0.0190966 | 0.48 | 0.0019651 | 2.50E-22 | 2.40E-04 | 94.44 |
| Apolipoprotein A-1 | rs55801554 | C | A | -0.0137178 | 0.753 | 0.0022792 | 1.80E-09 | 9.21E-05 | 36.22 |
| Apolipoprotein A-1 | rs559355 | A | T | 0.0446533 | 0.843 | 0.0026913 | 8.00E-62 | 7.00E-04 | 275.28 |
| Apolipoprotein A-1 | rs56090699 | T | C | -0.0153025 | 0.699 | 0.002151 | 1.10E-12 | 1.29E-04 | 50.61 |
| Apolipoprotein A-1 | rs58473820 | C | T | -0.0207422 | 0.622 | 0.0020265 | 1.40E-24 | 2.66E-04 | 104.77 |
| Apolipoprotein A-1 | rs589942 | C | G | 0.018547 | 0.662 | 0.0020714 | 3.40E-19 | 2.04E-04 | 80.17 |
| Apolipoprotein A-1 | rs59104589 | C | T | -0.0170267 | 0.642 | 0.0020421 | 7.60E-17 | 1.77E-04 | 69.52 |
| Apolipoprotein A-1 | rs59347135 | C | G | 0.0806336 | 0.954 | 0.0047902 | 1.40E-63 | 7.20E-04 | 283.35 |
| Apolipoprotein A-1 | rs6059958 | C | T | -0.0172529 | 0.829 | 0.0026308 | 5.40E-11 | 1.09E-04 | 43.01 |
| Apolipoprotein A-1 | rs6062510 | G | C | 0.015191 | 0.326 | 0.0020965 | 4.30E-13 | 1.34E-04 | 52.50 |
| Apolipoprotein A-1 | rs60667919 | C | A | -0.0135147 | 0.321 | 0.0021261 | 2.10E-10 | 1.03E-04 | 40.41 |
| Apolipoprotein A-1 | rs613808 | A | G | 0.0849428 | 0.285 | 0.0021841 | 1.00E-200 | 3.83E-03 | 1512.56 |
| Apolipoprotein A-1 | rs61596977 | C | T | 0.0154721 | 0.86 | 0.0028267 | 4.40E-08 | 7.62E-05 | 29.96 |
| Apolipoprotein A-1 | rs61789561 | G | A | -0.0313064 | 0.804 | 0.0024721 | 9.40E-37 | 4.08E-04 | 160.37 |
| Apolipoprotein A-1 | rs61805076 | T | C | 0.0153261 | 0.667 | 0.002078 | 1.60E-13 | 1.38E-04 | 54.40 |
| Apolipoprotein A-1 | rs62114506 | G | C | -0.0140515 | 0.738 | 0.0022263 | 2.80E-10 | 1.01E-04 | 39.84 |
| Apolipoprotein A-1 | rs62135193 | C | T | 0.0108683 | 0.459 | 0.0019762 | 3.80E-08 | 7.69E-05 | 30.24 |
| Apolipoprotein A-1 | rs62405458 | C | T | 0.0147331 | 0.817 | 0.0025502 | 7.60E-09 | 8.49E-05 | 33.38 |
| Apolipoprotein A-1 | rs635634 | C | T | -0.030651 | 0.816 | 0.0025369 | 1.30E-33 | 3.71E-04 | 145.97 |
| Apolipoprotein A-1 | rs6448429 | C | T | 0.02225 | 0.834 | 0.0026575 | 5.60E-17 | 1.78E-04 | 70.10 |
| Apolipoprotein A-1 | rs6458867 | A | G | -0.0113728 | 0.356 | 0.002052 | 3.00E-08 | 7.81E-05 | 30.72 |
| Apolipoprotein A-1 | rs6467595 | C | T | -0.0145539 | 0.228 | 0.0023488 | 5.80E-10 | 9.76E-05 | 38.39 |
| Apolipoprotein A-1 | rs6469605 | C | T | -0.0350594 | 0.432 | 0.0019825 | 5.50E-70 | 7.95E-04 | 312.73 |
| Apolipoprotein A-1 | rs676210 | G | A | -0.0601182 | 0.795 | 0.0024242 | 9.20E-136 | 1.56E-03 | 614.99 |
| Apolipoprotein A-1 | rs6765484 | C | T | -0.0203492 | 0.527 | 0.0019659 | 4.10E-25 | 2.72E-04 | 107.14 |
| Apolipoprotein A-1 | rs6807935 | A | G | 0.0126318 | 0.657 | 0.0020673 | 9.90E-10 | 9.49E-05 | 37.34 |
| Apolipoprotein A-1 | rs681869 | C | T | 0.0164637 | 0.296 | 0.0021513 | 2.00E-14 | 1.49E-04 | 58.57 |
| Apolipoprotein A-1 | rs686030 | C | A | -0.0563409 | 0.141 | 0.0028195 | 7.80E-89 | 1.01E-03 | 399.30 |
| Apolipoprotein A-1 | rs6977416 | G | A | 0.0157237 | 0.666 | 0.0020985 | 6.70E-14 | 1.43E-04 | 56.14 |
| Apolipoprotein A-1 | rs698927 | A | C | -0.0245571 | 0.816 | 0.0025304 | 2.90E-22 | 2.39E-04 | 94.18 |
| Apolipoprotein A-1 | rs7119167 | T | C | -0.0136483 | 0.789 | 0.0024035 | 1.40E-08 | 8.20E-05 | 32.25 |
| Apolipoprotein A-1 | rs7134035 | C | T | -0.0165743 | 0.184 | 0.0025628 | 1.00E-10 | 1.06E-04 | 41.83 |
| Apolipoprotein A-1 | rs71355297 | C | T | 0.0141052 | 0.77 | 0.002336 | 1.60E-09 | 9.27E-05 | 36.46 |
| Apolipoprotein A-1 | rs7136506 | T | C | 0.031893 | 0.784 | 0.0024231 | 1.40E-39 | 4.40E-04 | 173.24 |
| Apolipoprotein A-1 | rs7170463 | A | G | -0.0161348 | 0.689 | 0.0021184 | 2.60E-14 | 1.48E-04 | 58.01 |
| Apolipoprotein A-1 | rs7186799 | A | C | 0.0185871 | 0.562 | 0.00197 | 3.90E-21 | 2.26E-04 | 89.02 |
| Apolipoprotein A-1 | rs7238484 | G | T | 0.0214317 | 0.732 | 0.0022171 | 4.20E-22 | 2.38E-04 | 93.45 |
| Apolipoprotein A-1 | rs7251640 | T | C | -0.0152317 | 0.806 | 0.0025004 | 1.10E-09 | 9.44E-05 | 37.11 |
| Apolipoprotein A-1 | rs72729623 | C | T | 0.0153385 | 0.857 | 0.0028026 | 4.40E-08 | 7.62E-05 | 29.95 |
| Apolipoprotein A-1 | rs72836561 | C | T | 0.14208 | 0.969 | 0.0056404 | 5.20E-140 | 1.61E-03 | 634.52 |
| Apolipoprotein A-1 | rs72926946 | C | A | 0.015461 | 0.704 | 0.0021432 | 5.40E-13 | 1.32E-04 | 52.04 |
| Apolipoprotein A-1 | rs72959041 | G | A | 0.0359893 | 0.951 | 0.0046006 | 5.20E-15 | 1.56E-04 | 61.19 |
| Apolipoprotein A-1 | rs7304603 | T | C | 0.0126461 | 0.462 | 0.0019742 | 1.50E-10 | 1.04E-04 | 41.03 |
| Apolipoprotein A-1 | rs73052033 | T | C | 0.0183914 | 0.815 | 0.0025319 | 3.80E-13 | 1.34E-04 | 52.77 |
| Apolipoprotein A-1 | rs73119306 | A | G | -0.0276532 | 0.759 | 0.0022913 | 1.50E-33 | 3.70E-04 | 145.66 |
| Apolipoprotein A-1 | rs73216700 | G | A | -0.0164155 | 0.57 | 0.0019827 | 1.20E-16 | 1.74E-04 | 68.55 |
| Apolipoprotein A-1 | rs73375614 | T | C | -0.0242534 | 0.944 | 0.0043094 | 1.80E-08 | 8.06E-05 | 31.68 |
| Apolipoprotein A-1 | rs737338 | C | T | 0.108223 | 0.965 | 0.0053341 | 1.60E-91 | 1.05E-03 | 411.64 |
| Apolipoprotein A-1 | rs74444445 | T | C | 0.0591225 | 0.976 | 0.0067 | 1.10E-18 | 1.98E-04 | 77.87 |
| Apolipoprotein A-1 | rs74456742 | G | A | -0.030615 | 0.962 | 0.0053001 | 7.60E-09 | 8.48E-05 | 33.37 |
| Apolipoprotein A-1 | rs74500135 | T | C | -0.0736017 | 0.99 | 0.0103377 | 1.10E-12 | 1.29E-04 | 50.69 |
| Apolipoprotein A-1 | rs74963256 | T | C | 0.0191768 | 0.907 | 0.0034083 | 1.80E-08 | 8.05E-05 | 31.66 |
| Apolipoprotein A-1 | rs7503353 | G | T | 0.0135036 | 0.468 | 0.0019733 | 7.70E-12 | 1.19E-04 | 46.83 |
| Apolipoprotein A-1 | rs7514202 | G | A | -0.0167289 | 0.134 | 0.0028918 | 7.30E-09 | 8.51E-05 | 33.47 |
| Apolipoprotein A-1 | rs75152587 | G | T | 0.0797509 | 0.987 | 0.0087961 | 1.20E-19 | 2.09E-04 | 82.20 |
| Apolipoprotein A-1 | rs75246752 | G | C | -0.0496485 | 0.987 | 0.0086752 | 1.00E-08 | 8.33E-05 | 32.75 |
| Apolipoprotein A-1 | rs75265117 | C | G | -0.0314966 | 0.88 | 0.0030206 | 1.90E-25 | 2.76E-04 | 108.73 |
| Apolipoprotein A-1 | rs75406471 | G | A | 0.0246893 | 0.846 | 0.0027218 | 1.20E-19 | 2.09E-04 | 82.28 |
| Apolipoprotein A-1 | rs76213248 | C | T | -0.0213327 | 0.59 | 0.002002 | 1.60E-26 | 2.89E-04 | 113.55 |
| Apolipoprotein A-1 | rs76428106 | T | C | 0.0607578 | 0.987 | 0.0090464 | 1.90E-11 | 1.15E-04 | 45.11 |
| Apolipoprotein A-1 | rs76456334 | T | C | 0.0369205 | 0.96 | 0.0051203 | 5.60E-13 | 1.32E-04 | 51.99 |
| Apolipoprotein A-1 | rs7700617 | C | A | 0.0126975 | 0.503 | 0.0019699 | 1.20E-10 | 1.06E-04 | 41.55 |
| Apolipoprotein A-1 | rs7784748 | T | C | -0.0111733 | 0.428 | 0.0019826 | 1.70E-08 | 8.08E-05 | 31.76 |
| Apolipoprotein A-1 | rs77960347 | A | G | -0.336049 | 0.987 | 0.0085597 | 1.00E-200 | 3.90E-03 | 1541.30 |
| Apolipoprotein A-1 | rs78058190 | G | A | 0.0662511 | 0.95 | 0.0050516 | 2.70E-39 | 4.37E-04 | 172.00 |
| Apolipoprotein A-1 | rs7817574 | T | C | -0.0361808 | 0.815 | 0.0025202 | 9.70E-47 | 5.24E-04 | 206.11 |
| Apolipoprotein A-1 | rs78359342 | G | T | 0.0131701 | 0.733 | 0.0022296 | 3.50E-09 | 8.87E-05 | 34.89 |
| Apolipoprotein A-1 | rs7896518 | A | G | -0.0153161 | 0.572 | 0.0020057 | 2.20E-14 | 1.48E-04 | 58.31 |
| Apolipoprotein A-1 | rs79506257 | G | A | -0.0282748 | 0.952 | 0.0046512 | 1.20E-09 | 9.40E-05 | 36.95 |
| Apolipoprotein A-1 | rs79940707 | C | T | -0.0190564 | 0.843 | 0.0026859 | 1.30E-12 | 1.28E-04 | 50.34 |
| Apolipoprotein A-1 | rs79984435 | G | A | 0.0856304 | 0.908 | 0.0033738 | 4.10E-142 | 1.64E-03 | 644.18 |
| Apolipoprotein A-1 | rs80236739 | A | G | 0.0320701 | 0.95 | 0.0045066 | 1.10E-12 | 1.29E-04 | 50.64 |
| Apolipoprotein A-1 | rs8039305 | T | C | -0.0127294 | 0.524 | 0.0019694 | 1.00E-10 | 1.06E-04 | 41.78 |
| Apolipoprotein A-1 | rs8086351 | C | G | -0.0961121 | 0.176 | 0.0025786 | 1.00E-200 | 3.52E-03 | 1389.25 |
| Apolipoprotein A-1 | rs8103728 | C | G | -0.0228755 | 0.33 | 0.0020892 | 6.70E-28 | 3.05E-04 | 119.89 |
| Apolipoprotein A-1 | rs848638 | C | T | 0.0112 | 0.64 | 0.0020414 | 4.10E-08 | 7.65E-05 | 30.10 |
| Apolipoprotein A-1 | rs921919 | G | A | 0.0370772 | 0.331 | 0.0021276 | 5.10E-68 | 7.72E-04 | 303.71 |
| Apolipoprotein A-1 | rs9347737 | A | G | 0.0111895 | 0.572 | 0.0020062 | 2.40E-08 | 7.91E-05 | 31.11 |
| Apolipoprotein A-1 | rs9426827 | T | C | -0.0170499 | 0.522 | 0.0019592 | 3.30E-18 | 1.93E-04 | 75.73 |
| Apolipoprotein A-1 | rs9471972 | G | A | -0.0269155 | 0.465 | 0.0019688 | 1.50E-42 | 4.75E-04 | 186.89 |
| Apolipoprotein A-1 | rs9559901 | A | C | -0.0116984 | 0.661 | 0.0020837 | 2.00E-08 | 8.02E-05 | 31.52 |
| Apolipoprotein A-1 | rs9604045 | G | T | -0.0154374 | 0.746 | 0.0023562 | 5.70E-11 | 1.09E-04 | 42.93 |
| Apolipoprotein A-1 | rs9608972 | T | C | 0.017449 | 0.759 | 0.0023011 | 3.40E-14 | 1.46E-04 | 57.50 |
| Apolipoprotein A-1 | rs976002 | A | G | -0.0230775 | 0.755 | 0.0022822 | 4.90E-24 | 2.60E-04 | 102.25 |
| Apolipoprotein A-1 | rs9817452 | G | T | -0.0174723 | 0.612 | 0.0020228 | 5.70E-18 | 1.90E-04 | 74.61 |
| Apolipoprotein A-1 | rs9866679 | G | T | -0.0127699 | 0.236 | 0.0023124 | 3.30E-08 | 7.76E-05 | 30.50 |
| Apolipoprotein A-1 | rs989075 | A | T | -0.0188142 | 0.525 | 0.0019828 | 2.30E-21 | 2.29E-04 | 90.03 |
| Apolipoprotein A-1 | rs9987289 | A | G | -0.0853006 | 0.091 | 0.0034015 | 8.70E-139 | 1.60E-03 | 628.88 |
| Apolipoprotein A-1 | rs9989419 | A | G | -0.11499 | 0.394 | 0.001995 | 1.00E-200 | 8.38E-03 | 3322.15 |
| Apolipoprotein B | rs1003533 | C | T | 0.0199788 | 0.812 | 0.0026634 | 6.30E-14 | 1.28E-04 | 56.27 |
| Apolipoprotein B | rs10151436 | A | T | 0.0188241 | 0.888 | 0.003305 | 1.20E-08 | 7.39E-05 | 32.44 |
| Apolipoprotein B | rs10448340 | T | G | 0.0171608 | 0.68 | 0.0022317 | 1.50E-14 | 1.35E-04 | 59.13 |
| Apolipoprotein B | rs10832963 | T | G | -0.0220635 | 0.256 | 0.0023894 | 2.60E-20 | 1.94E-04 | 85.27 |
| Apolipoprotein B | rs10858093 | C | T | -0.0378458 | 0.967 | 0.0059224 | 1.70E-10 | 9.30E-05 | 40.84 |
| Apolipoprotein B | rs10876450 | T | C | -0.0157064 | 0.826 | 0.0027436 | 1.00E-08 | 7.46E-05 | 32.77 |
| Apolipoprotein B | rs10953298 | C | T | 0.0171918 | 0.764 | 0.0024599 | 2.80E-12 | 1.11E-04 | 48.84 |
| Apolipoprotein B | rs11057397 | C | T | 0.0182734 | 0.663 | 0.0022023 | 1.10E-16 | 1.57E-04 | 68.84 |
| Apolipoprotein B | rs11206517 | T | G | -0.0685214 | 0.967 | 0.0057832 | 2.20E-32 | 3.20E-04 | 140.38 |
| Apolipoprotein B | rs112220485 | T | C | -0.020503 | 0.914 | 0.0037369 | 4.10E-08 | 6.85E-05 | 30.10 |
| Apolipoprotein B | rs112426331 | G | A | 0.0344284 | 0.957 | 0.0051951 | 3.40E-11 | 1.00E-04 | 43.92 |
| Apolipoprotein B | rs112758337 | G | A | 0.0211918 | 0.814 | 0.0026803 | 2.60E-15 | 1.42E-04 | 62.51 |
| Apolipoprotein B | rs113177823 | G | A | 0.0411203 | 0.946 | 0.0046496 | 9.20E-19 | 1.78E-04 | 78.21 |
| Apolipoprotein B | rs113330691 | G | A | 0.282453 | 0.965 | 0.0056077 | 1.00E-200 | 5.74E-03 | 2536.97 |
| Apolipoprotein B | rs113743631 | G | A | -0.0637083 | 0.979 | 0.007548 | 3.20E-17 | 1.62E-04 | 71.24 |
| Apolipoprotein B | rs114165349 | G | C | -0.0900792 | 0.977 | 0.006934 | 1.40E-38 | 3.84E-04 | 168.76 |
| Apolipoprotein B | rs115478735 | A | T | -0.0412279 | 0.816 | 0.0026828 | 2.70E-53 | 5.37E-04 | 236.16 |
| Apolipoprotein B | rs11568318 | C | A | -0.0288504 | 0.934 | 0.0041601 | 4.10E-12 | 1.09E-04 | 48.10 |
| Apolipoprotein B | rs115692156 | A | G | 0.111354 | 0.991 | 0.011863 | 6.20E-21 | 2.01E-04 | 88.11 |
| Apolipoprotein B | rs115704890 | T | A | -0.0323101 | 0.918 | 0.0037771 | 1.20E-17 | 1.67E-04 | 73.17 |
| Apolipoprotein B | rs11591147 | G | T | 0.345975 | 0.983 | 0.0079061 | 1.00E-200 | 4.34E-03 | 1914.97 |
| Apolipoprotein B | rs11601507 | C | A | -0.0415074 | 0.931 | 0.0040387 | 8.90E-25 | 2.40E-04 | 105.62 |
| Apolipoprotein B | rs11621792 | C | T | -0.0210426 | 0.547 | 0.0021049 | 1.60E-23 | 2.27E-04 | 99.94 |
| Apolipoprotein B | rs116734477 | C | T | 0.0634849 | 0.959 | 0.00527 | 2.00E-33 | 3.30E-04 | 145.12 |
| Apolipoprotein B | rs1169292 | C | T | -0.0217273 | 0.689 | 0.0022523 | 5.10E-22 | 2.12E-04 | 93.06 |
| Apolipoprotein B | rs117733303 | A | G | -0.0864237 | 0.982 | 0.0077159 | 4.00E-29 | 2.86E-04 | 125.45 |
| Apolipoprotein B | rs118039278 | G | A | -0.086807 | 0.921 | 0.0038731 | 3.00E-111 | 1.14E-03 | 502.32 |
| Apolipoprotein B | rs12046278 | T | C | 0.0155363 | 0.655 | 0.0021741 | 8.90E-13 | 1.16E-04 | 51.07 |
| Apolipoprotein B | rs12054451 | T | G | -0.015958 | 0.741 | 0.0023832 | 2.10E-11 | 1.02E-04 | 44.84 |
| Apolipoprotein B | rs12208357 | C | T | -0.0632075 | 0.93 | 0.0040845 | 5.10E-54 | 5.45E-04 | 239.47 |
| Apolipoprotein B | rs1229984 | T | C | -0.0388703 | 0.027 | 0.0063169 | 7.60E-10 | 8.62E-05 | 37.86 |
| Apolipoprotein B | rs12471768 | T | C | -0.0139182 | 0.296 | 0.0022709 | 8.80E-10 | 8.55E-05 | 37.56 |
| Apolipoprotein B | rs1250258 | C | T | -0.0140874 | 0.263 | 0.0023573 | 2.30E-09 | 8.13E-05 | 35.71 |
| Apolipoprotein B | rs1260326 | T | C | 0.0495629 | 0.395 | 0.0021161 | 2.50E-121 | 1.25E-03 | 548.59 |
| Apolipoprotein B | rs12691088 | G | A | -0.246284 | 0.979 | 0.0077007 | 1.00E-200 | 2.32E-03 | 1022.84 |
| Apolipoprotein B | rs12916 | T | C | -0.0549232 | 0.6 | 0.0021215 | 8.80E-148 | 1.52E-03 | 670.25 |
| Apolipoprotein B | rs12970 | G | A | 0.0251616 | 0.939 | 0.0043558 | 7.60E-09 | 7.60E-05 | 33.37 |
| Apolipoprotein B | rs13076933 | T | G | 0.018049 | 0.741 | 0.0023841 | 3.70E-14 | 1.30E-04 | 57.31 |
| Apolipoprotein B | rs13108218 | A | G | 0.0228809 | 0.385 | 0.0021541 | 2.30E-26 | 2.57E-04 | 112.83 |
| Apolipoprotein B | rs13230111 | A | G | 0.0136169 | 0.508 | 0.0020795 | 5.80E-11 | 9.76E-05 | 42.88 |
| Apolipoprotein B | rs13247874 | C | T | 0.0215194 | 0.803 | 0.0026151 | 1.90E-16 | 1.54E-04 | 67.71 |
| Apolipoprotein B | rs13379043 | T | C | 0.0145274 | 0.72 | 0.0023603 | 7.50E-10 | 8.62E-05 | 37.88 |
| Apolipoprotein B | rs13389219 | C | T | 0.0162802 | 0.607 | 0.0021194 | 1.60E-14 | 1.34E-04 | 59.00 |
| Apolipoprotein B | rs1358980 | C | T | -0.0150327 | 0.517 | 0.0020959 | 7.40E-13 | 1.17E-04 | 51.45 |
| Apolipoprotein B | rs13702 | T | C | 0.0298366 | 0.712 | 0.002296 | 1.30E-38 | 3.84E-04 | 168.86 |
| Apolipoprotein B | rs138354 | T | C | 0.0120165 | 0.465 | 0.0020853 | 8.30E-09 | 7.56E-05 | 33.20 |
| Apolipoprotein B | rs138692741 | C | T | -0.0715279 | 0.964 | 0.0056903 | 3.10E-36 | 3.60E-04 | 158.01 |
| Apolipoprotein B | rs141469619 | A | G | -0.0778728 | 0.99 | 0.0111246 | 2.60E-12 | 1.12E-04 | 49.00 |
| Apolipoprotein B | rs143020224 | C | G | 0.16537 | 0.881 | 0.0031807 | 1.00E-200 | 6.12E-03 | 2703.14 |
| Apolipoprotein B | rs145730801 | T | C | -0.0374237 | 0.956 | 0.0051558 | 3.90E-13 | 1.20E-04 | 52.69 |
| Apolipoprotein B | rs1458038 | C | T | 0.0165547 | 0.709 | 0.002296 | 5.60E-13 | 1.18E-04 | 51.99 |
| Apolipoprotein B | rs146534110 | G | T | -0.0666046 | 0.987 | 0.0090698 | 2.10E-13 | 1.23E-04 | 53.93 |
| Apolipoprotein B | rs147539187 | C | G | 0.0227989 | 0.926 | 0.0039828 | 1.00E-08 | 7.46E-05 | 32.77 |
| Apolipoprotein B | rs148601586 | C | G | -0.183606 | 0.987 | 0.0091828 | 6.10E-89 | 9.09E-04 | 399.78 |
| Apolipoprotein B | rs150474434 | G | A | 0.0342764 | 0.899 | 0.0034542 | 3.30E-23 | 2.24E-04 | 98.47 |
| Apolipoprotein B | rs150820726 | A | T | -0.0714924 | 0.99 | 0.01057 | 1.30E-11 | 1.04E-04 | 45.75 |
| Apolipoprotein B | rs1556562 | G | T | -0.0160289 | 0.21 | 0.0024797 | 1.00E-10 | 9.51E-05 | 41.79 |
| Apolipoprotein B | rs1561139 | G | T | 0.0130731 | 0.576 | 0.0021056 | 5.30E-10 | 8.78E-05 | 38.55 |
| Apolipoprotein B | rs17036085 | A | G | 0.0685676 | 0.988 | 0.009445 | 3.90E-13 | 1.20E-04 | 52.70 |
| Apolipoprotein B | rs17050272 | G | A | 0.0243835 | 0.591 | 0.002106 | 5.30E-31 | 3.05E-04 | 134.05 |
| Apolipoprotein B | rs17447211 | C | G | -0.0143792 | 0.671 | 0.0022058 | 7.10E-11 | 9.67E-05 | 42.49 |
| Apolipoprotein B | rs174564 | A | G | 0.0451401 | 0.651 | 0.0021836 | 6.10E-95 | 9.72E-04 | 427.36 |
| Apolipoprotein B | rs17476364 | T | C | 0.0211293 | 0.892 | 0.0033495 | 2.80E-10 | 9.06E-05 | 39.79 |
| Apolipoprotein B | rs1801689 | A | C | -0.0647127 | 0.969 | 0.0060438 | 9.40E-27 | 2.61E-04 | 114.64 |
| Apolipoprotein B | rs188608977 | G | T | -0.0619017 | 0.989 | 0.0103757 | 2.40E-09 | 8.10E-05 | 35.59 |
| Apolipoprotein B | rs1888488 | C | T | -0.0173094 | 0.435 | 0.002104 | 1.90E-16 | 1.54E-04 | 67.68 |
| Apolipoprotein B | rs2058122 | T | C | -0.0186363 | 0.142 | 0.0030039 | 5.50E-10 | 8.76E-05 | 38.49 |
| Apolipoprotein B | rs2068888 | G | A | 0.0241457 | 0.549 | 0.0020888 | 6.60E-31 | 3.04E-04 | 133.62 |
| Apolipoprotein B | rs2073547 | A | G | -0.0312222 | 0.816 | 0.002666 | 1.10E-31 | 3.12E-04 | 137.16 |
| Apolipoprotein B | rs2137234 | T | C | -0.0151513 | 0.805 | 0.002625 | 7.80E-09 | 7.58E-05 | 33.31 |
| Apolipoprotein B | rs2160994 | T | C | -0.0174474 | 0.353 | 0.0021783 | 1.20E-15 | 1.46E-04 | 64.15 |
| Apolipoprotein B | rs2238162 | C | T | 0.0225635 | 0.477 | 0.0020833 | 2.50E-27 | 2.67E-04 | 117.31 |
| Apolipoprotein B | rs2256814 | G | A | -0.0144642 | 0.802 | 0.002618 | 3.30E-08 | 6.95E-05 | 30.52 |
| Apolipoprotein B | rs2335708 | A | G | -0.0197829 | 0.682 | 0.0023102 | 1.10E-17 | 1.67E-04 | 73.33 |
| Apolipoprotein B | rs261290 | T | C | 0.0209689 | 0.346 | 0.0021927 | 1.10E-21 | 2.08E-04 | 91.46 |
| Apolipoprotein B | rs2618566 | G | T | 0.0302601 | 0.34 | 0.0021945 | 3.00E-43 | 4.33E-04 | 190.13 |
| Apolipoprotein B | rs2705619 | G | A | -0.0143883 | 0.294 | 0.0022852 | 3.10E-10 | 9.02E-05 | 39.64 |
| Apolipoprotein B | rs2737263 | G | T | 0.0186563 | 0.72 | 0.002315 | 7.70E-16 | 1.48E-04 | 64.94 |
| Apolipoprotein B | rs2738447 | A | C | -0.0436053 | 0.407 | 0.0020917 | 1.60E-96 | 9.88E-04 | 434.58 |
| Apolipoprotein B | rs2761311 | C | T | -0.0121175 | 0.428 | 0.0021115 | 9.50E-09 | 7.50E-05 | 32.93 |
| Apolipoprotein B | rs278981 | T | C | -0.0149629 | 0.242 | 0.0023972 | 4.30E-10 | 8.87E-05 | 38.96 |
| Apolipoprotein B | rs2807854 | T | C | -0.0163014 | 0.329 | 0.0022058 | 1.50E-13 | 1.24E-04 | 54.62 |
| Apolipoprotein B | rs28406917 | C | T | -0.0134105 | 0.572 | 0.0021088 | 2.00E-10 | 9.21E-05 | 40.44 |
| Apolipoprotein B | rs28601761 | C | G | 0.0724469 | 0.581 | 0.00213 | 1.00E-200 | 2.63E-03 | 1156.89 |
| Apolipoprotein B | rs28768427 | G | A | -0.0118358 | 0.48 | 0.0021443 | 3.40E-08 | 6.94E-05 | 30.47 |
| Apolipoprotein B | rs34042070 | C | G | -0.0495027 | 0.812 | 0.0026727 | 1.40E-76 | 7.80E-04 | 343.06 |
| Apolipoprotein B | rs34767236 | G | A | -0.0198565 | 0.625 | 0.0021512 | 2.70E-20 | 1.94E-04 | 85.20 |
| Apolipoprotein B | rs35081008 | C | T | 0.0311931 | 0.852 | 0.0029032 | 6.30E-27 | 2.63E-04 | 115.44 |
| Apolipoprotein B | rs35882350 | A | G | -0.0134361 | 0.739 | 0.002368 | 1.40E-08 | 7.33E-05 | 32.19 |
| Apolipoprotein B | rs3764261 | C | A | 0.0479089 | 0.676 | 0.0022225 | 4.60E-103 | 1.06E-03 | 464.68 |
| Apolipoprotein B | rs3780181 | A | G | 0.0258993 | 0.932 | 0.0041728 | 5.40E-10 | 8.77E-05 | 38.52 |
| Apolipoprotein B | rs3821838 | T | C | -0.0275669 | 0.053 | 0.0046529 | 3.10E-09 | 7.99E-05 | 35.10 |
| Apolipoprotein B | rs3822855 | G | T | -0.0149153 | 0.598 | 0.0021181 | 1.90E-12 | 1.13E-04 | 49.59 |
| Apolipoprotein B | rs4299376 | G | T | 0.0481043 | 0.323 | 0.0022147 | 1.30E-104 | 1.07E-03 | 471.79 |
| Apolipoprotein B | rs4470903 | C | G | -0.0345683 | 0.785 | 0.0025347 | 2.40E-42 | 4.23E-04 | 185.99 |
| Apolipoprotein B | rs45537841 | C | T | 0.0159262 | 0.819 | 0.002706 | 4.00E-09 | 7.89E-05 | 34.64 |
| Apolipoprotein B | rs4671050 | G | T | 0.0196869 | 0.684 | 0.0022312 | 1.10E-18 | 1.77E-04 | 77.85 |
| Apolipoprotein B | rs472495 | G | T | -0.0408252 | 0.351 | 0.0021731 | 9.80E-79 | 8.03E-04 | 352.93 |
| Apolipoprotein B | rs473224 | T | G | 0.0223307 | 0.146 | 0.0029851 | 7.40E-14 | 1.27E-04 | 55.96 |
| Apolipoprotein B | rs4782568 | C | G | 0.0182176 | 0.549 | 0.0021021 | 4.50E-18 | 1.71E-04 | 75.10 |
| Apolipoprotein B | rs4965894 | T | C | 0.0116391 | 0.597 | 0.0021284 | 4.50E-08 | 6.81E-05 | 29.90 |
| Apolipoprotein B | rs531660643 | G | T | 0.457107 | 0.977 | 0.0070891 | 1.00E-200 | 9.38E-03 | 4157.68 |
| Apolipoprotein B | rs546240 | C | T | 0.0119372 | 0.38 | 0.0021473 | 2.70E-08 | 7.04E-05 | 30.90 |
| Apolipoprotein B | rs556107 | C | T | -0.0359348 | 0.477 | 0.0020807 | 7.90E-67 | 6.79E-04 | 298.26 |
| Apolipoprotein B | rs55714927 | C | T | 0.0320541 | 0.81 | 0.0026487 | 1.00E-33 | 3.33E-04 | 146.46 |
| Apolipoprotein B | rs55831924 | C | T | -0.0182534 | 0.639 | 0.0021769 | 5.10E-17 | 1.60E-04 | 70.31 |
| Apolipoprotein B | rs55921103 | G | T | -0.0140216 | 0.351 | 0.0021923 | 1.60E-10 | 9.31E-05 | 40.91 |
| Apolipoprotein B | rs56402930 | A | G | 0.0196542 | 0.093 | 0.00359 | 4.40E-08 | 6.82E-05 | 29.97 |
| Apolipoprotein B | rs581080 | G | C | -0.0185605 | 0.181 | 0.0027033 | 6.60E-12 | 1.07E-04 | 47.14 |
| Apolipoprotein B | rs58148580 | C | T | -0.0214703 | 0.89 | 0.0033224 | 1.00E-10 | 9.51E-05 | 41.76 |
| Apolipoprotein B | rs59328596 | G | A | 0.0211048 | 0.852 | 0.0029238 | 5.30E-13 | 1.19E-04 | 52.10 |
| Apolipoprotein B | rs597808 | A | G | -0.021719 | 0.484 | 0.002086 | 2.20E-25 | 2.47E-04 | 108.40 |
| Apolipoprotein B | rs6050464 | C | A | -0.0156065 | 0.511 | 0.002078 | 5.90E-14 | 1.28E-04 | 56.40 |
| Apolipoprotein B | rs6073958 | T | C | -0.0424491 | 0.801 | 0.0026073 | 1.40E-59 | 6.03E-04 | 265.06 |
| Apolipoprotein B | rs61754230 | C | T | -0.0427783 | 0.98 | 0.0074635 | 9.90E-09 | 7.48E-05 | 32.85 |
| Apolipoprotein B | rs61775180 | C | T | 0.0286213 | 0.58 | 0.002103 | 3.50E-42 | 4.22E-04 | 185.23 |
| Apolipoprotein B | rs62119267 | A | C | 0.334491 | 0.978 | 0.007085 | 1.00E-200 | 5.05E-03 | 2228.89 |
| Apolipoprotein B | rs62120573 | T | C | -0.0321084 | 0.93 | 0.0040389 | 1.90E-15 | 1.44E-04 | 63.20 |
| Apolipoprotein B | rs62122481 | C | A | -0.0839859 | 0.623 | 0.0021463 | 1.00E-200 | 3.47E-03 | 1531.16 |
| Apolipoprotein B | rs6426328 | G | T | -0.0120957 | 0.51 | 0.0020746 | 5.50E-09 | 7.74E-05 | 33.99 |
| Apolipoprotein B | rs6475606 | C | T | 0.0173839 | 0.516 | 0.0020786 | 6.10E-17 | 1.59E-04 | 69.94 |
| Apolipoprotein B | rs6560499 | G | A | 0.0122165 | 0.424 | 0.0021165 | 7.80E-09 | 7.58E-05 | 33.32 |
| Apolipoprotein B | rs6602909 | T | C | -0.0215663 | 0.672 | 0.0022218 | 2.80E-22 | 2.14E-04 | 94.22 |
| Apolipoprotein B | rs6657811 | A | T | 0.127747 | 0.87 | 0.0030877 | 1.00E-200 | 3.88E-03 | 1711.74 |
| Apolipoprotein B | rs6689611 | G | A | 0.132132 | 0.988 | 0.0095088 | 6.70E-44 | 4.39E-04 | 193.09 |
| Apolipoprotein B | rs67038483 | C | T | -0.0275587 | 0.955 | 0.0050231 | 4.10E-08 | 6.85E-05 | 30.10 |
| Apolipoprotein B | rs6709904 | A | G | 0.0402501 | 0.887 | 0.0032814 | 1.40E-34 | 3.42E-04 | 150.46 |
| Apolipoprotein B | rs6874202 | T | C | -0.0312735 | 0.366 | 0.0021569 | 1.20E-47 | 4.78E-04 | 210.23 |
| Apolipoprotein B | rs7012637 | G | A | -0.0198643 | 0.526 | 0.0020965 | 2.70E-21 | 2.04E-04 | 89.78 |
| Apolipoprotein B | rs704 | G | A | -0.0180904 | 0.524 | 0.0020811 | 3.50E-18 | 1.72E-04 | 75.57 |
| Apolipoprotein B | rs7108486 | T | C | 0.0433041 | 0.976 | 0.0069002 | 3.50E-10 | 8.97E-05 | 39.38 |
| Apolipoprotein B | rs71311871 | A | G | 0.0264323 | 0.917 | 0.003774 | 2.50E-12 | 1.12E-04 | 49.05 |
| Apolipoprotein B | rs719148 | G | A | 0.0161997 | 0.224 | 0.0024801 | 6.50E-11 | 9.71E-05 | 42.66 |
| Apolipoprotein B | rs7249565 | G | A | -0.0145421 | 0.585 | 0.002085 | 3.10E-12 | 1.11E-04 | 48.65 |
| Apolipoprotein B | rs7260871 | A | G | 0.0323484 | 0.963 | 0.0057211 | 1.60E-08 | 7.28E-05 | 31.97 |
| Apolipoprotein B | rs72631343 | C | G | 0.0278868 | 0.871 | 0.0031022 | 2.50E-19 | 1.84E-04 | 80.81 |
| Apolipoprotein B | rs72638977 | A | G | 0.0316061 | 0.966 | 0.0057604 | 4.10E-08 | 6.85E-05 | 30.10 |
| Apolipoprotein B | rs72823020 | T | A | 0.0227752 | 0.87 | 0.0031033 | 2.20E-13 | 1.23E-04 | 53.86 |
| Apolipoprotein B | rs73025516 | A | G | 0.0296434 | 0.955 | 0.0050374 | 4.00E-09 | 7.88E-05 | 34.63 |
| Apolipoprotein B | rs73075609 | C | T | -0.0481624 | 0.973 | 0.0064829 | 1.10E-13 | 1.26E-04 | 55.19 |
| Apolipoprotein B | rs7601153 | C | G | 0.012034 | 0.603 | 0.0021172 | 1.30E-08 | 7.36E-05 | 32.31 |
| Apolipoprotein B | rs7603427 | C | T | -0.0135798 | 0.467 | 0.0020799 | 6.60E-11 | 9.71E-05 | 42.63 |
| Apolipoprotein B | rs76186504 | C | T | 0.116409 | 0.975 | 0.006626 | 4.30E-69 | 7.02E-04 | 308.65 |
| Apolipoprotein B | rs7734476 | G | A | -0.0198862 | 0.45 | 0.0020883 | 1.70E-21 | 2.06E-04 | 90.69 |
| Apolipoprotein B | rs7746081 | G | A | 0.0227371 | 0.696 | 0.0022601 | 8.30E-24 | 2.30E-04 | 101.21 |
| Apolipoprotein B | rs77542162 | A | G | -0.115477 | 0.978 | 0.0070232 | 9.50E-61 | 6.15E-04 | 270.34 |
| Apolipoprotein B | rs79220007 | T | C | 0.0539299 | 0.924 | 0.0039107 | 2.90E-43 | 4.33E-04 | 190.18 |
| Apolipoprotein B | rs79931565 | A | G | -0.0265471 | 0.933 | 0.0041582 | 1.70E-10 | 9.28E-05 | 40.76 |
| Apolipoprotein B | rs80276949 | G | A | -0.0420929 | 0.977 | 0.0070019 | 1.80E-09 | 8.23E-05 | 36.14 |
| Apolipoprotein B | rs8107974 | A | T | 0.0909955 | 0.924 | 0.0038857 | 2.80E-121 | 1.25E-03 | 548.41 |
| Apolipoprotein B | rs9297994 | G | A | 0.0291068 | 0.335 | 0.0022023 | 7.00E-40 | 3.98E-04 | 174.68 |
| Apolipoprotein B | rs9471975 | T | C | 0.0199706 | 0.417 | 0.0021069 | 2.60E-21 | 2.05E-04 | 89.84 |
| Apolipoprotein B | rs9491697 | A | G | -0.0179089 | 0.537 | 0.0020848 | 8.70E-18 | 1.68E-04 | 73.80 |
| Apolipoprotein B | rs9496567 | G | A | 0.0191799 | 0.756 | 0.002425 | 2.60E-15 | 1.42E-04 | 62.56 |
| Apolipoprotein B | rs9616822 | G | A | -0.0158077 | 0.649 | 0.0021815 | 4.30E-13 | 1.20E-04 | 52.51 |
| Apolipoprotein B | rs964184 | G | C | 0.0764264 | 0.133 | 0.0030588 | 8.70E-138 | 1.42E-03 | 624.30 |
| Apolipoprotein B | rs969075 | T | C | -0.0140201 | 0.335 | 0.0022154 | 2.50E-10 | 9.12E-05 | 40.05 |
| Apolipoprotein B | rs9832727 | C | G | 0.0149953 | 0.66 | 0.0021981 | 9.00E-12 | 1.06E-04 | 46.54 |
| Apolipoprotein B | rs9834932 | A | G | 0.0318788 | 0.911 | 0.003654 | 2.70E-18 | 1.73E-04 | 76.11 |
| Apolipoprotein B | rs9884390 | T | C | -0.0231946 | 0.766 | 0.0024848 | 1.00E-20 | 1.98E-04 | 87.13 |
| Triglycerides | rs1009360 | T | C | 0.0185443 | 0.581 | 0.0020115 | 3.00E-20 | 1.93E-04 | 84.99 |
| Triglycerides | rs10152471 | G | A | 0.0134847 | 0.611 | 0.0020514 | 4.90E-11 | 9.80E-05 | 43.21 |
| Triglycerides | rs10172544 | C | A | 0.0114209 | 0.591 | 0.0020191 | 1.50E-08 | 7.25E-05 | 31.99 |
| Triglycerides | rs10242866 | C | T | -0.0155819 | 0.601 | 0.0020324 | 1.80E-14 | 1.33E-04 | 58.78 |
| Triglycerides | rs10422861 | C | T | 0.0194396 | 0.335 | 0.0021105 | 3.20E-20 | 1.92E-04 | 84.84 |
| Triglycerides | rs1045241 | C | T | 0.0208107 | 0.729 | 0.002251 | 2.30E-20 | 1.94E-04 | 85.47 |
| Triglycerides | rs10750766 | C | A | -0.0195875 | 0.29 | 0.0021913 | 3.90E-19 | 1.81E-04 | 79.90 |
| Triglycerides | rs10775406 | A | G | -0.0204601 | 0.24 | 0.00233 | 1.60E-18 | 1.75E-04 | 77.11 |
| Triglycerides | rs1077835 | A | G | -0.0473972 | 0.78 | 0.0024088 | 3.40E-86 | 8.77E-04 | 387.17 |
| Triglycerides | rs10811662 | G | A | 0.0153267 | 0.827 | 0.0026338 | 5.90E-09 | 7.68E-05 | 33.86 |
| Triglycerides | rs10822163 | C | G | 0.0318329 | 0.527 | 0.0019941 | 2.30E-57 | 5.77E-04 | 254.83 |
| Triglycerides | rs10899490 | C | T | 0.0170895 | 0.839 | 0.0026983 | 2.40E-10 | 9.09E-05 | 40.11 |
| Triglycerides | rs11030107 | A | G | -0.0161703 | 0.739 | 0.002261 | 8.60E-13 | 1.16E-04 | 51.15 |
| Triglycerides | rs11078597 | T | C | -0.019015 | 0.813 | 0.0025559 | 1.00E-13 | 1.25E-04 | 55.35 |
| Triglycerides | rs11100083 | T | C | 0.0160934 | 0.774 | 0.0023801 | 1.40E-11 | 1.04E-04 | 45.72 |
| Triglycerides | rs11118310 | A | T | -0.0192039 | 0.407 | 0.0020238 | 2.30E-21 | 2.04E-04 | 90.04 |
| Triglycerides | rs11122450 | T | G | 0.0482838 | 0.388 | 0.002041 | 9.90E-124 | 1.27E-03 | 559.66 |
| Triglycerides | rs11206374 | G | A | -0.0249878 | 0.775 | 0.0023801 | 8.80E-26 | 2.50E-04 | 110.22 |
| Triglycerides | rs11240358 | G | A | -0.013425 | 0.606 | 0.0020332 | 4.00E-11 | 9.88E-05 | 43.60 |
| Triglycerides | rs112424890 | C | T | -0.0172658 | 0.822 | 0.0026092 | 3.70E-11 | 9.93E-05 | 43.79 |
| Triglycerides | rs1126673 | C | T | -0.013906 | 0.303 | 0.0021673 | 1.40E-10 | 9.33E-05 | 41.17 |
| Triglycerides | rs1133400 | A | G | -0.0137577 | 0.78 | 0.0024019 | 1.00E-08 | 7.44E-05 | 32.81 |
| Triglycerides | rs113344423 | G | A | -0.0429482 | 0.94 | 0.0042969 | 1.60E-23 | 2.26E-04 | 99.90 |
| Triglycerides | rs114165349 | G | C | -0.0822141 | 0.977 | 0.006648 | 4.00E-35 | 3.47E-04 | 152.93 |
| Triglycerides | rs1152847 | G | A | 0.0116004 | 0.647 | 0.0020853 | 2.70E-08 | 7.02E-05 | 30.95 |
| Triglycerides | rs11657201 | A | G | -0.01297 | 0.757 | 0.0023242 | 2.40E-08 | 7.06E-05 | 31.14 |
| Triglycerides | rs11664106 | A | T | 0.0126383 | 0.626 | 0.0021119 | 2.20E-09 | 8.12E-05 | 35.81 |
| Triglycerides | rs116843064 | G | A | 0.226178 | 0.981 | 0.0072231 | 1.00E-200 | 2.22E-03 | 980.50 |
| Triglycerides | rs11722924 | G | C | -0.0128738 | 0.465 | 0.0019945 | 1.10E-10 | 9.45E-05 | 41.66 |
| Triglycerides | rs117291242 | C | T | -0.0300381 | 0.963 | 0.0052917 | 1.40E-08 | 7.31E-05 | 32.22 |
| Triglycerides | rs11781692 | C | A | -0.0749604 | 0.985 | 0.008335 | 2.40E-19 | 1.83E-04 | 80.88 |
| Triglycerides | rs12185242 | A | C | -0.0173785 | 0.545 | 0.0020026 | 4.00E-18 | 1.71E-04 | 75.31 |
| Triglycerides | rs12446515 | C | T | 0.0334693 | 0.677 | 0.0021397 | 3.80E-55 | 5.54E-04 | 244.67 |
| Triglycerides | rs12504746 | C | T | 0.0151583 | 0.807 | 0.002526 | 2.00E-09 | 8.17E-05 | 36.01 |
| Triglycerides | rs12530679 | A | G | 0.0123041 | 0.515 | 0.0020204 | 1.10E-09 | 8.41E-05 | 37.09 |
| Triglycerides | rs12600110 | T | C | 0.0146778 | 0.624 | 0.0020556 | 9.30E-13 | 1.16E-04 | 50.99 |
| Triglycerides | rs12610709 | G | A | -0.0222318 | 0.829 | 0.0026507 | 5.00E-17 | 1.59E-04 | 70.34 |
| Triglycerides | rs12880341 | T | C | -0.0208365 | 0.841 | 0.0027414 | 2.90E-14 | 1.31E-04 | 57.77 |
| Triglycerides | rs1292065 | C | G | 0.0137989 | 0.291 | 0.0021906 | 3.00E-10 | 9.00E-05 | 39.68 |
| Triglycerides | rs12926107 | A | G | -0.0128688 | 0.545 | 0.002005 | 1.40E-10 | 9.34E-05 | 41.20 |
| Triglycerides | rs12928099 | C | A | 0.0280607 | 0.704 | 0.0021852 | 9.60E-38 | 3.74E-04 | 164.90 |
| Triglycerides | rs13066793 | A | G | 0.0224735 | 0.91 | 0.003479 | 1.00E-10 | 9.46E-05 | 41.73 |
| Triglycerides | rs13108218 | A | G | 0.0305943 | 0.385 | 0.0020618 | 8.20E-50 | 4.99E-04 | 220.19 |
| Triglycerides | rs1316753 | G | C | 0.0145496 | 0.606 | 0.0020333 | 8.30E-13 | 1.16E-04 | 51.20 |
| Triglycerides | rs13269725 | A | G | -0.034783 | 0.922 | 0.003687 | 4.00E-21 | 2.02E-04 | 89.00 |
| Triglycerides | rs13389219 | C | T | 0.0374229 | 0.607 | 0.0020307 | 7.70E-76 | 7.69E-04 | 339.61 |
| Triglycerides | rs1340819 | A | C | 0.0123182 | 0.655 | 0.0020984 | 4.40E-09 | 7.81E-05 | 34.46 |
| Triglycerides | rs134551 | C | T | 0.0117186 | 0.665 | 0.0021148 | 3.00E-08 | 6.96E-05 | 30.70 |
| Triglycerides | rs1347188 | A | G | -0.0139717 | 0.754 | 0.002318 | 1.70E-09 | 8.24E-05 | 36.33 |
| Triglycerides | rs1365297 | A | G | 0.0183261 | 0.816 | 0.002566 | 9.20E-13 | 1.16E-04 | 51.00 |
| Triglycerides | rs138191773 | G | A | 0.047771 | 0.983 | 0.0079329 | 1.70E-09 | 8.22E-05 | 36.26 |
| Triglycerides | rs138751626 | A | T | -0.0284208 | 0.924 | 0.0037566 | 3.90E-14 | 1.30E-04 | 57.24 |
| Triglycerides | rs139386986 | C | T | 0.0215163 | 0.908 | 0.0035671 | 1.60E-09 | 8.25E-05 | 36.38 |
| Triglycerides | rs139453187 | T | C | -0.0231995 | 0.931 | 0.0039208 | 3.30E-09 | 7.94E-05 | 35.01 |
| Triglycerides | rs139974673 | T | C | -0.142694 | 0.974 | 0.0062498 | 2.20E-115 | 1.18E-03 | 521.29 |
| Triglycerides | rs140107293 | A | G | 0.0225703 | 0.845 | 0.0027579 | 2.70E-16 | 1.52E-04 | 66.97 |
| Triglycerides | rs143076454 | G | A | -0.0403694 | 0.981 | 0.0073668 | 4.30E-08 | 6.81E-05 | 30.03 |
| Triglycerides | rs147011441 | G | A | -0.0424968 | 0.975 | 0.0066002 | 1.20E-10 | 9.40E-05 | 41.46 |
| Triglycerides | rs149142833 | C | T | -0.0174178 | 0.844 | 0.00277 | 3.20E-10 | 8.96E-05 | 39.54 |
| Triglycerides | rs150423652 | G | T | -0.286267 | 0.993 | 0.0123145 | 1.50E-119 | 1.22E-03 | 540.39 |
| Triglycerides | rs150555490 | C | T | 0.0387462 | 0.943 | 0.0042831 | 1.50E-19 | 1.86E-04 | 81.84 |
| Triglycerides | rs150564454 | G | A | 0.101886 | 0.988 | 0.0096492 | 4.60E-26 | 2.53E-04 | 111.49 |
| Triglycerides | rs151235402 | C | T | -0.0529665 | 0.984 | 0.0081661 | 8.80E-11 | 9.54E-05 | 42.07 |
| Triglycerides | rs1532085 | A | G | 0.0308141 | 0.386 | 0.0020467 | 3.20E-51 | 5.14E-04 | 226.67 |
| Triglycerides | rs1567353 | C | G | -0.015087 | 0.692 | 0.0021674 | 3.40E-12 | 1.10E-04 | 48.45 |
| Triglycerides | rs17326656 | G | T | -0.0176669 | 0.762 | 0.0023379 | 4.10E-14 | 1.29E-04 | 57.11 |
| Triglycerides | rs174566 | A | G | -0.0486123 | 0.65 | 0.0020836 | 2.10E-120 | 1.23E-03 | 544.36 |
| Triglycerides | rs1799831 | C | T | -0.0245722 | 0.844 | 0.0027444 | 3.40E-19 | 1.82E-04 | 80.17 |
| Triglycerides | rs1800978 | C | G | 0.0267606 | 0.876 | 0.0030318 | 1.10E-18 | 1.77E-04 | 77.91 |
| Triglycerides | rs1801689 | A | C | 0.0662731 | 0.969 | 0.0057876 | 2.30E-30 | 2.97E-04 | 131.12 |
| Triglycerides | rs1835346 | A | G | 0.0388839 | 0.976 | 0.0065584 | 3.10E-09 | 7.97E-05 | 35.15 |
| Triglycerides | rs186696265 | C | T | 0.104035 | 0.985 | 0.0082999 | 4.80E-36 | 3.56E-04 | 157.11 |
| Triglycerides | rs188247550 | C | T | 0.133886 | 0.987 | 0.0091478 | 1.70E-48 | 4.85E-04 | 214.21 |
| Triglycerides | rs193735 | G | A | -0.0327254 | 0.963 | 0.0053086 | 7.10E-10 | 8.62E-05 | 38.00 |
| Triglycerides | rs1938566 | C | T | 0.0213189 | 0.165 | 0.0026754 | 1.60E-15 | 1.44E-04 | 63.50 |
| Triglycerides | rs200293726 | A | T | -0.0128609 | 0.694 | 0.0021704 | 3.10E-09 | 7.96E-05 | 35.11 |
| Triglycerides | rs2035816 | A | G | 0.0280325 | 0.916 | 0.0035886 | 5.60E-15 | 1.38E-04 | 61.02 |
| Triglycerides | rs2054067 | A | T | 0.0113173 | 0.63 | 0.0020689 | 4.50E-08 | 6.78E-05 | 29.92 |
| Triglycerides | rs2068888 | G | A | 0.0318472 | 0.549 | 0.0019995 | 4.10E-57 | 5.75E-04 | 253.69 |
| Triglycerides | rs2070341 | C | T | -0.0112103 | 0.397 | 0.0020378 | 3.80E-08 | 6.86E-05 | 30.26 |
| Triglycerides | rs2071887 | T | A | -0.0160194 | 0.655 | 0.0021004 | 2.40E-14 | 1.32E-04 | 58.17 |
| Triglycerides | rs2081687 | T | C | 0.0262292 | 0.337 | 0.002095 | 5.80E-36 | 3.55E-04 | 156.74 |
| Triglycerides | rs2110690 | A | G | -0.0108794 | 0.495 | 0.001986 | 4.30E-08 | 6.80E-05 | 30.01 |
| Triglycerides | rs2131311 | A | G | 0.0123261 | 0.285 | 0.0022316 | 3.30E-08 | 6.92E-05 | 30.51 |
| Triglycerides | rs2131919 | A | G | -0.0172675 | 0.836 | 0.0026887 | 1.30E-10 | 9.35E-05 | 41.24 |
| Triglycerides | rs213494 | C | T | -0.0155476 | 0.352 | 0.0020795 | 7.60E-14 | 1.27E-04 | 55.90 |
| Triglycerides | rs2187114 | G | A | 0.0185147 | 0.899 | 0.0033096 | 2.20E-08 | 7.10E-05 | 31.30 |
| Triglycerides | rs2194411 | G | A | 0.0171285 | 0.872 | 0.003013 | 1.30E-08 | 7.33E-05 | 32.32 |
| Triglycerides | rs2237029 | G | A | 0.0139601 | 0.399 | 0.0020438 | 8.50E-12 | 1.06E-04 | 46.65 |
| Triglycerides | rs2240466 | G | A | 0.122504 | 0.877 | 0.0030239 | 1.00E-200 | 3.71E-03 | 1641.21 |
| Triglycerides | rs2240533 | T | C | 0.0128299 | 0.69 | 0.0021562 | 2.70E-09 | 8.03E-05 | 35.41 |
| Triglycerides | rs2267373 | C | T | -0.021551 | 0.419 | 0.002025 | 1.90E-26 | 2.57E-04 | 113.26 |
| Triglycerides | rs2382825 | C | T | 0.0131222 | 0.377 | 0.0020529 | 1.60E-10 | 9.26E-05 | 40.86 |
| Triglycerides | rs2416759 | G | A | -0.0120803 | 0.303 | 0.0021778 | 2.90E-08 | 6.98E-05 | 30.77 |
| Triglycerides | rs2487294 | G | T | -0.0183644 | 0.277 | 0.0022248 | 1.50E-16 | 1.54E-04 | 68.13 |
| Triglycerides | rs2499797 | G | A | 0.0153808 | 0.165 | 0.0026788 | 9.40E-09 | 7.47E-05 | 32.97 |
| Triglycerides | rs2699805 | G | A | 0.0203214 | 0.6 | 0.0020411 | 2.40E-23 | 2.25E-04 | 99.12 |
| Triglycerides | rs2705616 | C | G | -0.0112493 | 0.469 | 0.0019965 | 1.80E-08 | 7.20E-05 | 31.75 |
| Triglycerides | rs2773469 | A | G | 0.0186292 | 0.267 | 0.0022583 | 1.60E-16 | 1.54E-04 | 68.05 |
| Triglycerides | rs2812208 | G | C | 0.0488932 | 0.979 | 0.0069757 | 2.40E-12 | 1.11E-04 | 49.13 |
| Triglycerides | rs28577186 | G | A | 0.0160261 | 0.335 | 0.0021205 | 4.10E-14 | 1.30E-04 | 57.12 |
| Triglycerides | rs28624578 | T | C | -0.0149261 | 0.829 | 0.0026559 | 1.90E-08 | 7.16E-05 | 31.58 |
| Triglycerides | rs2925979 | T | C | 0.0321923 | 0.3 | 0.0021748 | 1.40E-49 | 4.97E-04 | 219.10 |
| Triglycerides | rs293561 | T | G | -0.0116655 | 0.637 | 0.0020887 | 2.30E-08 | 7.07E-05 | 31.19 |
| Triglycerides | rs2943645 | C | T | -0.0402176 | 0.353 | 0.0020753 | 1.10E-83 | 8.51E-04 | 375.57 |
| Triglycerides | rs2980755 | A | G | 0.0248515 | 0.542 | 0.0020032 | 2.40E-35 | 3.49E-04 | 153.91 |
| Triglycerides | rs308 | T | G | 0.159426 | 0.979 | 0.006997 | 6.50E-115 | 1.18E-03 | 519.15 |
| Triglycerides | rs3103310 | A | G | -0.0204555 | 0.758 | 0.0023707 | 6.20E-18 | 1.69E-04 | 74.45 |
| Triglycerides | rs320369 | A | G | 0.0122743 | 0.317 | 0.0021496 | 1.10E-08 | 7.39E-05 | 32.61 |
| Triglycerides | rs325485 | A | G | 0.0117345 | 0.397 | 0.0020441 | 9.40E-09 | 7.47E-05 | 32.96 |
| Triglycerides | rs326222 | T | C | -0.0252299 | 0.302 | 0.0021608 | 1.70E-31 | 3.09E-04 | 136.33 |
| Triglycerides | rs343 | C | A | 0.141358 | 0.917 | 0.003617 | 1.00E-200 | 3.45E-03 | 1527.38 |
| Triglycerides | rs35763453 | T | C | -0.0282519 | 0.941 | 0.0043591 | 9.10E-11 | 9.52E-05 | 42.00 |
| Triglycerides | rs36043408 | G | A | 0.0128509 | 0.498 | 0.0019891 | 1.00E-10 | 9.46E-05 | 41.74 |
| Triglycerides | rs36061954 | C | T | -0.0119277 | 0.601 | 0.002022 | 3.70E-09 | 7.89E-05 | 34.80 |
| Triglycerides | rs3731696 | A | G | -0.0218418 | 0.879 | 0.0030373 | 6.40E-13 | 1.17E-04 | 51.71 |
| Triglycerides | rs37538 | G | C | 0.0144558 | 0.398 | 0.0020411 | 1.40E-12 | 1.14E-04 | 50.16 |
| Triglycerides | rs3758413 | T | C | -0.0113883 | 0.582 | 0.0020213 | 1.80E-08 | 7.20E-05 | 31.74 |
| Triglycerides | rs3775228 | C | T | -0.0339811 | 0.6 | 0.0020396 | 2.50E-62 | 6.29E-04 | 277.57 |
| Triglycerides | rs3814883 | C | T | -0.0147973 | 0.517 | 0.0020001 | 1.40E-13 | 1.24E-04 | 54.73 |
| Triglycerides | rs3820897 | T | C | -0.0197325 | 0.18 | 0.0026078 | 3.80E-14 | 1.30E-04 | 57.26 |
| Triglycerides | rs3826043 | C | T | 0.0122106 | 0.571 | 0.0020285 | 1.70E-09 | 8.22E-05 | 36.23 |
| Triglycerides | rs3936511 | A | G | -0.0459535 | 0.808 | 0.0025261 | 6.00E-74 | 7.50E-04 | 330.93 |
| Triglycerides | rs394872 | C | T | -0.0112774 | 0.464 | 0.0020064 | 1.90E-08 | 7.16E-05 | 31.59 |
| Triglycerides | rs4128205 | A | C | -0.0116817 | 0.491 | 0.0019983 | 5.00E-09 | 7.75E-05 | 34.17 |
| Triglycerides | rs4134963 | C | T | 0.0188136 | 0.81 | 0.0025469 | 1.50E-13 | 1.24E-04 | 54.56 |
| Triglycerides | rs41785 | C | A | 0.0148568 | 0.583 | 0.0020185 | 1.80E-13 | 1.23E-04 | 54.17 |
| Triglycerides | rs4382584 | G | A | -0.0130445 | 0.726 | 0.0022394 | 5.70E-09 | 7.69E-05 | 33.93 |
| Triglycerides | rs4662414 | A | G | 0.0119481 | 0.553 | 0.0019954 | 2.10E-09 | 8.13E-05 | 35.85 |
| Triglycerides | rs4665972 | T | C | 0.100204 | 0.395 | 0.0020394 | 1.00E-200 | 5.44E-03 | 2414.07 |
| Triglycerides | rs4675812 | G | A | 0.0143767 | 0.412 | 0.0020154 | 9.80E-13 | 1.15E-04 | 50.89 |
| Triglycerides | rs4722551 | T | C | 0.0372104 | 0.841 | 0.002721 | 1.40E-42 | 4.24E-04 | 187.01 |
| Triglycerides | rs4731701 | C | T | 0.0328926 | 0.507 | 0.0019928 | 3.30E-61 | 6.17E-04 | 272.45 |
| Triglycerides | rs4760254 | G | C | 0.027987 | 0.761 | 0.002329 | 2.90E-33 | 3.27E-04 | 144.40 |
| Triglycerides | rs4761234 | T | C | 0.0138507 | 0.516 | 0.0019973 | 4.10E-12 | 1.09E-04 | 48.09 |
| Triglycerides | rs480823 | T | C | -0.155635 | 0.921 | 0.0037264 | 1.00E-200 | 3.94E-03 | 1744.35 |
| Triglycerides | rs483082 | G | T | -0.0858847 | 0.765 | 0.002349 | 1.00E-200 | 3.02E-03 | 1336.85 |
| Triglycerides | rs490972 | G | A | -0.0142281 | 0.531 | 0.0019978 | 1.10E-12 | 1.15E-04 | 50.72 |
| Triglycerides | rs4909945 | T | C | -0.0130351 | 0.31 | 0.0021459 | 1.20E-09 | 8.37E-05 | 36.90 |
| Triglycerides | rs4930724 | T | C | 0.0261639 | 0.668 | 0.0021146 | 3.70E-35 | 3.47E-04 | 153.09 |
| Triglycerides | rs4969179 | T | G | 0.017794 | 0.396 | 0.0020401 | 2.70E-18 | 1.72E-04 | 76.07 |
| Triglycerides | rs4976033 | A | G | -0.0179116 | 0.598 | 0.0020554 | 2.90E-18 | 1.72E-04 | 75.94 |
| Triglycerides | rs498475 | G | A | 0.0116865 | 0.368 | 0.0020705 | 1.70E-08 | 7.22E-05 | 31.86 |
| Triglycerides | rs499293 | G | A | 0.0118005 | 0.342 | 0.0020928 | 1.70E-08 | 7.21E-05 | 31.80 |
| Triglycerides | rs5112 | C | G | -0.0682951 | 0.467 | 0.002138 | 1.00E-200 | 2.31E-03 | 1020.43 |
| Triglycerides | rs535241194 | A | G | -0.020829 | 0.786 | 0.0024642 | 2.90E-17 | 1.62E-04 | 71.45 |
| Triglycerides | rs55646464 | G | T | -0.012364 | 0.7 | 0.002174 | 1.30E-08 | 7.33E-05 | 32.34 |
| Triglycerides | rs55737395 | G | A | 0.0133153 | 0.665 | 0.0021185 | 3.30E-10 | 8.96E-05 | 39.50 |
| Triglycerides | rs55767272 | A | C | 0.0278189 | 0.935 | 0.0040433 | 6.00E-12 | 1.07E-04 | 47.34 |
| Triglycerides | rs55966194 | C | G | 0.0176844 | 0.718 | 0.0022164 | 1.50E-15 | 1.44E-04 | 63.66 |
| Triglycerides | rs56321085 | G | A | -0.0196204 | 0.916 | 0.0035944 | 4.80E-08 | 6.76E-05 | 29.80 |
| Triglycerides | rs56902258 | T | A | 0.0150826 | 0.804 | 0.0025174 | 2.10E-09 | 8.14E-05 | 35.90 |
| Triglycerides | rs57074291 | C | G | 0.0141049 | 0.739 | 0.0022594 | 4.30E-10 | 8.84E-05 | 38.97 |
| Triglycerides | rs581080 | G | C | -0.0175184 | 0.181 | 0.002589 | 1.30E-11 | 1.04E-04 | 45.78 |
| Triglycerides | rs58542926 | C | T | 0.102936 | 0.925 | 0.0037876 | 1.20E-162 | 1.67E-03 | 738.60 |
| Triglycerides | rs60610697 | T | G | -0.0179778 | 0.782 | 0.0024475 | 2.10E-13 | 1.22E-04 | 53.95 |
| Triglycerides | rs6073958 | T | C | -0.0555219 | 0.801 | 0.0024979 | 1.90E-109 | 1.12E-03 | 494.06 |
| Triglycerides | rs61729990 | C | A | 0.0559706 | 0.982 | 0.0075349 | 1.10E-13 | 1.25E-04 | 55.18 |
| Triglycerides | rs61780049 | A | G | -0.0154776 | 0.851 | 0.0027978 | 3.20E-08 | 6.94E-05 | 30.60 |
| Triglycerides | rs61830291 | A | C | -0.0289356 | 0.904 | 0.0033852 | 1.30E-17 | 1.66E-04 | 73.06 |
| Triglycerides | rs61885960 | T | A | 0.0317776 | 0.946 | 0.0044377 | 8.00E-13 | 1.16E-04 | 51.28 |
| Triglycerides | rs61905078 | A | C | -0.199765 | 0.926 | 0.0038018 | 1.00E-200 | 6.22E-03 | 2761.01 |
| Triglycerides | rs61993685 | T | C | 0.0234892 | 0.924 | 0.0037467 | 3.60E-10 | 8.91E-05 | 39.30 |
| Triglycerides | rs62118471 | T | C | -0.0425822 | 0.973 | 0.0064818 | 5.00E-11 | 9.79E-05 | 43.16 |
| Triglycerides | rs62271373 | T | A | -0.0421729 | 0.94 | 0.0042735 | 5.70E-23 | 2.21E-04 | 97.39 |
| Triglycerides | rs62397245 | C | G | -0.0149698 | 0.778 | 0.0024006 | 4.50E-10 | 8.82E-05 | 38.89 |
| Triglycerides | rs62427982 | C | T | 0.0130026 | 0.678 | 0.0021358 | 1.10E-09 | 8.40E-05 | 37.06 |
| Triglycerides | rs62473520 | T | C | 0.0211243 | 0.922 | 0.003808 | 2.90E-08 | 6.98E-05 | 30.77 |
| Triglycerides | rs6486122 | C | T | -0.019768 | 0.311 | 0.0021441 | 3.00E-20 | 1.93E-04 | 85.00 |
| Triglycerides | rs6506033 | C | T | 0.0233834 | 0.927 | 0.0038458 | 1.20E-09 | 8.38E-05 | 36.97 |
| Triglycerides | rs6517522 | T | C | 0.0129131 | 0.502 | 0.001996 | 9.80E-11 | 9.49E-05 | 41.86 |
| Triglycerides | rs6572807 | A | G | -0.0125047 | 0.733 | 0.002253 | 2.90E-08 | 6.98E-05 | 30.80 |
| Triglycerides | rs6700266 | G | A | 0.0125681 | 0.658 | 0.002097 | 2.10E-09 | 8.14E-05 | 35.92 |
| Triglycerides | rs676210 | G | A | 0.0734811 | 0.795 | 0.0024541 | 5.50E-197 | 2.03E-03 | 896.54 |
| Triglycerides | rs6792725 | A | G | 0.0154612 | 0.308 | 0.0022228 | 3.50E-12 | 1.10E-04 | 48.38 |
| Triglycerides | rs67981690 | A | G | -0.0297197 | 0.87 | 0.0029798 | 2.00E-23 | 2.26E-04 | 99.47 |
| Triglycerides | rs6800707 | C | G | -0.0297573 | 0.189 | 0.0025429 | 1.20E-31 | 3.10E-04 | 136.94 |
| Triglycerides | rs6805924 | G | T | -0.0109683 | 0.569 | 0.0020093 | 4.80E-08 | 6.76E-05 | 29.80 |
| Triglycerides | rs684773 | A | C | -0.0291315 | 0.233 | 0.0023514 | 3.00E-35 | 3.48E-04 | 153.49 |
| Triglycerides | rs6882076 | T | C | -0.0329854 | 0.366 | 0.0020649 | 1.90E-57 | 5.78E-04 | 255.18 |
| Triglycerides | rs6916318 | A | T | -0.0265583 | 0.469 | 0.0019943 | 1.80E-40 | 4.02E-04 | 177.35 |
| Triglycerides | rs6924805 | G | T | 0.0111417 | 0.412 | 0.0020236 | 3.70E-08 | 6.87E-05 | 30.32 |
| Triglycerides | rs696825 | C | T | 0.0203901 | 0.747 | 0.0022917 | 5.70E-19 | 1.79E-04 | 79.16 |
| Triglycerides | rs6999569 | A | G | 0.086226 | 0.529 | 0.0019842 | 1.00E-200 | 4.26E-03 | 1888.52 |
| Triglycerides | rs7000494 | G | C | -0.136371 | 0.97 | 0.005852 | 4.10E-120 | 1.23E-03 | 543.04 |
| Triglycerides | rs7077812 | T | C | -0.0140479 | 0.805 | 0.0025106 | 2.20E-08 | 7.10E-05 | 31.31 |
| Triglycerides | rs7134375 | C | A | 0.0171837 | 0.569 | 0.0020086 | 1.20E-17 | 1.66E-04 | 73.19 |
| Triglycerides | rs7138037 | G | C | -0.0191206 | 0.767 | 0.0023567 | 4.90E-16 | 1.49E-04 | 65.82 |
| Triglycerides | rs7140110 | T | C | -0.0281089 | 0.702 | 0.002182 | 5.70E-38 | 3.76E-04 | 165.95 |
| Triglycerides | rs71473777 | A | G | -0.0189191 | 0.88 | 0.0030806 | 8.20E-10 | 8.55E-05 | 37.72 |
| Triglycerides | rs71538127 | C | G | -0.017306 | 0.878 | 0.0030426 | 1.30E-08 | 7.34E-05 | 32.35 |
| Triglycerides | rs71603401 | A | G | -0.0263983 | 0.863 | 0.0029227 | 1.70E-19 | 1.85E-04 | 81.58 |
| Triglycerides | rs7186635 | A | G | -0.0117528 | 0.676 | 0.0021306 | 3.50E-08 | 6.90E-05 | 30.43 |
| Triglycerides | rs7215055 | A | G | -0.0391687 | 0.937 | 0.004108 | 1.50E-21 | 2.06E-04 | 90.91 |
| Triglycerides | rs7239575 | T | C | 0.0156502 | 0.51 | 0.0019948 | 4.30E-15 | 1.40E-04 | 61.55 |
| Triglycerides | rs7244 | G | A | -0.0152854 | 0.826 | 0.0026228 | 5.60E-09 | 7.70E-05 | 33.97 |
| Triglycerides | rs72555385 | A | G | -0.064988 | 0.951 | 0.004626 | 7.90E-45 | 4.47E-04 | 197.36 |
| Triglycerides | rs72691637 | G | A | 0.0152592 | 0.813 | 0.0025703 | 2.90E-09 | 7.99E-05 | 35.24 |
| Triglycerides | rs7274718 | G | A | -0.0158539 | 0.401 | 0.0020328 | 6.20E-15 | 1.38E-04 | 60.82 |
| Triglycerides | rs72801474 | G | A | 0.0307663 | 0.908 | 0.0034477 | 4.50E-19 | 1.81E-04 | 79.63 |
| Triglycerides | rs72836561 | C | T | -0.137064 | 0.969 | 0.0057308 | 2.00E-126 | 1.30E-03 | 572.03 |
| Triglycerides | rs72904737 | G | A | 0.0271857 | 0.913 | 0.0035244 | 1.20E-14 | 1.35E-04 | 59.50 |
| Triglycerides | rs729761 | T | G | -0.0177746 | 0.288 | 0.0022183 | 1.10E-15 | 1.46E-04 | 64.20 |
| Triglycerides | rs73025562 | G | A | -0.0138418 | 0.754 | 0.0023171 | 2.30E-09 | 8.09E-05 | 35.69 |
| Triglycerides | rs73243877 | A | G | -0.029039 | 0.832 | 0.0026603 | 9.70E-28 | 2.70E-04 | 119.16 |
| Triglycerides | rs7400002 | A | G | -0.0137859 | 0.769 | 0.0023708 | 6.10E-09 | 7.67E-05 | 33.81 |
| Triglycerides | rs7424120 | C | T | 0.0118514 | 0.398 | 0.0020343 | 5.70E-09 | 7.70E-05 | 33.94 |
| Triglycerides | rs75225803 | C | T | 0.0239222 | 0.912 | 0.0036105 | 3.50E-11 | 9.95E-05 | 43.90 |
| Triglycerides | rs75268115 | A | G | 0.0204249 | 0.915 | 0.0035862 | 1.20E-08 | 7.35E-05 | 32.44 |
| Triglycerides | rs75609851 | G | A | 0.198909 | 0.99 | 0.0100581 | 4.80E-87 | 8.86E-04 | 391.09 |
| Triglycerides | rs7631606 | T | G | 0.0138827 | 0.734 | 0.0022753 | 1.10E-09 | 8.44E-05 | 37.23 |
| Triglycerides | rs76895963 | T | G | 0.0874367 | 0.979 | 0.007709 | 8.10E-30 | 2.92E-04 | 128.64 |
| Triglycerides | rs7694869 | A | G | 0.0112396 | 0.376 | 0.0020608 | 4.90E-08 | 6.74E-05 | 29.75 |
| Triglycerides | rs77009508 | A | G | -0.044882 | 0.926 | 0.0038068 | 4.40E-32 | 3.15E-04 | 139.00 |
| Triglycerides | rs7704653 | A | G | -0.0156078 | 0.277 | 0.0022498 | 4.00E-12 | 1.09E-04 | 48.13 |
| Triglycerides | rs7714361 | A | C | -0.0136348 | 0.766 | 0.0023704 | 8.80E-09 | 7.50E-05 | 33.09 |
| Triglycerides | rs7735249 | C | G | -0.026895 | 0.887 | 0.0031656 | 2.00E-17 | 1.64E-04 | 72.18 |
| Triglycerides | rs78025076 | C | T | -0.0484707 | 0.98 | 0.0070538 | 6.30E-12 | 1.07E-04 | 47.22 |
| Triglycerides | rs78058190 | G | A | -0.0815632 | 0.95 | 0.0051157 | 3.20E-57 | 5.76E-04 | 254.20 |
| Triglycerides | rs7826246 | A | G | -0.0199647 | 0.9 | 0.0036417 | 4.20E-08 | 6.81E-05 | 30.06 |
| Triglycerides | rs78376313 | T | C | -0.048208 | 0.967 | 0.0057388 | 4.50E-17 | 1.60E-04 | 70.56 |
| Triglycerides | rs78484485 | G | A | 0.0761554 | 0.946 | 0.0044065 | 6.40E-67 | 6.77E-04 | 298.69 |
| Triglycerides | rs78588343 | G | A | 0.0157113 | 0.824 | 0.0026082 | 1.70E-09 | 8.23E-05 | 36.29 |
| Triglycerides | rs79153732 | C | T | -0.0769971 | 0.983 | 0.0075996 | 4.00E-24 | 2.33E-04 | 102.65 |
| Triglycerides | rs79192570 | G | A | 0.0250946 | 0.856 | 0.0028411 | 1.00E-18 | 1.77E-04 | 78.02 |
| Triglycerides | rs79287178 | G | A | -0.0502069 | 0.969 | 0.0060151 | 7.00E-17 | 1.58E-04 | 69.67 |
| Triglycerides | rs79357714 | A | G | 0.0288102 | 0.954 | 0.004773 | 1.60E-09 | 8.26E-05 | 36.43 |
| Triglycerides | rs8025505 | C | T | -0.0173098 | 0.743 | 0.0022896 | 4.00E-14 | 1.30E-04 | 57.16 |
| Triglycerides | rs80276949 | G | A | -0.0454698 | 0.977 | 0.006702 | 1.20E-11 | 1.04E-04 | 46.03 |
| Triglycerides | rs8126001 | C | T | 0.0163141 | 0.51 | 0.0020004 | 3.50E-16 | 1.51E-04 | 66.51 |
| Triglycerides | rs852388 | G | C | -0.0150872 | 0.789 | 0.0024604 | 8.70E-10 | 8.53E-05 | 37.60 |
| Triglycerides | rs863750 | C | T | -0.028859 | 0.396 | 0.0020404 | 2.00E-45 | 4.53E-04 | 200.05 |
| Triglycerides | rs867939 | G | A | 0.0138475 | 0.424 | 0.0020287 | 8.70E-12 | 1.06E-04 | 46.59 |
| Triglycerides | rs880315 | T | C | 0.0119226 | 0.66 | 0.0021088 | 1.60E-08 | 7.25E-05 | 31.96 |
| Triglycerides | rs921971 | T | C | -0.0157927 | 0.734 | 0.00226 | 2.80E-12 | 1.11E-04 | 48.83 |
| Triglycerides | rs935168 | G | A | -0.0142962 | 0.345 | 0.0020909 | 8.10E-12 | 1.06E-04 | 46.75 |
| Triglycerides | rs9376511 | A | G | 0.0151255 | 0.797 | 0.0024695 | 9.10E-10 | 8.51E-05 | 37.51 |
| Triglycerides | rs9436661 | T | G | 0.0775837 | 0.647 | 0.0020817 | 1.00E-200 | 3.14E-03 | 1389.00 |
| Triglycerides | rs9480889 | C | G | -0.0163419 | 0.217 | 0.0024144 | 1.30E-11 | 1.04E-04 | 45.81 |
| Triglycerides | rs954244 | C | G | -0.0152939 | 0.745 | 0.0022784 | 1.90E-11 | 1.02E-04 | 45.06 |
| Triglycerides | rs9561643 | A | C | -0.016521 | 0.685 | 0.0021486 | 1.50E-14 | 1.34E-04 | 59.12 |
| Triglycerides | rs9584870 | T | C | 0.0121656 | 0.634 | 0.0021075 | 7.80E-09 | 7.56E-05 | 33.32 |
| Triglycerides | rs9831084 | T | C | 0.0116305 | 0.538 | 0.0020001 | 6.10E-09 | 7.67E-05 | 33.81 |
| Triglycerides | rs998584 | C | A | -0.0398822 | 0.517 | 0.0019936 | 4.90E-89 | 9.07E-04 | 400.22 |
| A1, effect allele; A2, other allele | |  |  |  |  |  |  |  |  |
